# Novel peptaibiotics identified from *Trichoderma* clade *Viride*

**DOI:** 10.1007/s13659-025-00524-9

**Published:** 2025-08-01

**Authors:** Tamás Marik, Bonaya Gufu, Anusha Vishwanathula, Dóra Balázs, Ákos Rozsnyói, Gergő Terna, Fanni Kovács, Sándor Kocsubé, Mónika Varga, András Szekeres, Irina S. Druzhinina, Csaba Vágvölgyi, Tamás Papp, Chetna Tyagi, László Kredics

**Affiliations:** 1 Department of Biotechnology and Microbiology, Faculty of Science and Informatics, University of Szeged, Közép Fasor 52, 6726 Szeged, Hungary; 2 Doctoral School in Biology, Faculty of Science and Informatics, University of Szeged, Szeged, Hungary; 3 HUN-REN-SZTE Fungal Pathomechanisms Research Group, University of Szeged, Szeged, Hungary; 4 Royal Botanic Gardens, Kew, Kew Green, Richmond, Surrey, TW9 3AE UK

**Keywords:** *Trichoderma*, Clade *Viride*, Peptaibol, Lipopeptaibol, Peptaibiome, HPLC–MS, Accelerated MD simulations

## Abstract

This study focuses on the peptaibiome produced by different species of *Trichoderma* belonging to clade *Viride*: *T. koningii* SZMC 28387 (CBS 979.70), *T.* cf*. strigosellum* SZMC 28007 (TUCIM 4886/IQ 191), *T.* cf*. dorothopsis* SZMC 28390 (TUCIM 416/TUB F-597), *T.* cf*. strigosellum* SZMC 28391 (TUCIM 423/DAOM 230018), *T. atroviride* SZMC 28748 (IMI 206040), *T. hamatum* SZMC 28747 (TUCIM 2730) and *T.* cf*. dorothopsis* SZMC 28005 (TUCIM 4882/IQ 11). We were able to identify new compounds with similarity to already known groups of peptaibiotics, as well as completely newly discovered compounds using high-performance liquid chromatography (HPLC) -mass spectrometry (MS). From the 367 peptaibiotics identified, 216 are peptaibols and 111 are lipopeptaibols. Out of all peptaibols, 55 are previously known, while 161 are newly discovered. The new peptaibol subgroups Strigosellin A, B and Dorothopsin A, B are introduced. Furthermore, besides 38 previously known lipopeptaibols, 73 new lipopeptaibol sequences, named Lipostrigosellins and Lipohamatins are also reported. In addition, 41 peptaibol-like compounds with unusual C-terminus were also found. Out of the 7 strains examined, 5 produced both peptaibols and lipopeptaibols, while 2 only peptaibols. The well-known compound, Trikoningin KA V (TRK-V) also produced by *T. koningii* SZMC 28387 (CBS 979.70), was studied for its folding dynamics using accelerated molecular dynamics simulations (aMD) for understanding the plausible three-dimensional structures adopted by these peptaibols of clade *Viride*. We observed a propensity to form kinked, right-handed helical structures when simulated in an aqueous environment.

**Graphical Abstract:**

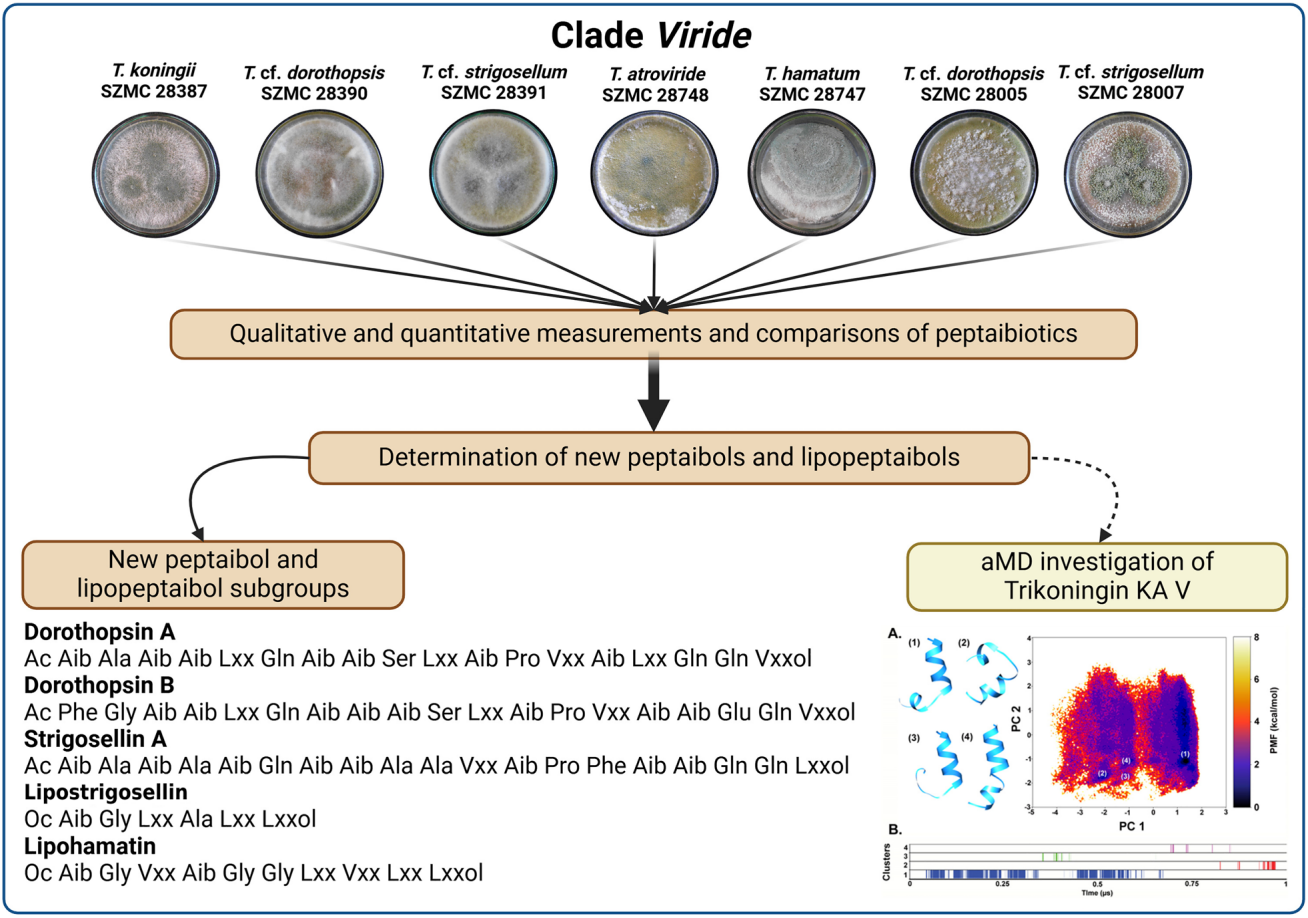

**Supplementary Information:**

The online version contains supplementary material available at 10.1007/s13659-025-00524-9.

## 1. Introduction

Clade *Viride* stands as one of the most extensive and varied group within the genus *Trichoderma*. Members of this clade exhibit a broad range of habitats and are found across diverse geographic regions [1]. They play significant roles in various sectors including industry, agriculture, and medicine [2]. One notable example is *T. viride*, which effectively combats decay in Obeche wood (*Triplochiton sceleroxylon*) caused by *Gloeophyllum* sp. and *G. sepiarium* through mycoparasitism and nutrient competition [3]. Certain species within this clade, such as *T. asperellum* and *T. atroviride*, were thought to be problematic for commercial mushroom cultivation, but eventually, they were not found to cause green mould infection of mushrooms even though they appeared in mushroom growing substrates [4]. *Trichoderma* green mould affects both *Agaricus bisporus* and *Pleurotus ostreatus,* two of the most important mushroom crops, leading to inquiries into the particular strains implicated and their preference for substrates. Green mould agents were described as *T. aggressivum*, *T. pleuroti* and *T. pleuroticola* belonging to clade *Harzianum* of the genus *Trichoderma*, while no green mould infections could be attributed to *T. asperellum* and *T. atroviride* [4–7]. Morphological characteristics of clade *Viride* representatives are consistent slow growth rate, globose to subglobose and strongly warted conidia, often solitary conidiophores and hooked phialides [8]. However, these morphological characteristics are not sufficient for exact species-level identification due to the overlap of morphology of *Trichoderma* species, and therefore, they need to be combined with molecular characterization. The mainly used one being integration of phenetic and phylogenetic characters, which is emphasized for species recognition, with a focus on concordance between both approaches, the translation elongation factor 1α (*tef1α*), internal transcribed spacer 1 and 2 (ITS1 and 2) regions, as well as, sequence analysis of fragments RNA polymerase II gene (*rpb2*) genes with subsequent phylogenetic analysis. These altogether are essential for characterizing new species and understanding the genetic diversity within the clade [9].

Genus *Trichoderma* is recognized for generating secondary metabolites, including polyketides, alkaloids, terpenoids, non-ribosomally biosynthesized peptides, and mixed biogenesis metabolites [10]. Currently, extensive research on this genus is underway due to the production of bioactive peptaibiotics. Besides *Trichoderma* species, members of other closely related genera like *Emericellopsis* and *Gliocladium* are also significant producers of peptaibiotics [11]. The analytical exploration of peptaibiotics is referred to as peptaibiomics [12]. Peptaibiome is described as the entirety of fungal peptides which contain the amino acid residue α-aminoisobutyric acid (Aib) [12, 13]. These acyclic peptides are synthesized by non-ribosomal peptide synthetase (NRPS) enzymes [14].

In this study, peptaibols and lipopeptaibols were identified out of the 5 groups of peptaibiotics, which were categorized based on their length and chemical structures [12]. Peptaibols constitute a class of linear antibiotic peptides characterized by their length, usually ranging from five to twenty amino acids. Notable features of peptaibols include a high occurrence of non-proteinogenic C-alpha tetrasubstituted amino acids [15], such as the relatively uncommon isovaline (Iva) or the achiral Aib. Typically, the N-terminal amino acid of peptaibols is acylated, while the C-terminus consists of a β-amino alcohol. Among the most prevalent types of β-amino alcohols at the C-terminus are isoleucinol, phenylalaninol, valinol, and leucinol (Leuol). These compounds represent the most extensive category within peptaibiotics and several peptaibols are already known to be produced by members of clade *Viride* [16]. Lipopeptaibols represent naturally occurring short peptides possessing antimicrobial properties [17]. They are distinguished by a lipophilic acyl chain at the N-terminus, a significant presence of turn/helix forming Aib, and a 1,2-amino alcohol at the C-terminus. These peptides typically consist of 6 to 11 amino acid residues and feature fatty acyl moieties ranging from 8 to 15 carbon atoms, which is shorter compared to the nonlipidated peptaibols that typically contain 11 to 20 amino acid residues [17, 18]. Due to the presence of a lipophilic N-terminal group, these peptides are known as lipopeptaibols. They have been extracted from several fungal cultures including *T. koningii* (trikoningins) and *T. viride* (trichodecenins) from clade *Viride*, as well as *T. longibrachiatum* (trichogins), *T. polysporum* (trichopolyns), *Tolypocladium geodes* (antibiotics LP 237), and *Mycogone rosea* (helioferins) [17, 18]. It is then imperative that more unknown structures of peptaibiotics should be studied and analyzed to continue the growth of the scientific knowledge and industrial application of this unique and promising group of secondary metabolites.

## Materials and methods

### Fungal strains, media, culture conditions

*Trichoderma* strains were selected from the TU Collection of Industrially Important Microorganisms (TUCIM), Vienna, Austria and deposited at Szeged Microbiology Collection (SZMC) (Table 1). The selected strains were cultivated on yeast-glucose agar medium enriched with malt extract (3 g/L yeast extract, 10 g/L glucose, 20 g/L agar, and 50 mL of 20% liquid malt extract solution). To increase peptaibol production, the strains were cultivated on malt extract agar (MEA) medium containing 150 mL (20%) malt extract solution, 3 g/L soybean peptone, and 15 g/L agar. Incubation was carried out for 7 days at 25 ℃, and mycelial growth was subsequently observed.

**Table 1:** *Trichoderma* strains selected for this study. The re-identification was based on the sequence analysis of the *tef1α* region

SZMC code	TUCIM code	Other code	Species	Reference	Re-Identified species
28387	201	CBS 979.70	*T.* sp.	[19]	*T. koningii*
28390	416	TUB F-597	*T. taiwanense*	[20]	*T.* cf. *dorothopsis*
28391	423	DAOM 230018	*T. strigosellum*	[21]	*T.* cf.* strigosellum*
28748	1680	IMI 206040	*T. atroviride*	[22]	*T. atroviride*
28747	2730	−	*T. fertile*	−	*T. hamatum*
28005	4882	IQ 11	*T. koningiopsis*	[23]	*T.* cf. *dorothopsis*
28007	4886	IQ 191	*T.* cf.* strigosellum*	[23]	*T.* cf.* strigosellum*

### Extraction of peptaibols

Following the incubation period, peptaibols were extracted from the *Trichoderma* cultures using a solution composed of chloroform and methanol in a 2/1 (*v/v*) ratio. The resulting mixture was subjected to evaporation using an IKA RV 10 rotatory evaporator (IKA Works, USA) until complete dryness. The resultant dry mass was reconstituted in 1 mL of methanol and thoroughly mixed. Subsequently, the samples underwent centrifugation for 15 min (Heraeus Fresco 17, Thermo Scientific, CA, USA) to eliminate insoluble particles, and the clarified samples were stored at − 20 ℃ for further analysis.

### Species reidentification

To cultivate mycelia of the *Trichoderma* strains, liquid medium was prepared in Eppendorf tubes on yeast-glucose agar medium enriched with malt extract mentioned above. The tubes were incubated for 2 days at 25 ℃. After incubation, approximately 100 mg of fungal tissue was introduced into sterile Eppendorf tubes. Glass beads with particle sizes of 0.4–0.6 mm and 0.9–0.15 mm were added to the tubes, and liquid nitrogen was employed for cell disruption. The tubes were placed in a cell disruptor for 4 min, and this process was repeated three times.

Following the initial procedures, DNA extraction was carried out using the E.Z.N.A.^®^ Fungal DNA Mini Kit in accordance with the manufacturer's instructions. Subsequent to DNA purification, the *tef1α* region was amplified through polymerase chain reaction (PCR). The PCR master mixture for each sample (1 μL) consisted of 2 μL of Dream Taq buffer, 2 μL of dNTP mix, 4–4 μL of 1 μM *tef1α* primers (forward: 5’-CATCGAGAAGTTCGAGAAGG-3’ and reverse: 5’-AACTTGCAGGCAATGTGG-3’), 7 μL of water, and 0.1 μL of Dream Taq DNA polymerase. The PCR cycle was conducted using the MJ MiniTM Personal Thermal Cycler (BIO-RAD) following these steps: an initial step at 94 ℃ for 5 min; cycling steps at 94 ℃ for 30 s (denaturation), 57 ℃ for 30 s (annealing), and 72 ℃ for 30 s (elongation), repeated for 40 cycles; and a final elongation step at 72 ℃ for 4 min. Agarose gel electrophoresis was performed to check the success of the reaction on 1% agarose gel (35 mL TAE buffer, 0.35 g agarose, 2 μL ethidium bromide). Two μL of DNA samples were applied with 2 μL of bromophenol blue. The run time for gel electrophoresis was 30 min at 90 V. The DNA fragments were sent for sequence analysis to an external service (Eurofins Scientific). Sequences were analyzed by using Finch TV 1.4.0 (Geospiza Inc.) and NCBI Nucleotide BLAST softwares (https://blast.ncbi.nlm.nih.gov/Blast.cgi).

Multiple sequence alignment of partial *tef1α* sequences (provided in the supplementary file) was made by PAGAN v1.53 [24] with default settings. The final dataset consisted of two partitions for the intron and exon regions. For both partitions, the best fitting model was TrN + G4, determined by using ModelTest-NG v0.1.7 [25], based on the Bayesian Information Criterion (BIC). Maximum likelihood analysis with the selected models was carried out by RAxML-NG v1.2.2 [26], with 1000 bootstrap replicates.

### Analytical procedures for peptaibols

The analysis of crude peptaibol extracts was conducted using high-performance liquid chromatography-mass spectrometry (HPLC–MS) based on a method previously described by Marik et al. [27]. For the analysis, an Orbitrap (QExactive Plus, Thermo Scientific, CA, USA) MS was used with a heated electrospray ion source (HESI) in positive mode coupled with an Dionex Ultimate 3000 (Thermo Scientific, CA, USA) HPLC containing a quaternary pump, a vacuum degasser, an autosampler, and a column heater. The instruments were controlled by the Xcalibur 4.2 Software (Thermo Scientific, CA, USA). The separation was achieved using a Purospher Star RP-15 HPLC column (100 × 2.1, 2 μm), while the solvent A consisted of H_2_O/MeOH/MeCN (8/1/1, *v/v*) with 5 mM ammonium acetate and 0.1% (*v/v*) acetic acid and solvent B comprised of acetonitrile/methanol (1/1, *v/v*) with 5 mM ammonium acetate and 0.1% (*v/v*) acetic acid. The flow rate was set to 0.2 mL/min, and the gradient program for Solvent B was as follows: 0 min—65%, 2 min—65%, 42 min—80%, 54 min—90%, 55 min—95%, 58 min—95%, 58.5—65% and 62.5—65%. The column temperature was maintained at 30 ℃, and the injection volume was 3 μL. The HESI parameters were as follows: spray voltage – 4.5 kV, sheath gas flow rate − 30 arbitrary units, aux gas flow rate − 12 arbitrary units, capillary temperature − 300 ℃, aux gas heater − 300 ℃. The acquisition mode was Full-MS-ddMS^2^. Full-MS parameters were: resolution − 70,000 at *m/z* 200, AGC target − 3e6, maximum injection time − 100 ms, scan range − 200–2000 m*/z*. The ddMS^2^ parameters: fixed first scan at *m/z* 80, resolution 17500 at *m/z* 200, AGC target − 1e6, maximum injection time − 50 ms, isolation window − 1 *m/z*, collision energy − 25 NCE. Loop count was set to 5.

### Determination and nomenclature of peptaibol sequences

Peptaibols typically undergo fragmentation between Aib − Pro residues already in the ion source resulting in the generation of b- and y-ions. The identification of a specific peptaibol compound relies on its retention times (Rt) and on the mass of its b- and y-fragment ions as well as the mass of the hydrogen adduct of the primary mass of the compound [M + H]^+^ (b-ion + y-ion). During full scan measurements, the mass of the protonated molecular ion coexists with other adduct ions such as the primary mass and sodium adduct [M + Na]^+^, the primary mass and 2 hydrogen adducts [M + 2H]^2+^, the primary mass and 2 sodium adducts [M + 2Na]^2+^, and the primary mass and hydrogen and sodium adducts [M + H + Na]^2+^. In the longer peptaibols (16–19 residues), the fragmentation typically occurred between the centrally located Aib − Pro residues, leading to the formation of b_11-12_ and y_5-7_ ions. The analysis of MS^2^ results from b- and y-ion fragments provided insights into the complete sequences of the compounds (Supplementary Fig. 1).

Lipopeptaibols were identified based on the MS^2^ fragmentation of [M + H]^+^. The whole sequence could be observed on the MS^2^ spectra.

To assess the novelty of the obtained sequences, an initial comparison was made using the ‘Comprehensive Peptaibiotics Database’ [28]. This database is not available online since 2017, however, Prof. Rainer Schuhmacher kindly provided the offline version of the Peptaibiotics Database including the peptaibiotics records till 2017. Consequently, to gather more information and conduct a comprehensive comparison, an additional online literature search was conducted using PubMed (https://pubmed.ncbi.nlm.nih.gov/) with the keywords ‘peptaibol’ or ‘lipopeptaibol’.

The quantity of peptaibols were defined based on the integration of the peaks under the major y-ions originated from the decomposition of the Aib–Pro bond in the ion source on the extracted ion chromatograms (EIC) of the full scan MS measurement. Adding the separately integrated peaks resulted in the total peptaibol production of the strain, out of which, percentages were calculated for each compound. The quantity of lipopeptaibols was defined based on the integration of the peaks under the [M + H]^+^ on the EIC of the full scan MS measurement. Percentages of each compound were calculated as mentioned above.

The known peptaibols and lipopeptaibols were named using the closest identified compounds attached to a Roman numeral based on their elution order. The completely novel sequences were named after the producer species attached to a Roman numeral based on their elution order.

### aMD simulations to reveal folding dynamics of Trikoningin KA V (TRK-V)

Trikoningin KA V was selected because the isotype of Vxx and Lxx residues of this compound was determined by Goulard et al. [29]. For non-standard amino acid residues, Aib and Leuol, the partial charges and force fields were calculated using a previously developed method reported by Tyagi et al. [30]. The unfolded peptide topology was prepared using the ‘tleap’ module of AmberTools20 using the Amberff19SB force field and solvated in TIP3P water solvent model [31]. The peptide solvation added 2324 water residues with the periodic box size of 45.96 × 44.35 × 46.88 Å and volume of 95587 Å^3^. The system preparation, minimization and equilibration steps were carried out as described in Tyagi et al. [32]. To calculate boost parameters, total number of atoms in the system (Natoms = 19467), number of residues (Nres = 20) and coefficients a1, a2 (4.5) and b1, b2 (0.20) were utilized. The aMD simulation was run for 1000 ns or 1 µs in total. The resulting trajectory files were prepared by the removal of water molecules. Dihedral angles were calculated for every residue from each frame of the simulation and the distribution was reweighted using Maclaurin series expansion.

## Results and discussion

### Reidentification of the investigated *Trichoderma* strains

**Fig. 1:**
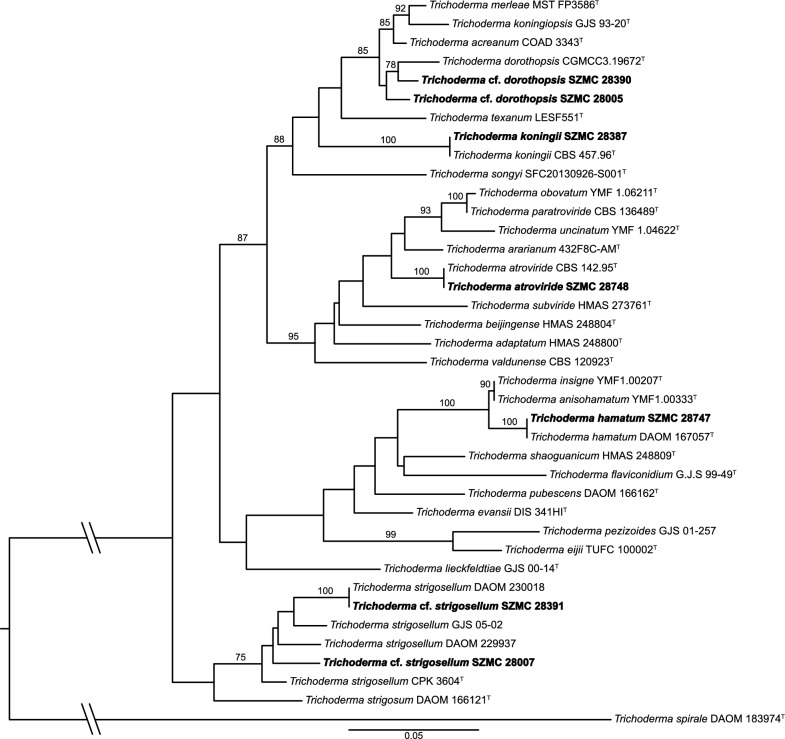
Phylogenetic tree of the examined strains from clade *Viride* based on their partial *tef1α* sequences

The examined isolates were assigned to species based on sequence analysis of partial *tef1α* sequences ( provided in the supplementary file). Figure 1 shows the phylogenetic relation between the strains investigated in this article and the type strains of closely related species. *T. koningii* SZMC 28387 and *T. atroviride* SZMC 28748 showed 100% identity with their type strains. Strain SZMC 28747 originally was identified as *T. fertile*, however, it showed 100% identity with the type strains of *T. hamatum*. *T.* cf*. strigosellum* SZMC 28391 and SZMC 28007 could not be identified to species level, therefore, cf. *strigosellum* was introduced. *T. taiwanense* SZMC 28390 and *T.* cf*. dorothopsis* SZMC 28005 did not match with their type strains either, but were similar to *T. dorothopsis* type strain and were named as *T.* cf. *dorothopsis* SZMC 28390 and *T.* cf. *dorothopsis* SZMC 28005.

### Peptaibiome of the investigated *Trichoderma* strains

Out of the 7 strains investigated, (Table 1), 5 produced both peptaibols and lipopeptaibols (*T.* cf*. strigosellum SZMC 28391* and SZMC 28007, *T. koningii* SZMC 28387, *T. hamatum* SZMC 28747, and *T.* cf*. dorothopsis* SZMC 28005), while *T.* cf. *dorothopsis* SZMC 28390 and *T. atroviride* SZMC 28748 did not produce any lipopeptaibols, their peptaibiome consisted of exclusively SF1 peptaibols (Table 2, Supplementary Fig. 1). The two *T.* cf. *strigosellum* strains produced ∼40/60% ratio of peptaibols/lipopeptaibols *T. koningii* SZMC 28387 and *T.* cf*. dorothopsis* SZMC 28005 produced one third ratio of peptaibols of the peptaibiome, while *T. hamatum* SZMC 28747 produced only one fourth of peptaibols of the peptaibiome (Table 2). Based on their length, lipopeptaibols could be categorised into the short (6–7-residue long), medium (10–11- residue long) or long (15-residue long) groups. The most amount of short lipopeptaibols was produced by *T.* cf*. strigosellum* SZMC 28007 with 61.35% of the peptaibiome, while the greatest number of long lipopeptaibols were produced by *T. hamatum* SZMC 28747 with 40.36% of the peptaibiome. The amount of medium lipopeptaibols was ranging from 30 to 65% of the total production (Table 2).

**Table 2:** Ratio of peptaibiotics (peptaibiome) produced by the members of clade *Viride* investigated in this study

Species	SZMC	Peptaibols	Short lipopeptaibols	Medium lipopeptaibols	Long lipopeptaibols
*T.* cf.* strigosellum*	28391	39.25%	60.25%	−	−
*T.* cf.* strigosellum*	28007	39.35%	61.35%	−	−
*T. koningii*	28387	32.08%	2.78%	65.14%	−
*T.* cf*. dorothopsis*	28390	100.00%	−	−	−
*T. atroviride*	28748	100.00%	−	−	−
*T. hamatum*	28747	23.62%	3.71%	32.36%	40.36%
*T.* cf*. dorothopsis*	28005	33.25%	−	53.15%	13.60%

### Peptaibol profiles of the investigated *Trichoderma* strains

Tables 3 and 4 were obtained from the chromatographic and MS analysis of the investigated *Trichoderma* strains and show the subfamily 1 (SF-1) peptaibols of the investigated *Trichoderma* strains. The diagnostic fragment ions resulted by MS^2^ fragmentation are collected in Supplementary Table 1.

**Table 3:** SF1 peptaibols produced by *Trichoderma* strains from clade *Viride*

Peptaibol	[M + H]^+^	b_13_	y_7_	RT (min)	Area (%) total peptaibiome	Area (%) peptaibol	R	R1	R2	R3	R4	R5	R6	R7	R8	R9	R10	R11	R12	R13	R14	R15	R16	R17	R18	R19
*T.* cf*. strigosellum* SZMC 28391																										
**Strigaibol-like I**	1893.12	1152.66	740.46	24.66	0.38%	0.96%	Ac	Aib	Ser	Aib	Aib	Lxx	Gln	Aib	Aib	Aib	Ser	Vxx	Aib	Pro	Vxx	Aib	Aib	Gln	Gln	Lxxol
**Strigaibol-like II**	1907.14	1166.68	740.46	24.66	0.33%	0.85%	Ac	*Vxx*	Ser	Aib	Aib	Lxx	Gln	Aib	*Ala*	Aib	Ser	Lxx	Aib	Pro	Vxx	Aib	Aib	Gln	Gln	Lxxol
**Strigaibol-like III**	1893.13	1166.68	726.45	24.9	0.03%	0.08%	Ac	*Vxx*	Ser	Aib	Aib	Lxx	Gln	Aib	*Ala*	Aib	Ser	Lxx	Aib	Pro	Vxx	Aib	*Ala*	Gln	Gln	Lxxol
**Strigaibol-like IV**	1907.14	1180.69	726.45	25.53	0.10%	0.24%	Ac	*Vxx*	Ser	Aib	Aib	Lxx	Gln	Aib	Aib	Aib	Ser	Lxx	Aib	Pro	Vxx	Aib	Aib	Gln	Gln	*Vxxol*
**Strigaibol-**like V	1907.14	1166.68	740.46	26.67	2.02%	5.14%	Ac	Aib	Ser	Aib	Aib	Lxx	Gln	Aib	Aib	Aib	Ser	Lxx	Aib	Pro	Vxx	Aib	Aib	Gln	Gln	Lxxol
**Strigaibol-like VI**	1907.14	1166.68	740.46	27.53	2.53%	6.44%	Ac	*Vxx*	*Ser*	Aib	Aib	Lxx	Gln	Aib	Aib	Aib	Ser	*Vxx*	Aib	Pro	Vxx	Aib	Aib	Gln	Gln	Lxxol
**Strigaibol-like VII**	1893.13	1166.68	726.45	27.75	0.09%	0.23%	Ac	*Vxx*	*Ser*	Aib	Aib	Lxx	Gln	Aib	Aib	Aib	Ser	*Vxx*	Aib	Pro	Vxx	Aib	*Ala*	Gln	Gln	Lxxol
**Strigaibol-like VIII**	1877.13	1150.68	726.45	28.2	0.14%	0.35%	Ac	Aib	Ala	Aib	Aib	Lxx	Gln	Aib	Aib	Aib	Ser	Lxx	Aib	Pro	Vxx	Aib	Aib	Gln	Gln	*Vxxol*
**Strigaibol-like IX**	1891.14	1150.68	740.46	28.4	0.27%	0.68%	Ac	Aib	Ala	Aib	Aib	Lxx	Gln	Aib	Aib	Aib	Ser	Lxx	Aib	Pro	Vxx	Aib	Aib	Gln	Gln	Lxxol
**Strigaibol-like X**	1877.13	1136.67	740.46	29.66	1.70%	4.34%	Ac	Aib	Ala	Aib	Aib	Lxx	Gln	Aib	Aib	Aib	Ser	Vxx	Aib	Pro	Vxx	Aib	Aib	Gln	Gln	Lxxol
**Strigaibol-like XI**	1907.14	1166.68	740.46	29.66	0.22%	0.57%	Ac	Aib	Ser	Aib	Aib	Lxx	Gln	Aib	Aib	Aib	Ser	Lxx	Aib	Pro	Vxx	Aib	Aib	Gln	Gln	Lxxol
**Strigaibol-like XII**	1921.15	1180.69	740.46	29.66	14.19%	36.15%	Ac	*Vxx*	Ser	Aib	Aib	Lxx	Gln	Aib	Aib	Aib	Ser	Lxx	Aib	Pro	Vxx	Aib	Aib	Gln	Gln	Lxxol
**Strigaibol-like XIII**	1907.14	1166.68	740.46	30.52	1.01%	2.58%	Ac	*Vxx*	*Ser*	Aib	Aib	Lxx	Gln	Aib	Aib	Aib	Ser	Vxx	Aib	Pro	Vxx	Aib	Aib	Gln	Gln	Lxxol
**Strigaibol-like XIV**	1891.14	1150.68	740.46	32.31	10.83%	27.59%	Ac	Aib	Ala	Aib	Aib	Lxx	Gln	Aib	Aib	Aib	Ser	Lxx	Aib	Pro	Vxx	Aib	Aib	Gln	Gln	Lxxol
**Strigaibol-like XV**	1905.16	1164.7	740.46	32.31	0.83%	2.11%	Ac	*Vxx*	Ser	Aib	Aib	Lxx	Gln	Aib	Aib	Aib	*Ala*	Lxx	Aib	Pro	Vxx	Aib	Aib	Gln	Gln	Lxxol
**Strigaibol-like XVI**	1921.15	1180.69	740.46	32.31	0.01%	0.02%	Ac	Vxx	Ser	Aib	Aib	Lxx	Gln	Aib	Aib	Aib	Ser	Lxx	Aib	Pro	Vxx	Aib	Aib	Gln	Gln	Lxxol
**Strigaibol-like XVII**	1877.13	1136.67	740.46	33.98	0.34%	0.85%	Ac	Aib	Ala	Aib	Aib	Lxx	Gln	Aib	Aib	Aib	Ser	Vxx	Aib	Pro	Vxx	Aib	Aib	Gln	Gln	Lxxol
**Strigaibol-like XVIII**	1875.15	1134.69	740.46	34.86	0.41%	1.06%	Ac	Aib	Ala	Aib	Aib	Lxx	Gln	Aib	Aib	Aib	Ala	Lxx	Aib	Pro	Vxx	Aib	Aib	Gln	Gln	Lxxol
**Strigaibol-like XIX**	1905.16	1164.7	740.46	34.86	0.63%	1.61%	Ac	Aib	Aib	Aib	Aib	Lxx	Gln	Aib	Aib	Aib	Ser	Lxx	Aib	Pro	Vxx	Aib	Aib	Gln	Gln	Lxxol
** Strigaibol-like XX**	1891.14	1150.68	740.46	36.1	2.83%	7.20%	Ac	Aib	Ala	Aib	Aib	Lxx	Gln	Aib	Aib	Aib	Ser	Lxx	Aib	Pro	Vxx	Aib	Aib	Gln	Gln	Lxxol
**Strigaibol-like XXI**	1919.17	1178.71	740.46	37.55	0.37%	0.94%	Ac	Aib	Vxx	Aib	Aib	Lxx	Gln	Aib	Aib	Aib	Ser	Lxx	Aib	Pro	Vxx	Aib	Aib	Gln	Gln	Lxxol
*T. koningii* SZMC 28387																										
**Trikoningin KA-like I**	1863.12	1108.64	754.48	31.31	0.02%	0.06%	Ac	Aib	Gly	Ala	Aib	Vxx	Gln	Aib	Aib	Aib	Ser	Lxx	Aib	Pro	Vxx	Aib	Vxx	Gln	Gln	Lxxol
**Trikoningin KA-like II**	1864.10	1108.64	755.46	31.88	0.02%	0.06%	Ac	Aib	Gly	Ala	Aib	Vxx	Gln	Aib	Aib	Aib	Ser	Lxx	Aib	Pro	Vxx	Aib	Vxx	*Glu*	Gln	Lxxol
**Trikoningin KA-like III**	1878.11	1122.65	755.46	31.88	0.01%	0.02%	Ac	Aib	Gly	Ala	Aib	Lxx	Gln	Aib	Aib	Aib	Ser	Lxx	Aib	Pro	Vxx	Aib	Vxx	*Glu*	Gln	Lxxol
**Trikoningin KA-like IV**	1877.13	1108.64	768.49	32.89	0.09%	0.29%	Ac	Aib	Gly	*Gly*	Aib	Lxx	Gln	Aib	Aib	Aib	Ser	Lxx	Aib	Pro	Vxx	Aib	Lxx	Gln	Gln	Lxxol
**Trikoningin KA-like V**	1907.28	1138.79	768.49	32.89	0.22%	0.70%	Ac	Aib	Gly	*Ser*	Aib	Lxx	Gln	Aib	Aib	Aib	Ser	Lxx	Aib	Pro	Vxx	Aib	Lxx	Gln	Gln	Lxxol
**Trikoningin KA-like VI**	1878.12	1108.64	769.48	33.42	0.09%	0.28%	Ac	Aib	Gly	Ala	Aib	Lxx	Gln	Aib	Aib	Aib	Ser	Lxx	Aib	Pro	Vxx	Aib	Lxx	*Glu*	Gln	Lxxol
**Trikoningin KA-like VII**	1908.27	1138.79	769.48	33.42	0.15%	0.48%	Ac	Aib	Gly	*Ser*	Aib	Lxx	Gln	Aib	Aib	Aib	Ser	Lxx	Aib	Pro	Vxx	Aib	Lxx	*Glu*	Gln	Lxxol
**Trikoningin KA-like VIII**	1847.12	1092.64	754.48	34.01	0.01%	0.02%	Ac	Aib	Gly	*Gly*	Aib	Lxx	Gln	Aib	Aib	Aib	*Ala*	Lxx	Aib	Pro	Vxx	Aib	Vxx	Gln	Gln	Lxxol
**Trikoningin KA-like IX**	1863.12	1108.64	754.48	34.01	0.05%	0.16%	Ac	Aib	Gly	Ala	Aib	Vxx	Gln	Aib	Aib	Aib	Ser	Lxx	Aib	Pro	Vxx	Aib	Vxx	Gln	Gln	Lxxol
**Trikoningin KA-like X**	1877.13	1122.65	754.48	34.01	0.03%	0.08%	Ac	Aib	Gly	Ala	Aib	Lxx	Gln	Aib	Aib	Aib	Ser	Lxx	Aib	Pro	Vxx	Aib	Vxx	Gln	Gln	Lxxol
**Trikoningin KA-like XI**	1877.13	1108.64	768.49	34.76	0.13%	0.40%	Ac	Aib	Gly	Ala	Aib	Vxx	Gln	Aib	Aib	Aib	Ser	Lxx	Aib	Pro	Vxx	Aib	Lxx	Gln	Gln	Lxxol
**Trikoningin KA-like XII**	1907.14	1138.65	768.49	34.76	0.19%	0.60%	Ac	Aib	Gly	*Ser*	Aib	Lxx	Gln	Aib	Aib	Aib	Ser	Lxx	Aib	Pro	Vxx	Aib	Lxx	Gln	Gln	Lxxol
**Trikoningin KA-like XIII**	1863.12	1108.64	754.48	34.97	0.01%	0.02%	Ac	Aib	Gly	Ala	Aib	Vxx	Gln	Aib	Aib	Aib	Ser	Lxx	Aib	Pro	Vxx	Aib	Vxx	Gln	Gln	Lxxol
**Trikoningin KA-like XIV**	1877.13	1122.65	754.48	34.97	0.00%	0.01%	Ac	Aib	Gly	Ala	Aib	Lxx	Gln	Aib	Aib	Aib	Ser	Lxx	Aib	Pro	Vxx	Aib	Vxx	Gln	Gln	Lxxol
**Trikoningin KA-like XV**	1878.12	1108.64	769.48	35.36	0.17%	0.52%	Ac	Aib	Gly	Ala	Aib	Vxx	Gln	Aib	Aib	Aib	Ser	Lxx	Aib	Pro	Vxx	Aib	Lxx	*Glu*	Gln	Lxxol
**Trikoningin KA-like XVI**	1908.27	1138.79	769.48	35.36	0.16%	0.50%	Ac	Aib	Gly	*Ser*	Aib	Lxx	Gln	Aib	Aib	Aib	Ser	Lxx	Aib	Pro	Vxx	Aib	Lxx	*Glu*	Gln	Lxxol
**Trikoningin KA-like XVII**	1878.11	1122.65	755.46	35.5	0.01%	0.02%	Ac	Aib	Gly	Ala	Aib	Lxx	Gln	Aib	Aib	Aib	Ser	Lxx	Aib	Pro	Vxx	Aib	*Vxx*	*Glu*	Gln	Lxxol
**Trikoningin KA-like XVIII**	1877.13	1108.64	768.49	35.88	0.05%	0.17%	Ac	Aib	Gly	Ala	Aib	Vxx	Gln	Aib	Aib	Aib	Ser	Lxx	Aib	Pro	Vxx	Aib	Lxx	Gln	Gln	Lxxol
**Trikoningin KA-like XIX**	1891.14	1122.65	768.49	35.88	0.13%	0.41%	Ac	Aib	Gly	Ala	Aib	Lxx	Gln	Aib	Aib	Aib	Ser	Lxx	Aib	Pro	Vxx	Aib	Lxx	Gln	Gln	Lxxol
**Trikoningin KA-like XX**	1878.12	1108.64	769.48	36.34	0.69%	2.15%	Ac	Aib	Gly	Ala	Aib	Vxx	Gln	Aib	Aib	Aib	Ser	Lxx	Aib	Pro	Vxx	Aib	Lxx	*Glu*	Gln	Lxxol
**Trikoningin KA-like XXI**	1892.13	1122.65	769.48	36.34	0.05%	0.17%	Ac	Aib	Gly	Ala	Aib	Lxx	Gln	Aib	Aib	Aib	Ser	Lxx	Aib	Pro	Vxx	Aib	Lxx	*Glu*	Gln	Lxxol
**Trikoningin KA-like XXII**	1861.13	1092.64	768.49	37.36	0.03%	0.09%	Ac	Aib	Gly	Ala	Aib	Vxx	Gln	Aib	Aib	Aib	*Ala*	Lxx	Aib	Pro	Vxx	Aib	Lxx	Gln	Gln	Lxxol
**Trikoningin KA-like XXIII**	1877.13	1108.64	768.49	37.36	0.20%	0.63%	Ac	Aib	Gly	Ala	Aib	Lxx	Gln	Aib	*Ala*	Aib	Ser	Lxx	Aib	Pro	Vxx	Aib	Lxx	Gln	Gln	Lxxol
**Trikoningin KA-like XXIV**	1847.12	1092.64	754.48	37.46	0.04%	0.13%	Ac	Aib	Gly	Ala	Aib	Vxx	Gln	Aib	Aib	Aib	*Ala*	Lxx	Aib	Pro	Vxx	Aib	Vxx	Gln	Gln	Lxxol
**Trikoningin KA-like XXV**	1863.12	1108.64	754.48	37.46	0.22%	0.69%	Ac	Aib	Gly	Ala	Aib	Vxx	Gln	Aib	Aib	Aib	Ser	Lxx	Aib	Pro	Vxx	Aib	Vxx	Gln	Gln	Lxxol
**Trikoningin KA-like XXVI**	1877.13	1122.65	754.48	37.46	0.40%	1.26%	Ac	Aib	Gly	Ala	Aib	Lxx	Gln	Aib	Aib	Aib	Ser	Lxx	Aib	Pro	Vxx	Aib	Vxx	Gln	Gln	Lxxol
**Trikoningin KA-like XXVII**	1862.12	1092.64	769.48	37.94	0.11%	0.35%	Ac	Aib	Gly	Ala	Aib	Vxx	Gln	Aib	Aib	Aib	*Ala*	Lxx	Aib	Pro	Vxx	Aib	Lxx	*Glu*	Gln	Lxxol
**Trikoningin KA-like XXVIII**	1878.12	1108.64	769.48	37.94	0.28%	0.86%	Ac	Aib	Gly	*Gly*	Aib	Lxx	Gln	Aib	Aib	Aib	Ser	Lxx	Aib	Pro	Vxx	Aib	Lxx	*Glu*	Gln	Lxxol
**Trikoningin KA-like XXIX**	1848.10	1092.64	755.46	38.08	0.05%	0.17%	Ac	Aib	Gly	Ala	Aib	Vxx	Gln	Aib	Aib	Aib	*Ala*	Lxx	Aib	Pro	Vxx	Aib	Vxx	*Glu*	Gln	Lxxol
**Trikoningin KA-like XXX**	1864.10	1108.64	755.46	38.08	0.10%	0.30%	Ac	Aib	Gly	Ala	*Ala*	Lxx	Gln	Aib	Aib	Aib	Ser	Lxx	Aib	Pro	Vxx	Aib	Vxx	*Glu*	Gln	Lxxol
**Trikoningin KA-like XXXI**	1878.11	1122.65	755.46	38.08	0.42%	1.31%	Ac	Aib	Gly	Ala	Aib	Lxx	Gln	Aib	Aib	Aib	Ser	Lxx	Aib	Pro	Vxx	Aib	Vxx	*Glu*	Gln	Lxxol
**Trikoningin KA-like XXXII**	1847.12	1092.64	754.48	38.94	0.01%	0.03%	Ac	Ab	Gly	Ala	Aib	Vxx	Gln	Aib	Aib	Aib	*Ala*	Lxx	Aib	Pro	Vxx	Aib	Vxx	Gln	Gln	Lxxol
**Trikoningin KA-like XXXIII**	1861.14	1106.66	754.48	38.94	0.01%	0.04%	Ac	Aib	Gly	Ala	Aib	Lxx	Gln	Aib	Aib	Aib	*Ala*	Lxx	Aib	Pro	Vxx	Aib	Vxx	Gln	Gln	Lxxol
**Trikoningin KA-like XXXIV**	1877.13	1122.65	754.48	38.94	0.09%	0.28%	Ac	Aib	Gly	Ala	Aib	Lxx	Gln	Aib	Aib	Aib	Ser	Lxx	Aib	Pro	Vxx	Aib	Vxx	Gln	Gln	Lxxol
**Trikoningin KA-like XXXV**	1891.14	1122.65	768.49	39.41	0.97%	3.03%	Ac	Aib	Gly	Ala	Aib	Lxx	Gln	Aib	Aib	Aib	Ser	Lxx	Aib	Pro	Vxx	Aib	Lxx	Gln	Gln	Lxxol
**Trikoningin KA-like XXXVI**	1848.10	1092.64	755.46	39.46	0.00%	0.01%	Ac	Aib	Gly	Ala	Aib	Lxx	Gln	Aib	Aib	*Ala*	*Ala*	Lxx	Aib	Pro	Vxx	Aib	*Vxx*	*Glu*	Gln	Lxxol
**Trikoningin KA-like XXXVII**	1862.12	1106.66	755.46	39.46	0.01%	0.03%	Ac	Aib	Gly	Ala	Aib	Lxx	Gln	Aib	Aib	Aib	*Ala*	Lxx	Aib	Pro	Vxx	Aib	Vxx	*Glu*	Gln	Lxxol
**Trikoningin KA-like XXXVIII**	1878.11	1122.65	755.46	39.46	0.08%	0.23%	Ac	Aib	Gly	Ala	Aib	Lxx	Gln	Aib	Aib	Aib	Ser	Lxx	Aib	Pro	Vxx	Aib	Vxx	*Glu*	Gln	Lxxol
**Trikoningin KA-like XXXIX**	1892.13	1122.65	769.48	39.85	0.55%	1.71%	Ac	Aib	Gly	Ala	Aib	Lxx	Gln	Aib	Aib	Aib	Ser	Lxx	Aib	Pro	Vxx	Aib	Lxx	*Glu*	Gln	Lxxol
**Trikoningin KA-like XL**	1891.14	1122.65	768.49	40.59	1.49%	4.65%	Ac	Aib	Gly	Ala	Aib	Lxx	Gln	Aib	Aib	Aib	Ser	Lxx	Aib	Pro	Vxx	Aib	Lxx	Gln	Gln	Lxxol
**Trikoningin KA-like XLI**	1892.13	1122.65	769.48	40.9	0.64%	1.99%	Ac	Aib	Gly	Ala	Aib	Lxx	Gln	Aib	Aib	Aib	Ser	Lxx	Aib	Pro	Vxx	Aib	Lxx	*Glu*	Gln	Lxxol
**Trikoningin KA-like XLII**	1875.13	1106.64	768.49	41.88	0.32%	0.99%	Ac	Aib	Gly	Ala	Aib	Lxx	Gln	Aib	Aib	Aib	Ala	Lxx	Aib	Pro	Vxx	Aib	Lxx	Gln	Gln	Lxxol
**Trikoningin KA-like XLIII**	1891.14	1122.65	768.49	41.88	8.28%	25.83%	Ac	Aib	Gly	Ala	Aib	Lxx	Gln	Aib	Aib	Aib	Ser	Lxx	Aib	Pro	Vxx	Aib	Lxx	Gln	Gln	Lxxol
**Trikoningin KA-like XLIV**	1876.12	1106.64	769.48	42.36	0.19%	0.59%	Ac	Aib	Gly	Ala	Aib	Lxx	Gln	Aib	Aib	Aib	*Ala*	Lxx	Aib	Pro	Vxx	Aib	Lxx	*Glu*	Gln	Lxxol
**Trikoningin KA-like XLV**	1892.13	1122.65	769.48	42.36	9.14%	28.48%	Ac	Aib	Gly	Ala	Aib	Lxx	Gln	Aib	Aib	Aib	Ser	Lxx	Aib	Pro	Vxx	Aib	Lxx	*Glu*	Gln	Lxxol
**Trikoningin KA-like XLVI**	1875.15	1106.66	768.49	43.33	2.01%	6.25%	Ac	Aib	Gly	Ala	Aib	Lxx	Gln	Aib	Aib	Aib	Ala	Lxx	Aib	Pro	Vxx	Aib	Lxx	Gln	Gln	Lxxol
**Trikoningin KA-like XLVII**	1905.16	1136.67	768.49	43.33	0.07%	0.20%	Ac	Aib	Ala	Ala	Aib	Lxx	Gln	Aib	Aib	Aib	Ser	Lxx	Aib	Pro	Vxx	Aib	Lxx	Gln	Gln	Lxxol
**Trikoningin KA-like XLVIII**	1876.12	1106.64	769.48	43.83	2.16%	6.74%	Ac	Aib	Gly	Ala	Aib	Lxx	Gln	Aib	Aib	Aib	*Ala*	Lxx	Aib	Pro	Vxx	Aib	Lxx	*Glu*	Gln	Lxxol
**Trikoningin KA-like XLIX**	1906.15	1136.67	769.48	43.83	0.07%	0.21%	Ac	Aib	*Ala*	Ala	Aib	Lxx	Gln	Aib	Aib	Aib	Ser	Lxx	Aib	Pro	Vxx	Aib	Lxx	*Glu*	Gln	Lxxol
**Trikoningin KA-like L**	1891.14	1122.65	768.49	46.05	0.07%	0.23%	Ac	Aib	Gly	Ala	Aib	Lxx	Gln	Aib	Aib	Aib	Ser	Lxx	Aib	Pro	Vxx	Aib	Lxx	Gln	Gln	Lxxol
**Trikoningin KA-like LI**	1905.16	1136.67	768.49	46.05	0.71%	2.22%	Ac	Aib	Ala	Ala	Aib	Lxx	Gln	Aib	Aib	Aib	Ser	Lxx	Aib	Pro	Vxx	Aib	Lxx	Gln	Gln	Lxxol
**Trikoningin KA-like LII**	1892.13	1122.65	769.48	46.43	0.07%	0.20%	Ac	Aib	Gly	Ala	Aib	Lxx	Gln	Aib	Aib	Aib	Ser	Lxx	Aib	Pro	Vxx	Aib	Lxx	*Glu*	Gln	Lxxol
**Trikoningin KA-like LIII**	1906.15	1136.67	769.48	46.43	0.74%	2.31%	Ac	Aib	*Ala*	Ala	Aib	Lxx	Gln	Aib	Aib	Aib	Ser	Lxx	Aib	Pro	Vxx	Aib	Lxx	*Glu*	Gln	Lxxol
**Trikoningin KA-like LIV**	1889.16	1120.67	768.49	47.39	0.10%	0.32%	Ac	Aib	*Ala*	Ala	Aib	Lxx	Gln	Aib	Aib	Aib	*Ala*	Lxx	Aib	Pro	Vxx	Aib	Lxx	Gln	Gln	Lxxol
**Trikoningin KA-like LV**	1905.16	1136.67	768.49	47.39	0.01%	0.04%	Ac	Aib	Gly	Ala	Aib	Lxx	Gln	*Vxx*	Aib	Aib	Ser	Lxx	Aib	Pro	Vxx	Aib	Lxx	Gln	Gln	Lxxol
**Trikoningin KA-like LVI**	1890.15	1120.67	769.48	47.77	0.12%	0.38%	Ac	Aib	Ala	Ala	Aib	Lxx	Gln	Aib	Aib	Aib	*Ala*	Lxx	Aib	Pro	Vxx	Aib	Lxx	*Glu*	Gln	Lxxol
**Trikoningin KA-like LVII**	1906.15	1136.67	769.48	47.77	0.04%	0.13%	Ac	Aib	Gly	Ala	Aib	Lxx	Gln	Aib	Aib	*Vxx*	Ser	Lxx	Aib	Pro	Vxx	Aib	Lxx	*Glu*	Gln	Lxxol
*T.* cf*. dorothopsis* SZMC 28390																										
**Dorothopsin A-a I**	1821.05	1150.68	670.37	12.56	0.31%	0.31%	Ac	Aib	Ala	Aib	Aib	Lxx	Gln	Aib	Aib	Aib	Ser	Lxx	Aib	Pro	Vxx	Aib	*Lxx*	*Glu*	*Ala*	*Alao*l
**Dorothopsin A-a II**	1821.05	1150.68	670.37	13.59	1.33%	1.33%	Ac	Aib	Ala	Aib	Aib	Lxx	Gln	Aib	Aib	Aib	Ser	Lxx	Aib	Pro	Vxx	Aib	*Lxx*	*Glu*	*Ala*	*Alaol*
**Dorothopsin A-a III**	1891.14	1150.68	740.46	32.28	0.37%	0.37%	Ac	Aib	Ala	Aib	Aib	Lxx	Gln	Aib	Aib	Aib	Ser	Lxx	Aib	Pro	Vxx	Aib	Vxx	Gln	Gln	*Vxxol*
**Dorothopsin A-a IV**	1891.14	1150.68	740.46	33.3	1.64%	1.64%	Ac	Aib	Ala	Aib	Aib	Lxx	Gln	Aib	Aib	Aib	Ser	Lxx	Aib	Pro	Vxx	Aib	Vxx	Gln	Gln	*Vxxol*
**Dorothopsin A-a V**	1892.13	1150.68	741.45	33.76	0.72%	0.72%	Ac	Aib	Ala	Aib	Aib	Lxx	Gln	Aib	Aib	Aib	Ser	Lxx	Aib	Pro	Vxx	Aib	Vxx	*Glu*	Gln	*Vxxol*
**Dorothopsin A-a VI**	1905.16	1150.68	754.48	36.28	6.66%	6.66%	Ac	Aib	Ala	Aib	Aib	Lxx	Gln	Aib	Aib	Aib	Ser	Lxx	Aib	Pro	Vxx	Aib	*Lxx*	Gln	Gln	*Vxxol*
**Dorothopsin A-a VII**	1905.16	1150.68	754.48	37.53	54.05%	54.05%	Ac	Aib	Ala	Aib	Aib	Lxx	Gln	Aib	Aib	Aib	Ser	Lxx	Aib	Pro	Vxx	Aib	*Lxx*	Gln	Gln	*Vxxol*
**Dorothopsin A-a VIII**	1906.14	1150.68	755.46	37.98	10.48%	10.48%	Ac	Aib	Ala	Aib	Aib	Lxx	Gln	Aib	Aib	Aib	Ser	Lxx	Aib	Pro	Vxx	Aib	Lxx	*Glu*	Gln	*Vxxol*
**Dorothopsin A-a IX**	1906.22	1164.7	741.52	39.99	0.09%	0.09%	Ac	*Vxx*	Ala	Aib	Aib	Lxx	Gln	Aib	Aib	Aib	Ser	Lxx	Aib	Pro	Vxx	Aib	Vxx	*Glu*	Gln	*Vxxol*
**Dorothopsin A-a X**	1919.18	1164.7	754.48	40.0	4.26%	4.26%	Ac	*Vxx*	Ala	Aib	Aib	Lxx	Gln	Aib	Aib	Aib	Ser	Lxx	Aib	Pro	Vxx	Aib	*Lxx*	Gln	Gln	*Vxxol*
**Dorothopsin A-a XI**	1920.16	1164.7	755.46	40.4	0.10%	0.10%	Ac	*Vxx*	Ala	Aib	Aib	Lxx	Gln	Aib	Aib	Aib	Ser	Lxx	Aib	Pro	Vxx	Aib	*Lxx*	*Glu*	Gln	*Vxxol*
**Dorothopsin A-a XII**	1919.17	1150.68	768.49	41.9	2.63%	2.63%	Ac	Aib	Ala	Aib	Aib	Lxx	Gln	Aib	Aib	Aib	Ser	Lxx	Aib	Pro	Vxx	Aib	*Lxx*	Gln	Gln	Lxxol
**Dorothopsin A-a XIII**	1933.19	1164.7	768.49	42.26	0.27%	0.27%	Ac	*Vxx*	Ala	Aib	Aib	Lxx	Gln	Aib	Aib	Aib	Ser	Lxx	Aib	Pro	Vxx	Aib	*Lxx*	Gln	Gln	Lxxol
**Dorothopsin A-b I**	1736.00	1065.63	670.37	7.84	0.08%	0.08%	Ac	Aib	Ala	Aib	Aib	Lxx	Gln	Aib	Aib	-	Ser	Lxx	Aib	Pro	Vxx	Aib	*Lxx*	*Glu*	*Ala*	*Alaol*
**Dorothopsin A-b II**	1736.00	1065.63	670.37	8.98	0.42%	0.42%	Ac	Aib	Ala	Aib	Aib	Lxx	Gln	Aib	Aib	-	Ser	Lxx	Aib	Pro	Vxx	Aib	*Lxx*	*Glu*	*Ala*	*Alaol*
**Dorothopsin A-b III**	1806.09	1065.63	740.46	24.46	0.09%	0.09%	Ac	Aib	Ala	Aib	Aib	Lxx	Gln	Aib	Aib	-	Ser	Lxx	Aib	Pro	Vxx	Aib	Vxx	Gln	Gln	*Vxxol*
**Dorothopsin A-b IV**	1807.08	1065.63	741.45	25.72	0.07%	0.07%	Ac	Aib	Ala	Aib	Aib	Lxx	Gln	Aib	Aib	-	Ser	Lxx	Aib	Pro	Vxx	Aib	Vxx	*Glu*	Gln	*Vxxol*
**Dorothopsin A-b V**	1806.09	1065.63	740.46	29.68	0.36%	0.36%	Ac	Aib	Ala	Aib	Aib	Lxx	Gln	Aib	Aib	-	Ser	Lxx	Aib	Pro	Vxx	Aib	Vxx	Gln	Gln	*Vxxol*
**Dorothopsin A-b VI**	1807.08	1065.63	741.45	30.61	0.03%	0.03%	Ac	Aib	Ala	Aib	Aib	Lxx	Gln	Aib	Aib	-	Ser	Lxx	Aib	Pro	Vxx	Aib	Vxx	*Glu*	Gln	*Vxxol*
**Dorothopsin A-b VII**	1820.11	1065.63	754.48	29.29	2.51%	2.51%	Ac	Aib	Ala	Aib	Aib	Lxx	Gln	Aib	Aib	-	Ser	Lxx	Aib	Pro	Vxx	Aib	*Lxx*	Gln	Gln	*Vxxol*
**Dorothopsin A-b VIII**	1821.09	1065.63	755.46	30.44	1.29%	1.29%	Ac	Aib	Ala	Aib	Aib	Lxx	Gln	Aib	Aib	-	Ser	Lxx	Aib	Pro	Vxx	Aib	*Lxx*	*Glu*	Gln	*Vxxol*
**Doothopsin A-c I**	1606.96	1065.63	541.33	9.5	1.28%	1.28%	Ac	Aib	Ala	Aib	Aib	Lxx	Gln	Aib	Aib	-	Ser	Lxx	Aib	Pro	Vxx	Aib	*Lxx*	-	*Ala*	*Alaol*
**Dorothopsin A-d I**	1692.01	1150.68	541.33	16.09	8.35%	8.35%	Ac	Aib	Ala	Aib	Aib	Lxx	Gln	Aib	Aib	Aib	Ser	Lxx	Aib	Pro	Vxx	Aib	*Lxx*	-	*Ala*	*Alaol*
**Dorothopsin A-d II**	1692.01	1150.68	541.33	18.36	0.47%	0.47%	Ac	Aib	Ala	Aib	Aib	Lxx	Gln	Aib	Aib	Aib	Ser	Lxx	Aib	Pro	Vxx	Aib	*Lxx*	-	*Ala*	*Alaol*
**Dorothopsin A-d III**	1706.03	1164.7	541.33	18.36	0.28%	0.28%	Ac	*Vxx*	Ala	Aib	Aib	Lxx	Gln	Aib	Aib	Aib	Ser	Lxx	Aib	Pro	Vxx	Aib	*Lxx*	-	*Ala*	*Alaol*
**Dorothopsin A-e I**	1478.91	1065.63	413.28	10.69	0.12%	0.12%	Ac	Aib	Ala	Aib	Aib	Lxx	Gln	Aib	Aib	-	Ser	Lxx	Aib	Pro	Vxx	Aib	-	-	*Gly*	*Alaol*
**Dorothopsin A-f I**	1563.96	1150.68	413.28	18.29	0.29%	0.29%	Ac	Aib	Ala	Aib	Aib	Lxx	Gln	Aib	Aib	Aib	Ser	Lxx	Aib	Pro	Vxx	Aib	-	-	*Gly*	*Alaol*
**Dorothopsin A-f II**	1563.96	1150.68	413.28	19.49	1.46%	1.46%	Ac	Aib	Ala	Aib	Aib	Lxx	Gln	Aib	Aib	Aib	Ser	Lxx	Aib	Pro	Vxx	Aib	-	-	*Gly*	*Alaol*
*T. atroviride* SZMC 28748																										
**Trichorzianin TA-like I**	1964.14	1108.64	855.5	20.26	0.09%	0.19%	Ac	Aib	Ala	Ala	Aib	Aib	Gln	Aib	Aib	Aib	Ser	Lxx	Aib	Pro	Lxx	Aib	Lxx	Gln	Gln	Trpol
**Trichorzianin TA-like II**	1950.13	1108.64	841.49	21.39	0.37%	0.75%	Ac	Aib	Ala	Ala	Aib	Aib	Gln	Aib	Aib	Aib	Ser	Lxx	Aib	Pro	Vxx	Aib	Lxx	Gln	Gln	Trpol
**Trichorzianin TA-like III**	1964.14	1108.64	855.5	23.02	0.23%	0.48%	Ac	Aib	*Gly*	Ala	Aib	Vxx	Gln	Aib	Aib	Aib	Ser	Lxx	Aib	Pro	Lxx	Aib	Lxx	Gln	Gln	Trpol
**Trichorzianin TA-like IV**	1950.13	1108.64	841.49	24.2	0.88%	1.81%	Ac	Aib	*Gly*	Ala	Aib	Vxx	Gln	Aib	Aib	Aib	Ser	Lxx	Aib	Pro	Vxx	Aib	Lxx	Gln	Gln	Trpol
**Trichorzianin TA-like V**	1964.14	1108.64	855.5	25.43	0.92%	1.89%	Ac	Aib	Ala	Ala	Aib	Aib	Gln	Aib	Aib	Aib	Ser	Lxx	Aib	Pro	Lxx	Aib	Lxx	Gln	Gln	Trpol
**Trichorzianin TA-like VI**	1964.14	1108.64	855.5	25.9	0.21%	0.42%	Ac	Aib	Ala	Ala	Aib	Aib	Gln	Aib	Aib	Aib	Ser	Lxx	Aib	Pro	Lxx	Aib	Lxx	Gln	Gln	Trpol
**Trichorzianin TA-like VII**	1950.13	1108.64	841.49	26.72	3.74%	7.68%	Ac	Aib	Ala	Ala	Aib	Aib	Gln	Aib	Aib	Aib	Ser	Lxx	Aib	Pro	Vxx	Aib	Lxx	Gln	Gln	Trpol
**Trichorzianin TA-like VIII**	1941.12	1124.63	816.49	27.55	0.24%	0.49%	Ac	Aib	Ala	*Ser*	Aib	Aib	Gln	Aib	Aib	Aib	Ser	Lxx	Aib	Pro	Lxx	Aib	Lxx	Gln	Gln	Pheol
**Trichorzianin TA-like IX**	1950.13	1108.64	841.49	28.13	0.47%	0.96%	Ac	Aib	Ala	Ala	Aib	Vxx	Gln	Ala	Aib	Aib	Ser	Lxx	Aib	Pro	Vxx	Aib	Lxx	Gln	Gln	Trpol
**Trichorzianin TA-like X**	1978.15	1122.65	855.5	28.62	1.35%	2.77%	Ac	Aib	Ala	Ala	Aib	Vxx	Gln	Aib	Aib	Aib	Ser	Lxx	Aib	Pro	Lxx	Aib	Lxx	Gln	Gln	Trpol
**Trichorzianin TA-like XI**	1964.14	1122.65	841.49	28.84	0.26%	0.54%	Ac	Aib	Ala	Ala	Aib	Vxx	Gln	Aib	Aib	Aib	Ser	Lxx	Aib	Pro	Vxx	Aib	Lxx	Gln	Gln	Trpol
**Trichorzianin TA-like XII**	1911.11	1094.62	816.49	28.98	0.10%	0.21%	Ac	Aib	Ala	Ala	Aib	Aib	Gln	Aib	*Ala*	Aib	Ser	Lxx	Aib	Pro	Lxx	Aib	Lxx	Gln	Gln	Pheol
**Trichorzianin TA-like XIII**	1978.15	1122.65	855.5	29.09	0.24%	0.49%	Ac	Aib	Ala	Ala	Aib	Vxx	Gln	Aib	Aib	Aib	Ser	Lxx	Aib	Pro	Lxx	Aib	Lxx	Gln	Gln	Trpol
**Trichorzianin TA-like XIV**	1927.11	1124.63	802.48	29.25	0.99%	2.03%	Ac	Aib	Ala	*Ser*	Aib	Aib	Gln	Aib	Aib	Aib	Ser	Lxx	Aib	Pro	Vxx	Aib	Lxx	Gln	Gln	Pheol
**Trichorzianin TA-like XV**	1964.14	1122.65	841.49	29.94	4.37%	8.99%	Ac	Aib	Ala	Ala	Aib	Vxx	Gln	Aib	Aib	Aib	Ser	Lxx	Aib	Pro	Vxx	Aib	Lxx	Gln	Gln	Trpol
**Trichorzianin TA-like XVI**	1955.14	1138.65	816.49	30.54	0.65%	1.33%	Ac	Aib	*Ser*	Ala	Aib	Vxx	Gln	Aib	Aib	Aib	Ser	Lxx	Aib	Pro	Lxx	Aib	Lxx	Gln	Gln	Pheol
**Trichorzianin TA-like XVII**	1962.16	1106.66	855.5	31.09	0.09%	0.18%	Ac	Aib	Ala	Ala	Aib	Vxx	Gln	Aib	Aib	Aib	*Ala*	Lxx	Aib	Pro	Lxx	Aib	Lxx	Gln	Gln	Trpol
**Trichorzianin TA-like XVIII**	1897.10	1094.62	802.48	31.33	0.82%	1.68%	Ac	Aib	Ala	Ala	Aib	Aib	Gln	Aib	*Ala*	Aib	Ser	Lxx	Aib	Pro	Vxx	Aib	Lxx	Gln	Gln	Pheol
**Trichorzianin TA-like XIX**	1948.15	1106.66	841.49	31.97	0.41%	0.83%	Ac	Aib	Ala	Ala	Aib	Vxx	Gln	Aib	Aib	Aib	*Ala*	Lxx	Aib	Pro	Vxx	Aib	Lxx	Gln	Gln	Trpol
**Trichorzianin TA-like XX**	1941.13	1138.65	802.48	32.35	2.56%	5.26%	Ac	Aib	*Ser*	Ala	Aib	Vxx	Gln	Aib	Aib	Aib	Ser	Lxx	Aib	Pro	Vxx	Aib	Lxx	Gln	Gln	Pheol
**Trichorzianin TA-like XXI**	1925.13	1108.64	816.49	32.78	0.36%	0.75%	Ac	Aib	Ala	Ala	Aib	Aib	Gln	Aib	Aib	Aib	Ser	Lxx	Aib	Pro	Lxx	Aib	Lxx	Gln	Gln	Pheol
**Trichorzianin TA-like XXII**	1925.13	1108.64	816.49	33.16	2.65%	5.44%	Ac	Aib	Ala	Ala	Aib	Aib	Gln	Aib	Aib	Aib	Ser	Lxx	Aib	Pro	Lxx	Aib	Lxx	Gln	Gln	Pheol
**Trichorzianin TA-like XXIII**	1911.12	1108.64	802.48	33.81	1.88%	3.86%	Ac	Aib	Ala	Ala	Aib	Aib	Gln	Aib	Aib	Aib	Ser	Lxx	Aib	Pro	Vxx	Aib	Lxx	Gln	Gln	Pheol
**Trichorzianin TA-like XXIV**	1925.13	1108.64	816.49	34.42	0.35%	0.72%	Ac	Aib	Ala	Ala	Aib	Vxx	Gln	*Ala*	Aib	Aib	Ser	Lxx	Aib	Pro	Lxx	Aib	Lxx	Gln	Gln	Pheol
**Trichorzianin TA-like XXV**	1911.12	1108.64	802.48	35.02	7.42%	15.24%	Ac	Aib	Ala	Ala	Aib	Aib	Gln	Aib	Aib	Aib	Ser	Lxx	Aib	Pro	Vxx	Aib	Lxx	Gln	Gln	Pheol
**Trichorzianin TA-like XXVI**	1939.14	1122.65	816.49	35.97	0.57%	1.17%	Ac	Aib	Ala	Ala	Aib	Vxx	Gln	Aib	Aib	Aib	Ser	Lxx	Aib	Pro	Lxx	Aib	Lxx	Gln	Gln	Pheol
**Trichorzianin TA-like XXVII**	1939.14	1122.65	816.49	36.45	3.01%	6.19%	Ac	Aib	Ala	Ala	Aib	Vxx	Gln	Aib	Aib	Aib	Ser	Lxx	Aib	Pro	Lxx	Aib	Lxx	Gln	Gln	Pheol
**Trichorzianin TA-like XXVIII**	1925.13	1122.65	802.48	37.03	2.60%	5.34%	Ac	Aib	Ala	Ala	Aib	Vxx	Gln	Aib	Aib	Aib	Ser	Lxx	Aib	Pro	Vxx	Aib	Lxx	Gln	Gln	Pheol
**Trichorzianin TA-like XXIX**	1925.13	1122.65	802.48	38.2	8.56%	17.59%	Ac	Aib	Ala	Ala	Aib	Vxx	Gln	Aib	Aib	Aib	Ser	Lxx	Aib	Pro	Vxx	Aib	Lxx	Gln	Gln	Pheol
**Trichorzianin TA-like XXX**	1923.15	1106.66	816.49	38.7	0.41%	0.85%	Ac	Aib	Ala	Ala	Aib	Vxx	Gln	Aib	Aib	Aib	*Ala*	Lxx	Aib	Pro	Lxx	Aib	Lxx	Gln	Gln	Pheol
**Trichorzianin TA-like XXXI**	1909.14	1106.66	802.48	40.1	0.90%	1.84%	Ac	Aib	Ala	Ala	Aib	Vxx	Gln	Aib	Aib	Aib	*Ala*	Lxx	Aib	Pro	Vxx	Aib	Lxx	Gln	Gln	Pheol
**Trichorzianin TA-like XXXII**	1939.14	1122.65	816.49	40.36	0.19%	0.39%	Ac	Aib	Ala	Ala	Aib	Vxx	Gln	Aib	Aib	Aib	Ser	Lxx	Aib	Pro	Lxx	Aib	Lxx	Gln	Gln	Pheol
**Trichorzianin TA-like XXXIII**	1925.13	1122.65	802.48	42.16	0.82%	1.68%	Ac	Aib	Ala	Ala	Aib	Vxx	Gln	Aib	Aib	Aib	Ser	Lxx	Aib	Pro	Vxx	Aib	Lxx	Gln	Gln	Pheol
**Peptaibol-like I**	1792.04	1108.64	683.4	4.57	0.04%	1.65%	Ac	Aib	Ala	Ala	Aib	Aib	Gln	Aib	Aib	Aib	Ser	Lxx	Aib	Pro	Lxx	Aib	Lxx	Gln	146	
**Peptaibol-like II**	1791.05	1122.65	668.4	5.69	0.12%	4.47%	Ac	Aib	Ala	Ala	Aib	Vxx	Gln	Aib	Aib	Aib	Ser	Lxx	Aib	Pro	Vxx	Aib	Lxx	Gln	145	
**Peptaibol-like III**	1792.04	1108.64	683.4	6.81	0.01%	0.50%	Ac	Aib	Ala	Ala	Aib	Aib	Gln	Aib	Aib	Aib	Ser	Lxx	Aib	Pro	Lxx	Aib	Lxx	Gln	146	
**Peptaibol-like IV**	1778.03	1108.64	669.39	6.9	0.04%	1.52%	Ac	Aib	Ala	Ala	Aib	Aib	Gln	Aib	Aib	Aib	Ser	Lxx	Aib	Pro	Vxx	Aib	Lxx	Gln	146	
**Peptaibol-like V**	1777.04	1108.64	668.4	7.4	0.04%	1.64%	Ac	Aib	Ala	Ala	Aib	Aib	Gln	Aib	Aib	Aib	Ser	Lxx	Aib	Pro	Vxx	Aib	Lxx	Gln	145	
**Peptaibol-like VI**	1792.04	1108.64	683.4	7.75	0.05%	1.86%	Ac	Aib	Ala	Ala	Aib	Aib	Gln	Aib	Aib	Aib	Ser	Lxx	Aib	Pro	Lxx	Aib	Lxx	Gln	146	
**Peptaibol-like VII**	1778.03	1108.64	669.39	8.09	0.13%	4.72%	Ac	Aib	Ala	Ala	Aib	Aib	Gln	Aib	Aib	Aib	Ser	Lxx	Aib	Pro	Vxx	Aib	Lxx	Gln	146	
**Peptaibol-like VIII**	1805.06	1122.65	682.41	8.28	0.00%	0.13%	Ac	Aib	Ala	Ala	Aib	Vxx	Gln	Aib	Aib	Aib	Ser	Lxx	Aib	Pro	Lxx	Aib	Lxx	Gln	145	
**Peptaibol-like IX**	1792.04	1122.65	669.39	9.12	0.02%	0.90%	Ac	Aib	Ala	Ala	Aib	Vxx	Gln	Aib	Aib	Aib	Ser	Lxx	Aib	Pro	Vxx	Aib	Lxx	Gln	146	
**Peptaibol-like X**	1806.05	1122.65	683.4	9.59	0.07%	2.48%	Ac	Aib	Ala	Ala	Aib	Vxx	Gln	Aib	Aib	Aib	Ser	Lxx	Aib	Pro	Lxx	Aib	Lxx	Gln	146	
**Peptaibol-like XI**	1792.04	1122.65	669.39	9.97	0.16%	6.04%	Ac	Aib	Ala	Ala	Aib	Vxx	Gln	Aib	Aib	Aib	Ser	Lxx	Aib	Pro	Vxx	Aib	Lxx	Gln	146	
**Peptaibol-like XII**	1791.05	1122.65	668.4	10.27	0.02%	0.86%	Ac	Aib	Ala	Ala	Aib	Vxx	Gln	Aib	Aib	Aib	Ser	Lxx	Aib	Pro	Vxx	Aib	Lxx	Gln	145	
**Peptaibol-like XIII**	1791.05	1108.64	682.41	10.64	0.01%	0.28%	Ac	Aib	Ala	Ala	Aib	Aib	Gln	Aib	Aib	Aib	Ser	Lxx	Aib	Pro	Lxx	Aib	Lxx	Gln	145	
**Peptaibol-like XIV**	1777.04	1108.64	668.4	11.97	0.10%	3.57%	Ac	Aib	Ala	Ala	Aib	Aib	Gln	Aib	Aib	Aib	Ser	Lxx	Aib	Pro	Vxx	Aib	Lxx	Gln	145	
**Peptaibol-like XV**	1791.05	1108.64	682.41	12.19	0.02%	0.80%	Ac	Aib	Ala	Ala	Aib	Aib	Gln	Aib	Aib	Aib	Ser	Lxx	Aib	Pro	Lxx	Aib	Lxx	Gln	145	
**Peptaibol-like XVI**	1791.05	1122.65	668.4	13.37	0.02%	0.57%	Ac	Aib	Ala	Ala	Aib	Vxx	Gln	Aib	Aib	Aib	Ser	Lxx	Aib	Pro	Vxx	Aib	Lxx	Gln	145	
**Peptaibol-like XVII**	1791.05	1108.64	682.41	13.82	0.00%	0.14%	Ac	Aib	Ala	Ala	Aib	Aib	Gln	Aib	Aib	Aib	Ser	Lxx	Aib	Pro	Lxx	Aib	Lxx	Gln	145	
**Peptaibol-like XVIII**	1791.05	1122.65	668.4	14.45	0.13%	4.70%	Ac	Aib	Ala	Ala	Aib	Vxx	Gln	Aib	Aib	Aib	Ser	Lxx	Aib	Pro	Vxx	Aib	Lxx	Gln	145	
**Peptaibol-like XIX**	1805.06	1122.65	682.41	14.73	0.04%	1.31%	Ac	Aib	Ala	Ala	Aib	Vxx	Gln	Aib	Aib	Aib	Ser	Lxx	Aib	Pro	Lxx	Aib	Lxx	Gln	145	
**Peptaibol-like XX**	1806.05	1122.65	683.4	14.73	0.02%	0.74%	Ac	Aib	Ala	Ala	Aib	Vxx	Gln	Aib	Aib	Aib	Ser	Lxx	Aib	Pro	Vxx	Aib	Lxx	Gln	160	
**Peptaibol-like XXI**	1791.05	1122.65	668.4	16.01	0.02%	0.65%	Ac	Aib	Ala	Ala	Aib	Vxx	Gln	Aib	Aib	Aib	Ser	Lxx	Aib	Pro	Vxx	Aib	Lxx	Gln	145	
**Peptaibol-like XXII**	1791.05	1108.64	682.41	16.51	0.01%	0.22%	Ac	Aib	Ala	Ala	Aib	Aib	Gln	Aib	Aib	Aib	Ser	Lxx	Aib	Pro	Lxx	Aib	Lxx	Gln	145	
**Peptaibol-like XXIII**	1792.04	1108.64	683.4	16.93	0.09%	3.50%	Ac	Aib	Ala	Ala	Aib	Aib	Gln	Aib	Aib	Aib	Ser	Lxx	Aib	Pro	Vxx	Aib	Lxx	Gln	160	
**Peptaibol-like XXIV**	1792.04	1108.64	683.4	18.36	0.02%	0.70%	Ac	Aib	Ala	Ala	Aib	Aib	Gln	Aib	Aib	Aib	Ser	Lxx	Aib	Pro	Vxx	Aib	Lxx	Gln	160	
**Peptaibol-like XXV**	1806.05	1122.65	683.4	19.95	0.12%	4.54%	Ac	Aib	Ala	Ala	Aib	Vxx	Gln	Aib	Aib	Aib	Ser	Lxx	Aib	Pro	Vxx	Aib	Lxx	Gln	160	
**Peptaibol-like XXVI**	1806.05	1122.65	683.4	21.55	0.02%	0.80%	Ac	Aib	Ala	Ala	Aib	Vxx	Gln	Aib	Aib	Aib	Ser	Lxx	Aib	Pro	Vxx	Aib	Lxx	Gln	160	
**Peptaibol-like XXVII**	1982.12	1108.64	873.48	9.81	0.17%	6.31%	Ac	Aib	Ala	Ala	Aib	Aib	Gln	Aib	Aib	Aib	Ser	Lxx	Aib	Pro	Vxx	Aib	Lxx	Gln	Gln	222
**Peptaibol-like XXVIII**	1996.14	1108.64	887.5	10.53	0.05%	1.76%	Ac	Aib	Ala	Ala	Aib	Aib	Gln	Aib	Aib	Aib	Ser	Lxx	Aib	Pro	Lxx	Aib	Lxx	Gln	Gln	222
**Peptaibol-like XXIX**	1982.12	1108.64	873.48	11.6	0.07%	2.53%	Ac	Aib	Ala	Ala	Aib	Aib	Gln	Aib	Aib	Aib	Ser	Lxx	Aib	Pro	Vxx	Aib	Lxx	Gln	Gln	222
**Peptaibol-like XXX**	1982.12	1108.64	873.48	11.9	0.22%	8.09%	Ac	Aib	Ala	Ala	Aib	Aib	Gln	Aib	Aib	Aib	Ser	Lxx	Aib	Pro	Vxx	Aib	Lxx	Gln	Gln	222
**Peptaibol-like XXXI**	1996.14	1108.64	887.5	12.00	0.03%	1.30%	Ac	Aib	Ala	Ala	Aib	Aib	Gln	Aib	Aib	Aib	Ser	Lxx	Aib	Pro	Lxx	Aib	Lxx	Gln	Gln	222
**Peptaibol-like XXXII**	2010.15	1122.65	887.5	12.78	0.06%	2.14%	Ac	Aib	Ala	Ala	Aib	Vxx	Gln	Aib	Aib	Aib	Ser	Lxx	Aib	Pro	Lxx	Aib	Lxx	Gln	Gln	222
**Peptaibol-like XXXIII**	1996.13	1122.65	873.48	14.04	0.15%	5.73%	Ac	Aib	Ala	Ala	Aib	Vxx	Gln	Aib	Aib	Aib	Ser	Lxx	Aib	Pro	Vxx	Aib	Lxx	Gln	Gln	222
**Peptaibol-like XXXIV**	1996.14	1108.64	887.5	14.45	0.06%	2.38%	Ac	Aib	Ala	Ala	Aib	Aib	Gln	Aib	Aib	Aib	Ser	Lxx	Aib	Pro	Lxx	Aib	Lxx	Gln	Gln	222
**Peptaibol-like XXXV**	1996.13	1122.65	873.48	15.49	0.04%	1.53%	Ac	Aib	Ala	Ala	Aib	Vxx	Gln	Aib	Aib	Aib	Ser	Lxx	Aib	Pro	Vxx	Aib	Lxx	Gln	Gln	222
**Peptaibol-like XXXVI**	1996.14	1108.64	887.5	16.57	0.05%	1.96%	Ac	Aib	Ala	Ala	Aib	Aib	Gln	Aib	Aib	Aib	Ser	Lxx	Aib	Pro	Lxx	Aib	Lxx	Gln	Gln	222
**Peptaibol-like XXXVII**	1996.14	1108.64	887.5	17.62	0.03%	1.05%	Ac	Aib	Ala	Ala	Aib	Aib	Gln	Aib	Aib	Aib	Ser	Lxx	Aib	Pro	Lxx	Aib	Lxx	Gln	Gln	222
**Peptaibol-like XXXVIII**	1982.12	1108.64	873.48	17.81	0.14%	5.35%	Ac	Aib	Ala	Ala	Aib	Aib	Gln	Aib	Aib	Aib	Ser	Lxx	Aib	Pro	Vxx	Aib	Lxx	Gln	Gln	222
**Peptaibol-like XXXIX**	2010.15	1122.65	887.5	19.43	0.06%	2.34%	Ac	Aib	Ala	Ala	Aib	Vxx	Gln	Aib	Aib	Aib	Ser	Lxx	Aib	Pro	Lxx	Aib	Lxx	Gln	Gln	222
**Peptaibol-like XL**	2010.15	1122.65	887.5	20.6	0.04%	1.55%	Ac	Aib	Ala	Ala	Aib	Vxx	Gln	Aib	Aib	Aib	Ser	Lxx	Aib	Pro	Lxx	Aib	Lxx	Gln	Gln	222
**Peptaibol-like XLI**	1996.13	1122.65	873.48	20.69	0.18%	6.68%	Ac	Aib	Ala	Ala	Aib	Vxx	Gln	Aib	Aib	Aib	Ser	Lxx	Aib	Pro	Vxx	Aib	Lxx	Gln	Gln	222
*T. hamatum* SZMC 28747																										
**Tricholongin LB-like I**	1925.14	1170.66	754.48	16.82	0.28%	1.17%	Ac	Aib	Gly	Phe	Aib	Aib	Gln	Aib	Aib	Aib	Ser	Lxx	Aib	Pro	Vxx	Aib	Vxx	Gln	Gln	Lxxol
**Tricholongin LB-like II**	1835.08	1094.62	740.46	19.69	0.39%	1.66%	Ac	Aib	Gly	*Ala*	Aib	Aib	Gln	Aib	Aib	Aib	Ser	Lxx	Aib	Pro	Vxx	Aib	Aib	Gln	Gln	Lxxol
**Tricholongin LB-like III**	1897.10	1156.64	740.46	20.39	0.21%	0.91%	Ac	*Ala*	Gly	Phe	Aib	Aib	Gln	Aib	Aib	Aib	Ser	Lxx	Aib	Pro	Vxx	Aib	Aib	Gln	Gln	Lxxol
**Tricholongin LB-like IV**	1849.10	1094.62	754.48	22.32	0.36%	1.50%	Ac	Aib	Gly	*Ala*	Aib	Aib	Gln	Aib	Aib	Aib	Ser	Lxx	Aib	Pro	Vxx	Aib	Vxx	Gln	Gln	Lxxol
**Tricholongin LB-like V**	1927.10	1186.64	740.46	22.83	0.31%	1.29%	Ac	Aib	Gly	*Tyr*	Aib	Aib	Gln	Aib	Aib	Aib	Ser	Lxx	Aib	Pro	Vxx	Aib	Aib	Gln	Gln	Lxxol
**Tricholongin LB-like VI**	1911.12	1156.64	754.48	22.94	0.31%	1.31%	Ac	*Ala*	Gly	Phe	Aib	Aib	Gln	Aib	Aib	Aib	Ser	Lxx	Aib	Pro	Vxx	Aib	Vxx	Gln	Gln	Lxxol
**Tricholongin LB-like VII**	1941.12	1186.64	754.48	25.48	0.35%	1.47%	Ac	Aib	Gly	*Tyr*	Aib	Aib	Gln	Aib	Aib	Aib	Ser	Lxx	Aib	Pro	Vxx	Aib	Vxx	Gln	Gln	Lxxol
**Tricholongin LB-like VIII**	1897.10	1156.64	740.46	26.14	0.59%	2.49%	Ac	Aib	Gly	Phe	Aib	Aib	Gln	*Ala*	Aib	Aib	Ser	Lxx	Aib	Pro	Vxx	Aib	Aib	Gln	Gln	Lxxol
**Tricholongin LB-like IX**	1897.10	1156.64	740.46	26.66	0.46%	1.93%	Ac	Aib	Gly	Phe	*Ala*	Aib	Gln	Aib	Aib	Aib	Ser	Lxx	Aib	Pro	Vxx	Aib	Aib	Gln	Gln	Lxxol
**Tricholongin LB-like X**	1911.12	1156.64	754.48	27.13	0.50%	2.10%	Ac	Aib	Gly	Phe	Aib	*Ala*	Gln	Aib	Aib	Aib	Ser	Lxx	Aib	Pro	Vxx	Aib	Vxx	Gln	Gln	Lxxol
**Tricholongin LB-like XI**	1911.11	1170.65	740.46	27.43	0.34%	1.43%	Ac	Aib	Gly	Phe	Aib	Aib	Gln	Aib	Aib	Aib	Ser	Lxx	Aib	Pro	Vxx	Aib	Aib	Gln	Gln	Lxxol
**Tricholongin LB-like XII**	1911.12	1156.64	754.48	28.14	0.45%	1.90%	Ac	Aib	Gly	Phe	Aib	Aib	Gln	Aib	*Ala*	Aib	Ser	Lxx	Aib	Pro	Vxx	Aib	Vxx	Gln	Gln	Lxxol
**Tricholongin LB-like XIII**	1911.11	1170.65	740.46	28.7	6.07%	25.68%	Ac	Aib	Gly	Phe	Aib	Aib	Gln	Aib	Aib	Aib	Ser	Lxx	Aib	Pro	Vxx	Aib	Aib	Gln	Gln	Lxxol
**Tricholongin LB-like XIV**	1911.12	1156.64	754.48	29.36	0.71%	3.01%	Ac	Aib	Gly	Phe	*Ala*	Aib	Gln	Aib	Aib	Aib	Ser	Lxx	Aib	Pro	Vxx	Aib	Vxx	Gln	Gln	Lxxol
**Tricholongin LB-like XV**	1911.11	1170.65	740.46	29.91	0.40%	1.70%	Ac	Aib	Gly	Phe	Aib	Aib	Gln	Aib	Aib	Aib	Ser	Lxx	Aib	Pro	Vxx	Aib	Aib	Gln	Gln	Lxxol
**Tricholongin LB-like XVI**	1925.13	1170.65	754.48	31.37	6.87%	29.09%	Ac	Aib	Gly	Phe	Aib	Aib	Gln	Aib	Aib	Aib	Ser	Lxx	Aib	Pro	Vxx	Aib	Vxx	Gln	Gln	Lxxol
**Tricholongin LB-like XVII**	1925.13	1184.67	740.46	32.02	1.68%	7.13%	Ac	Aib	*Ala*	Phe	Aib	Aib	Gln	Aib	Aib	Aib	Ser	Lxx	Aib	Pro	Vxx	Aib	Aib	Gln	Gln	Lxxol
**Tricholongin LB-like XVIII**	1925.13	1170.65	754.48	32.53	0.59%	2.51%	Ac	Aib	Gly	Phe	Aib	Aib	Gln	Aib	Aib	Aib	Ser	Lxx	Aib	Pro	Vxx	Aib	Vxx	Gln	Gln	Lxxol
**Tricholongin LB-like XIX**	1939.15	1184.67	754.48	34.64	2.37%	10.03%	Ac	Aib	*Ala*	Phe	Aib	Aib	Gln	Aib	Aib	Aib	Ser	Lxx	Aib	Pro	Vxx	Aib	Vxx	Gln	Gln	Lxxol
**Tricholongin LB-like XX**	1909.13	1168.67	740.46	34.74	0.09%	0.36%	Ac	Aib	*Ala*	Phe	Aib	Aib	Gln	Aib	Aib	Aib	*Ala*	Lxx	Aib	Pro	Vxx	Aib	*Aib*	Gln	Gln	Lxxol
**Tricholongin LB-like XXI**	1923.15	1168.67	754.48	37.27	0.31%	1.33%	Ac	Aib	*Ala*	Phe	Aib	Aib	Gln	Aib	Aib	Aib	Ala	Lxx	Aib	Pro	Vxx	Aib	Vxx	Gln	Gln	Lxxol
*T.* cf*. dorothopsis* SZMC 28005																										
**Dorothopsin B I**	1897.10	1170.65	726.45	20.25	0.46%	1.38%	Ac	*Phe*	Gly	*Aib*	Aib	*Vxx*	Gln	Aib	Aib	Aib	Ser	*Vxx*	Aib	Pro	Vxx	Aib	Aib	Gln	Gln	*Vxxol*
**Dorothopsin B II**	1898.08	1170.65	727.43	20.39	0.61%	1.85%	Ac	*Phe*	Gly	*Aib*	Aib	*Vxx*	Gln	Aib	Aib	Aib	Ser	*Vxx*	Aib	Pro	Vxx	Aib	Aib	*Glu*	Gln	*Vxxol*
**Dorothopsin B III**	1911.12	1184.67	726.45	21.53	0.31%	0.92%	Ac	*Phe*	Gly	*Aib*	Aib	*Lxx*	Gln	Aib	*Ala*	Aib	Ser	Lxx	Aib	Pro	Vxx	Aib	Aib	Gln	Gln	*Vxxol*
**Dorothopsin B IV**	1912.10	1184.67	727.43	21.68	0.49%	1.47%	Ac	*Phe*	Gly	*Aib*	Aib	*Lxx*	Gln	Aib	*Ala*	Aib	Ser	Lxx	Aib	Pro	Vxx	Aib	Aib	*Glu*	Gln	*Vxxol*
**Dorothopsin B V**	1911.12	1184.67	726.45	22.34	0.78%	2.35%	Ac	*Phe*	Gly	*Aib*	Aib	*Vxx*	Gln	Aib	Aib	Aib	Ser	Lxx	Aib	Pro	Vxx	Aib	Aib	Gln	Gln	*Vxxol*
**Dorothopsin B VI**	1912.10	1184.67	727.43	22.51	1.29%	3.89%	Ac	*Phe*	Gly	*Aib*	Aib	*Vxx*	Gln	Aib	Aib	Aib	Ser	Lxx	Aib	Pro	Vxx	Aib	Aib	*Glu*	Gln	*Vxxol*
**Dorothopsin B VII**	1964.14	1237.69	726.45	23.4	1.67%	5.03%	Ac	*Trp*	Gly	*Aib*	Aib	*Lxx*	Gln	Aib	Aib	Aib	Ser	Lxx	Aib	Pro	Vxx	Aib	Aib	Gln	Gln	*Vxxol*
**Dorothopsin B VIII**	1965.12	1237.69	727.43	23.65	1.01%	3.03%	Ac	*Trp*	Gly	*Aib*	Aib	*Lxx*	Gln	Aib	Aib	Aib	Ser	Lxx	Aib	Pro	Vxx	Aib	Aib	*Glu*	Gln	*Vxxol*
**Dorothopsin B IX**	1911.12	1184.67	726.45	23.81	0.34%	1.03%	Ac	*Phe*	Gly	*Aib*	Aib	*Lxx*	Gln	Aib	*Ala*	Aib	Ser	Lxx	Aib	Pro	Vxx	Aib	Aib	Gln	Gln	*Vxxol*
**Dorothopsin B X**	1912.10	1184.67	727.43	24.06	1.00%	2.99%	Ac	*Phe*	Gly	*Aib*	Aib	*Lxx*	Gln	Aib	*Ala*	Aib	Ser	Lxx	Aib	Pro	Vxx	Aib	Aib	*Glu*	Gln	*Vxxol*
**Dorothopsin B XI**	1911.12	1184.67	726.45	25.05	0.54%	1.62%	Ac	*Phe*	Gly	*Aib*	Aib	*Vxx*	Gln	Aib	Aib	Aib	Ser	Lxx	Aib	Pro	Vxx	Aib	Aib	Gln	Gln	*Vxxol*
**Dorothopsin B XII**	1964.14	1237.69	726.45	25.05	0.24%	0.72%	Ac	*Trp*	Gly	*Aib*	Aib	*Lxx*	Gln	Aib	Aib	Aib	Ser	Lxx	Aib	Pro	Vxx	Aib	Aib	Gln	Gln	*Vxxol*
**Dorothopsin B XIII**	1925.13	1198.68	726.45	25.99	3.23%	9.71%	Ac	*Phe*	Gly	*Aib*	Aib	*Lxx*	Gln	Aib	Aib	Aib	Ser	Lxx	Aib	Pro	Vxx	Aib	Aib	Gln	Gln	*Vxxol*
**Dorothopsin B XIV**	1964.14	1237.69	726.45	25.99	0.74%	2.22%	Ac	*Trp*	Gly	*Aib*	Aib	*Lxx*	Gln	Aib	Aib	Aib	Ser	Lxx	Aib	Pro	Vxx	Aib	Aib	Gln	Gln	*Vxxol*
**Dorothopsin B XV**	1926.11	1198.68	727.43	26.18	2.64%	7.95%	Ac	*Phe*	Gly	*Aib*	Aib	*Lxx*	Gln	Aib	Aib	Aib	Ser	Lxx	Aib	Pro	Vxx	Aib	Aib	*Glu*	Gln	*Vxxol*
**Dorothopsin B XVI**	1965.12	1237.69	727.43	26.18	0.60%	1.82%	Ac	*Trp*	Gly	*Aib*	Aib	*Lxx*	Gln	Aib	Aib	Aib	Ser	Lxx	Aib	Pro	Vxx	Aib	Aib	*Glu*	Gln	*Vxxol*
**Dorothopsin B XVII**	1911.12	1184.67	726.45	27.78	0.14%	0.41%	Ac	*Phe*	Gly	*Aib*	Aib	*Vxx*	Gln	Aib	Aib	Aib	Ser	Lxx	Aib	Pro	Vxx	Aib	Aib	Gln	Gln	*Vxxol*
**Dorothopsin B XVIII**	1964.14	1237.69	726.45	27.78	0.23%	0.70%	Ac	*Trp*	Gly	*Aib*	Aib	*Lxx*	Gln	Aib	Aib	Aib	Ser	Lxx	Aib	Pro	Vxx	Aib	Aib	Gln	Gln	*Vxxol*
**Dorothopsin B XIX**	1925.13	1198.68	726.45	27.78	1.27%	3.83%	Ac	*Phe*	Gly	*Aib*	Aib	*Lxx*	Gln	Aib	Aib	Aib	Ser	Lxx	Aib	Pro	Vxx	Aib	Aib	Gln	Gln	*Vxxol*
**Dorothopsin B XX**	1978.16	1251.71	726.45	27.78	0.23%	0.69%	Ac	*Trp*	*Ala*	*Aib*	Aib	*Lxx*	Gln	Aib	Aib	Aib	Ser	Lxx	Aib	Pro	Vxx	Aib	Aib	Gln	Gln	*Vxxol*
**Dorothopsin B XXI**	1912.10	1184.67	727.43	28.01	0.14%	0.43%	Ac	*Phe*	Gly	*Aib*	Aib	*Vxx*	Gln	Aib	Aib	Aib	Ser	Lxx	Aib	Pro	Vxx	Aib	Aib	*Glu*	Gln	*Vxxol*
**Dorothopsin B XXII**	1965.12	1237.69	727.43	28.01	0.24%	0.72%	Ac	*Trp*	Gly	*Aib*	Aib	*Lxx*	Gln	Aib	Aib	Aib	Ser	Lxx	Aib	Pro	Vxx	Aib	Aib	*Glu*	Gln	*Vxxol*
**Dorothopsin B XXIII**	1926.11	1198.68	727.43	28.01	1.31%	3.93%	Ac	*Phe*	Gly	*Aib*	Aib	*Lxx*	Gln	Aib	Aib	Aib	Ser	Lxx	Aib	Pro	Vxx	Aib	Aib	*Glu*	Gln	*Vxxol*
**Dorothopsin B XXIV**	1979.14	1251.71	727.43	28.01	0.24%	0.71%	Ac	*Trp*	*Ala*	*Aib*	Aib	*Lxx*	Gln	Aib	Aib	Aib	Ser	Lxx	Aib	Pro	Vxx	Aib	Aib	*Glu*	Gln	*Vxxol*
**Dorothopsin B XXV**	1925.13	1198.68	726.45	28.58	2.05%	6.16%	Ac	*Phe*	Gly	*Aib*	Aib	*Lxx*	Gln	Aib	Aib	Aib	Ser	Lxx	Aib	Pro	Vxx	Aib	Aib	Gln	Gln	*Vxxol*
**Dorothopsin B XXVI**	1926.11	1198.68	727.43	28.73	3.38%	10.15%	Ac	*Phe*	Gly	*Aib*	Aib	*Lxx*	Gln	Aib	Aib	Aib	Ser	Lxx	Aib	Pro	Vxx	Aib	Aib	*Glu*	Gln	*Vxxol*
**Dorothopsin B XXVII**	1939.15	1212.7	726.45	29.68	1.25%	3.75%	Ac	*Phe*	*Ala*	*Aib*	Aib	*Lxx*	Gln	Aib	Aib	Aib	Ser	Lxx	Aib	Pro	Vxx	Aib	Aib	Gln	Gln	*Vxxol*
**Dorothopsin B XXVIII**	1940.13	1212.7	727.43	29.89	1.23%	3.71%	Ac	*Phe*	*Ala*	*Aib*	Aib	*Lxx*	Gln	Aib	Aib	Aib	Ser	Lxx	Aib	Pro	Vxx	Aib	Aib	*Glu*	Gln	*Vxxol*
**Dorothopsin B XXIX**	1925.13	1198.68	726.45	30.57	0.79%	2.38%	Ac	*Phe*	Gly	*Aib*	Aib	*Lxx*	Gln	Aib	Aib	Aib	Ser	Lxx	Aib	Pro	Vxx	Aib	Aib	Gln	Gln	*Vxxol*
**Dorothopsin B XXX**	1978.16	1251.71	726.45	30.57	0.16%	0.48%	Ac	*Trp*	*Ala*	*Aib*	Aib	*Lxx*	Gln	Aib	Aib	Aib	Ser	Lxx	Aib	Pro	Vxx	Aib	Aib	Gln	Gln	*Vxxol*
**Dorothopsin B XXXI**	1926.11	1198.68	727.43	30.6	1.33%	4.01%	Ac	*Phe*	Gly	*Aib*	Aib	*Lxx*	Gln	Aib	Aib	Aib	Ser	Lxx	Aib	Pro	Vxx	Aib	Aib	*Glu*	Gln	*Vxxol*
**Dorothopsin B XXXII**	1979.14	1251.71	727.43	30.6	0.27%	0.80%	Ac	*Trp*	*Ala*	*Aib*	Aib	*Lxx*	Gln	Aib	Aib	Aib	Ser	Lxx	Aib	Pro	Vxx	Aib	Aib	*Glu*	Gln	*Vxxol*
**Dorothopsin B XXXIII**	1939.15	1212.7	726.45	32.41	0.93%	2.78%	Ac	*Phe*	*Ala*	*Aib*	Aib	*Lxx*	Gln	Aib	Aib	Aib	Ser	Lxx	Aib	Pro	Vxx	Aib	Aib	Gln	Gln	*Vxxol*
**Dorothopsin B XXXIV**	1940.13	1212.7	727.43	32.56	1.51%	4.53%	Ac	*Phe*	*Ala*	*Aib*	Aib	*Lxx*	Gln	Aib	Aib	Aib	Ser	Lxx	Aib	Pro	Vxx	Aib	Aib	*Glu*	Gln	*Vxxol*
**Dorothopsin B XXXV**	1939.15	1212.7	726.45	35.05	0.23%	0.69%	Ac	*Phe*	*Ala*	*Aib*	Aib	*Lxx*	Gln	Aib	Aib	Aib	Ser	Lxx	Aib	Pro	Vxx	Aib	Aib	Gln	Gln	*Vxxol*
**Dorothopsin B XXXVI**	1940.13	1212.7	727.43	35.18	0.38%	1.15%	Ac	*Phe*	*Ala*	*Aib*	Aib	*Lxx*	Gln	Aib	Aib	Aib	Ser	Lxx	Aib	Pro	Vxx	Aib	Aib	*Glu*	Gln	*Vxxol*
*T.* cf.* strigosellum* SZMC 28007																										
**Strigosellin A I**	1870.06	1080.61	789.45	12.97	0,18%	0,48%	Ac	Aib	*Ser*	*Aib*	Ala	Aib	Gln	Aib	*Aib*	*Ala*	*Ala*	*Vxx*	Aib	Pro	*Phe*	Aib	Aib	*Glu*	Gln	*Lxxol*
**Strigosellin A II**	1869.07	1080.61	788.46	13,58	2,87%	7,41%	Ac	Aib	*Ser*	*Aib*	Ala	Aib	Gln	Aib	*Aib*	*Ala*	*Ala*	*Vxx*	Aib	Pro	*Phe*	Aib	Aib	Gln	Gln	*Lxxol*
**Strigosellin A III**	1869.07	1080.61	788.46	14,29	0,69%	1,78%	Ac	Aib	Ala	Aib	Ala	Aib	Gln	Aib	*Aib*	*Ser*	*Ala*	*Vxx*	Aib	Pro	*Phe*	Aib	Aib	Gln	Gln	*Lxxol*
**Strigosellin A IV**	1884.07	1094.62	789.45	14.57	0,19%	0,49%	Ac	Aib	*Ser*	*Aib*	Ala	Aib	Gln	Aib	*Aib*	*Ala*	*Ala*	*Lxx*	Aib	Pro	*Phe*	Aib	Aib	*Glu*	Gln	*Lxxol*
**Strigosellin A V**	1883.08	1094.62	788.46	15	1,96%	5,07%	Ac	Aib	*Ser*	*Aib*	Ala	Aib	Gln	Aib	*Aib*	*Ala*	*Ala*	*Lxx*	Aib	Pro	*Phe*	Aib	Aib	Gln	Gln	*Lxxol*
**Strigosellin A VI**	1883.09	1080.61	802.48	15,83	2,16%	5,60%	Ac	Aib	*Ser*	*Aib*	Ala	Aib	Gln	Aib	*Aib*	*Ala*	*Ala*	*Vxx*	Aib	Pro	*Phe*	*Vxx*	Aib	Gln	Gln	*Lxxol*
**Strigosellin A VII**	1854.07	1080.61	789.45	16,55	0,40%	1,03%	Ac	Aib	Ala	Aib	Ala	Aib	Gln	Aib	*Aib*	*Ser*	*Ala*	*Vxx*	Aib	Pro	*Phe*	Aib	Aib	*Glu*	Gln	*Lxxol*
** Strigosellin A VIII**	1897.1	1094.62	802.48	17,25	1,07%	2,78%	Ac	Aib	*Ser*	*Aib*	Ala	Aib	Gln	Aib	*Aib*	*Ala*	*Ala*	*Lxx*	Aib	Pro	*Phe*	*Vxx*	Aib	Gln	Gln	*Lxxol*
**Strigosellin A IX**	1853.08	1064.62	788.46	17,08	5,62%	14.55%	Ac	Aib	Ala	Aib	Ala	Aib	Gln	Aib	*Aib*	*Ala*	*Ala*	*Vxx*	Aib	Pro	*Phe*	Aib	Aib	Gln	Gln	*Lxxol*
**Strigosellin A X**	1884.07	1094.62	789.45	18.32	0,47%	1.21%	Ac	Aib	*Ser*	*Aib*	Ala	Aib	Gln	Aib	*Aib*	*Ala*	*Ala*	*Lxx*	Aib	Pro	*Phe*	Aib	Aib	*Glu*	Gln	*Lxxol*
**Strigosellin A XI**	1883.09	1080.61	802.48	18.32	0,21%	0.55%	Ac	Aib	*Ser*	*Aib*	Ala	Aib	Gln	Aib	*Aib*	*Ala*	*Ala*	*Vxx*	Aib	Pro	*Phe*	Vxx	Aib	Gln	Gln	*Lxxol*
**Strigosellin A XII**	1867.09	1078.63	788.46	18.79	4,15%	10.73%	Ac	Aib	Ala	Aib	Ala	Aib	Gln	Aib	*Aib*	*Ala*	*Ala*	*Lxx*	Aib	Pro	*Phe*	Aib	Aib	Gln	Gln	*Lxxol*
**Strigosellin A XIII**	1867.09	1064.61	802.48	19.75	5,03%	13.01%	Ac	Aib	Ala	Aib	Ala	Aib	Gln	Aib	*Aib*	*Ala*	*Ala*	*Vxx*	Aib	Pro	*Phe*	*Vxx*	Aib	Gln	Gln	*Lxxol*
**Strigosellin A XIV**	1881.11	1078.63	802.48	21.33	4,23%	10.95%	Ac	Aib	Ala	Aib	Ala	Aib	Gln	Aib	*Aib*	*Ala*	*Ala*	*Lxx*	Aib	Pro	*Phe*	*Vxx*	Aib	Gln	Gln	*Lxxol*
**Strigosellin B I**	1833,11	1078,63	754,48	22,13	2,08%	5,38%	Ac	Aib	Ala	Aib	Ala	Aib	Gln	Aib	*Aib*	*Ala*	*Ala*	Lxx	Aib	Pro	*Lxx*	Aib	Aib	Gln	Gln	*Lxxo*l
**Strigosellin B II**	1833,1	1064,61	768,49	22,45	3,17%	8,20%	Ac	Aib	Ala	Aib	Ala	Aib	Gln	Aib	*Aib*	*Ala*	*Ala*	*Vxx*	Aib	Pro	*Lxx*	Aib	*Vxx*	Gln	Gln	*Lxxol*
**Strigosellin B III**	1819,09	1064,61	754,48	23,17	0,94%	2,44%	Ac	Aib	Ala	Aib	Ala	Aib	Gln	Aib	*Aib*	*Ala*	*Ala*	*Vxx*	Aib	Pro	Vxx	Aib	*Vxx*	Gln	Gln	*Lxxol*
**Strigosellin B IV**	1833,11	1078,63	754,48	24,27	0,31%	0,80%	Ac	Aib	Ala	Aib	Ala	Aib	Gln	Aib	*Aib*	*Ala*	*Ala*	Lxx	Aib	Pro	*Lxx*	Aib	Aib	Gln	Gln	*Lxxol*
**Strigosellin B V**	1847,12	1078,63	768,49	24,92	2,51%	6,51%	Ac	Aib	Ala	Aib	Ala	Aib	Gln	Aib	*Aib*	*Ala*	*Ala*	Lxx	Aib	Pro	*Lxx*	Aib	*Vxx*	Gln	Gln	*Lxxol*
**Strigosellin B VI**	1833,11	1078,63	754,48	25,76	0,58%	1,51%	Ac	Aib	Ala	Aib	Ala	Aib	Gln	Aib	*Aib*	*Ala*	*Ala*	Lxx	Aib	Pro	Vxx	Aib	*Vxx*	Gln	Gln	*Lxxo*l

**Table 4:** Similarities of SF1 peptaibols produced by *Trichoderma* strains from clade *Viride* to known peptaibols

Peptaibol	Compound identical or positionally isomeric with	Reference
*T.* cf*. strigosellum* SZMC 28391		
**Strigaibol-like I**	Strigaibol A	[33]
**Strigaibol-like II**	**New:** Strigaibol B: [Aib]^1^ → [Vxx]^1^; [Aib]^8^ → [Ala]^8^	[33]
**Strigaibol-like III**	**New:** Strigaibol B: [Aib]^1^ → [Vxx]^1^; [Aib]^8^ → [Ala]^8^; [Gln]^16^ → [Ala]^16^	→ Strigaibol-like II
**Strigaibol-like IV**	**New:** Strigaibol B: [Aib]^1^ → [Vxx]^1^; [Lxxol]^19^ → [Vxxol]^19^	[33]
**Strigaibol-like V**	Strigaibol B	[33]
**Strigaibol-like VI**	**New:** Strigaibol D: [Aib]^1^ → [Vxx]^1^; [Ala]^2^ → [Ser]^2^ (Positional isomer of Strigaibol-like XIII)	[33]
	**New:** Strigaibol C: [Aib]^1^ → [Vxx]^1^; [Lxx]^11^ → [Vxx]^11^	[33]
**Strigaibol-like VII**	**New:** Strigaibol D: [Aib]^1^ → [Vxx]^1^; [Ala]^2^ → [Ser]^2^; [Gln]^16^ → [Ala]^16^	[33]
	**New:** Strigaibol C: [Aib]^1^ → [Vxx]^1^; [Lxx]^11^ → [Vxx]^11^; [Gln]^16^ → [Ala]^16^	[33]
**Strigaibol-like VIII**	**New:** Strigaibol E: [Lxxol]^19^ → [Vxxol]^19^	[33]
**Strigaibol-like IX**	Strigaibol E (Positional isomer of Strigaibol-like XIV, XX)	[33]
**Strigaibol-like X**	Strigaibol D	[33]
**Strigaibol-like XI**	Strigaibol C	[33]
**Strigaibol-like XII**	**New:** Strigaibol C: [Aib]^1^ → [Vxx]^1^	[33]
**Strigaibol-like XIII**	**New:** (Positional isomer of Strigaibol-like VI)	→ Strigaibol-like VI
**Strigaibol-like XIV**	(Positional isomer of Strigaibol-like IX, XX)	→ Strigaibol-like IX
**Strigaibol-like XV**	**New:** Strigaibol C: [Aib]^1^ → [Vxx]^1^; [Aib]^10^ → [Vxx]^10^	[33]
	**New:** Strigaibol G: [Aib]^1^ → [Vxx]^1^; [Ser]^10^ → [Ala]^10^	[33]
**Strigaibol-like XVI**	**New:** (Positional isomer of Strigaibol-like XII)	→ Strigaibol-like XII
**Strigaibol-like XVII**	Strigaibol F	→ Strigaibol-like X
**Strigaibol-like XVIII**	Strigaibol G	[33]
**Strigaibol-like XIX**	Strigaibol H	[33]
**Strigaibol-like XX**	(Positional isomer of Strigaibol-like IX, XIV)	→ Strigaibol-like IX
**Strigaibol-like XXI**	**New:** Strigaibol E, H: [Ala]^2^ → [Vxx]^2^	[33]
*T. koningii* SZMC 28387		
**Trikoningin KA-like I**	Pept-Ia	[38]
**Trikoningin KA-like II**	**New:** Pept-Ia: [Gln]^17^ → [Glu]^17^	[38]
**Trikoningin KA-like III**	**New:** Pept-VIa: [Gln]^17^ → [Glu]^17^	[38]
**Trikoningin KA-like IV**	**New:** Trikoningin KA V: [Ala]^3^ → [Gly]^3^	[29]
**Trikoningin KA-like V**	**New:** Trikoningin KA V: [Ala]^3^ → [Ser]^3^ (Positional isomer of Trikoningin KA-like XII)	[29]
**Trikoningin KA-like VI**	**New:** Trikoningin KA V: [Gln]^17^ → [Glu]^17^ (Positional isomer of Trikoningin KA-like XXI, XXXIX, XLI, XLV, LII)	[29]
**Trikoningin KA-like VII**	**New:** Trikoningin KA V: [ Ala]^3^ → [Ser]^3^; [Gln]^17^ → [Glu]^17^ (Positional isomer of Trikoningin KA-like XVI)	[29]
**Trikoningin KA-like VIII**	**New:** Pept-VIa: [Ala]^3^ → [Gly]^3^; [Ser]^10^ → [Ala]^10^	[38]
**Trikoningin KA-like IX**	Pept-IVa (Positional isomer of Trikoningin KA-like XIII, XXV)	[38]
**Trikoningin KA-like X**	Pept-VIa	[38]
**Trikoningin KA-like XI**	Pept-IVb (Positional isomer of Trikoningin KA-like XVIII)	[38]
**Trikoningin KA-like XII**	**New:** (Positional isomer of Trikoningin KA-like V)	→ Trikoningin KA-like V
**Trikoningin KA-like XIII**	(Positional isomer of Trikoningin KA-like XIII, XXV)	→ Trikoningin KA-like IX
**Trikoningin KA-like XIV**	Pept-Va	[38]
**Trikoningin KA-like XV**	**New:** Pept-IVb: [Gln]^17^ → [Glu]^17^ (Positional isomer of Trikoningin KA-like XX)	[38]
**Trikoningin KA-like XVI**	**New:** (Positional isomer of Trikoningin KA-like VII)	→ Trikoningin KA-like VII
**Trikoningin KA-like XVII**	**New:** Pept-Va: [Lxx]^16^ → [Vxx]^16^; [Gln]^17^ → [Glu]^17^	[38]
**Trikoningin KA-like XVIII**	(Positional isomer of Trikoningin KA-like XI)	→ Trikoningin KA-like XI
**Trikoningin KA-like XIX**	Trikoningin KA V (Positional isomer of Trikoningin KA-like XXXV, XL, XLIII, L)	[29]
**Trikoningin KA-like XX**	**New:** (Positional isomer of Trikoningin KA-like XV)	→ Trikoningin KA-like XV
**Trikoningin KA-like XXI**	**New:** (Positional isomer of Trikoningin KA-like VI, XXXIX, XLI, XLV, LII)	→ Trikoningin KA-like VI
**Trikoningin KA-like XXII**	**New:** Pept-IVb: [Ser]^10^ → [Ala]^10^	[38]
**Trikoningin KA-like XXIII**	**New:** Trikoningin KA V: [Aib]^8^ → [Ala]^8^	[29]
**Trikoningin KA-like XXIV**	**New:** Pept-IVa: [Ser]^10^ → [Ala]^10^ (Positional isomer of Trikoningin KA-like XXXII)	[38]
**Trikoningin KA-like XXV**	(Positional isomer of Trikoningin KA-like IX, XIII)	→ Trikoningin KA-like IX
**Trikoningin KA-like XXVI**	(Positional isomer of Trikoningin KA-like XIV, XXXIV)	→ Trikoningin KA-like XIV
**Trikoningin KA-like XXVII**	**New:** Pept-IVa: [Ser]^10^ → [Ala]^10^; [Gln]^17^ → [Glu]^17^	[38]
**Trikoningin KA-like XXVIII**	**New: **Trikoningin KA V: [Ala]^3^ → [Gly]^3^; [Gln]^17^ → [Glu]^17^	[29]
**Trikoningin KA-like XXIX**	**New:** Pept-IVa: [Ser]^10^ → [Ala]^10^; [Gln]^17^ → [Glu]^17^	[38]
**Trikoningin KA-like XXX**	**New:** Pept-Va: [ Aib]^4^ → [Ala]^4^; [Gln]^17^ → [Glu]^17^	[38]
**Trikoningin KA-like XXXI**	**New:** Pept-Va: [Gln]^17^ → [Glu]^17^	[38]
**Trikoningin KA-like XXXII**	**New:** (Positional isomer of Pept-XXIV)	→ Trikoningin KA-like XXIV
**Trikoningin KA-like XXXIII**	**New:** Pept-Va: [Ser]^10^ → [Ala]^10^	[38]
**Trikoningin KA-like XXXIV**	(Positional isomer of Pept-XIV, Pept-XXVI)	→ Trikoningin KA-like XIV
**Trikoningin KA-like XXXV**	(Positional isomer of Pept-XIX, Pept-XL, Pept-XLIII, Pept-L)	→ Trikoningin KA-like XIX
**Trikoningin KA-like XXXVI**	**New:** Pept-Va: [Aib]^9^ → [Ala]^9^; [Ser]^10^ → [Ala]^10^; [Gln]^17^ → [Glu]^17^	[38]
**Trikoningin KA-like XXXVII**	**New:** Pept-Va: [Ser]^10^ → [Ala]^10^; [Gln]^17^ → [Glu]^17^	[38]
**Trikoningin KA-like XXXVIII**	**New:** Pept-Va: [Gln]^17^ → [Glu]^17^	[38]
**Trikoningin KA-like XXXIX**	**New:** (Positional isomer of Trikoningin KA-like VI, XXI, XLI, XLV, LII)	→ Trikoningin KA-like VI
**Trikoningin KA-like XL**	(Positional isomer of Trikoningin KA-like XIX, XXXV, XLIII, L)	→ Trikoningin KA-like XIX
**Trikoningin KA-like XLI**	**New:** (Positional isomer of Trikoningin KA-like VI, XXI, XXXIX, XLV, LII)	→ Trikoningin KA-like VI
**Trikoningin KA-like XLII**	Pept-XVI (Positional isomer of Trikoningin KA-like XLVI)	[38]
**Trikoningin KA-like XLIII**	(Positional isomer ofTrikoningin KA-like XIX, XXXV, XL, L)	→ Trikoningin KA-like XIX
**Trikoningin KA-like XLIV**	**New:** Trikoningin KA V: [ Ser]^10^ → [Ala]^10^; [Gln]^17^ → [Glu]^17^ (Positional isomer of Trikoningin KA-like XLVIII)	[29]
**Trikoningin KA-like XLV**	**New:** (Positional isomer of Trikoningin KA-like VI, XXI, XXXIX, XLI, LII)	→ Trikoningin KA-like VI
**Trikoningin KA-like XLVI**	(Positional isomer of Trikoningin KA-like XLII)	→ Trikoningin KA-like XLII
**Trikoningin KA-like XLVII**	Pept-XI	[38]
**Trikoningin KA-like XLVIII**	**New:** (Positional isomer of Trikoningin KA-like XLIV)	→ Trikoningin KA-like XLIV
**Trikoningin KA-like XLIX**	**New:** Trikoningin KA V: [Gly]^2^ → [Ala]^2^; [Gln]^17^ → [Glu]^17^ (Positional isomer of Trikoningin KA-like LIII)	[29]
**Trikoningin KA-like L**	(Positional isomer of Trikoningin KA-like XIX, XXXV, XL, XLIII)	→ Trikoningin KA-like XIX
**Trikoningin KA-like LI**	Pept-XI	[38]
**Trikoningin KA-like LII**	**New:** (Positional isomer of Trikoningin KA-like VI, XXI, XXXIX, XLI, XLV)	→ Trikoningin KA-like VI
**Trikoningin KA-like LIII**	**New:** Trikoningin KA V: [Gly]^2^ → [Ala]^2^; [Gln]^17^ → [Glu]^17^ (Positional isomer of Trikoningin KA-like XLIX)	→ Trikoningin KA-like XLIX
**Trikoningin KA-like LIV**	**New:** Trikoningin KA V: [Gly]^2^ → [Ala]^2^; [Ser]^10^ → [Ala]^10^	[29]
**Trikoningin KA-like LV**	**New:** Trikoningin KA V: [Aib]^7^ → [Vxx]^7^	[29]
**Trikoningin KA-like LVI**	**New:** Pept-XI: [Ser]^10^ → [Ala]^10^; [Gln]^17^ → [Glu]^17^	[38]
**Trikoningin KA-like LVII**	**New:** Trikoningin KA V: [Aib]^9^ → [Vxx]^9^; [Gln]^17^ → [Glu]^17^	[29]
*T.* cf*. dorothopsis* SZMC 28390		
**Dorothopsin A-a I**	**New:** Trichostrigocin TSG-A, TSG-B: [Vxx]^16^ → [Lxx]^16^; [Gln]^17^ → [Glu]^17^; [Gln]^18^ → [Ala]^18^; [Lxxol]19 → [Alaol]19 (Positional isomer of Dorothopsin A-a II)	[37]
**Dorothopsin A-a II**	**New:** (Positional isomer of Dorothopsin A-a I)	→ Dorothopsin A-a I
**Dorothopsin A-a III**	**New:** Trichostrigocin TSG-A, TSG-B: [Lxxol]^19^ → [Vxxol]^19^ (Positional isomer of Dorothopsin A-a IV)	[37]
**Dorothopsin A-a IV**	**New:** (Positional isomer of Dorothopsin A-a III)	→ Dorothopsin A-a III
**Dorothopsin A-a V**	**New:** Trichostrigocin TSG-A, TSG-B: [Gln]^17^ → [Glu]^17^; [Lxxol]^19^ → [Vxxol]^19^	[37]
**Dorothopsin A-a VI**	**New:** Trichostrigocin TSG-A, TSG-B: [Vxx]^16^ → [Lxx]^16^; [Lxxol]^19^ → [Vxxol]^19^ (Positional isomer of Dorothopsin A-a VII)	[37]
**Dorothopsin A-a VII**	**New:** (Positional isomer of Dorothopsin A-a VI)	→ Dorothopsin A-a VI
**Dorothopsin A-a VIII**	**New:** Trichostrigocin TSG-A, TSG-B: [Gln]^17^ → [Glu]^17^; [Lxxol]^19^ → [Vxxol]^19^	[37]
**Dorothopsin A-a IX**	**New:** Trichostrigocin TSG-A, TSG-B: [Aib]^1^ → [Vxx]^1^; [Gln]^17^ → [Glu]^17^; [Lxxol]^19^ → [Vxxol]^19^	[37]
**Dorothopsin A-a X**	**New:** Trichostrigocin TSG-A, TSG-B: [Aib]^1^ → [Vxx]^1^; [Vxx]^16^ → [Lxx]^16^; [Lxxol]^19^ → [Vxxol]^19^	[37]
**Dorothopsin A-a XI**	**New:** Trichostrigocin TSG-A, TSG-B: [Aib]^1^ → [Vxx]^1^; [Vxx]^16^ → [Lxx]^16^; [Gln]^17^ → [Glu]^17^; [Lxxol]^19^ → [Vxxol]^19^	[37]
**Dorothopsin A-a XII**	**New:** Trichostrigocin TSG-A, TSG-B: [Vxx]^16^ → [Lxx]^16^	[37]
**Dorothopsin A-a XIII**	**New:** Trichostrigocin TSG-A, TSG-B: [Aib]^1^ → [Vxx]^1^; [Vxx]^16^ → [Lxx]^16^	[37]
**Dorothopsin A-b I**	**New:** Trichostrigocin TSG-A, TSG-B: [Aib]^9^; [Vxx]^16^ → [Lxx]^15^; [Gln]^17^ → [Glu]^16^; [Gln]^18^ → [Ala]^17^; [Lxxol]^19^ → [Alaol]^18^ (Positional isomer of Dorothopsin A-b II)	[37]
**Dorothopsin A-b II**	**New:** (Positional isomer of Dorothopsin A-b I)	→ Dorothopsin A-b I
**Dorothopsin A-b III**	**New:** Trichostrigocin TSG-A, TSG-B: [Aib]^9^; [Lxxol]^19^ → [Vxxol]^18^ (Positional isomer of Dorothopsin A-b V)	[37]
**Dorothopsin A-b IV**	**New:** Trichostrigocin TSG-A, TSG-B: [Aib]^9^; [Gln]^17^ → [Glu]^16^; [Lxxol]^19^ → [Vxxol]^18^ (Positional isomer of Dorothopsin A-b VI)	[37]
**Dorothopsin A-b V**	**New:** (Positional isomer of Dorothopsin A-b III)	→ Dorothopsin A-b III
**Dorothopsin A-b VI**	**New:** (Positional isomer of Dorothopsin A-b IV)	→ Dorothopsin A-b IV
**Dorothopsin A-b VII**	**New:** Trichostrigocin TSG-A, TSG-B: Aib^9^; [Vxx]^16^ → [Lxx]^15^; [Lxxol]^19^ → [Vxxol]^18^	[37]
**Dorothopsin A-b VIII**I	**New:** Trichostrigocin TSG-A, TSG-B: Aib^9^; [Vxx]^16^ → [Lxx]^15^; [Gln]^17^ → [Glu]^16^; [Lxxol]^19^ → [Vxxol]^18^	[37]
**Dorothopsin A-c I**	**New:** Trichostrigocin TSG-A, TSG-B: [Aib]^9^; [Vxx]^16^ → [Lxx]^15^; [Gln]^17^; [Gln]^18^ → [Ala]^16^; [Lxxol]^19^ → [Alaol]^17^	[37]
**Dorothopsin A-d I**	**New:** Trichostrigocin TSG-A, TSG-B: [Vxx]^16^ → [Lxx]^16^; [Gln]^17^; [Gln]^18^ → [Ala]^17^; [Lxxol]^19^ → [Alaol]^18^ (Positional isomer of Dorothopsin A-d II)	[37]
**Dorothopsin A-d II**	**New:** (Positional isomer of Dorothopsin A-d I)	→ Dorothopsin A-d I
**Dorothopsin A-d III**	**New:** Trichostrigocin TSG-A, TSG-B: [Aib]^1^ → [Vxx]^1^; [Vxx]^16^ → [Lxx]^16^; [Gln]^17^; [Gln]^18^ → [Ala]^17^; [Lxxol]^19^ → [Alaol]^18^	[37]
**Dorothopsin A-e I**	**New:** Trichostrigocin TSG-A, TSG-B: [Aib]^9^; [Vxx]^16^; [Gln]^17^; [Gln]^18^ → [Gly]^15^; [Lxxol]^19^ → [Alaol]^16^	[37]
**Dorothopsin A-f I**	**New:** Trichostrigocin TSG-A, TSG-B: [Vxx]; [Gln]^17^; [Gln]^18^ → [Gly]^16^; [Lxxol]^19^ → [Alaol]^17^ (Positional isomer of Dorothopsin A-f II)	[37]
**Dorothopsin A-f II**	**New:** (Positional isomer of Dorothopsin A-f I)	→ Dorothopsin A-f I
*T. atroviride* SZMC 28748		
**Trichorzianin TA-like I**	Trichorzianin TA IIa (Positional isomer of Trichorzianin TA-like V and VI)	[41]
**Trichorzianin TA-like II**	Trichorzianin TA IIIb; IIIc (Positional isomer of Trichorzianin TA-like VII)	[41]
**Trichorzianin TA-like III**	**New:** Trichorzianin TA IIIa: [Ala]^2^ → [Gly]^2^	[41]
**Trichorzianin TA-like IV**	**New:** Trichorzianin TA IVb: [Ala]^2^ → [Gly]^2^	[41]
**Trichorzianin TA-like V**	(Positional isomer of Trichorzianin TA-like I and VI)	→ Trichorzianin TA-like I
**Trichorzianin TA-like VI**	(Positional isomer of Trichorzianin TA-like I and V)	→ Trichorzianin TA-like I
**Trichorzianin TA-like VII**	(Positional isomer of Trichorzianin TA-like II)	→ Trichorzianin TA-like I
**Trichorzianin TA-like VIII**	**New:** Trichorzianin TA Vb: [Ala]^3^ → [Ser]^3^	[41]
**Trichorzianin TA-** **like IX**	Trichorzianin TA IVb (Positional isomer of Trichorzianin TA-like XI and XV)	[41]
**Trichorzianin TA-like X**	Trichorzianin TA IIIa (Positional isomer of Trichorzianin TA-like XIII)	[41]
**Trichorzianin TA-like XI**	(Positional isomer of Trichorzianin TA-like IX and XV)	→ Trichorzianin TA-like IX
**Trichorzianin TA-like XII**	**New:** Trichorzianin TA Vb: [Aib]^8^ → [Ala]^8^	[41]
**Trichorzianin TA-like XIII**	(Positional isomer of Trichorzianin TA-like X)	→ Trichorzianin TA-like X
**Trichorzianin TA-like XIV**	**New:** Trichorzianin TA VIb: [Ala]^3^ → [Ser]^3^	[41]
**Trichorzianin TA-like XV**	(Positional isomer of Trichorzianin TA-like IX and XI)	→ Trichorzianin TA-like IX
**Trichorzianin TA-like XVI**	**New:** Trichorzianin TA VIa: [Ala]^2^ → [Ser]^2^	[41]
**Trichorzianin TA-like XVII**	**New:** Trichorzianin TA IIIa: [Ser]^10^ → [Ala]^10^	[41]
**Trichorzianin TA-like XVIII**	**New:** Trichorzianin TA VIb: [Aib]^8^ → [Ala]^8^	[41]
**Trichorzianin TA-like XIX**	**New:** Trichorzianin TA IVb: [Ser]^10^ → [Ala]^10^	[41]
**Trichorzianin TA-like XX**	**New:** Trichorzianin TA VII: [Ala]^2^ → [Ser]^2^	[41]
**Trichorzianin TA-like XXI**	Trichorzianin TA Vb (Positional isomer of Trichorzianin TA-like XXII)	[41]
**Trichorzianin TA-like XXII**	(Positional isomer of Trichorzianin TA-like XXI)	→ Trichorzianin TA-like XXI
**Trichorzianin TA-like XXIII**	Trichorzianin TA VIb (Positional isomer of Trichorzianin TA-like XXV)	[41]
**Trichorzianin TA-like XXIV**	**New:** Trichorzianin TA VIa: [Aib]^7^ → [Ala]^7^	[41]
**Trichorzianin TA-like XXV**	(Positional isomer of Trichorzianin TA-like XXIII)	→ Trichorzianin TA-like XXIII
**Trichorzianin TA-like XXVI**	Trichorzianin TA VIa (Positional isomer of Trichorzianin TA-like XXVII and XXXII)	[41]
**Trichorzianin TA-like XXVII**	(Positional isomer of Trichorzianin TA-like XXVI and XXXII)	→ Trichorzianin TA-like XXVI
**Trichorzianin TA-like XXVIII**	Trichorzianin TA VII (Positional isomer of Trichorzianin TA-like XXIX)	[41]
**Trichorzianin TA-like XXIX**	(Positional isomer of Trichorzianin TA-like XXVIII)	→ Trichorzianin TA-like XXVIII
**Trichorzianin TA-like XXX**	**New:** Trichorzianin TA VIa: [Ser]^10^ → [Ala]^10^	[41]
**Trichorzianin TA-like XXXI**	**New:** Trichorzianin TA VII: [Ser]^10^ → [Ala]^10^	[41]
**Trichorzianin TA-like XXXII**	(Positional isomer of Trichorzianin TA-like XXVI and XXVII)	→ Trichorzianin TA-like XXVI
**Trichorzianin TA-like XXXIII**	Trichorzianin TA VII	[41]
**Peptaibol-like compounds I-XLI**	**New:** incomplete at C-terminus	Discussed in text
*T. hamatum* SZMC 28747		
**Tricholongin LB-like I**	Tricholongin LBII and Tricholongin LBIV (Positional isomer of Tricholongin LB-like XVI and XVIII)	[37]
**Tricholongin LB-like II**	**New:** Tricholongin LBI: [Phe]^3^ → [Ala]^3^	[37]
**Tricholongin LB-like III**	**New:** Tricholongin LBI: [Aib]^1^ → [Ala]^1^	[37]
**Tricholongin LB-like IV**	**New:** Tricholongin LBII and Tricholongin LBIV: [Phe]^3^ → [Ala]^3^	[37]
**Tricholongin LB-like V**	**New**: Tricholongin LBI: [Phe]^3^ → [Tyr]^3^	[37]
**Tricholongin LB-like VI**	**New:** Tricholongin LBII and Tricholongin LBIV: [Aib]^1^ → [Ala]^1^	[37]
**Tricholongin LB-like VII**	**New:** Tricholongin LBII and Tricholongin LBIV: [Phe]^3^ → [Tyr]^3^	[37]
**Tricholongin LB-like VIII**	**New:** Tricholongin LBI: [Aib]^7^ → [Ala]^7^	[37]
**Tricholongin LB-like IX**	**New:** Tricholongin LBI: [Aib]^4^ → [Ala]^4^	[37]
**Tricholongin LB-like X**	**New:** Tricholongin LBII and Tricholongin LBIV: [Aib]^5^ → [Ala]^5^	[37]
**Tricholongin LB-like XI**	Tricholongin LBI (Positional isomer of Tricholongin LB-like XIII and XV)	[37]
**Tricholongin LB-like XII**	**New:** Tricholongin LBII and Tricholongin LBIV: [Aib]^8^ → [Ala]^8^	[37]
**Tricholongin LB-like XIII**	(Positional isomer of Tricholongin LB-like XI and XV)	→ Tricholongin LB-like XI
**Tricholongin LB-like XIV**	**New:** Tricholongin LBII and Tricholongin LBIV: [Aib]^4^ → [Ala]^4^	[37]
**Tricholongin LB-like XV**	(Positional isomer of Tricholongin LB-like XI and XIII)	→ Tricholongin LB-like XI
**Tricholongin LB-like XVI**	(Positional isomer of Tricholongin LB-like I and XVIII)	→ Tricholongin LB-like I
**Tricholongin LB-like XVII**	**New:** Tricholongin LBI: [Gly]^2^ → [Ala]^2^	[37]
**Tricholongin LB-like XVIII**	(Positional isomer of Tricholongin LB-like I and XVI)	→ Tricholongin LB-like I
**Tricholongin LB-like XIX**	**New:** Tricholongin LBII and Tricholongin LBIV: [Gly]^2^ → [Ala]^2^	[37]
**Tricholongin LB-like XX**	**New:** Tricholongin LBI: [Gly]^2^ → [Ala]^2^; [Ser]^10^ → [Ala]^10^	[37]
	**New:** Tricholongin LBIII: [Gly]^2^ → [Ala]^2^; [Vxx]^16^ → [Aib]^16^	[37]
**Tricholongin LB-like XXI**	**New:** Tricholongin LBIII: [Gly]^2^ → [Ala]^2^	[37]
*T.* cf*. dorothopsis* SZMC 28005		
**Dorothopsin B I**	**New:** Tricholongin LBI: [Aib]^1^ ↔ [Phe]^3^; [Aib]^5^ → [Vxx]^5^; [Lxx]^11^ → [Vxx]^11^; [Lxxol]^19^ → [Vxxol]^19^	[37]
**Dorothopsin B II**	**New:** Tricholongin LBI: [Aib]^1^ ↔ [Phe]^3^; [Aib]^5^ → [Vxx]^5^; [Lxx]^11^ → [Vxx]^11^; [Gln]^17^ → [Glu]^17^; [Lxxol]^19^ → [Vxxol]^19^	[37]
**Dorothopsin B III**	**New:** Tricholongin LBI: [Aib]^1^ ↔ [Phe]^3^; [Aib]^5^ → [Lxx]^5^; [Aib]^8^ → [Ala]^8^; [Lxxol]^19^ → [Vxxol]^19^ (Positional isomer of Dorothopsin B IX)	[37]
**Dorothopsin B IV**	**New:** Tricholongin LBI: [Aib]^1^ ↔ [Phe]^3^; [Aib]^5^ → [Lxx]^5^; [Gln]^17^ → [Glu]^17^; [Lxxol]^19^ → [Vxxol]^19^ (Positional isomer of Dorothopsin B X)	[37]
**Dorothopsin B V**	**New:** Tricholongin LBI: [Aib]^1^ ↔ [Phe]^3^; [Aib]^5^ → [Vxx]^5^; [Lxxol]^19^ → [Vxxol]^19^ (Positional isomer of Dorothopsin B XI and Dorothopsin B XVII)	[37]
**Dorothopsin B VI**	**New:** Tricholongin LBI: [Aib]^1^ ↔ [Phe]^3^; [Aib]^5^ → [Vxx]^5^; [Gln]^17^ → [Glu]^17^; [Lxxol]^19^ → [Vxxol]^19^ (Positional isomer of Dorothopsin B XXI)	[37]
**Dorothopsin B VII**	**New:** Tricholongin LBI: [Aib]^1^ → [Trp]^1^; [Phe]^3^ → [Aib]^3^; [Aib]^5^ → [Lxx]^5^; [Lxxol]^19^ → [Vxxol]^19^ (Positional isomer of Dorothopsin B XII, Dorothopsin B XIV and Dorothopsin B XVIII)	[37]
**Dorothopsin B VIII**	**New:** Tricholongin LBI: [Aib]^1^ → [Trp]^1^; [Phe]^3^ → [Aib]^3^; [Aib]^5^ → [Lxx]^5^; [Gln]^17^ → [Glu]^17^; [Lxxol]^19^ → [Vxxol]^19^ (Positional isomer of Dorothopsin B XVI and Dorothopsin B XXII)	[37]
**Dorothopsin B IX**	**New:** (Positional isomer of Dorothopsin B III)	→ Dorothopsin B III
**Dorothopsin B X**	**New:** (Positional isomer of Dorothopsin B IV)	→ Dorothopsin B IV
**Dorothopsin B XI**	**New:** (Positional isomer of Dorothopsin B V and Dorothopsin B XVII)	→ Dorothopsin B V
**Dorothopsin B XII**	**New:** (Positional isomer of Dorothopsin B VII, Dorothopsin B XIV and Dorothopsin B XVIII)	→ Dorothopsin B VII
**Dorothopsin B XIII**	**New:** Tricholongin LBI: [Aib]^1^ ↔ [Phe]^3^; [Aib]^5^ → [Lxx]^5^; [Lxxol]^19^ → [Vxxol]^19^ (Positional isomer of Dorothopsin B XIX; Dorothopsin B XXV and Dorothopsin B XXIX)	[37]
**Dorothopsin B XIV**	**New:** (Positional isomer of Dorothopsin B VII, Dorothopsin B XII and Dorothopsin B XVIII)	→ Dorothopsin B VII
**Dorothopsin B XV**	**New:** Tricholongin LBI: [Aib]^1^ ↔ [Phe]^3^; [Aib]^5^ → [Lxx]^5^; [Gln]^17^ → [Glu]^17^; [Lxxol]^19^ → [Vxxol]^19^ (Positional isomer of Dorothopsin B XXIII; Dorothopsin B XXVI and Dorothopsin B XXXI)	[37]
**Dorothopsin B XVI**	**New:** (Positional isomer of Dorothopsin B VIII and Dorothopsin B XXII)	→ Dorothopsin B VIII
**Dorothopsin B XVII**	**New:** (Positional isomer of Dorothopsin B V and Dorothopsin B XI)	→ Dorothopsin B V
**Dorothopsin B XVIII**	**New:** (Positional isomer of Dorothopsin B VII, Dorothopsin B XII and Dorothopsin B XIV)	→ Dorothopsin B VII
**Dorothopsin B XIX**	**New:** (Positional isomer of Dorothopsin B XIII; Dorothopsin B XXV and Dorothopsin B XXIX)	→ Dorothopsin B XIII
**Dorothopsin B XX**	**New:** Tricholongin LBI: [Aib]^1^ → [Trp]^1^; [Gly]^2^ → [Ala]^2^; [Phe]^3^ → [Aib]^3^; [Aib]^5^ → [Lxx]^5^; [Lxxol]^19^ → [Vxxol]^19^ (Positional isomer of Dorothopsin B XXX)	[37]
**Dorothopsin B XXI**	**New:** (Positional isomer of Dorothopsin B VI)	→ Dorothopsin B VI
**Dorothopsin B XXII**	**New:** (Positional isomer of Dorothopsin B VIII and Dorothopsin B XVI)	→ Dorothopsin B VIII
**Dorothopsin B XXIII**	**New:** (Positional isomer of Dorothopsin B XV; Dorothopsin B XXVI and Dorothopsin B XXXI)	→ Dorothopsin B XV
**Dorothopsin B XXIV**	**New:** Tricholongin LBI: [Aib]^1^ → [Trp]^1^; [Gly]^2^ → [Ala]^2^; [Phe]^3^ → [Aib]^3^; [Aib]^5^ → [Lxx]^5^; [Gln]^17^ → [Glu]^17^; [Lxxol]^19^ → [Vxxol]^19^ (Positional isomer of Dorothopsin B XXXII)	[37]
**Dorothopsin B XXV**	**New:** (Positional isomer of Dorothopsin B XIII; Dorothopsin B XIX and Dorothopsin B XXIX)	→ Dorothopsin B XIII
**Dorothopsin B XXVI**	**New:** (Positional isomer of Dorothopsin B XV; Dorothopsin B XXIII and Dorothopsin B XXXI)	→ Dorothopsin B XV
**Dorothopsin B XXVII**	**New:** Tricholongin LBI: [Aib]^1^ ↔ [Phe]^3^; [Gly]^2^ → [Ala]^2^; [Aib]^5^ → [Lxx]^5^; [Lxxol]^19^ → [Vxxol]^19^ (Positional isomer of Dorothopsin B XXXIII and Dorothopsin B XXXV)	[37]
**Dorothopsin B XXVIII**	**New:** Tricholongin LBI: [Aib]^1^ ↔ [Phe]^3^; [Gly]^2^ → [Ala]^2^; [Aib]^5^ → [Lxx]^5^; [Gln]^17^ → [Glu]^17^; [Lxxol]^19^ → [Vxxol]^19^ (Positional isomer of Dorothopsin B XXXIV and Dorothopsin B XXXVI)	[37]
**Dorothopsin B XXIX**	**New:** (Positional isomer of Dorothopsin B XIII; Dorothopsin B XIX and Dorothopsin B XXV)	→ Dorothopsin B XIII
**Dorothopsin B XXX**	**New**: (Positional isomer of Dorothopsin B XX)	→ Dorothopsin B XX
**Dorothopsin B XXXI**	**New:** (Positional isomer of Dorothopsin B XV; Dorothopsin B XXIII and Dorothopsin B XXVI)	→ Dorothopsin B XV
**Dorothopsin B XXXII**	**New:** (Positional isomer of Dorothopsin B XXIV)	→ Dorothopsin B XXIV
**Dorothopsin B XXXIII**	**New:** (Positional isomer of Dorothopsin B XXVII and Dorothopsin B XXXV)	→ Dorothopsin B XXXIII
**Dorothopsin B XXXIV**	**New:** (Positional isomer of Dorothopsin B XXVIII and Dorothopsin B XXXVI)	→ Dorothopsin B XXXIV
**Dorothopsin B XXXV**	**New:** (Positional isomer of Dorothopsin B XXVII and Dorothopsin B XXXIII)	→ Dorothopsin B XXXIII
**Dorothopsin B XXXVI**	**New:** (Positional isomer of Dorothopsin B XXVIII and Dorothopsin B XXXIV)	→ Dorothopsin B XXXIV
**Strigosellin A I**	**New:** Trichokonin TKO-V: [Ala]^2^ → [Ser]^2^; [Vxx]^8^ → [Aib]^8^; [Aib]^9^ → [Ala]^9^; [Gly]^10^ → [Ala]^10^; [Lxx]^11^ → [Vxx]^11^; [Vxx]^14^ → [Phe]^14^; [Gln]^17^ → [Glu]^17^; [Pheol]^19^ → [Lxxol]^19^	[34]
	**New:** Trichokonin V: [Ala]^2^ → [Ser]^2^; [Val]^8^ → [Aib]^8^; [Aib]^9^ → [Ala]^9^; [Gly]^10^ → [Ala]^10^; [Leu]^11^ → [Vxx]^11^; [Val]^14^ → [Phe]^14^; [Gln]^17^ → [Glu]^17^; [Pheol]^19^ → [Lxxol]^19^	[35]
	**New:** Tricholongin BI: [Gly]^2^ → [Ser]^2^; [Phe]^3^ → [Aib]^3^; [Aib]^4^ → [Ala]^4^; [Aib]^9^ → [Ala]^9^; [Ser]^10^ → [Ala]^10^; [Leu]^11^ → [Vxx]^11^; [Val]^14^ → [Phe]^14^; [Gln]^17^ → [Glu]^17^	[36]
	**New:** Tricholongin LBI: [Gly]^2^ → [Ser]^2^; [Phe]^3^ → [Aib]^3^; [Aib]^4^ → [Ala]^4^; [Aib]^9^ → [Ala]^9^; [Ser]^10^ → [Ala]^10^; [Lxx]^11^ → [Vxx]^11^; [Vxx]^14^ → [Phe]^14^; [Gln]^17^ → [Glu]^17^	[37]
**Strigosellin A II**	**New:** Trichokonin TKO-V: [Ala]^2^ → [Ser]^2^; [Vxx]^8^ → [Aib]^8^; [Aib]^9^ → [Ala]^9^; [Gly]^10^ → [Ala]^10^; [Lxx]^11^ → [Vxx]^11^; [Vxx]^14^ → [Phe]^14^; [Pheol]^19^ → [Lxxol]^19^	[34]
	**New:** Trichokonin V: [Ala]^2^ → [Ser]^2^; [Val]^8^ → [Aib]^8^; [Aib]^9^ → [Ala]^9^; [Gly]^10^ → [Ala]^10^; [Leu]^11^ → [Vxx]^11^; [Val]^14^ → [Phe]^14^; [Pheol]^19^ → [Lxxol]^19^	[35]
	**New:** Tricholongin BI: [Gly]^2^ → [Ser]^2^; [Phe]^3^ → [Aib]^3^; [Aib]^4^ → [Ala]^4^; [Aib]^9^ → [Ala]^9^; [Ser]^10^ → [Ala]^10^; [Leu]^11^ → [Vxx]^11^; [Val]^14^ → [Phe]^14^	[36]
	**New:** Tricholongin LBI: [Gly]^2^ → [Ser]^2^; [Phe]^3^ → [Aib]^3^; [Aib]^4^ → [Ala]^4^; [Aib]^9^ → [Ala]^9^; [Ser]^10^ → [Ala]^10^; [Lxx]^11^ → [Vxx]^11^; [Vxx]^14^ → [Phe]^14^	[37]
**Strigosellin A III**	**New:** Trichokonin TKO-V: [Vxx]^8^ → [Aib]^8^; [Aib]^9^ → [Ser]^9^; [Gly]^10^ → [Ala]^10^; [Lxx]^11^ → [Vxx]^11^; [Vxx]^14^ → [Phe]^14^; [Pheol]^19^ → [Lxxol]^19^	[34]
	**New:** Trichokonin V: [Val]^8^ → [Aib]^8^; [Aib]^9^ → [Ser]^9^; [Gly]^10^ → [Ala]^10^; [Leu]^11^ → [Vxx]^11^; [Val]^14^ → [Phe]^14^; [Pheol]^19^ → [Lxxol]^19^	[35]
**Strigosellin A IV**	**New:** Trichokonin TKO-V: [Ala]^2^ → [Ser]^2^; [Vxx]^8^ → [Aib]^8^; [Aib]^9^ → [Ala]^9^; [Gly]^10^ → [Ala]^10^; [Vxx]^14^ → [Phe]^14^; [Gln]^17^ → [Glu]^17^; [Pheol]^19^ → [Lxxol]^19^ (Positional isomer of Strigosellin A X)	[34]
	**New:** Trichokonin V: [Ala]^2^ → [Ser]^2^; [Val]^8^ → [Aib]^8^; [Aib]^9^ → [Ala]^9^; [Gly]^10^ → [Ala]^10^; [Leu]^11^ → [Vxx]^11^; [Val]^14^ → [Phe]^14^; [Gln]^17^ → [Glu]^17^; [Pheol]^19^ → [Lxxol]^19^	[35]
	**New:** Tricholongin BI: [Gly]^2^ → [Ser]^2^; [Phe]^3^ → [Aib]^3^; [Aib]^4^ → [Ala]^4^; [Aib]^9^ → [Ala]^9^; [Ser]^10^ → [Ala]^10^; [Leu]^11^ → [Lxx]^11^; [Val]^14^ → [Phe]^14^; [Gln]^17^ → [Glu]^17^	[36]
	**New:** Tricholongin LBI: [Gly]^2^ → [Ser]^2^; [Phe]^3^ → [Aib]^3^; [Aib]^4^ → [Ala]^4^; [Aib]^9^ → [Ala]^9^; [Ser]^10^ → [Ala]^10^; [Vxx]^14^ → [Phe]^14^; [Gln]^17^ → [Glu]^17^	[37]
**Strigosellin A V**	**New:** Trichokonin TKO-V: [Ala]^2^ → [Ser]^2^; [Vxx]^8^ → [Aib]^8^; [Aib]^9^ → [Ala]^9^; [Gly]^10^ → [Ala]^10^; [Vxx]^14^ → [Phe]^14^; [Pheol]^19^ → [Lxxol]^19^	[34]
	**New:** Trichokonin V: [Ala]^2^ → [Ser]^2^; [Val]^8^ → [Aib]^8^; [Aib]^9^ → [Ala]^9^; [Gly]^10^ → [Ala]^10^; [Leu]^11^ → [Lxx]^11^; [Val]^14^ → [Phe]^14^; [Pheol]^19^ → [Lxxol]^19^	[35]
	**New:** Tricholongin BI: [Gly]^2^ → [Ser]^2^; [Phe]^3^ → [Aib]^3^; [Aib]^4^ → [Ala]^4^; [Aib]^9^ → [Ala]^9^; [Ser]^10^ → [Ala]^10^; [Leu]^11^ → [Lxx]^11^; [Val]^14^ → [Phe]^14^	[36]
	**New:** Tricholongin LBI: [Gly]^2^ → [Ser]^2^; [Phe]^3^ → [Aib]^3^; [Aib]^4^ → [Ala]^4^; [Aib]^9^ → [Ala]^9^; [Ser]^10^ → [Ala]^10^; [Vxx]^14^ → [Phe]^14^	[37]
**Strigosellin A VI**	**New:** Trichokonin TKO-V: [Ala]^2^ → [Ser]^2^; [Vxx]^8^ → [Aib]^8^; [Aib]^9^ → [Ala]^9^; [Gly]^10^ → [Ala]^10^; [Lxx]^11^ → [Vxx]^11^; [Vxx]^14^ → [Phe]^14^; [Aib]^15^ → [Vxx]^15^; [Pheol]^19^ → [Lxxol]^19^ (Positional isomer of Strigosellin A XI)	[34]
	**New:** Trichokonin V: [Ala]^2^ → [Ser]^2^; [Val]^8^ → [Aib]^8^; [Aib]^9^ → [Ala]^9^; [Gly]^10^ → [Ala]^10^; [Leu]^11^ → [Vxx]^11^; [Val]^14^ → [Phe]^14^; [Aib]^15^ → [Vxx]^15^; [Pheol]^19^ → [Lxxol]^19^	[35]
	**New:** Tricholongin BI: [Gly]^2^ → [Ser]^2^; [Phe]^3^ → [Aib]^3^; [Aib]^4^ → [Ala]^4^; [Aib]^9^ → [Ala]^9^; [Ser]^10^ → [Ala]^10^; [Leu]^11^ → [Lxx]^11^; [Val]^14^ → [Phe]^14^; [Aib]^15^ → [Vxx]^15^	[36]
	**New:** Tricholongin LBI: [Gly]^2^ → [Ser]^2^; [Phe]^3^ → [Aib]^3^; [Aib]^4^ → [Ala]^4^; [Aib]^9^ → [Ala]^9^; [Ser]^10^ → [Ala]^10^; [Lxx]^11^ → [Vxx]^11^; [Vxx]^14^ → [Phe]^14^; [Aib]^15^ → [Vxx]^15^	[37]
**Strigosellin A VII**	**New:** Trichokonin TKO-V: [Vxx]^8^ → [Aib]^8^; [Aib]^9^ → [Ser]^9^; [Gly]^10^ → [Ala]^10^; [Lxx]^11^ → [Vxx]^11^; [Vxx]^14^ → [Phe]^14^; [Pheol]^19^ → [Lxxol]^19^	[34]
	**New:** Trichokonin V: [Val]^8^ → [Aib]^8^; [Aib]^9^ → [Ser]^9^; [Gly]^10^ → [Ala]^10^; [Leu]^11^ → [Vxx]^11^; [Val]^14^ → [Phe]^14^; [Gln]^17^ → [Glu]^17^ [Pheol]^19^ → [Lxxol]^19^	[35]
**Strigosellin A VIII**	**New:** Trichokonin TKO-V: [Ala]^2^ → [Ser]^2^; [Vxx]^8^ → [Aib]^8^; [Aib]^9^ → [Ala]^9^; [Gly]^10^ → [Ala]^10^; [Vxx]^14^ → [Phe]^14^; [Aib]^15^ → [Vxx]^15^; [Pheol]^19^ → [Lxxol]^19^	[34]
	**New:** Trichokonin V: [Ala]^2^ → [Ser]^2^; [Val]^8^ → [Aib]^8^; [Aib]^9^ → [Ala]^9^; [Gly]^10^ → [Ala]^10^; [Leu]^11^ → [Lxx]^11^; [Val]^14^ → [Phe]^14^; [Aib]^15^ → [Vxx]^15^; [Pheol]^19^ → [Lxxol]^19^	[35]
	**New:** Tricholongin BI: [Gly]^2^ → [Ser]^2^; [Phe]^3^ → [Aib]^3^; [Aib]^4^ → [Ala]^4^; [Aib]^9^ → [Ala]^9^; [Ser]^10^ → [Ala]^10^; [Leu]^11^ → [Lxx]^11^; [Val]^14^ → [Phe]^14^; [Aib]^15^ → [Vxx]^15^	[36]
	**New:** Tricholongin LBI: [Gly]^2^ → [Ser]^2^; [Phe]^3^ → [Aib]^3^; [Aib]^4^ → [Ala]^4^; [Aib]^9^ → [Ala]^9^; [Ser]^10^ → [Ala]^10^; [Vxx]^14^ → [Phe]^14^; [Aib]^15^ → [Vxx]^15^	[37]
**Strigosellin A IX**	New: Trichokonin TKO-V: [Vxx]^8^ → [Aib]^8^; [Aib]^9^ → [Ala]^9^; [Gly]^10^ → [Ala]^10^; [Lxx]^11^ → [Vxx]^11^; [Vxx]^14^ → [Phe]^14^; [Pheol]^19^ → [Lxxol]^19^	[34]
	**New:** Trichokonin V: [Val]^8^ → [Aib]^8^; [Aib]^9^ → [Ala]^9^; [Gly]^10^ → [Ala]^10^; [Leu]^11^ → [Vxx]^11^; [Val]^14^ → [Phe]^14^; [Pheol]^19^ → [Lxxol]^19^	[35]
**Strigosellin A X**	**New:** (Positional isomer of Strigosellin A IV)	→ Strigosellin A IV
**Strigosellin A XI**	**New:** (Positional isomer of Strigosellin A VI)	→ Strigosellin A VI
**Strigosellin A XII**	**New:** Trichokonin TKO-V: [Vxx]^8^ → [Aib]^8^; [Aib]^9^ → [Ala]^9^; [Gly]^10^ → [Ala]^10^; [Vxx]^14^ → [Phe]^14^; [Pheol]^19^ → [Lxxol]^19^	[34]
	**New:** Trichokonin V: [Val]^8^ → [Aib]^8^; [Aib]^9^ → [Ala]^9^; [Gly]^10^ → [Ala]^10^; [Leu]^11^ → [Lxx]^11^; [Val]^14^ → [Phe]^14^; [Pheol]^19^ → [Lxxol]^19^	[35]
**Strigosellin A XIII**	**New:** Trichokonin TKO-V: [Vxx]^8^ → [Aib]^8^; [Aib]^9^ → [Ala]^9^; [Gly]^10^ → [Ala]^10^; [Lxx]^11^ → [Vxx]^11^; [Vxx]^14^ → [Phe]^14^; [Aib]^15^ → [Vxx]^15^; [Pheol]^19^ → [Lxxol]^19^	[34]
	**New:** Trichokonin V: [Val]^8^ → [Aib]^8^; [Aib]^9^ → [Ala]^9^; [Gly]^10^ → [Ala]^10^; [Leu]^11^ → [Vxx]^11^; [Val]^14^ → [Phe]^14^; [Aib]^15^ → [Vxx]^15^; [Pheol]^19^ → [Lxxol]^19^	[35]
**Strigosellin A XIV**	**New:** Trichokonin TKO-V: [Vxx]^8^ → [Aib]^8^; [Aib]^9^ → [Ala]^9^; [Gly]^10^ → [Ala]^10^; [Vxx]^14^ → [Phe]^14^; [Aib]^15^ → [Vxx]^15^; [Pheol]^19^ → [Lxxol]^19^	[34]
	**New:** Trichokonin V: [Val]^8^ → [Aib]^8^; [Aib]^9^ → [Ala]^9^; [Gly]^10^ → [Ala]^10^; [Leu]^11^ → [Lxx]^11^; [Val]^14^ → [Phe]^14^; [Aib]^15^ → [Vxx]^15^; [Pheol]^19^ → [Lxxol]^19^	[35]
**Strigosellin B I**	**New:** Trichokonin TKO-V: [Vxx]^8^ → [Aib]^8^; [Aib]^9^ → [Ala]^9^; [Gly]^10^ → [Ala]^10^; [Vxx]^14^ → [Lxx]^14^; [Pheol]^19^ → [Lxxol]^19^ (Positional isomer of Strigosellin B IV and Strigosellin B VI)	[34]
	**New:** Trichokonin V: [Val]^8^ → [Aib]^8^; [Aib]^9^ → [Ala]^9^; [Gly]^10^ → [Ala]^10^; [Leu]^11^ → [Lxx]^11^; [Val]^14^ → [Lxx]^14^; [Pheol]^19^ → [Lxxol]^19^	[35]
**Strigosellin B II**	**New:** Trichokonin TKO-V: [Vxx]^8^ → [Aib]^8^; [Aib]^9^ → [Ala]^9^; [Gly]^10^ → [Ala]^10^; [Lxx]^11^ → [Vxx]^11^; [Vxx]^14^ → [Lxx]^14^; [Aib]^16^ → [Vxx]^16^; [Pheol]^19^ → [Lxxol]^19^	[34]
	**New:** Trichokonin V: [Val]^8^ → [Aib]^8^; [Aib]^9^ → [Ala]^9^; [Gly]^10^ → [Ala]^10^; [Leu]^11^ → [Vxx]^11^; [Val]^14^ → [Lxx]^14^; [Aib]^16^ → [Vxx]^16^; [Pheol]^19^ → [Lxxol]^19^	[35]
**Strigosellin B III**	**New:** Trichokonin TKO-V: [Vxx]^8^ → [Aib]^8^; [Aib]^9^ → [Ala]^9^; [Gly]^10^ → [Ala]^10^; [Lxx]^11^ → [Vxx]^11^; [Aib]^16^ → [Vxx]^16^; [Pheol]^19^ → [Lxxol]^19^	[34]
	**New:** Trichokonin V: [Val]^8^ → [Aib]^8^; [Aib]^9^ → [Ala]^9^; [Gly]^10^ → [Ala]^10^; [Leu]^11^ → [Vxx]^11^; [Val]^14^ → [Lxx]^14^; [Pheol]^19^ → [Lxxol]^19^	[35]
**Strigosellin B IV**	New: (Positional isomer of Strigosellin B I and Strigosellin B VI)	→ Strigosellin B I
** Strigosellin B V**	**New: **Trichokonin TKO-V: [Vxx]^8^ → [Aib]^8^; [Aib]^9^ → [Ala]^9^; [Gly]^10^ → [Ala]^10^; [Vxx]^14^ → [Lxx]^14^; [Aib]^16^ → [Vxx]^16^; [Pheol]^19^ → [Lxxol]^19^	[34]
	New: Trichokonin V: [Val]^8^ → [Aib]^8^; [Aib]^9^ → [Ala]^9^; [Gly]^10^ → [Ala]^10^; [Leu]^11^ → [Lxx]^11^; [Val]^14^ → [Lxx]^14^; [Aib]^16^ → [Vxx]^16^; [Pheol]^19^ → [Lxxol]^19^	[35]
** Strigosellin B VI**	**New:** (Positional isomer of Strigosellin B I and Strigosellin B IV)	→ Strigosellin B I

#### Identification of new Strigaibol sequences and the new groups of Strigosellins from *T.* cf*. strigosellum*

Investigation of the peptaibol profile of *T.* cf*. strigosellum* SZMC 28391 revealed twenty-one 19-residue peptaibols belonging to the SF-1 peptaibol family (Tables 3 and 4). Appart from the known Strigaibol sequences, 14 proved to be new. Their closest positional isomers are Strigaibol sequences described by Park et al. [33]. MS and chromatographical analysis of *T.* cf*. strigosellum* SZMC 28007 revealed the production of 14 peptaibols novel to science and were named as Strigosellin A as well as further 6 sequences named as Strigosellin B peptaibols. These peptaibols showed a great similarity to the sequences of Trichokonin TKO-V, Trichokonin V, Tricholongin B and Tricholongin LBI sequences [34–37].

Strain *T.* cf*. strigosellum* SZMC 28391 produced two peptaibols in higher quantities, which were Strigaibol XII and XIV (36.15% and 27.59% of the total peptaibol production, respectively). Four more sequences were produced above 4%, Strigaibol V, VI, X and XX (5.14%, 6.44%, 4.34% and 7.2%, respectively). Strain *T.* cf*. strigosellum* SZMC 28007 produced Strigosellin A IX covering 14.55% of the total peptaibol production, while Strigosellins A XII, XIII, and XIV were also produced in relatively higher quantities (10.73%, 13.01% and 10.95%, respectively). Strigosellins A II, V, VI, and VIII, were also produced in significant quantities between 2.78 and 7.41%, while Strigosellins A I, III, IV, VII, X, and XI were produced at 0.48–1.78% of the total peptaibol production. Strigosellins B I, II and V were produced also in significant quantities (5.38%, 8.2% and 6.51%, respectively), while Strigosellin B I, B III, B IV and B VI were below 2.44%.

#### New Trikoningin KA-like peptaibol compounds from *T. koningii*

*T. koningii* SZMC 28387 produced thirty-eight new sequences, while nineteen were positionally isomeric with the previously described Trikoningin KA V from *T. koningii* described by Goulard et al. [29], and Trikoningin KA-like compounds Ia, IVa, IVb, Va, VIa and XI from *T. gamsii* SZMC 1656 described by Marik et al. [38] (Tables 3 and 4).

*T. koningii* SZMC 28387 also produced two peptaibols, Trikoningin KA-like XLIII and XLV (25.83% and 28.48%, respectively) in high quantities, while apart from Trikoningin KA-like XL, XLVI and XLVIII (4.65%, 6.25% and 6.74%, respectively), the rest of the peptaibols were produced for less than 3% and mostly below 1%.

#### Identification of the new group of Dorothopsins from *T.* cf. *dorothopsis*

Strain *T.* cf*. dorothopsis* SZMC 28390 produced thirteen 19-residue peptaibols also belonging to the SF-1 peptaibol family. All the thirteen compounds were completely new (Tables 3, 4) and their closest positional isomers are Trichostrigocins TSG-A and TSG-B described by Degenkolb et al. [37]. These new compounds were named as Dorothopsins A-a I-XIII (Table 3). However, 18-residue peptaibols, Dorothopsins A-b I-VIII and Dorothopsins A-d I-III, as well as, the 17-residue Dorothopsin A-c I and Dorothopsin A-f I-II sequences, furthermore, a 16-residue Dorothopsin A-e I sequence were also produced by this strain (Table 3). All Dorothopsin A sequences are also new additions to literature (Table 4). Strain *T.* cf*. dorothopsis* SZMC 28390 produced Dorothopsin A-a VII covering more than half of the total peptaibol production (54.05%). After that, Dorothopsins A-a VIII and A-d I, were produced the second highest with 10.48% and 8.35%, respectively. Among the Dorothopsin A-a sequences, a few peptaibols were 1.5%–4.5%, but the rest and all Dorothopsin A-b, -c, -d, -e and -f sequences were below 1.5% apart from Dorothopsin A-d I with 8.35%.

*T.* cf*. dorothopsis* SZMC 28005 produced thirty-four new sequences, which were the most unique among the already known SF1 peptaibols and named as Dorothopsin B. They had the most varied sequences, sometimes with differences in 6 positions from the already known Tricholongin LBI sequence described by Degenkolb et al. [37]. The most interesting characteristic of these sequences is the substitution of the acetylated Aib1 (Ac-Aib1) and Phe3 known from Tricholongin sequences to the acetylated Phe1 (Ac-Phe1) and Aib3. Furthermore, Phe1 was also substituted with Trp1 in several Dorothopsin B sequences. The most produced sequence by *T.* cf*. dorothopsis* SZMC 28005 was Dorothopsin B XXVI with a relatively low quantity of 10.15%, which was followed by Dorothopsins B VII, XIII, XV, XXV, XXXI, and XXXIV (5.05%, 9.71%, 7.95%, 6.16%, 4.01% and 4.53%, respectively). All the other sequences were produced below 4%.

#### Identification of new Trichorzianin TA sequences and peptaibol-like compounds from *T. atroviride*

*T. atroviride* SZMC 28748 produced thirty-three peptaibol compounds, as well as fourty-one incomplete peptaibol sequences named as Peptaibol-like compounds in Tables 3 and 4. The thirty-three peptaibol sequences were positionally isomeric with the Trichorzianin TA sequences [41], out of which, thirteen proved to be new. The Peptaibol-like sequences were also identical with the thirty-three trichorzianin TA sequences [41], but unusual at their C-terminus, where the last part of the sequences is shown with the remaining mass difference, which were Δm = 145, 146, 160, or 222 D, furthermore, all MS^2^ spectra contain the water loss [y_6_–H_2_O]^+^ fragment ions (Supplementary Fig. 2), which usually occurs when the C-terminus is an aminoalcohol. The sequences contain only two variable positions, R5 contains Aib/Vxx, while R14 Vxx/Lxx. All other residues are the same in the sequences, compared to each other. These sequences were produced in a relatively low concentration (all below 0.25%), therefore, they were not investigated further.

The two most produced peptaibols of *T. atroviride* SZMC 28748 were Trichorzianin TA-like XXV and XXIX with 15.24% and 17.59% of the total peptaibol production. They were followed by Trichorzianin TA-like VII, XV, XX, XXII, XXVII and XXVIII peptaibols with 7.68%, 8.99%, 5.26%, 5.44%, 6.19% and 5.34%, respectively. The rest of the peptaibols produced by this strain were around or below 1–2%. All the 41 Peptaibol-like compounds produced by *T. atroviride* SZMC 28748 were below 0.25% of the total peptaibiome.

#### Identification of new Tricholongin sequences from *T. hamatum*

The chromatographic and MS analysis of the *T. hamatum* SZMC 28747 extract revealed the production of twenty-one 19-residue-peptaibols belonging to the SF-1 peptaibol family (Tables 3 and 4). Among them, 6 peptaibols were new to science and the other sequences were identical to already determined sequences of Tricholongins LB I, II, III, and IV [37]. The newly identified sequences also shared similarities with the sequences of Tricholongins LB I, II, III, and IV. The known peptaibols are produced by *T.* cf. *strigosum* BBA 69577 and *T.* cf. *pubescens* BBA 66989 strains [37].

Tricholongin LB-like XIII and XVI sequences produced by *T. hamatum* SZMC 28747 covered 25.68% and 29.09% of the total peptaibol production, which were followed by Tricholongin LB-like XVII and XIX sequences (7.13% and 10.03%, respectively). All other sequences were produced mostly around 1%.

#### Module skipping hypothesis

The microheterogeneity of non-ribosomal peptides like peptaibols arises from the ability of NRPSs to incorporate various amino acids [42]. Usually, *Trichoderma* species produce 1 to 4 main peptaibol compounds in high quantities, while the rest of the peptaibol variants are in much lower quantities, which can be the result of the modular synthesis and the misassembly of the peptaibol compounds by NRPS and the lack of repair mechanisms compared to ribosomal synthesis. This may be the reason for the wide range of new peptaibols that can be identified even from already known peptaibol subgroups. This hypothesis needs a thorough genetic data support based on the number of NRPS genes that can be found in the available *Trichoderma* full genomes.

Based on the results of this study, we hypothesize that the 16, 17 and 18-residue peptaibols produced by *T.* cf*. dorothopsis* SZMC 28390 also occured through a ‘module skipping’ event, which means that the NRPS enzyme skipped a module during the synthesis [27, 39]. Two skipping mechanisms were proposed in the literature: direct intermediate transfer between nonadjacent modules caused by a “loss of function mutation” [40], or nonfunctional thiolation or condensation domains of the NRPSs during biosynthesis [39]. Module skipping is usually seen in the case of 10-, 13-, 18-, and 19-residue peptaibols. Similar results were found in the case of SF-1 peptaibols produced by *T*. *flagellatum, T*. *sinense* and *T*. *parareesei*, which produced 19-residue peptaibols among the 20-residue peptaibols, but the investigation of the genome of the producer strains revealed no extra 19-module NRPS synthetases, only the gene of a 20-module enzyme was present [27]. Based on this hypothesis, the positions at which module skipping most probably occurred are shown in Table 4 with a minus sign. Our prediction is based on comparing the sequences of 16-residue (Dorothopsin A-e), 17-residue (Dorothopsin A-c) and 18-residue (Dorothopsins A-b and A-d) peptaibols to the sequences of 19-residue peptaibols (Dorothopsin A-a) (Tables 3 and 5).

**Table 5:**
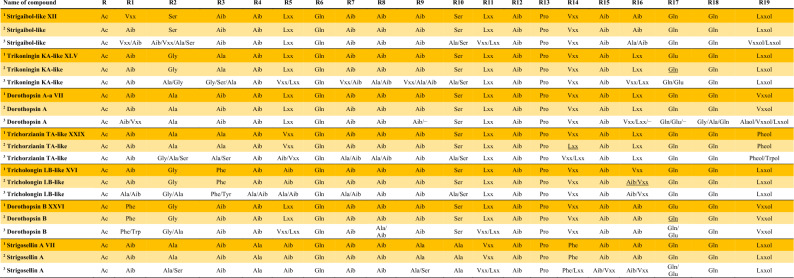
The ^1^most produced sequences, ^2^consensus sequences and ^3^amino acid varieties of the peptaibols produced by each strain

Amino acids in consensus sequences differing from the most produced compound are underlined. Darker color denotes the most produced sequences of the strain, lighter color denotes the consensus sequences denoting from the most abundant amino acid at each position

### Comparative analysis of peptaibols produced by species of clade *Viride*

The microheterogeneity within the sequences occurs in the positions where one amino acid is substituted by another one. The strictest positions were found to be in the middle of the sequences usually appearing as the Gln6-Aib7-Aib8-Aib9-Ser10-Lxx11-Aib12-Pro13-Vxx14 motif. This motif was different only in Strigosellin A sequences, where it was substituted with Gln6-Aib7-Aib8-Ala9-Ala10-Vxx11-Aib12-Pro-13-Phe14/Lxx14 produced by *T.* cf*. strigosellum* SZMC 28007. R1 usually contained an acetylated Aib except for a few *T.* cf*. strigosellum* SZMC 28391 and *T.* cf*. dorothopsis* SZMC 28390 sequences, where it was often substituted with Vxx. Acetylated Vxx is not present in SF-1 peptaibols, only in 11–16-residue peptaibols [10, 34, 37, 39]. Mostly, AcAib can be found in SF-1 peptaibols, however, several compounds produced by these strains contained another amino acid residue at the N-terminus (Table 3). Dorothopsin B sequences produced by *T.* cf*. dorothopsis* SZMC 28005 always contained Phe1 or Trp1. Boletusin 1, Chrysospermins and Peptaivirins, a group of another 19 residue-peptaibols identified from *Boletus* spp., *Apiocrea chrysosperma* and *Trichoderma* spp. (KGT142), respectively, also contained AcPhe at their N-termini [43–45]. Phe occurred strictly in R1, R3, R14 and R19 positions in *T.* cf*. dorothopsis* SZMC 28005, *T. hamatum* SZMC 28747, *T.* cf. *strigosellum* SZMC 28007 and *T. atroviride* SZMC 28748, respectively, while the other strains investigated did not contain Phe at all. The difference in Strigosellin A and B sequences relies in Phe14 and Lxx14 from *T.* cf. *strigosellum* SZMC 28007. Interestingly, both types are produced almost equally by this strain. Regularly, Phe is close to one of the termini of the sequences in all peptaibols, however, Phe is in the middle of the sequences in Strigosellin A peptaibols. This strain shows production of both Strigosellin A (Phe14) and B (Lxx14) sequences, as if the production of the regular Lxx14 containing peptaibols were replaced to the rare Phe14 sequences. AcPhe1 was often substituted with AcTrp1 in Dorothopsin sequences, also observed in the literature for 15–16-residue peptaibols (Ampullosporins and Zervamicins) produced by *Sepedonium ampullosporum* (HKI-0053) and *Emericellopsis salmosynnemata*, respectively [46, 47]. Sequences of *T. hamatum* SZMC 28747 contained Ala1 instead of Aib1 in case of only 2 sequences. Only three 20-residue sequences (Trichoaureocin 1a, Trichokonin IIb and Atroviridin D) with AcAla were identified from *T. aureoviride, T. koningii* Oudemans and an unidentified *Trichoderma* sp., respectively [35, 48, 49], out of which, the first two species belong to clade *Viride*, while the latter one was later reidentified as *T. arundinaceum* belonging to clade *Brevicompactum* [50]. Gly2 was substituted to Ala or Ser in several sequences of all strains investigated, while R3 contained mostly Ala or Aib. However, in the sequences of *T. hamatum* SZMC 28747, Phe or Tyr was always identified at the R3 position. Tyr has never been identified from peptaibols at this position, only from lipopeptaibols and other peptaibiotics, such as LP237 F5 and Trichoatrokontins, respectively [18, 51, 52], as well as from all-Aib-replaced peptides by *Amycolatopsis azurea*, used for bioconversion of AS 1387392 [53]. R4 was strictly Aib in all sequences except for a few sequences produced by *T. hamatum* SZMC 28747. At R5, mostly Vxx and Lxx were identified, except for *T. hamatum* SZMC 28747 and *T.* cf. *strigosellum* SZMC 28007 sequences, which mostly contained Aib5. This position was followed by the R6-R7-R8-R9-R10-R11 and R12-R13-R14 motifs discussed above. The module skipping occurred at R9 position in all Dorothopsin A-b, -c and -e sequences. R15 contained mostly Aib like in R4, except for a few sequences produced by *T.* cf. *strigosellum* SZMC 28007, where it was substituted to Vxx in several sequences. R16 contained either Ala, Aib, Vxx or Lxx which determined the mass of the y_7_ ion fragments. Gln16 was often substituted with Glu which, apart from the mass of the y_7_ ion fragments, defines the acidity of the peptaibols as Gln is neutral and Glu shows acidic characteristics. Dorothopsin A sequences also showed the 2^nd^ module skipping at this position. One Dorothopsin A sequence (Dorothopsin A-e I from Table 3) showed in total 3 module skippings at R9, R16 and R17. R18 mostly contained Gln except for Dorothopsin A compounds, where it was often substituted with Gly or Ala. The aminoalcohol was mostly Vxxol or Lxxol, except for Dorothopsin A and Tricholongin LB-like sequences, which often contained Alaninol (Alaol) or phenylalaninol (Pheol)/tryptophanol (Trpol) at the C-terminus, respectively. Pheol is often identified as the C-terminus of peptaibols. The first naming of peptaibols by Pandey et al. [54] as ‘peptaibophols’ was also based on the inclusion of Pheol, which was defined as peptide antibiotics containing the marker amino acid Aib and a C-terminal Pheol residue [54, 55]. After this, additional 2-alcohols such as valaninol (Valol) in TXT-A40 and Leuol in Hypelcin from *Hypocrea peltata* (current name: *T. peltatum*) were detected as C-terminal constituents, the above definition of ‘peptaibophols’ needed to be revised and updated. Nevertheless, Trpol is rare at the C-terminus of peptaibols. It was only identified from Boletusin 1, Chrysospermins, Peptaivirin A, as well as Trichorzianin TA and PA sequences [41, 43–45, 56, 57].

In a few cases, the consensus sequence (which is a created sequence based on the most abundant amino acid present at each position) was different from the most produced sequence of the strain. These residues are underlined in Table 5. Trikoningin KA-like peptaibols mostly contained Gln at R17 while the most produced Trikoningin KA-like XLV had Glu at this position. Exactly this could be observed among Dorothopsin B sequences where Dorothopsin B XXVI contained Glu instead of Gln. Similarly, Trichorzianin TA-like peptaibols at R14 contained Lxx instead of Vxx in the most produced Trichorzianin TA-like XXIX. Tricholongin LB-like sequences equally produced Aib and Vxx containing sequences at R16, however, the most produced Tricholongin LB-like XVI had Vxx at this position.

### Lipopeptaibol production of the investigated *Trichoderma* strains

Strain *T.* cf*. dorothopsis* SZMC 28390 and *T. atroviride* SZMC 28748 did not produce any lipopeptaibols (Table 1), their peptaibiomes consist of exclusively SF1 peptaibols. In Tables 6 and 7 the sequences are ordered based on their similarities to each other, the producer strains are shown in the 2^nd^ column. The diagnostic fragment ions resulted by MS^2^ fragmentation are collected in Supplementary Table 2.

**Table 6:** Lipopeptaibols produced by *Trichoderma* strains from clade *Viride* The area percentages are calculated based on the total lipopeptaibol production of each strain

Lipopeptaibols	Producer species	[M + H]^+^	RT (min)	Area (%) total peptaibiome	Area (%) lipopeptaibol	R	R1	R2	R3	R4	R5	R6	R7	R8	R9	R10	R11	R12	R13	R14	R15
**Lipostrigosellin I**	*T.* cf. strigosellum^*^	669.48	6.57	1.16%	1.91%	Oc	Aib	Gly	Lxx	Gly	Lxx	Lxxol									
**Lipostrigosellin II**	*T.* cf. strigosellum^*^	669.48	7.23	0.17%	0.28%	Oc	Aib	Gly	Lxx	Gly	Lxx	Lxxol									
**Lipostrigosellin III**	*T.* cf. strigosellum^*^	683.5	9.3	1.32%	2.17%	Oc	Aib	Gly	Lxx	Ala	Lxx	Lxxol									
**Lipostrigosellin IV**	*T.* cf. strigosellum^*^	683.5	10.54	0.17%	0.28%	Oc	Aib	Gly	Lxx	Ala	Lxx	Lxxol									
**Lipostrigocin LSG-like I**	*T.* cf. strigosellum^*^	740.52	10.96	0.14%	0.23%	Oc	Ala	Gly	Lxx	Aib	Gly	Lxx	Lxxol								
**Lipostrigocin LSG-like II**	*T.* cf. strigosellum^*^	740.52	12.15	0.18%	0.30%	Oc	Ala	Gly	Lxx	Aib	Gly	Lxx	Lxxol								
**Lipostrigocin LSG-like IIIa**	*T. hamatum*	740.52	14.34	0.83%	1.08%	Oc	Aib	Gly	*Vxx*	Aib	Gly	Lxx	Lxxol								
	*T. koningii*	740.52	13.16	0.87%	1.28%	Oc	Aib	Gly	*Vxx*	Aib	Gly	Lxx	Lxxol								
**Lipostrigocin LSG-like IIIb**	*T.* cf. strigosellum^*^	740.52	13.2	0.83%	1.37%	Oc	Aib	Gly	Lxx	Aib	Gly	Vxx	Lxxol								
**Lipostrigocin LSG-like IVa**	*T. hamatum*	740.52	15.83	0.06%	0.08%	Oc	Aib	Gly	*Vxx*	Aib	Gly	Lxx	Lxxol								
**Lipostrigocin LSG-like V**	*T. hamatum*	754.54	16.32	0.28%	0.37%	Oc	Vxx	Gly	*Vxx*	Aib	Gly	Lxx	Lxxol								
	*T. koningii*	754.54	15.06	0.82%	1.21%	Oc	Vxx	Gly	*Vxx*	Aib	Gly	Lxx	Lxxol								
**Lipostrigocin LSG-like VI**	*T. hamatum*	754.54	16.82	0.27%	0.35%	Oc	Aib	Gly	Lxx	Aib	Gly	Lxx	Lxxol								
	*T. koningii*	754.54	16.47	0.41%	0.61%	Oc	Aib	Gly	Lxx	Aib	Gly	Lxx	Lxxol								
	*T.* cf. strigosellum^*^	754.54	16.51	19.42%	31.96%	Oc	Aib	Gly	Lxx	Aib	Gly	Lxx	Lxxol								
**Lipostrigocin LSG-like VII**	*T. hamatum*	754.54	17.84	1.31%	1.71%	Oc	Aib	Gly	Lxx	Aib	Gly	Lxx	Lxxol								
	*T. koningii*	754.54	18.24	0.07%	3.05%	Oc	Aib	Gly	Lxx	Aib	Gly	Lxx	Lxxol								
	*T.* cf. strigosellum^*^	754.54	18.28	1.85%	3.70%	Oc	Aib	Gly	Lxx	Aib	Gly	Lxx	Lxxol								
**Lipostrigocin LSG-like VIIIa**	*T. hamatum*	768.55	20.56	0.90%	1.18%	Oc	Vxx	Gly	Lxx	Aib	Gly	Lxx	Lxxol								
	*T. koningii*	768.56	19.06	0.42%	0.62%	Oc	Vxx	Gly	Lxx	Aib	Gly	Lxx	Lxxol								
**Lipostrigocin LSG-like VIIIb**	*T.* cf. strigosellum^*^	768.56	19.81	22.53%	37.08%	Oc	Aib	Gly	Lxx	*Vxx*	Gly	Lxx	Lxxol								
**Lipostrigocin LSG-like IXa**	*T. hamatum*	768.55	22.51	0.07%	0.09%	Oc	Vxx	Gly	Lxx	Aib	Gly	Lxx	Lxxol								
	*T. koningii*	768.56	20.94	0.08%	0.12%	Oc	Vxx	Gly	Lxx	Aib	Gly	Lxx	Lxxol								
**Lipostrigocin LSG-like IXb**	*T.* cf. strigosellum^*^	768.56	22.05	2.12%	3.49%	Oc	Aib	Gly	Lxx	*Vxx*	Gly	Lxx	Lxxol								
**Lipostrigaibol-like I**	*T.* cf*. strigosellum*^*#*^	726.51	8.14	0.05%	0.08%	Oc	Gly	Ala	Ala	*Vxx*	Ala	Lxx	Lxxol								
**Lipostrigaibol-like II**	*T.* cf*. strigosellum*^*#*^	726.51	8.68	0.21%	0.35%	Oc	Gly	Ala	Vxx	Ala	Ala	Lxx	Lxxol								
**Lipostrigaibol-like III**	*T.* cf*. strigosellum*^*#*^	726.51	9.46	0.15%	0.25%	Oc	Gly	Ala	*Aib*	Aib	Ala	Lxx	Lxxol								
**Lipostrigaibol-like IV**	*T.* cf*. strigosellum*^*#*^	726.51	9.93	0.07%	0.11%	Oc	Gly	Ala	*Aib*	Aib	Ala	Lxx	Lxxol								
**Lipostrigaibol-like V**	*T.* cf*. strigosellum*^*#*^	726.51	10.47	0.14%	0.22%	Oc	Gly	Ala	Vxx	Aib	Ala	*Vxx*	Lxxol								
**Lipostrigaibol-like VI**	*T.* cf*. strigosellum*^*#*^	740.52	12.13	0.91%	1.49%	Oc	Gly	Ala	*Lxx*	Aib	Ala	*Vxx*	Lxxol								
**Lipostrigaibol-like VII**	*T.* cf*. strigosellum*^*#*^	740.52	12.82	0.76%	1.24%	Oc	Gly	Ala	*Lxx*	Aib	Ala	*Vxx*	Lxxol								
**Lipostrigaibol-like VIII**	*T.* cf*. strigosellum*^*#*^	740.52	13.32	3.20%	5.22%	Oc	Gly	Ala	*Lxx*	Aib	Ala	*Vxx*	Lxxol								
**Lipostrigaibol-like IX**	*T.* cf*. strigosellum*^*#*^	740.52	13.85	2.36%	3.85%	Oc	Gly	Ala	Vxx	Aib	Ala	Lxx	Lxxol								
**Lipostrigaibol-like X**	*T.* cf*. strigosellum*^*#*^	754.54	13.99	0.30%	0.49%	Oc	Gly	Ala	Vxx	Vxx	Ala	Lxx	Lxxol								
**Lipostrigaibol-like XI**	*T.* cf*. strigosellum*^*#*^	754.54	14.41	0.81%	1.31%	Oc	Gly	Ala	Vxx	Vxx	Ala	Lxx	Lxxol								
**Lipostrigaibol-like XII**	*T.* cf*. strigosellum*^*#*^	754.54	15.52	5.38%	8.77%	Oc	Gly	Ala	Vxx	Vxx	Ala	Lxx	Lxxol								
**Lipostrigaibol-like XIII**	*T.* cf*. strigosellum*^*#*^	754.54	16.29	13.59%	22.15%	Oc	Gly	Ala	Lxx	Aib	Ala	Lxx	Lxxol								
**Lipostrigaibol-like XIV**	*T.* cf*. strigosellum*^*#*^	754.54	17.25	6.12%	9.97%	Oc	Gly	Ala	Lxx	Aib	Ala	Lxx	Lxxol								
**Lipostrigaibol-like XV**	*T.* cf*. strigosellum*^*#*^	768.56	19.28	14.54%	23.70%	Oc	Gly	Ala	Lxx	Vxx	Ala	Lxx	Lxxol								
**Lipostrigaibol-like XVI**	*T.* cf*. strigosellum*^*#*^	768.56	20.75	12.76%	20.80%	Oc	Gly	Ala	Lxx	Vxx	Ala	Lxx	Lxxol								
**Lipohamatin I**	*T. hamatum*	1009.7	12.27	0.08%	0.11%	Oc	Aib	Gly	Lxx	Aib	*Gly*	*Gly*	*Vxx*	*Vxx*	*Lxx*	Lxxol					
**Lipohamatin II**	*T. hamatum*	1009.7	15.39	0.23%	0.30%	Oc	Aib	Gly	Vxx	Aib	*Gly*	*Gly*	*Lxx*	*Vxx*	Lxx	Lxxol					
**Lipohamatin III**	*T. hamatum*	1023.71	17.63	0.19%	0.25%	Oc	Aib	Gly	Lxx	Aib	*Gly*	*Gly*	*Vxx*	*Lxx*	*Lxx*	Lxxol					
**Lipohamatin IV**	*T. hamatum*	1023.71	18.06	0.15%	0.19%	Oc	Aib	Gly	Lxx	Aib	*Gly*	*Gly*	*Lxx*	*Vxx*	*Lxx*	Lxxol					
**Lipostrigocin LSG-like X**	*T. koningii*	1024.67	13.59	0.17%	0.25%	Oc	*Ala*	Gly	Vxx	Aib	Gly	Gly	Vxx	Aib	Gly	Lxx	Lxxol				
**Lipostrigocin LSG-like XI**	*T. koningii*	1024.67	14.19	0.11%	0.16%	Oc	Aib	Gly	Vxx	*Ala*	Gly	Gly	Vxx	Aib	Gly	Lxx	Lxxol				
**Lipostrigocin LSG-like XII**	*T. koningii*	1024.67	17.31	0.31%	0.45%	Oc	*Ala*	Gly	Vxx	Aib	Gly	Gly	Vxx	Aib	Gly	Lxx	Lxxol				
**Lipostrigocin LSG-like XIII**	*T. koningii*	1024.67	18.5	0.89%	1.31%	Oc	Aib	Gly	Vxx	Aib	Gly	Gly	Vxx	Aib	Gly	*Vxx*	Lxxol				
**Lipostrigocin LSG-like XIV**	*T. koningii*	1038.69	22.61	25.44%	37.46%	Oc	Aib	Gly	Vxx	Aib	Gly	Gly	Vxx	Aib	Gly	Lxx	Lxxol				
**Lipostrigocin LSG-like XV**	*T. koningii*	1052.71	23.71	6.52%	9.60%	Oc	Aib	Gly	Lxx	Aib	Gly	Gly	Vxx	Aib	Gly	Lxx	Lxxol				
**Lipostrigocin LSG-like XVI**	*T. koningii*	1052.71	24.56	7.48%	11.01%	Oc	Vxx	Gly	Vxx	Aib	Gly	Gly	Vxx	Aib	Gly	Lxx	Lxxol				
**Lipostrigocin LSG-like XVII**	*T. koningii*	1038.69	25.16	4.17%	6.13%	Oc	Aib	Gly	Vxx	Aib	Gly	Gly	Vxx	Aib	Gly	Lxx	Lxxol				
**Lipostrigocin LSG-like XVIII**	*T. koningii*	1052.71	25.91	0.87%	1.29%	Oc	Aib	Gly	Lxx	Aib	Gly	Gly	Vxx	Aib	Gly	Lxx	Lxxol				
**Lipostrigocin LSG-like XIX**	*T. koningii*	1066.72	26.25	2.79%	4.11%	Oc	Vxx	Gly	Lxx	Aib	Gly	Gly	*Vxx*	Aib	Gly	Lxx	Lxxol				
**Lipostrigocin LSG-like XX**	*T. koningii*	1052.71	27.17	7.72%	11.37%	Oc	Aib	Gly	Vxx	Aib	Gly	Gly	Lxx	Aib	Gly	Lxx	Lxxol				
**Lipostrigocin LSG-like XXI**	*T. koningii*	1066.72	28.46	2.59%	3.81%	Oc	Vxx	Gly	Lxx	Aib	Gly	Gly	*Vxx*	Aib	Gly	Lxx	Lxxol				
**Lipostrigocin LSG-like XXII**	*T. koningii*	1052.71	29.52	0.77%	1.14%	Oc	Aib	Gly	Vxx	Aib	Gly	Gly	Lxx	Aib	Gly	Lxx	Lxxol				
**Lipostrigocin LSG-like XXIII**	*T. koningii*	1066.72	29.3	3.40%	5.01%	Oc	Vxx	Gly	*Vxx*	Aib	Gly	Gly	Lxx	Aib	Gly	Lxx	Lxxol				
**Lipostrigocin LSG-like XXIV**	*T. koningii*	1066.72	30.54	0.39%	0.57%	Oc	*Aib*	Gly	Lxx	Aib	Gly	Gly	Lxx	Aib	Gly	Lxx	Lxxol				
**Lipostrigocin LSG-like XXV**	*T. koningii*	1080.74	31.17	1.10%	1.61%	Oc	Vxx	Gly	Lxx	Aib	Gly	Gly	Lxx	Aib	Gly	Lxx	Lxxol				
**Lipostrigocin LSG-like XXVI**	*T. koningii*	1066.72	31.66	0.35%	0.51%	Oc	Vxx	Gly	Vxx	Aib	Gly	Gly	Lxx	Aib	Gly	Lxx	Lxxol				
**Lipostrigocin LSG-like XXVII**	*T. koningii*	1080.74	33.24	0.08%	0.11%	Oc	Vxx	Gly	Lxx	Aib	Gly	Gly	Lxx	Aib	Gly	Lxx	Lxxol				
**Lipostrigocin LSG-like XXVIII**	*T. hamatum*	1024.67	18.83	0.14%	0.19%	Oc	*Ala*	Gly	Vxx	Aib	Gly	Gly	Vxx	Aib	Gly	Lxx	Lxxol				
**Lipostrigocin LSG-like XXIX**	*T. hamatum*	1024.67	20.06	0.41%	0.54%	Oc	Aib	Gly	Vxx	Aib	Gly	Gly	Vxx	Aib	Gly	*Vxx*	Lxxol				
**Lipostrigocin LSG-like XXX**	*T. hamatum*	1038.69	21.08	0.68%	0.89%	Oc	Aib	Gly	Lxx	Aib	Gly	Gly	Vxx	Aib	Gly	*Vxx*	Lxxol				
**Lipostrigocin LSG-like XXXI**	*T. hamatum*	1038.69	23.88	0.70%	0.91%	Oc	Aib	Gly	Vxx	Aib	Gly	Gly	Lxx	Aib	Gly	*Vxx*	Lxxol				
**Lipostrigocin LSG-like XXXII**	*T. hamatum*	1038.69	24.38	2.36%	3.08%	Oc	Aib	Gly	Vxx	Aib	Gly	Gly	Vxx	Aib	Gly	Lxx	Lxxol				
	*T.* cf*. dorothopsis*	1038.69	24.29	16.29%	24.40%	Oc	Aib	Gly	Vxx	Aib	Gly	Gly	Vxx	Aib	Gly	Lxx	Lxxol				
**Lipostrigocin LSG-like XXXIII**	*T. hamatum*	1052.71	24.63	0.40%	0.52%	Oc	*Ala*	Gly	Lxx	Aib	Gly	Gly	Lxx	Aib	Gly	Lxx	Lxxol				
**Lipostrigocin LSG-like XXXIV**	*T. hamatum*	1052.71	25.46	4.80%	6.28%	Oc	Aib	Gly	Lxx	Aib	Gly	Gly	Vxx	Aib	Gly	Lxx	Lxxol				
**Lipostrigocin LSG-like XXXV**	*T. hamatum*	1052.71	26.37	0.94%	1.23%	Oc	Vxx	Gly	Vxx	Aib	Gly	Gly	Vxx	Aib	Gly	Lxx	Lxxol				
	*T.* cf*. dorothopsis*	1052.71	26.33	10.57%	15.84%	Oc	Vxx	Gly	Vxx	Aib	Gly	Gly	Vxx	Aib	Gly	Lxx	Lxxol				
**Lipostrigocin LSG-like XXXVI**	*T. hamatum*	1052.71	27.79	0.62%	0.81%	Oc	Aib	Gly	Lxx	Aib	Gly	Gly	Vxx	Aib	Gly	Lxx	Lxxol				
**Lipostrigocin LSG-like XXXVII**	*T. hamatum*	1052.71	29.05	2.86%	3.75%	Oc	Aib	Gly	Vxx	Aib	Gly	Gly	Lxx	Aib	Gly	Lxx	Lxxol				
**Lipostrigocin LSG-like XXXVIII**	*T. hamatum*	1066.72	28.14	1.76%	2.31%	Oc	Vxx	Gly	Lxx	Aib	Gly	Gly	Vxx	Aib	Gly	Lxx	Lxxol				
**Lipostrigocin LSG-like XXXIX**	*T. hamatum*	1066.72	30.34	8.48%	11.10%	Oc	Aib	Gly	Lxx	Aib	Gly	Gly	Lxx	Aib	Gly	Lxx	Lxxol				
**Lipostrigocin LSG-like XL**	*T. hamatum*	1066.72	31.05	1.20%	1.58%	Oc	Aib	Gly	Lxx	Aib	Gly	Gly	Lxx	Aib	Gly	Lxx	Lxxol				
**Lipostrigocin LSG-like XLI**	*T. hamatum*	1066.72	32.41	1.29%	1.70%	Oc	Aib	Gly	Lxx	Aib	Gly	Gly	Lxx	Aib	Gly	Lxx	Lxxol				
**Lipostrigocin LSG-like XLII**	*T. hamatum*	1080.74	33.03	4.62%	6.05%	Oc	Vxx	Gly	Lxx	Aib	Gly	Gly	Lxx	Aib	Gly	Lxx	Lxxol				
**Lipostrigocin LSG-like XLIII**	*T. hamatum*	1080.74	35.08	0.37%	0.48%	Oc	Vxx	Gly	Lxx	Aib	Gly	Gly	Lxx	Aib	Gly	Lxx	Lxxol				
**Lipostrigocin LSG-like XLIV**	*T. hamatum*	1094.75	36.48	0.07%	0.09%	Oc	Vxx	*Ala*	Lxx	Aib	Gly	Gly	Lxx	Aib	Gly	Lxx	Lxxol				
**Lipostrigocin LSG-like XLV**	*T.* cf*. dorothopsis*	1038.69	21.9	1.29%	1.94%	Oc	Vxx	Gly	Vxx	Aib	Gly	Gly	Vxx	Aib	Gly	Vxx	Lxxol				
**Lipostrigocin LSG-like XLVI**	*T.* cf*. dorothopsis*	1038.69	26.89	14.59%	21.86%	Oc	Aib	Gly	Vxx	Aib	Gly	Gly	Vxx	Aib	Gly	Lxx	Lxxol				
**Lipostrigocin LSG-like XLVII**	*T.* cf*. dorothopsis*	1052.71	28.95	10.40%	15.58%	Oc	Vxx	Gly	Vxx	Aib	Gly	Gly	Vxx	Aib	Gly	Lxx	Lxxol				
**Trichogin GB IX-like I**	*T. hamatum*	1322.85	31.89	0.01%	0.01%	Oc	*Ala*	Gly	*Vxx*	Aib	Gly	Gly	*Vxx*	Aib	Gly	Gly	Lxx	Aib	Gly	Lxx	*Vxxol*
**Trichogin GB IX-like II**	*T. hamatum*	1336.86	32.76	0.07%	0.09%	Oc	*Ala*	Gly	Lxx	Aib	Gly	Gly	*Vxx*	Aib	Gly	Gly	*Vxx*	Aib	Gly	Lxx	Lxxol
**Trichogin GB IX-like III**	*T. hamatum*	1322.85	33.42	0.09%	0.12%	Oc	*Ala*	Gly	*Vxx*	Aib	Gly	Gly	*Vxx*	Aib	Gly	Gly	*Vxx*	Aib	Gly	Lxx	Lxxol
**Trichogin GB IX-like IV**	*T. hamatum*	1336.86	33.79	0.17%	0.22%	Oc	Aib	Gly	Lxx	Aib	Gly	Gly	*Vxx*	Aib	Gly	Gly	*Vxx*	Aib	Gly	*Vxx*	Lxxol
**Trichogin GB IX-like V**	*T. hamatum*	1322.85	34.63	0.12%	0.16%	Oc	Aib	Gly	*Vxx*	Aib	Gly	Gly	*Vxx*	Aib	Gly	Gly	*Vxx*	Aib	Gly	*Vxx*	Lxxol
**Trichogin GB IX-like VI**	*T. hamatum*	1336.86	35.39	0.26%	0.34%	Oc	Aib	Gly	*Vxx*	Aib	Gly	Gly	Lxx	Aib	Gly	Gly	*Vxx*	Aib	Gly	*Vxx*	Lxxol
**Trichogin GB IX-like VII**	*T. hamatum*	1350.87	35.39	0.26%	0.34%	Oc	Aib	Gly	Lxx	Aib	Gly	Gly	Lxx	Aib	Gly	Gly	*Vxx*	Aib	Gly	*Vxx*	Lxxol
**Trichogin GB IX-like VIII**	*T. hamatum*	1322.85	36.18	0.01%	0.02%	Oc	*Ala*	Gly	*Vxx*	Aib	Gly	Gly	*Vxx*	Aib	Gly	Gly	*Vxx*	Aib	Gly	Lxx	Lxxol
**Trichogin GB IX-like IX**	*T. hamatum*	1350.87	37.03	0.56%	0.73%	Oc	Aib	Gly	Lxx	Aib	Gly	Gly	*Vxx*	Aib	Gly	Gly	Lxx	Aib	Gly	*Vxx*	Lxxol
**Trichogin GB IX-like X**	*T. hamatum*	1336.86	37.84	0.52%	0.68%	Oc	Aib	Gly	*Vxx*	Aib	Gly	Gly	*Vxx*	Aib	Gly	Gly	Lxx	Aib	Gly	*Vxx*	Lxxol
**Trichogin GB IX-like XI**	*T. hamatum*	1350.87	38.7	2.93%	3.84%	Oc	Aib	Gly	Lxx	Aib	Gly	Gly	*Vxx*	Aib	Gly	Gly	*Vxx*	Aib	Gly	Lxx	Lxxol
**Trichogin GB IX-like XII**	*T. hamatum*	1364.89	39.19	0.60%	0.79%	Oc	Aib	Gly	Lxx	Aib	Gly	Gly	Lxx	Aib	Gly	Gly	Lxx	Aib	Gly	*Vxx*	Lxxol
**Trichogin GB IX-like XIII**	*T. hamatum*	1336.86	39.57	2.22%	2.90%	Oc	Aib	Gly	*Vxx*	Aib	Gly	Gly	*Vxx*	Aib	Gly	Gly	*Vxx*	Aib	Gly	Lxx	Lxxol
	*T.* cf*. dorothopsis*	1336.86	39.51	3.54%	5.30%	Oc	Aib	Gly	*Vxx*	Aib	Gly	Gly	*Vxx*	Aib	Gly	Gly	*Vxx*	Aib	Gly	Lxx	Lxxol
**Trichogin GB IX-like XIV**	*T. hamatum*	1364.89	40.05	2.58%	3.38%	Oc	Aib	Gly	Lxx	Aib	Gly	Gly	Lxx	Aib	Gly	Gly	*Vxx*	Aib	Gly	Lxx	Lxxol
**Trichogin GB IX-like XV**	*T. hamatum*	1350.87	40.18	2.31%	3.02%	Oc	Aib	Gly	*Vxx*	Aib	Gly	Gly	Lxx	Aib	Gly	Gly	*Vxx*	Aib	Gly	Lxx	Lxxol
**Trichogin GB IX-like XVI**	*T. hamatum*	1364.89	40.9	1.18%	1.55%	Oc	*Vxx*	Gly	Lxx	Aib	Gly	Gly	*Vxx*	Aib	Gly	Gly	*Vxx*	Aib	Gly	Lxx	Lxxol
**Trichogin GB IX-like XVII**	*T.* cf*. dorothopsis*	1350.87	41.14		3.57%	Oc	*Vxx*	Gly	*Vxx*	Aib	Gly	Gly	*Vxx*	Aib	Gly	Gly	*Vxx*	Aib	Gly	Lxx	Lxxol
**Trichogin GB IX-like XVIII**	*T. hamatum*	1350.87	41.22	1.24%	1.62%	Oc	Aib	Gly	*Vxx*	Aib	Gly	Gly	Lxx	Aib	Gly	Gly	*Vxx*	Aib	Gly	Lxx	Lxxol
**Trichogin GB IX-like XIX**	*T. hamatum*	1364.89	41.97	1.17%	1.54%	Oc	*Vxx*	Gly	*Vxx*	Aib	Gly	Gly	Lxx	Aib	Gly	Gly	*Vxx*	Aib	Gly	Lxx	Lxxol
**Trichogin GB IX-like XX**	*T. hamatum*	1336.86	42.16	0.21%	0.28%	Oc	Aib	Gly	*Vxx*	Aib	Gly	Gly	*Vxx*	Aib	Gly	Gly	*Vxx*	Aib	Gly	Lxx	Lxxol
	*T.* cf*. dorothopsis*	1336.86	42.17	4.45%	6.67%	Oc	Aib	Gly	*Vxx*	Aib	Gly	Gly	*Vxx*	Aib	Gly	Gly	*Vxx*	Aib	Gly	Lxx	Lxxol
**Trichogin GB IX-like XXI**	*T. hamatum*	1378.9	42.3	0.97%	1.28%	Oc	*Vxx*	Gly	Lxx	Aib	Gly	Gly	Lxx	Aib	Gly	Gly	*Vxx*	Aib	Gly	Lxx	Lxxol
**Trichogin GB IX-like XXII**	*T. hamatum*	1350.87	42.65	0.22%	0.29%	Oc	Aib	Gly	*Vxx*	Aib	Gly	Gly	Lxx	Aib	Gly	Gly	*Vxx*	Aib	Gly	Lxx	Lxxol
**Trichogin GB IX-like XXIII**	*T. hamatum*	1364.89	42.68	4.08%	5.34%	Oc	Aib	Gly	Lxx	Aib	Gly	Gly	*Vxx*	Aib	Gly	Gly	Lxx	Aib	Gly	Lxx	Lxxol
**Trichogin GB IX-like XXIII**	*T. hamatum*	1350.87	43.72	3.54%	4.63%	Oc	Aib	Gly	*Vxx*	Aib	Gly	Gly	*Vxx*	Aib	Gly	Gly	Lxx	Aib	Gly	Lxx	Lxxol
**Trichogin GB IX-like XXIV**	*T.* cf*. dorothopsis*	1350.87	43.86	3.23%	4.83%	Oc	*Vxx*	Gly	*Vxx*	Aib	Gly	Gly	*Vxx*	Aib	Gly	Gly	*Vxx*	Aib	Gly	Lxx	Lxxol
**Trichogin GB IX-like XXV**	*T. hamatum*	1364.89	44.91	3.20%	4.19%	Oc	Aib	Gly	*Vxx*	Aib	Gly	Gly	Lxx	Aib	Gly	Gly	Lxx	Aib	Gly	Lxx	Lxxol
**Trichogin GB IX-like XXVI**	*T. hamatum*	1378.9	44.53	5.09%	6.67%	Oc	Aib	Gly	Lxx	Aib	Gly	Gly	Lxx	Aib	Gly	Gly	Lxx	Aib	Gly	Lxx	Lxxol
**Trichogin GB IX-like XXVII**	*T. hamatum*	1364.89	45.42	1.39%	1.82%	Oc	*Vxx*	Gly	*Vxx*	Aib	Gly	Gly	*Vxx*	Aib	Gly	Gly	Lxx	Aib	Gly	Lxx	Lxxol
**Trichogin GB IX-like XXVIII**	*T. hamatum*	1350.87	45.96	0.55%	0.72%	Oc	Aib	Gly	*Vxx*	Aib	Gly	Gly	*Vxx*	Aib	Gly	Gly	Lxx	Aib	Gly	Lxx	Lxxol
**Trichogin GB IX-like XXIX**	*T. hamatum*	1378.9	46.36	2.03%	2.66%	Oc	*Vxx*	Gly	*Vxx*	Aib	Gly	Gly	Lxx	Aib	Gly	Gly	Lxx	Aib	Gly	Lxx	Lxxol
**Trichogin GB IX-like XXX**	*T. hamatum*	1392.92	46.57	1.80%	2.36%	Oc	*Vxx*	Gly	Lxx	Aib	Gly	Gly	Lxx	Aib	Gly	Gly	Lxx	Aib	Gly	Lxx	Lxxol
**Trichogin GB IX-like XXXI**	*T. hamatum*	1364.89	46.83	0.56%	0.73%	Oc	Aib	Gly	*Vxx*	Aib	Gly	Gly	Lxx	Aib	Gly	Gly	Lxx	Aib	Gly	Lxx	Lxxol
**Trichogin GB IX-like XXXII**	*T. hamatum*	1392.92	48.09	0.29%	0.38%	Oc	*Vxx*	Gly	Lxx	Aib	Gly	Gly	Lxx	Aib	Gly	Gly	Lxx	Aib	Gly	Lxx	Lxxol
**Trichogin GB IX-like XXXIII**	*T. hamatum*	1406.93	49.27	0.03%	0.04%	Oc	*Vxx*	Gly	Lxx	Aib	Gly	Gly	Lxx	*Vxx*	Gly	Gly	Lxx	Aib	Gly	Lxx	Lxxol
**Trichogin GB IX-like XXXIV**	*T. hamatum*	1406.93	52.9	0.01%	0.01%	Oc	*Lxx*	Gly	Lxx	Aib	Gly	Gly	Lxx	Aib	Gly	Gly	Lxx	Aib	Gly	Lxx	Lxxol
**Trichogin GB IX-like XXXV**	*T. hamatum*	1406.93	55.85	0.00%	0.01%	Oc	*Vxx*	Gly	Lxx	Aib	*Ala*	Gly	Lxx	Aib	Gly	Gly	Lxx	Aib	Gly	Lxx	Lxxol
**Unidentified compound (values given Δ*m*)**	*T.* cf. strigosellum^*^	814.51	10.24	10.60%	17.45%	200.1	72.0	127.1	72.0	127.1	72.0	127.1	17.0								

Amino acid exchanges in new compounds shown in Table 6 are set in italic. Lipopeptaibols produced by *T*. cf. *dorothopsis* are from strain SZMC 28005 in this table

^*^Produced by *T.* cf*. strigosellum* SZMC 28391

^#^produced by *T.* cf*. strigosellum* SZMC 28007

**Table 7:** Similarities to known compounds of lipopeptaibols produced by *Trichoderma* strains from clade *Viride*

Lipopeptaibol	Compound identical or positionally isomeric with	Reference
**Lipostrigosellin I**	**New:** Lipostrigocin LSG A3: [Aib]^4^ → (Positional isomer of Lipostrigosellin II)	[37]
**Lipostrigosellin II**	**New:** (Positional isomer of Lipostrigosellin I)	→ Lipostrigosellin I
**Lipostrigosellin III**	**New:** Lipostrigocin LSG A1: [Aib]^4^ → (Positional isomer of Lipostrigosellin IV)	[37]
**Lipostrigosellin IV**	**New:** Lipostrigocin LSG A1: [Aib]^4^ → (Positional isomer of Lipostrigosellin III)	→ Lipostrigosellin III
**Lipopeptaibol**	Compound identical or positionally isomeric with	Reference
**Lipostrigocin LSG-like I**	Lipostrigocin LSG A1 (Positional isomer of Lipostrigocin LSG-like II)	[37]
**Lipostrigocin LSG-like II**	(Positional isomer of Lipostrigocin LSG-like I)	→ Lipostrigocin LSG-like I
**Lipostrigocin LSG-like IIIa**	**New:** Lipostrigocin LSG A3: [Lxx]^3^ → [Vxx]^3^ (Positional isomer of Lipostrigocin LSG-like IVa)	[37]
**Lipostrigocin LSG-like IIIb**	Lipostrigocin LSG A2	[37]
**Lipostrigocin LSG-like Iva**	**New:** (Positional isomer of Lipostrigocin LSG-like IIIa)	→ Lipostrigocin LSG-like IIIa
**Lipostrigocin LSG-like IVb**	**New:** Lipostrigocin LSG A3: [Lxx]^3^ → [Phe]^3^	[37]
**Lipostrigocin LSG-like V**	**New:** Lipostrigocin LSG A6: [Lxx]^3^ → [Vxx]^3^	[37]
**Lipostrigocin LSG-like VI**	Lipostrigocin LSG A3	[37]
**Lipostrigocin LSG-like VII**	Lipostrigocin LSG A4	[37]
**Lipostrigocin LSG-like VIIIa**	Lipostrigocin LSG A5	[37]
**Lipostrigocin LSG-like VIIIb**	**New:** Lipostrigocin LSG A4: [Aib]^4^ → [Vxx]^4^ (Positional isomer of Lipostrigocin LSG-like IXb)	[37]
**Lipostrigocin LSG-like IXa**	Lipostrigocin LSG A6	[37]
**Lipostrigocin LSG-like IXb**	**New:** (Positional isomer of Lipostrigocin LSG-like VIIIb)	→ Lipostrigocin LSG-like VIIIb
**Lipostrigaibol-like I**	**New:** Lipostrigaibol A: [Ala]^3^ ↔ [Vxx]^4^	[33]
**Lipostrigaibol-like II**	Lipostrigaibol A	[33]
**Lipostrigaibol-like III**	**New:** Lipostrigaibol B: [Vxx]^3^ → [Aib]^3^ (Positional isomer of Lipostrigaibol-like IV)	[33]
**Lipostrigaibol-like IV**	**New:** (Positional isomer of Lipostrigaibol-like III)	→ Lipostrigaibol-like III
**Lipostrigaibol-like V**	**New:** Lipostrigaibol A: [Lxx]^6^ → [Vxx]^6^	[33]
**Lipostrigaibol-like VI**	**New:** Lipostrigaibol A: [Vxx]^3^ → [Lxx]^3^; [Lxx]^6^ → [Vxx]^6^ (Positional isomer of Lipostrigaibol-like VII and VIII)	[33]
**Lipostrigaibol-like VII**	**New:** (Positional isomer of Lipostrigaibol-like VI and VIII)	[33]
**Lipostrigaibol-like VIII**	**New:** (Positional isomer of Lipostrigaibol-like VI and VII)	→ Lipostrigaibol-like VI
**Lipostrigaibol-like IX**	Lipostrigaibol B	[33]
**Lipostrigaibol-like X**	Lipostrigaibol C (Positional isomer of Lipostrigaibol-like XI and XII)	[33]
**Lipostrigaibol-like XI**	(Positional isomer of Lipostrigaibol-like X and XII)	→ Lipostrigaibol-like X
**Lipostrigaibol-like XII**	(Positional isomer of Lipostrigaibol-like X and XI)	→ Lipostrigaibol-like X
**Lipostrigaibol-like XIII**	Lipostrigaibol D (Positional isomer of Lipostrigaibol-like XIV)	[33]
**Lipostrigaibol-like XIV**	(Positional isomer of Lipostrigaibol-like XIII)	→ Lipostrigaibol-like XIII
**Lipostrigaibol-like XV**	Lipostrigaibol E (Positional isomer of Lipostrigaibol-like XVI)	[33]
**Lipostrigaibol-like XVI**	(Positional isomer of Lipostrigaibol-like XV)	→ Lipostrigaibol-like XV
**Lipohamatin I**	**New:** Linopubescin LPB B: [Vxx]^5^ → [Gly]^5^; [Lxx]^6^ → [Gly]^6^; [Aib]^7^ → [Vxx]^7^; [Gly]^8^ → [Vxx]^8^	[37]
	**New:** Linopubescin LPB C: [Vxx]^5^ → [Gly]^5^; [Lxx]^6^ → [Gly]^6^; [Aib]^7^ → [Vxx]^7^; [Aib]^9^ → [Lxx]^9^	[37]
**Lipohamatin II**	**New:** Linopubescin LPB A: [Vxx]^5^ → [Gly]^5^; [Lxx]^6^ → [Gly]^6^; [Aib]^7^ → [Lxx]^7^; [Gly]^8^ → [Vxx]^8^	[37]
**Lipohamatin III**	**New:** Linopubescin LPB B: [Vxx]^5^ → [Gly]^5^; [Lxx]^6^ → [Gly]^6^; [Aib]^7^ → [Vxx]^7^; [Gly]^8^ → [Lxx]^8^	[37]
**Lipohamatin IV**	**New:** Linopubescin LPB B: [Vxx]^5^ → [Gly]^5^; [Lxx]^6^ → [Gly]^6^; [Aib]^7^ → [Lxx]^7^; [Gly]^8^ → [Vxx]^8^	[37]
	**New:** Linopubescin LPB C: [Vxx]^5^ → [Gly]^5^; [Lxx]^6^ → [Gly]^6^; [Aib]^7^ → [Lxx]^7^; [Aib]^9^ → [Lxx]^9^	[37]
**Lipostrigocin LSG-like X**	**New:** Trikoningin KB I: [Aib]^1^ → [Ala]^1^ (Positional isomer of Lipostrigocin LSG-like XII)	[61]
**Lipostrigocin LSG-like XI**	**New:** Trikoningin KB I: [Aib]^4^ → [Ala]^4^	[61]
**Lipostrigocin LSG-like XII**	**New:** (Positional isomer of Lipostrigocin LSG-like X)	→ Lipostrigocin LSG-like X
**Lipostrigocin LSG-like XIII**	**New:** Trikoningin KB I: [Ile]^10^ → [Vxx]^10^	[61]
**Lipostrigocin LSG-like XIV**	Lipostrigocin LSG B3 (Positional isomer of Lipostrigocin LSG-like XVII)	[37]
**Lipostrigocin LSG-like XV**	Lipostrigocin LSG B04	[37]
**Lipostrigocin LSG-like XVI**	Lipostrigocin LSG B11	[37]
**Lipostrigocin LSG-like XVII**	(Positional isomer of Lipostrigocin LSG-like XIV)	→ Lipostrigocin LSG-like XIV
**Lipostrigocin LSG-like XVIII**	Lipostrigocin LSG B06	[37]
**Lipostrigocin LSG-like XIX**	**New:** Lipostrigocin LSG B08: [Lxx]^7^ → [Vxx]^7^ (Positional isomer of Lipostrigocin LSG-like XXI)	[37]
**Lipostrigocin LSG-like XX**	Lipostrigocin LSG B03 (v2) (Positional isomer of Lipostrigocin LSG-like XXII)	[37]
**Lipostrigocin LSG-like XXI**	**New:** (Positional isomer of Lipostrigocin LSG-like XIX)	→ Lipostrigocin LSG-like XIX
**Lipostrigocin LSG-like XXII**	(Positional isomer of Lipostrigocin LSG-like XX)	→ Lipostrigocin LSG-like XX
**Lipostrigocin LSG-like XXIII**	**New:** Lipostrigocin LSG B08: [Lxx]^3^ → [Vxx]^3^	[37]
**Lipostrigocin LSG-like XXIV**	**New:** Lipostrigocin LSG B08: [Vxx]^1^ → [Aib]^1^	[37]
**Lipostrigocin LSG-like XXV**	Lipostrigocin LSG B08 (Positional isomer of Lipostrigocin LSG-like XXVII)	[37]
**Lipostrigocin LSG-like XXVI**	**New:** Lipostrigocin LSG B03 (v2): [Aib]^1^ → [Vxx]^1^	[37]
**Lipostrigocin LSG-like XXVII**	(Positional isomer of Lipostrigocin LSG-like XXV)	→ Lipostrigocin LSG-like XXV
**Lipostrigocin LSG-like XXVIII**	**New:** Lipostrigocin LSG B3: [Aib]^1^ → [Ala]^1^	[37]
**Lipostrigocin LSG-like XXIX**	**New:** Lipostrigocin LSG B3: [Lxx]^10^ → [Vxx]^10^	[37]
**Lipostrigocin LSG-like XXX**	**New:** Lipostrigocin LSG B4 and B5: [Lxx]^10^ → [Vxx]^10^	[37]
**Lipostrigocin LSG-like XXXI**	**New:** Lipostrigocin LSG B03 v2, B6 and B09: [Lxx]^10^ → [Vxx]^10^	[37]
**Lipostrigocin LSG-like XXXIII**	New: Trichogin GA IV: [Aib]^1^ → [Ala]^1^	[37]
**Lipostrigocin LSG-like XXXIV**	Lipostrigocin LSG B04	[37]
**Lipostrigocin LSG-like XXXV**	Lipostrigocin LSG B11	[37]
**Lipostrigocin LSG-like XXXVI**	Lipostrigocin LSG B05	[37]
**Lipostrigocin LSG-like XXXVII**	Lipostrigocin LSG B06	[37]
**Lipostrigocin LSG-like XXXVIII**	Lipostrigocin LSG B07	[37]
**Lipostrigocin LSG-like XXXIX**	Trichogin GA IV (Positional isomer of Lipostrigocin LSG-like XL and XLI)	[37]
**Lipostrigocin LSG-like XL**	(Positional isomer of Lipostrigocin LSG-like XXXIX and XLI)	→ Lipostrigocin LSG-like XXXIX
**Lipostrigocin LSG-like XLI**	(Positional isomer of Lipostrigocin LSG-like XXXIX and XL)	→ Lipostrigocin LSG-like XXXIX
**Lipostrigocin LSG-like XLII**	Lipostrigocin LSG B08	[37]
**Lipostrigocin LSG-like XLIII**	Lipostrigocin LSG B10	[37]
**Lipostrigocin LSG-like XLIV**	**New:** Lipostrigocin LSG B10: [Gly]^2^ → [Ala]^2^	[37]
**Lipostrigocin LSG-like XLV**	Lipostrigocin LSG B11 (Positional isomer of Lipostrigocin LSG-like XLVII)	[37]
**Lipostrigocin LSG-like XLVI**	Lipostrigocin LSG B3	[37]
**Lipostrigocin LSG-like XLVII**	(Positional isomer of Lipostrigocin LSG-like XLV)	→ Lipostrigocin LSG-like XLV
**Trichogin GB IX-like I**	**New:** Trichogin GB IX: [Aib]^1^ → [Ala]^1^; [Lxx]^3^ → [Vxx]^3^; [Lxx]^7^ → [Vxx]^7^; [Lxxol]^15^ → [Vxxol]^15^	[60]
**Trichogin GB IX-like II**	**New:** Trichogin GB IX: [Aib]^1^ → [Ala]^1^; [Lxx]^7^ → [Vxx]^7^; [Lxx]^11^ → [Vxx]^11^	[60]
**Trichogin GB IX-like III**	**New:** Trichogin GB IX: [Aib]^1^ → [Ala]^1^; [Lxx]^3^ → [Vxx]^3^; [Lxx]^7^ → [Vxx]^7^; [Lxx]^11^ → [Vxx]^11^ (Positional isomer of Trichogin GB IX-like VIII)	[60]
**Trichogin GB IX-like IV**	**New:** Trichogin GB IX: [Lxx]^7^ → [Vxx]^7^; [Lxx]^11^ → [Vxx]^11^; [Lxx]^14^ → [Vxx]^14^	[60]
**Trichogin GB IX-like V**	**New:** Trichogin GB IX: [Lxx]^3^ → [Vxx]^3^; [Lxx]^7^ → [Vxx]^7^; [Lxx]^11^ → [Vxx]^11^; [Lxx]^14^ → [Vxx]^14^	[60]
**Trichogin GB IX-like VI**	**New:** Trichogin GB IX: [Lxx]^3^ → [Vxx]^3^; [Lxx]^11^ → [Vxx]^11^; [Lxx]^14^ → [Vxx]^14^	[60]
**Trichogin GB IX-like VII**	**New:** Trichogin GB IX: [Lxx]^11^ → [Vxx]^11^; [Lxx]^14^ → [Vxx]^14^	[60]
**Trichogin GB IX-like VIII**	**New:** (Positional isomer of Trichogin GB IX-like III)	→ Trichogin GB IX-like III
**Trichogin GB IX-like IX**	**New:** Trichogin GB IX: [Lxx]^7^ → [Vxx]^7^; [Lxx]^14^ → [Vxx]^14^	[60]
**Trichogin GB IX-like X**	**New:** Trichogin GB IX: [Lxx]^3^ → [Vxx]^3^; [Lxx]^7^ → [Vxx]^7^; [Lxx]^14^ → [Vxx]^14^	[60]
**Trichogin GB IX-like XI**	**New:** Trichogin GB IX: [Lxx]^7^ → [Vxx]^7^; [Lxx]^11^ → [Vxx]^11^	[60]
**Trichogin GB IX-like XII**	**New:** Trichogin GB IX: [Lxx]^14^ → [Vxx]^14^	[60]
**Trichogin GB IX-like XIII**	**New:** Trichogin GB IX: [Lxx]^3^ → [Vxx]^3^; [Lxx]^7^ → [Vxx]^7^; [Lxx]^11^ → [Vxx]^11^ (Positional isomer of Trichogin GB IX-like XX)	[60]
**Trichogin GB IX-like XIV**	**New:** Trichogin GB IX: [Lxx]^11^ → [Vxx]^11^	[60]
**Trichogin GB IX-like XVI**	**New:** Trichogin GB IX: [Aib]^1^ → [Vxx]^1^; [Lxx]^7^ → [Vxx]^7^; [Lxx]^11^ → [Vxx]^11^	[60]
**Trichogin GB IX-like XVII**	**New:** (Positional isomer of Trichogin GB IX-like XXIV)	→ Trichogin GB IX-like XXIV
**Trichogin GB IX-like XVIII**	**New:** (Positional isomer of Trichogin GB IX-like XV and XII)	→ Trichogin GB IX-like XV
**Trichogin GB IX-like XIX**	**New:** Trichogin GB IX: [Aib]^1^ → [Vxx]^1^; [Lxx]^3^ → [Vxx]^3^; [Lxx]^11^ → [Vxx]^11^	[60]
**Trichogin GB IX-like XX**	**New:** (Positional isomer of Trichogin GB IX-like XIII)	→ Trichogin GB IX-like XIII
**Trichogin GB IX-like XXI**	**New:** Trichogin GB IX: [Aib]^1^ → [Vxx]^1^; [Lxx]^11^ → [Vxx]^11^	[60]
**Trichogin GB IX-like XXII**	**New:** (Positional isomer of Trichogin GB IX-like XV and XVIII)	→ Trichogin GB IX-like XV
**Trichogin GB IX-like XXIII**	**New:** Trichogin GB IX: [Lxx]^7^ → [Vxx]^7^	[60]
**Trichogin GB IX-like XXIV**	**New:** (Positional isomer of Trichogin GB IX-like XVII)	→ Trichogin GB IX-like XVII
**Trichogin GB IX-like XXV**	**New:** Trichogin GB IX: [Lxx]^3^ → [Vxx]^3^ (Positional isomer of Trichogin GB IX-like XXXI)	[60]
**Trichogin GB IX-like XXVI**	Trichogin GB IX	[60]
**Trichogin GB IX-like XXVII**	**New:** Trichogin GB IX: [Aib]^1^ → [Vxx]^1^; [Lxx]^3^ → [Vxx]^3^; [Lxx]^7^ → [Vxx]^7^	[60]
**Trichogin GB IX-like XXVIII**	**New:** (Positional isomer of Trichogin GB IX-like XXIII)	→ Trichogin GB IX-like XXIII
**Trichogin GB IX-like XXIX**	**New:** Trichogin GB IX: [Aib]^1^ → [Vxx]^1^; [Lxx]^3^ → [Vxx]^3^	[60]
**Trichogin GB IX-like XXX**	**New:** Trichogin GB IX: [Aib]^1^ → [Vxx]^1^ (Positional isomer of Trichogin GB IX-like XXXII)	[60]
**Trichogin GB IX-like XXXI**	**New:** (Positional isomer of Trichogin GB IX-like XXV)	→ Trichogin GB IX-like XXV
**Trichogin GB IX-like XXXII**	**New:** (Positional isomer of Trichogin GB IX-like XXX)	→ Trichogin GB IX-like XXX
**Trichogin GB IX-like XXXIII**	**New:** Trichogin GB IX: [Aib]^1^ → [Vxx]^1^; [Aib]^8^ → [Vxx]^8^	[60]
**Trichogin GB IX-like XXXIV**	**New:** Trichogin GB IX: [Aib]^1^ → [Lxx]^1^	[60]
**Trichogin GB IX-like XXXV**	**New:** Trichogin GB IX: [Aib]^1^ → [Vxx]^1^; [Gly]^5^ → [Ala]^5^	[60]

#### Identification of the new group of Lipostrigosellins from *T.* cf*. strigosellum*

*T.* cf*. strigosellum* SZMC 28391 produced a total of 12 lipopeptaibols, out of which, four were 6-residue long, while eight were 7-residue long (Table 6). Seven sequences were new, while five were positionally isomeric with already identified Lipostrigocin LSG sequences [37] (Table 7). All the 6-residue lipopeptaibols were new to science and named as Lipostrigosellin I-IV. They are different from 7-residue lipopeptaibols in skipping of Aib/Vxx4. Other *Trichoderma* species were not found to produce 6-residue lipopeptaibols. Only Halovir A-E sequences from *Scytalidium* sp. CNL240 [58] and Lipohexin from *Moeszia lindtneri* HKI-0054 [59] were identified as 6-residue lipopeptaibols. Strain *T.* cf. *strigosellum* SZMC 28391 also produced a unique compound in a relatively high amount, the amino acid composition of which could not be revealed. The mass differences (Δm) on the MS^2^ spectra are shown in Table 6, which is a repetition of 72 and 127 mass differences. Strain *T.* cf*. strigosellum* SZMC 28007 produced nine already known and seven new 7-residue sequences which were named as Lipostrigaibols I-XVI. The sequences always started with an OcGly only known from Lipostrigaibols [33]. It is followed by an Ala2, which is again unique, because this position is usually taken by Gly2. R3 and 4 were highly variable, Ala/Aib/Vxx/Lxx and Ala/Aib/Vxx were at these positions. R5 was strictly Ala and R6-7 contained either Vxx-Lxxol, or Lxx-Lxxol, which are usual in the lipopeptaibols (Table 7).

Strain *T.* cf*. strigosellum* SZMC 28391 produced two lipopeptaibols in higher quantities, which were Lipostrigocin LSG-like VIIIb and VI, with 37.08% and 31.96% of the total lipopeptaibol production, respectively (Table 6). The “unidentified compound” mentioned above was also produced in a relatively higher amount, 17.45%, compared to the rest of the lipopeptaibols, which were all below 4% (Table 6). The 7-residue lipopetaibols were more produced than the 6-residue lipopeptaibols, the latter all being below 2.17%. Three lipopeptaibols were produced in high quantities by *T.* cf. *strigosellum* SZMC 28007, Lipostrigaibols XV, XIII and XVI (23.7%, 22.15% and 20.8%, respectively) followed by Lipostrigaibols XIV and XII with 9.97% and 8.77%, respectively. The rest of the sequences were all produced below 5.22%.

#### New Lipostrigocin LSG-like sequences peptaibol compounds from *T. koningii*

*T. koningii* SZMC 28387 produced a total of 25 lipopeptaibols, out of which seven were 7-residue long while eighteen were 11-residue long (Table 6). Twelve sequences were new, while thirteen were positionally isomeric with already identified Lipostrigocin LSG sequences [37].

*T. koningii* SZMC 28387 produced more 11-residue than 7-residue lipopeptaibols, out of which Lipostrigocin LSG-like XIV was the most produced (37.46%). Lipostrigocin LSG-like XV, XVI and XX were also produced in a relatively high amount, at 9.6%, 11.01% and 11.37%, respectively. The 7-residue lipopeptaibols were produced below 1.28%.

#### Identification of the new group of Lipohamatins from *T. hamatum*

*T. hamatum* SZMC 28747 produced sixty-two 7-, 10-, 11- and 15-residue lipopeptaibols, out of which 16 compounds were known, while 47 were newly identified, out of which all the 10-residue lipopeptaibols were completely new to science and were named as Lipohamatin I-IV. They were similar to Linopubescin LPB A, B and C [37]. The 7-, and 11- residue sequences showed similarity to the Lipostrigocin LSG sequences, while the 15-residue compounds to Trichogin GB IX [37, 60], which was the only 15-residue lipopeptaibol reported.

*T. hamatum* SZMC 28747 produced the most diverse lipopeptaibol profile. The lipopeptaibol most produced by this strain was Lipostrigocin LSG-like XXXIX with 11.1%. Trichogin GB IX-like XXVI was the most produced 15-residue lipopeptaibol by this strain (6.67%). All 7- and 10-residue lipopeptaibols were produced below 1.71%.

#### New Lipostrigocin LSG-like peptaibol sequences from *T. cf. dorothopsis*

Strain *T.* cf*. dorothopsis* SZMC 28005 produced only nine lipopeptaibols, out of which five were 11-residue, four were 15-residue sequences. Similarly to the lipopeptaibols of *T. hamatum* SZMC 28747, the 11-residue sequences were positionally isomeric with Lipostrigocin LSG B3 and B11 sequences, while all the 15-residue sequences were new but similar to Trichogin GB IX [37, 60]. Two sequences, Lipostrigocin LSG-like XXXII and XXXV were also produced by *T. hamatum* SZMC 28747 (Table 6).

Strain SZMC 28005 produced four 11-residue lipopeptaibols in high quantities, which were Lipostrigocin LSG-like XXXII, XLVI, XXXV and XLVII (24.4%, 21.86%, 15.84% and 15.58%, respectively), as well as a further 11-residue compound, Lipostrigocin LSG-like XLV at 1.94%. All the other 15-residue sequences were produced between 3.57% and 6.67% of the total lipopeptaibol production.

### Comparative analysis of lipopeptaibols produced by species of clade *Viride*

The microheterogeneity within the lipopeptaibols were different from the SF1 peptaibols (Table 8). The repetitive motif Vxx/Lxx-Aib-Gly appeared in all sequences between R3-R5 in 7- and 10-residue lipopeptaibols, except for *T.* cf*. strigosellum* SZMC 28007 lipopeptaibols, where Gly5 was substituted with Ala5. In 11-residue lipopeptaibols, the R3-R5 Vxx/Lxx-Aib-Gly motif continued with a Gly with the repetition of the Vxx/Lxx-Aib-Gly motif between R7-9. In 15-residue lipopeptaibols, the Vxx/Lxx-Aib-Gly motif appeared from R3-R5, R7-9, and R11-13. Between these motifs, Gly was at positions R6 and R10. This phenomenon can also be the result of module skipping based on the sequence similarities, that the “original” lipopeptaibols are the longer ones [27, 39], however, it needs further research and proof.

**Table 8:**
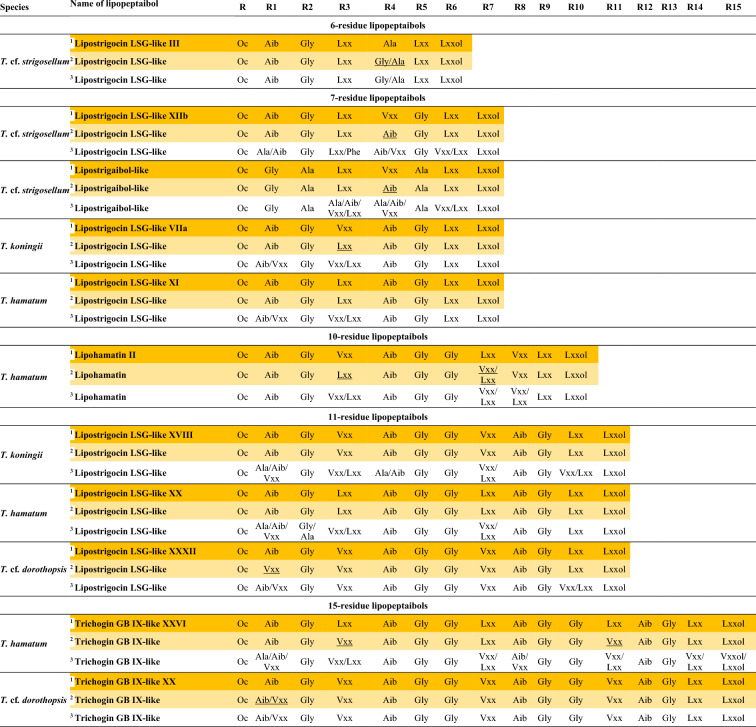
The ^1^most produced sequences, ^2^consensus sequences and ^3^amino acid varieties of lipopeptaibols produced by each strain (Lipostrigocin LSG like lipopeptaibols from *T.* cf. *strigosellum* were produced by SZMC 28391, while Lipostrigaibols were from *T.* cf. *strigosellum* SZMC 28007)

Amino acids in consensus sequences differing from the most produced compound are underlined. Darker color denotes the most produced sequences of the strain, lighter color denotes the consensus sequences showing the most abundant amino acid at each position

The 6-residue lipopeptaibols contained only one variable position at R4 which could be Gly/Ala (Table 8). Gly and Ala were present at this position equally. The same Gly/Ala variability is also present in 7-residue lipopeptaibols at R5, with Gly in Lipostrigocin LSG-like sequences and Ala in the Lipostrigaibol sequences. R1 could be Oc/Aib/OcVxx, while R6 was Vxx/Lxx. Lipostrigocin LSG-like IVb produced by *T.* cf*. strigosellum* SZMC 28391 is unique, because it has Phe at R3 instead of Lxx (Tables 6, 7 and 8). Only LP237 F7 and LP237 F8 lipopeptaibols from *Tolypocladium geodes* contain Phe3 in their sequences, however, they are 11-residue long [18, 51]. The microheterogeneity of 7-residue Lipostrigaibols appeared at R4, R5 and R6, which is discussed above. In the 10-residue Lipohamatins, Vxx/Lxx variability could be detected at R3, R7 and R8. The 11-residue Lipostrigocin LSG-like sequences showed microheterogenity at R1, R3, R4, R7 and R10 (Table 8). R1 was OcAla/OcAib/OcVxx, R4 Ala/Aib, while R3, R7 and R10 were Vxx/Lxx. The microheterogenity of the 15-residue Trichogin GA IX like lipopeptaibols was similar to the 11-residue lipopeptaibols, however, R4 was only Aib and R11 and R14 were also Vxx/Lxx (Table 8). Trichogin GB IX-like I was unique among these sequences, it contained Vxxol instead of Lxxol at its C terminus (Tables 6 and 7).

The consensus sequence was different from the most produced sequence of the two *T.* cf*. strigosellum* strains at R4, which are underlined in Table 8. At R3, Lipostrigocin LSG-like peptaibols containing mostly Lxx differed from the most produced Vxx containing Lipostrigocin LSG-like VIIa. This could also be observed among Lipohamatins where Lipohamatin II contained Vxx instead of the most abundant Lxx at R3. Lipohamatins also differed from the most produced Lipohamatin II at their R7, they contained equally Vxx and Lxx (Table 8). Lipostrigocin LSG-like peptaibols produced by T. cf. strigosellum SZMC 28391 contained Aib4, while the most produced Lipostrigocin LSG-like XIIb contained Vxx4 (Table 8). Lipostrigaibols produced by T. cf. strigosellum SZMC 28007 contained Aib4, while the most produced Lipostrigaibol-like XIIbV contained Vxx4 (Table 8). Similarly, Lipostrigocin LSG-like lipopeptaibols produced by T. koningii SZMC 28387 contained Lxx3, while the most produced Lipostrigocin LSG-like VIIa Vxx3 (Table 8). The most produced Lipohamatin II contained Vxx3 instead of the most abundant Lxx3 and Lxx7, while R7 was equally Vxx or Aib (Table 8). The most produced Trichogin GA IX like XXVI contained Lxx3 and Lxx11 instead of the most abundant Vxx3 and Vxx11 in lipopeptaibols produced by T. hamatum SZMC 28747. OcAib1was detected in Trichogin GB IX-like XX, while OcAib and OcVxx was equally present in Trichogin GB IX-like sequences produced by T. cf. dorothopsis SZMC 28005.

### Quantitative analysis of the whole peptaibiome produced by the members of clade *Viride*

Tables 3 and 6 also contain the integrated area of each peak belonging to each compound produced by the strains investigated. The values are based on the integrated area under each peak. In the previous sections, the peptaibols and lipopeptaibols were quantified based on the percentages of the total peptaibol and lipopeptaibol production separately, however, it is also important to quantify the entire peptaibiom altogether. *T.* cf*. dorothopsis* SZMC 28390 produced exculively peptaibols, which quantity was discussed above, while *T. atroviride* SZMC 28748 also produced peptaibol-like sequences apart from peptaibols, but no lipopeptaibols. Within the strains investigated in clade *Viride*, one peptaibol subgroup was produced by each strain, on the other hand, a few lipopeptaibols were the same produced by different strains, which are shown in Table 6. Strains *T. koningii* SZMC 28387, *T.* cf*. strigosellum* SZMC 28007 and *T. hamatum* SZMC 28747 all produced the 7-residue Lipostrigocins LSG VI and VII, while only *T. koningii* SZMC 28387 and *T. hamatum* SZMC 28747 produced Lipostrigocins LSG IIIa, IVa, V, VIIIa and IXa. *T.* cf. *strigosellum* SZMC 28007 produced similar lipopeptaibols only differing in 1 amino acid exchange from the ‘a’ type variants, which are Lipostrigocins LSG IIIb, IVb, VIIIb and IXb (Table 6). Strains *T. hamatum* SZMC 28747 and *T.* cf*. dorothopsis* SZMC 28005 produced the same 11-residue Lipostrigocin LSG-like XXXII and XXXV, and the 15-residue Trichogin GB IX-like XIII and XX lipopeptaibols.

*T. koningii* SZMC 28387 produced only two peptaibol compounds in higher abundances, Trikoningins KA-like XLV and XLIII (9.14% and 8.28%, respectively). More lipopeptaibols were produced in higher quantities, Lipostrigocin LSG-like XIV (25.44%) is the most produced, followed by Lipostrigocin LSG-like XX (7.72%), Lipostrigocin LSG-like XVI (7.48%) and Lipostrigocin LSG-like XV (6.52%). The rest of the peptaibiome for *T. koningii* ranges from 0.002% to 4.17%. Strigaibol-like XII from *T.* cf*. strigosellum* SZMC 28391 appeared as the most produced peptaibol (14.19%), followed by Strigaibol-like XIV (10.83%). The most produced lipopeptaibol by this strain is Lipostrigocin LSG-like VIIIb (22.53%), followed by Lipostrigocin LSG-like VI (19.42%). The “unidentified compound” was also produced at higher quantity (10.6%). The rest of the peptaibiome ranges from 0,03 to 2.53%. Among *T. atroviride* SZMC 28748 peptaibols, the most produced compound is Trichorzianin TA-like XXIX (8.56%) followed by Trichorzianin TA-like XXV (7.42%). The rest of the peptaibiome ranged from 0.03 to 4.37%. The peptaibol-like compounds were produced in much less quantities ranging from 0.003 to 0.22%. *T. hamatum* SZMC 28747 produced Tricholongin LB-like XVI (6.87%) as the most produced peptaibol, which was followed by Tricholongin LB-like XIII (6.07%). This strain also produced several lipopeptaibols in high amounts, the most produced being Lipostrigocin LSG-like XXXIX (8.48%) followed by Lipostrigocin LSG-like XXXIV (4.8%) and Lipostrigocin LSG-like XLII (4.62%). Other lipopeptaibols, Lipostrigocin LSG-like XXXVII, Trichogin GB IX-like XI, Trichogin GB IX-like XXIII, Trichogin GB IX-like XXIII, Trichogin GB IX-like XXV and Trichogin GB IX-like XXVI were also produced in high quantities rarnging from 2.87% to 4.08%. This was the only investigated strain, which produced these many compounds in high amounts. The most produced peptaibol of *T.* cf*. dorothopsis* SZMC 28005 is Dorothopsin B XXVI (3.38%) and Dorothopsin B XIII (3.23%), which is much less than the most produced lipopeptaibols, Lipostrigocin LSG-like XXXII (16.29%), Lipostrigocin LSG-like XLVI (14.59%), Lipostrigocin LSG-like XXXV (10.57%) and Lipostrigocin LSG-like XLVII (10.4%). *T.* cf*. strigosellum* SZMC 28007 produced less peptaibols than lipopeptaibols similarly to *T.* cf*. dorothopsis* SZMC 28005. Strigosellin A IX (5.62%) was the most produced peptaibol followed by Strigosellin A XIII (5.03%), Strigosellin A XIV (4.23%) and Strigosellin A XII (4.15%). The most produced lipopeptaibols are Lipostrigaibol-like XV (14.54%), Lipostrigaibol-like XIII (13.59%) and Lipostrigaibol-like XVI (12.76%). The rest of the peptaibiome ranges from 0.05% to 6.12%.

### Structural investigation of a representative peptaibol of clade *Viride*, Trikoningin KA V produced by *T. koningii*

**Fig. 2:**
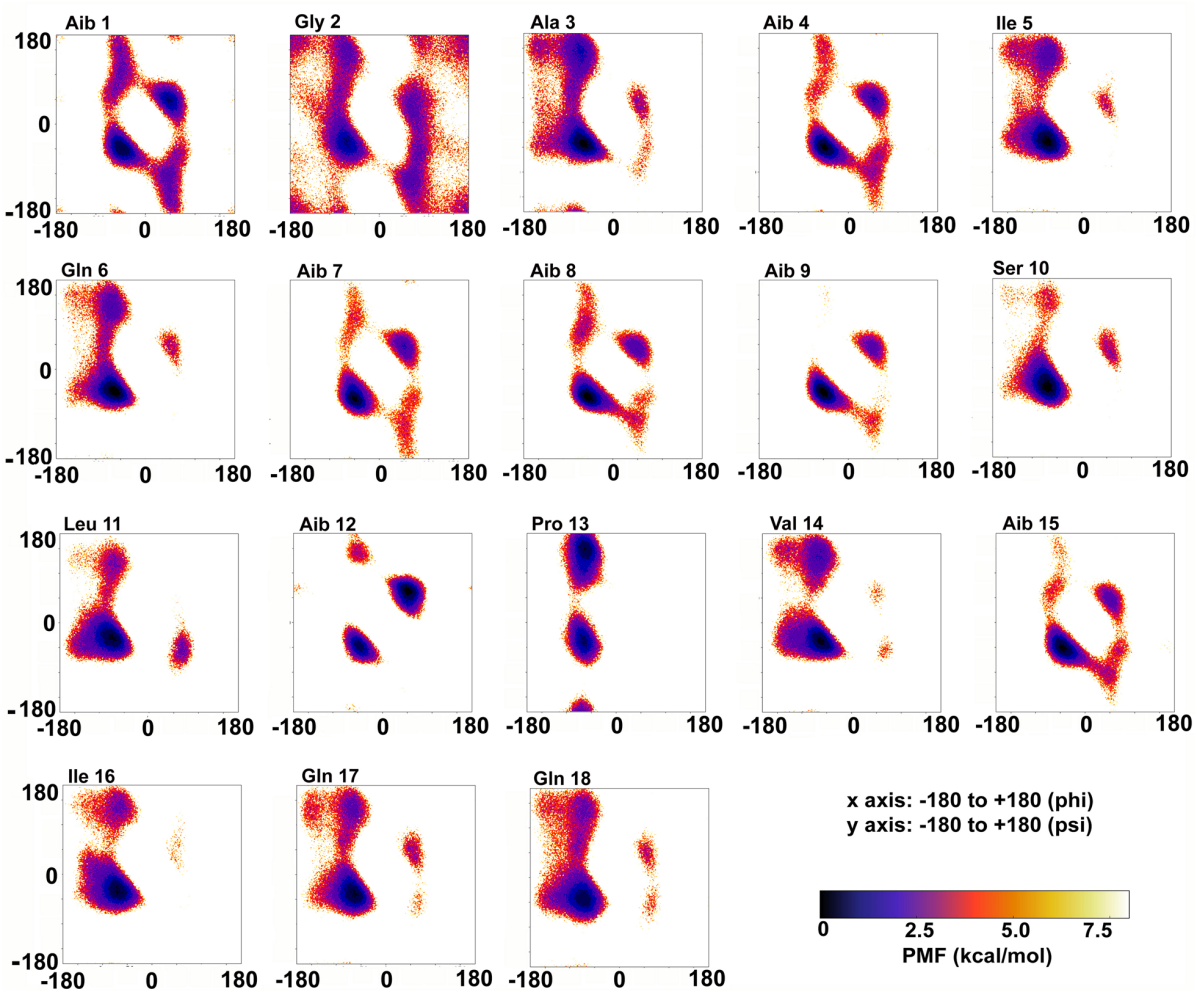
The reweighted phi-psi torsional distribution for each residue of TRK-V. Most residues show an energy minimum in the right-handed α-helical region

Strain *T. koningii* SZMC 28387 produced Trikoningin KA-like peptaibols out of which Trikoningin KA-like XIX and its positional isomers (Trikoningin KA-like XXXV, XL, XLIII and L) from Tables 3 and 4 show the same sequences as Trikoningin KA (TRK-V) identified by Goulard et al. [29]. They also determined the isomers of Lxx and Vxx, and since TRK-V is a good representation of peptaibols produced by members of clade *Viride*, this sequence was selected for aMD simulations. Auvin-Guette et al. [61] firstly reported the structure of TRK-V to be “right-handed helical form” using circular dichroism spectroscopy and was found to membrane active and displayed antibiotic activity against *Staphylococcus aureus*. We previously reported the classical MD simulations-based results of TRK-V [38]. Using the newly optimized aMD parameters, the previously reported TRK-V molecule was elucidated again to obtain the complete canonical ensemble and compare with short classical MD conducted earlier. TRK-V was previously identified as a peptaibol produced by *T. koningiopsis* along with two other 11-residue sequences, Trikoningins KB I and KB II [62]. Another study by our group identified novel peptaibols which were named as “Koningiopsins” with TRK-V as the closest sequence [38]. TRK-V, positionally isomeric with sequences Pept-Vb, -VIb, and -VII of *T. gamsii,* is a 19-residue peptaibol with seven Aib residues in its sequence. Aib is an achiral residue, which has been shown to promote helix formation and can exist in both right- and left-handed helix regions on the Ramachandran plot. To determine the propensities of each residue for a given secondary structure type, their relative free energies were calculated which clearly describe an energetically favourable conformation (Fig. 2). The spread of dihedral angle scatter during the simulation indicates that the system underwent through all the conformations. The darkest regions indicate energetically preferable conformations.

Our previous results of Ramachandran plots of TRK-V [38] showed overall secondary structural propensity towards a left-handed helical conformation. However, upon comparing the reweighted phi-psi populations in Fig. 2 with the same plots from Fig. 1 from Marik et al. [38] (100 ns long classical MD), it becomes clear that most residues like Aib1-Ile5, Ser10, Leu11, Pro13, and Gln17-Gln18 lie in the same free energy minima. However, many others flanking the central region like Gln6-Aib9, Aib12, and Val14-Ile16 show shifts from the predominant left-handed helical regions to the right-handed helical region. It is understandable for all Aib residues as the probability of occurring in both left-handed and right-handed helical quadrants is the same due to its achiral nature. A significant shift was observed in the free energy minima of Gln6 and Val14 residues from the left-handed to the right-handed helical region.

**Fig. 3:**
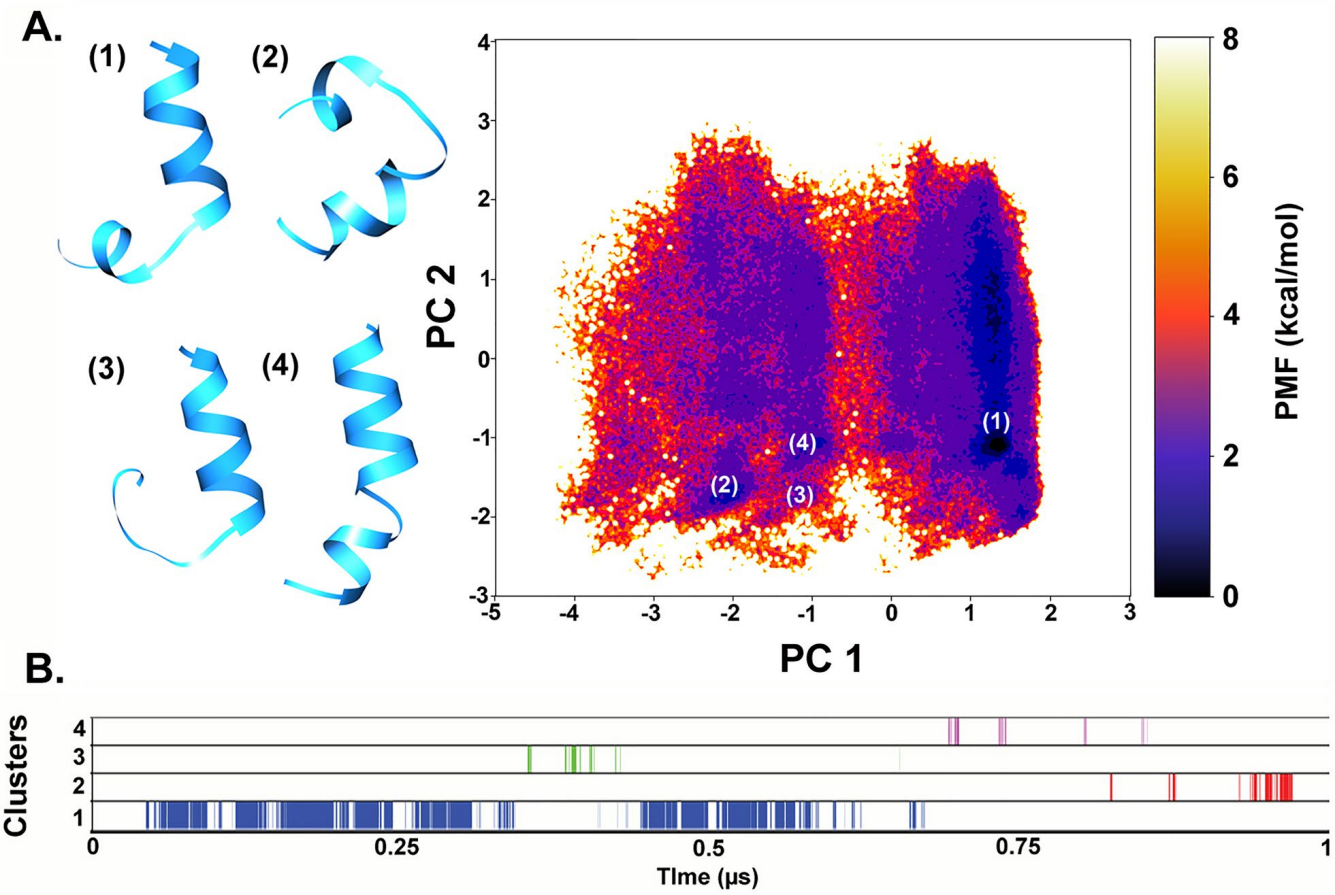
**A** Reweighted FEL of the first two principal components calculated from dihedral angles, phi-psi, for better clustering based on internal motions. The representative structures of TRK-V corresponding to various energy minima have also been provided, **B** Diagrammatic representation of cluster distribution along the simulation trajectory

Figure 3A describes the reweighted free-energy landscape of TRK-V as obtained from the first two principal components of dihedral PCA of 1 μs long aMD simulation. It is clear that the largest cluster lies at the energy minimum and the corresponding representative structure presumes a helical shape with the C-terminus showing a hinge-like bend. The loss of helical fold before the C-terminus is responsible for this hinge-like motion and is a characteristic of the Aib-Pro bond found in all long peptaibols. The next two largest clusters 2 and 3 correspond to highly bent and C-terminus loss-of-helix conformations, respectively, which probably indicates intermediate states. The 4^th^ cluster with the smallest population lies at a separate region on the free-energy landscape and corresponds to the highly helical, slightly bent conformation. This structure is most likely to be observed using experimental methods like X-ray crystallography along with the curved backbone conformations. The 1^st^ cluster appears to populate a separate region on the FEL map which can be accessed under 2 kcal mol^−1^. Its representative structure can be explained by the presence of a helix-destabilizing residue Ile16 at the C-terminus [63]. Their occurrence along the course of the simulation can be visualized in Fig. 3B. Interestingly, Ile residues at the 5^th^ and 16^th^ positions also show a strong right-handed α-helical propensity (Fig. 2). Li and Deber [64] measured the helical propensity of amino acid residues in membrane and reported that ß-branched Ile and Val can act as ‘helix-promoters’ in membrane environment but as ‘helix-destabilizers’ in aqueous environment. The effect of seemingly similar residues Leu/Ile/Val substitution on peptaibol sequences must be studied in detail. There is evidence in case of other antimicrobial peptides where a single substitution could affect interaction with lipid membranes and resulting bioactivity. For example, Aurein 2.2 and 2.3 vary only at a single position between Leu/Ile and show different bioactivities against bacterial species [65]. Studies on derivatives of another peptide, δ-lysin, showed that peptides rich in Ile may bind bilayer membranes more efficiently than Leu-rich peptides [66]. Deber and Stone [67] published a systematic study of the effect of Leu/Ile substitution on membrane-active peptides and found that peptides with all Leu/Ile placed on one side (lipophilic side) in contrast to the less hydrophobic face on the other side, showed that the Ile-rich peptide had a higher capability to insert in the membrane than the Leu-rich peptide. Similarly, Ile-rich peptides were found to be more protected from the action of proteinases than Leu peptides in the presence of lipid bilayers. Recently, Nakatani et al. [68] also reported the construction of derivatives of Trichorovin-XII with varying Ile content where all of them folded into α-helix like β-bend spiral. However, the compound found to be the most bioactive contained the highest number of Ile in its sequence. Ile analogs also showed greater ion channel activity compared to Leu analogs.

### Predicted bioactivity of peptaibiotics produced by the members of *Trichoderma* clade *Viride*

A detailed investigation of the bioactivity and function of the reported peptaibols is required to evaluate their application potential in agriculture or as therapeutics. However, their potential bioactivity can be predicted based on their sequences and the already available knowledge. First of all, the longer peptaibols (19-residues) are supposed to show higher bioactivity against bilayer membranes than their shorter counterparts. It is known that the 20-residue Alamethicin folds into a strict α-helix at the N-terminus and a 3_10_-helix at the C-terminus, and the resulting length of the folded peptide exactly spans the transmembrane width to form ion-channels. On the other hand, the 15-residue Ampullosporin A rather shows a detergent-like activity in membranes and is less active [69]. Adam et al. [70] reported on the conclusive effect of Aib-foldamers (peptaibol) length and C-terminal lipophilicity on ionophoric and antibacterial activity. The longer foldamers with a hydrophobic C-terminus were found to be more active, suggesting that the length of the peptides is an important criterion that may affect their activity. The 20-residue Paracelsins investigated in our previous work [27] folded into strict α-helices very similarly to Alamethicin, and thus, can be expected to have a similar mechanism of action.

Another major marker is the amount of Aib residues in the sequence which imparts a helical structure but not in a highly strict conformation. It can fluctuate between α-helical and 3_10_-helical conformations, thereby, expanding the length of the folded peptide to span the transmembrane region and also provide space to fit in bulky neighbouring side chains [71]. In the newly reported subgroups of Strigosellins and Dorothopsins, a total of 8 Aib residues could be found in the sequences. All peptaibol subgroups, except for Strigosellins, have 3 consecutive Aib residues from R7-R9. The presence of three consecutive helix-shape promoting Aib residues [72, 73] provides a strong helical character to the structure which seems to be crucial for most membrane-active peptides. Therefore, the presence of helix-promoting residues is also one of the main crucial features.

Lastly, the presence and position of helix-breaking residues like Gly and Pro can give us crucial clues towards helical stability. Peptide curvature or “kink” may also be a crucial feature in determining the biological activity. Cheng and Chang [74] discussed the implication of the more stable kinked form of Alamethicin than the energetically less stable linear form in voltage-gating. They proposed that linearization of the Alamethicin helices from the kinked structure upon increasing electric potential beyond a threshold may be the first event in voltage-gating mechanism. The kinks are introduced by helix-breaker residues like Pro and Gly in peptaibols. Kaduk et al. [75] showed that the substitution of Gly11 and Pro14 in Alamethicin did not affect channel formation but reduced conductance levels and significantly reduced lifetimes. Duclohier [76] in his review noted that without any applied voltage to the membrane system, Alamethicin with a higher kink angle than Trichotoxin will have a greater embedment of the N-terminus in the hydrocarbon core while the C-terminus lies flat at the bilayer interface. This greater embedment of the N-terminus, in turn, explains the higher voltage dependence of Alamethicin. Similarly, Trikoningin KA-V with a helical N-terminus, 19-residue length and a slight kink at the Aib-Pro bond also seems to be ideal for transmembrane orientation and ion channel formation. In this study, all sequences have an Aib-Pro bond which is expected to introduce this influential ‘kink’ to the folded structure. Moreover, a high bioactivity is expected from lipopeptaibols due to the presence of the fatty acid chain which is prone to strongly interact with the lipid bilayer [77]. Due to their shorter length they may not form transmembrane ion channels, but may have a different mechanism of action than peptaibols.

## Conclusions

The peptaibiotic production of *Trichoderma* clade *Viride* revealed known and new subgroups of peptaibiotics. The new subgroups of peptaibols are Strigosellins A and B with 14 and 6, Dorothopsins A-a, -b, -c, -d, -e and -f with 13, 8, 1, 3, 1 and 2, as well as, Dorothopsin B compounds with 36 completely new sequences, respectively. The already known and certain new compounds belong to the Trikoningin KA, Trichorzianin TA and Tricholongin LB subgroups with 51, 33 and 21 identified sequences, respectively. New lipopeptaibol subgroups are Lipostrigosellins and Lipohamatins with 4, and 4 completely new sequences. The other identified new and already known lipopeptaibols belong to the Lipostrigocin LSG and Trichogin GB subgroups with a total of 103 sequences. The peptaibol subgroups produced by the examined species were completely different from each other, while a few lipopeptaibols were produced by more than one species within the clade. We selected the TRK-V sequence for structure elucidation as a good representation of peptaibols produced by *T. koningii* SZMC 28387 which also has been elucidated before. Enhanced sampling of MD simulations allowed to observe that TRK-V takes a right-handed helical shape with a strong helix-disrupting effect of Ile residues when simulated in an aqueous environment. Previous research indicates towards a better membrane interactive potential of Ile-containing peptides in a hydrophobic environment. The results provide an idea about the three-dimensional structural folding of all Trikoningin-like peptaibols mentioned in this work.

## Supplementary Information

Additional file 1.

## Data Availability

All data generated or analysed during this study are included in this published article.
