# Supplementary material for: Novel peptaibiotics identified from Trichoderma clade Viride
Source: Nat Prod Bioprospect. 2025 Aug 1;15(1):48. doi: 10.1007/s13659-025-00524-9 (PMC12316631; doi:10.1007/s13659-025-00524-9)
Supplement: Supplementary file 1 — Additional file 1. [file 13659_2025_524_MOESM1_ESM.docx]

**
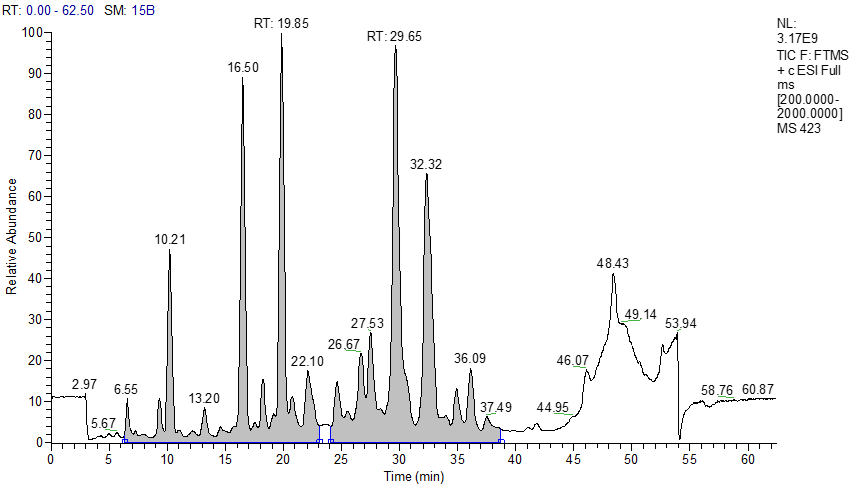
**

**A**

**Peptaibols**

**Short lipopeptaibols**

**Peptaibols**

**Short lipopeptaibols**

**B**

**
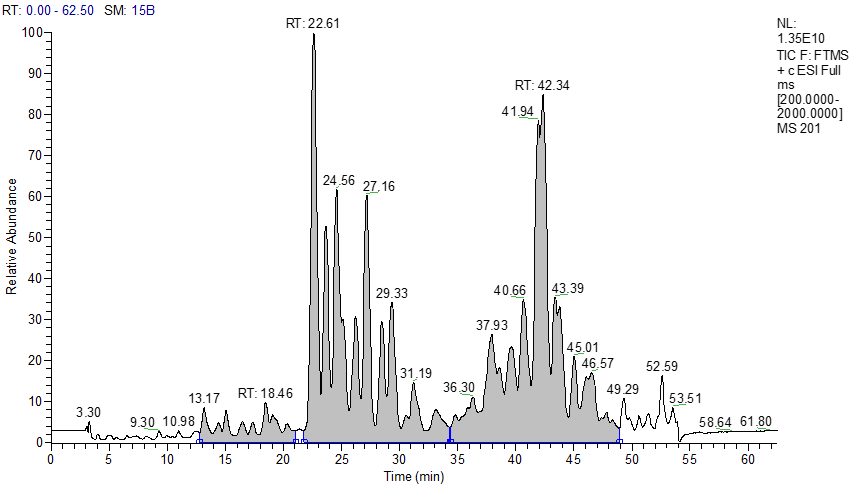
**

**Peptaibols**

**Medium lipopeptaibols**

**C**

**Short lipopeptaibols**

**
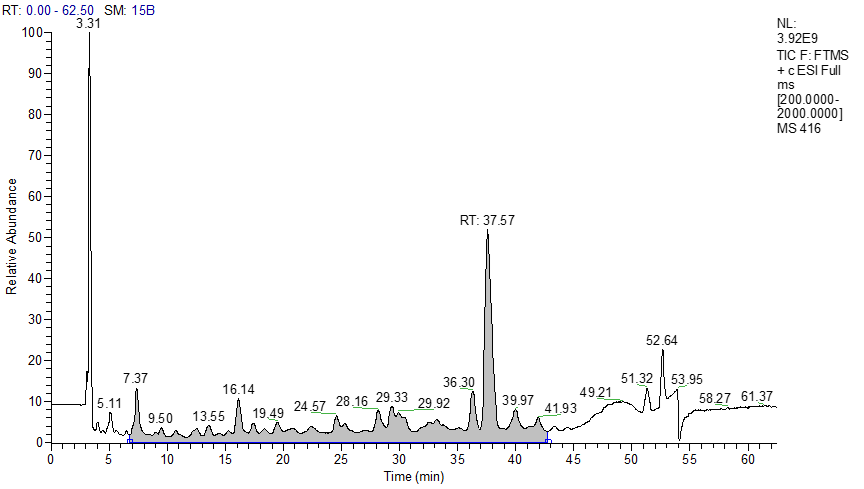
**

**Peptaibols**

**D**

**Incomplete Peptaibols**

**E**

**Peptaibols**

**Peptaibols / medium lipopeptaibols**

**F**

**Long lipopeptaibols**

**Short lipopeptaibols**

**G**

**Long lipopeptaibols**

**Peptaibols / medium lipopeptaibols**

**Supplementary figure 1** Peptaibiome of the investigated members of *Trichoderma* clade Viride shown on the total ion chromatogram of HPLC-MS. **A:** *T.* cf. *strigosellum* SZMC 28391, **B:** *T.* cf. *strigosellum* SZMC 28007, **C:** *T. koningii* SZMC 28387, **D:** *T.* cf. *dorothopsis* SZMC 28390, **E:** *T. atroviride* SZMC 28748, **F:** *T. hamatum* SZMC 28747 and **G:** *T.* cf. *dorothopsis* SZMC 28005

**Supplementary Table 1**: Diagnostic fragment ions of peptaibols detected with the MS^2^ measurement of peptaibol compounds

| \| **Peptaibol** \| \| --- \| | **y_7_** | **RT y_7_ (min)** | **R1** | **R2** | **R3** | **R4** | **R5** | **R6** | **R7** | **R8** | **R9** | **R10** | **R11** | **R12** | **R13** | **R14** | **R15** | **R16** | **R17** | **R18** | **R19** |
| --- | --- | --- | --- | --- | --- | --- | --- | --- | --- | --- | --- | --- | --- | --- | --- | --- | --- | --- | --- | --- | --- | --- |
| **Strigaibol-like I** | 740.4611 | 24.66 | 128.0727 | n.d. | 300.1563 | 385.209 | 498.2921 | 626.3451 | 711.398 | 796.4514 | 881.5051 | 968.5261 | 1067.5912 | 1152.6617 | 98.034 | 197.1283 | 282.1813 | 367.2348 | 495.2924 | 623.3447 | 722.4499 |
| **Strigaibol-like II** |  |  | n.d. | 229.1188 | 314.1716 | 399.2243 | 512.3073 | 640.3604 | 725.4132 | 796.4511 | 881.5049 | 968.5318 | 1081.6238 | 1166.6773 | 98.034 | 197.1283 | 282.1813 | 367.2348 | 495.2924 | 623.3447 | 722.4499 |
| **Strigaibol-like III** | 726.4463 | 24.9 | n.d. | 229.1191 | 314.1717 | 399.2243 | 512.3073 | 640.3604 | 725.4135 | 796.4508 | 881.5053 | 968.5322 | 1081.6244 | 1166.6779 | n.d. | 197.1282 | 282.1815 | 353.2195 | 481.2768 | 609.33 | 708.4341 |
| **Strigaibol-like IV** | 726.4462 | 25.53 | n.d. | 229.1185 | 314.1717 | 399.2243 | 512.3073 | 640.3602 | 725.4134 | 810.4664 | 895.5208 | 982.5542 | 1095.6447 | 1180.694 | n.d. | 197.1283 | 282.1814 | 353.2197 | 481.2767 | 609.3273 | 708.4328 |
| **Strigaibol-like V** | 740.4608 | 26.67 | 128.0731 | n.d. | 300.1561 | 385.2088 | 498.2917 | 626.3446 | 711.3972 | 796.4505 | 881.5043 | 968.5357 | 1081.6253 | 1166. 6771 | 98.0406 | 197.1281 | 282.1812 | 367.2347 | 495.2922 | 623.3447 | 722.4498 |
| **Strigaibol-like VI** | 740.461 | 27.53 | n.d. | 229.1178 | 314.1715 | 399.2241 | 512.3069 | 640.36 | 725.4128 | 810.466 | 895.5197 | 982.5476 | 1081.6254 | 1166.6774 | n.d. | 197.1281 | 282.1812 | 367.2346 | 495.2921 | 623.3445 | 722.45 |
| **Strigaibol-like VII** | 726.4457 | 27.75 | n.d. | 229.1176 | 313.1716 | 399.2242 | 512.3071 | 640.3602 | 725.413 | 810.4663 | 895.5197 | 982.5446 | 1081.6254 | 1166.6774 | n.d. | 197.1282 | 282.1813 | 353.2195 | 481.2769 | 609.33 | 708.4337 |
| **Strigaibol-like VIII** | 726.4463 | 28.2 | n.d. | n.d. | 284.1605 | 369.2141 | 482.2972 | 610.3515 | 695.4028 | 780.4562 | 865.5096 | 952.5378 | 1065.543 | 1150.6835 | n.d. | 197.1282 | 282.1812 | 367.2348 | 495.2926 | 623.3459 | 708.4332 |
| **Strigaibol-like IX** | 740.4618 | 28.4 | Aib | Ala | 284.1605 | 369.2141 | 482.2972 | 610.3515 | 695.4028 | 780.4562 | 865.5096 | 952.5378 | 1065.6275 | 1150.6835 | n.d. | 197.1282 | 282.1813 | 367.2349 | 495.2924 | 623.3445 | 722.4485 |
| **Strigaibol-like X** | 740.4603 | 29.66 | 128.0724 | 199.1075 | 284.1605 | 369.2141 | 482.2971 | 610.3502 | 695.4028 | 780.4564 | 865.5096 | 952.5385 | 1051.6091 | 1136.667 | n.d. | 197.1281 | 282.1811 | 367.2346 | 495.292 | 623.3446 | 722.4498 |
| **Strigaibol-like XI** |  |  | n.d. | n.d. | 300.1563 | 385.2091 | 498.2914 | 626.344 | 711.3979 | 796.451 | 881.5042 | 968.5307 | 1081.6266 | 1166.6775 | n.d. | 197.1281 | 282.1811 | 367.2346 | 495.292 | 623.3446 | 722.4498 |
| **Strigaibol-like XII** |  |  | n.d. | 229.1175 | 314.1714 | 399.2238 | 512.3066 | 640.3598 | 725.4124 | 810.4655 | 895.5192 | 982.5494 | 1095.642 | 1180.6922 | n.d. | 197.1281 | 282.1811 | 367.2346 | 495.292 | 623.3446 | 722.4498 |
| **Strigaibol-like XIII** | 740.464 | 30.52 | n.d. | 229.1191 | 314.1714 | 399.2242 | 512.3071 | 640.3602 | 725.4131 | 810.4662 | 895.52 | 982.5568 | 1081.6261 | 1166.6778 | n.d. | 197.1282 | 282.1813 | 367.2349 | 495.2924 | 623.3445 | 722.45 |
| **Strigaibol-like XIV** | 740.4607 | 32.31 | 128.0727 | 199.1071 | 284.1604 | 369.2138 | 482.2968 | 610.3497 | 695.4022 | 780.4557 | 865.5092 | 952.5385 | 1065.6263 | 1150.682 | n.d. | 197.1281 | 282.1812 | 367.2346 | 495.2921 | 623.3445 | 722.4496 |
| **Strigaibol-like XV** |  |  | n.d. | 229.1186 | 314.1716 | 399.2242 | 512.3071 | 640.3604 | 725.4131 | 810.4663 | 895.5198 | 966.5607 | 1079.7884 | 1164.6973 | n.d. | 197.1281 | 282.1812 | 367.2346 | 495.2921 | 623.3445 | 722.4496 |
| **Strigaibol-like XVI** |  |  | n.d. | 229.1183 | 314.1716 | 399.2242 | 512.3071 | 640.3605 | 725.4132 | 810.4666 | 895.52 | 982.5499 | 1095.6434 | 1180.6927 | n.d. | 197.1281 | 282.1812 | 367.2346 | 495.2921 | 623.3445 | 722.4496 |
| **Strigaibol-like XVII** | 740.4624 | 33.98 | 128.0725 | 199.1081 | 284.1606 | 369.2142 | 482.2975 | 610.3503 | 695.4032 | 780.4566 | 865.5106 | 952.551 | 1051.6145 | 1136.6688 | n.d. | 197.1283 | 282.1815 | 367.235 | 495.2926 | 623.3455 | 722.4506 |
| **Strigaibol-like XVIII** | 740.4615 | 34.86 | 128.0719 | 199.1082 | 284.1607 | 369.2142 | 482.2974 | 610.3502 | 695.403 | 780.4568 | 865.5099 | 936.5494 | 1049.6347 | 1134.6884 | 98.0417 | 197.1283 | 282.1814 | 367.235 | 495.2925 | 623.345 | 722.4498 |
| **Strigaibol-like XIX** |  |  | n.d. | 213.1233 | 298.1769 | 383.2298 | 496.313 | 624.3563 | 709.4188 | 794.4721 | 879.5258 | 966.5636 | 1079.6331 | 1164.6988 | 98.0417 | 197.1283 | 282.1814 | 367.235 | 495.2925 | 623.345 | 722.4498 |
| **Strigaibol-like XX** | 740.4613 | 36.1 | 128.0733 | 199.1074 | 284.1605 | 369.214 | 482.287 | 610.3502 | 695.4026 | 780.4562 | 865.5096 | 952.535 | 1065.6271 | 1150.6829 | n.d. | 197.1282 | 282.1813 | 367.2348 | 495.2924 | 623.3449 | 722.4496 |
| **Strigaibol-like XXI** | 740.4619 | 37.55 | 128.2583 | 227.1388 | 312.1926 | 397.2453 | 510.3282 | 638.3813 | 723.4343 | 808.4878 | 893.5401 | 980.5854 | 1093.6562 | 1178.715 | n.d. | 197.1282 | 282.1814 | 367.235 | 495.2924 | 623.3453 | 722.4511 |
| **Peptaibol** | **y_7_** | **RT y_7_ (min)** | **R1** | **R2** | **R3** | **R4** | **R5** | **R6** | **R7** | **R8** | **R9** | **R10** | **R11** | **R12** | **R13** | **R14** | **R15** | **R16** | **R17** | **R18** | **R19** |
| **Trikoningin KA-like I** | 754.4777 | 31.31 | 128.072 | 185.0928 | 256.1287 | 341.183 | 440.2503 | 568.3047 | 653.3549 | 738.408 | 823.4619 | 910.495 | 1023.7155 | 1108.6353 | n.d. | 197.1282 | 282.1813 | 381.2504 | 509.3076 | 637.3632 | 736.4617 |
| **Trikoningin KA-like II** | 755.4613 | 31.88 | n.d. | 185.0923 | 256.1287 | 341.183 | 440.2504 | 568.3057 | 653.3551 | 738.4081 | 823.4615 | 910.4952 | 1023.5932 | 1108.6365 | n.d. | 197.1282 | 282.1814 | 381.2503 | 510.292 | 638.3436 | 737.4516 |
| **Trikoningin KA-like III** |  |  | n.d. | 185.0933 | 256.1285 | 341.1832 | 454.2663 | 582.3217 | 667.3709 | 752.4255 | 837.4815 | 924.5043 | 1037.5941 | 1122.6523 | n.d. | 197.1282 | 282.1814 | 381.2503 | 510.292 | 638.3436 | 737.4516 |
| **Trikoningin KA-like IV** | 768.4936 | 32.89 | 128.0723 | 185.0931 | 256.1284 | 341.183 | 440.2502 | 568.3052 | 653.3548 | 738.4079 | 823.4616 | 910.496 | 1023.5896 | 1108.6358 | 98.0421 | 197.1282 | 282.1813 | 395.2658 | 523.3233 | 651.3763 | 750.482 |
| **Trikoningin KA-like V** |  |  | n.d. | n.d. | 272.1238 | 357.178 | 470.2606 | 598.3171 | 683.3668 | 768.4167 | 853.4727 | n.d. | 1053.7106 | 1138.7915 | 98.0421 | 197.1282 | 282.1813 | 395.2658 | 523.3233 | 651.3763 | 750.482 |
| **Trikoningin KA-like VI** | 769.4769 | 33.42 | 128.0718 | 185.093 | 256.1287 | 341.1831 | 440.2504 | 568.3054 | 653.3551 | 738.4083 | 823.4617 | 910.4961 | 2023.5818 | 1108.5358 | n.d. | 197.1281 | 282.1812 | 395.2657 | 524.3075 | 652.361 | 751.4675 |
| **Trikoningin KA-like VII** |  |  | 128.0714 | 185.0936 | 272.1234 | 357.1779 | 470.2608 | 598.3157 | 683.3666 | 768.4197 | 853.5633 | 940.5079 | 1053.7089 | 1138.7913 | Pro | 197.1281 | 282.1812 | 395.2657 | 524.3075 | 652.361 | 751.4675 |
| **Trikoningin KA-like VIII** | 754.4784 | 34.01 | n.d. | n.d. | 242.1131 | 327.1679 | 440.2503 | 568.3047 | 653.3553 | 738.4081 | 823.462 | 894.4943 | 1007.5431 | 1092.1193 | 98.0231 | 197.1282 | 282.1812 | 381.2504 | 509.3074 | 637.3603 | 736.4653 |
| **Trikoningin KA-like IX** |  |  | 128.0719 | 185.0927 | 256.1284 | 341.1829 | 440.2503 | 568.3052 | 653.3548 | 738.4079 | 823.4616 | 910.4952 | 1023.5833 | 1108.6358 | 98.0231 | 197.1282 | 282.1812 | 381.2504 | 509.3074 | 637.3603 | 736.4653 |
| **Trikoningin KA-like X** |  |  | 128.073 | 185.0933 | 256.1285 | 341.1832 | 454.2663 | 582.3217 | 667.3709 | 752.4255 | 837.4815 | 924.5043 | 1037.6716 | 1122.6512 | 98.0231 | 197.1282 | 282.1812 | 381.2504 | 509.3074 | 637.3603 | 736.4653 |
| **Trikoningin KA-like XI** | 768.4936 | 34.76 | 128.0714 | 185.0916 | 256.1285 | 341.1829 | 440.2502 | 568.3052 | 653.3549 | 738.408 | 823.4614 | 910.495 | 1023.5838 | 1108.6359 | n.d. | 197.1282 | 282.1812 | 395.2657 | 523.3231 | 651.3758 | 750.4818 |
| **Trikoningin KA-like XII** |  |  | 128.0725 | 185.0916 | 272.1234 | 357.1779 | 470.2607 | 598.316 | 683.3663 | 768.4193 | 853.4732 | 940.5082 | 1053.7109 | 1138.6454 | n.d. | 197.1282 | 282.1812 | 395.2657 | 523.3231 | 651.3758 | 750.4818 |
| **Trikoningin KA-like XIII** | 754.4782 | 34.97 | 128.0729 | 185.0916 | 256.1286 | 341.183 | 440.2503 | 568.3052 | 653.355 | 738.408 | 823.4618 | 910.4982 | 1023.5815 | 1108.6358 | n.d. | 197.1282 | 282.1814 | 381.2506 | 509.3075 | 637.361 | 736.4677 |
| **Trikoningin KA-like XIV** |  |  | 128.0729 | 185.0913 | 256.1287 | 341.1831 | 454.2663 | 582.3214 | 667.3707 | 752.4254 | 837.4785 | 924.5179 | 1037.5937 | 1122.6515 | n.d. | 197.1282 | 282.1814 | 381.2506 | 509.3075 | 637.361 | 736.4677 |
| **Trikoningin KA-like XV** | 769.4771 | 35.36 | 128.0725 | 185.0907 | 256.1287 | 341.1831 | 440.2506 | 568.3057 | 653.3553 | 738.4084 | 823.4622 | 910.4953 | 1023.5818 | 1108.6357 | n.d. | 197.1281 | 282.1812 | 395.2657 | 524.3075 | 652.361 | 751.4672 |
| **Trikoningin KA-like XVI** |  |  | 128.0724 | 185.0915 | 272.1236 | 357.1779 | 470.2606 | 698.316 | 683.3663 | 768.4196 | 853.4729 | 940.509 | 1053.7108 | 1138.6464 | n.d. | 197.1281 | 282.1812 | 395.2657 | 524.3075 | 652.361 | 751.4672 |
| **Trikoningin KA-like XVII** | 755.4604 | 35.5 | 128.0718 | 185.091 | 256.1286 | 341.183 | 454.2662 | 582.3215 | 667.3706 | 752.4252 | 837.479 | 924.5071 | 1037.694 | 1122.6513 | n.d. | 197.1283 | 282.1813 | 381.2505 | 510.2921 | 639.3459 | 737.4509 |
| **Trikoningin KA-like XVIII** | 768.4935 | 35.88 | 128.0724 | 185.0914 | 256.1285 | 341.1828 | 440.2501 | 658.3049 | 653.3546 | 738.4077 | 823.4613 | 910.4954 | 1023.582 | 1108.6357 | n.d. | 197.1281 | 282.1812 | 395.2656 | 523.3229 | 651.3759 | 750.4826 |
| **Trikoningin KA-like XIX** |  |  | 128.0724 | 185.093 | 256.1284 | 341.183 | 454.2662 | 582.321 | 667.3705 | 752.4254 | 837.4788 | 924.5099 | 1037.6707 | 1122.6511 | n.d. | 197.1281 | 282.1812 | 395.2656 | 523.3229 | 651.3759 | 750.4826 |
| **Trikoningin KA-like XX** | 769.4772 | 36.34 | 128.0727 | 185.0916 | 256.1286 | 341.183 | 440.2503 | 568.3052 | 653.355 | 738.408 | 823.4618 | 910.4982 | 1023.5815 | 1108.6358 | n.d. | 197.1281 | 282.1812 | 395.2656 | 524.3071 | 652.3605 | 751.4671 |
| **Trikoningin KA-like XXI** |  |  | 128.072 | 185.0927 | 256.1285 | 341.1831 | 454.2663 | 582.3217 | 667.3709 | 752.4255 | 837.4815 | 924.5043 | 1037.6716 | 1122.6512 | n.d. | 197.1281 | 282.1812 | 395.2656 | 524.3071 | 652.3605 | 751.4671 |
| **Trikoningin KA-like XXII** | 768.4932 | 37.36 | 128.0717 | 185.0912 | 256.1285 | 341.183 | 440.2503 | 568.3051 | 653.355 | 738.4081 | 823.4614 | 894.5007 | 1007.532 | 1092.6409 | 98.3348 | 197.1282 | 282.1812 | 395.2658 | 523.3231 | 651.4757 | 750.4817 |
| **Trikoningin KA-like XXIII** |  |  | 128.0721 | 185.0915 | 256.1286 | 341.1829 | 440.2504 | 568.3051 | 653.355 | 738.408 | 823.4614 | 910.4969 | 1023.582 | 1108.6357 | 98.3348 | 197.1282 | 282.1812 | 395.2658 | 523.3231 | 651.4757 | 750.4817 |
| **Trikoningin KA-like XXIV** | 754.4779 | 37.46 | 128.0717 | 185.0912 | 256.1285 | 341.183 | 440.2503 | 568.3051 | 653.355 | 738.4081 | 823.4614 | 894.5007 | 1007.5346 | 1092.6408 | n.d. | 197.1282 | 282.1812 | 381.2503 | 509.3074 | 637.3607 | 736.4655 |
| **Trikoningin KA-like XXV** |  |  | 128.0727 | 185.0916 | 256.1286 | 341.183 | 440.2504 | 568.305 | 653.355 | 738.4081 | 823.4618 | 910.4966 | 1023.5794 | 1108.6357 | n.d. | 197.1282 | 282.1812 | 381.2503 | 509.3074 | 637.3607 | 736.4655 |
| **Trikoningin KA-like XXVI** |  |  | 128.0726 | 185.0912 | 256.1284 | 341.1827 | 454.2657 | 582.3206 | 667.3698 | 752.4243 | 837.4777 | 924.5095 | 1037.5947 | 1122.6512 | n.d. | 197.1282 | 282.1812 | 381.2503 | 509.3074 | 637.3607 | 736.4655 |
| **Trikoningin KA-like XXVII** | 769.4752 | 37.94 | 128.072 | 185.0913 | 256.1286 | 341.183 | 440.2504 | 568.3051 | 653.3552 | 738.4084 | 823.4617 | 894.4983 | 1007.6183 | 1092.6408 | 98.0418 | 197.1282 | 282.1812 | 395.2657 | 524.3077 | 652.3618 | 751.4682 |
| **Trikoningin KA-like XXVIII** |  |  | 128.0723 | 185.0919 | 256.1286 | 341.1831 | 440.2504 | 568.3054 | 653.355 | 738.4081 | 823.4619 | 910.4921 | 1023.6784 | 1108.1107 | 98.0418 | 197.1282 | 282.1812 | 395.2657 | 524.3077 | 652.3618 | 751.4682 |
| **Trikoningin KA-like XXIX** | 755.4615 | 38.08 | 128.0716 | 185.091 | 256.1286 | 341.1831 | 440.2505 | 558.3051 | 653.3553 | 738.4084 | 823.4619 | 894.501 | 1007.5872 | 1092.6409 | n.d. | 197.1282 | 282.1814 | 381.2503 | 510.292 | 638.3446 | 737.4501 |
| **Trikoningin KA-like XXX** |  |  | 128.0723 | 185.0919 | 256.1286 | 341.1831 | 440.2504 | 568.3054 | 653.355 | 738.4081 | 823.4619 | 910.4921 | 1023.6777 | 1108.6356 | n.d. | 197.1282 | 282.1814 | 381.2503 | 510.292 | 638.3446 | 737.4501 |
| **Trikoningin KA-like XXXI** |  |  | 128.0725 | 185.0913 | 256.1287 | 341.1829 | 454.2663 | 582.3214 | 667.3707 | 752.4254 | 837.4781 | 924.5085 | 1037.5937 | 1122.6515 | n.d. | 197.1282 | 282.1814 | 381.2503 | 510.292 | 638.3446 | 737.4501 |
| **Trikoningin KA-like XXXII** | 754.478 | 38.94 | 128.0715 | 185.0912 | 256.1287 | 341.1831 | 440.2505 | 568.3049 | 653.3553 | 738.4083 | 823.4619 | 894.4986 | 1007.5439 | 1092.6411 | n.d. | 197.1282 | 282.1814 | 381.2505 | 509.3076 | 637.3605 | 736.466 |
| \| **Trikoningin KA-like XXXIII** \| \| --- \| |  |  | 128.0727 | 185.0915 | 256.1285 | 341.183 | 454.2552 | 558.3051 | 667.3706 | 752.4253 | 837.4786 | 908.5156 | 1021.6813 | 1106.6669 | n.d. | 197.1282 | 282.1814 | 381.2505 | 509.3076 | 637.3605 | 736.466 |
| **Trikoningin KA-like XXXIV** |  |  | 128.071 | 185.0933 | 256.1285 | 341.1832 | 454.2663 | 582.3217 | 667.3709 | 752.4255 | 837.4815 | 924.5043 | 1037.6716 | 1122.6517 | n.d. | 197.1282 | 282.1814 | 381.2505 | 509.3076 | 637.3605 | 736.466 |
| **Trikoningin KA-like XXXV** | 768.4936 | 39.41 | 128.0725 | 185.0913 | 256.1285 | 341.1828 | 454.2658 | 582.3217 | 667.3709 | 752.4255 | 837.4815 | 924.5043 | 1037.5975 | 1122.6517 | n.d. | 197.1282 | 282.1814 | 395.2658 | 523.3231 | 651.376 | 750.4819 |
| **Trikoningin KA-like XXXVI** | 755.4614 | 39.46 | 128.0721 | 185.093 | 256.1288 | 341.1831 | 440.2505 | 568.3071 | 653.3552 | 738.4081 | 823.4615 | 894.5024 | 1007.5511 | 1092.6413 | 98.033 | 197.1283 | 282.1814 | 381.2505 | 510.2919 | 638.3459 | 737.4523 |
| **Trikoningin KA-like XXXVII** |  |  | 128.0723 | 185.0911 | 256.1286 | 341.183 | 454.2661 | 582.3213 | 667.3705 | 752.4252 | 837.4785 | 908.5154 | 1021.6011 | 1106.6596 | 98.033 | 197.1283 | 282.1814 | 381.2505 | 510.2919 | 638.3459 | 737.4523 |
| **Trikoningin KA-like XXXVIII** |  |  | 128.0725 | 185.0913 | 256.1285 | 341.1828 | 454.2658 | 582.3208 | 667.3699 | 752.4246 | 837.478 | 924.5135 | 1037.5974 | 1122.6517 | 98.033 | 197.1283 | 282.1814 | 381.2505 | 510.2919 | 638.3459 | 737.4523 |
| **Trikoningin KA-like XXXIX** | 769.4773 | 39.85 | 128.0727 | 185.0915 | 256.1285 | 341.1827 | 454.2658 | 582.3206 | 667.3698 | 752.4243 | 837.4777 | 924.5095 | 1037.595 | 1122.6516 | n.d. | 197.1283 | 282.1814 | 395.2657 | 524.3073 | 652.3611 | 751.4678 |
| **Trikoningin KA-like XL** | 768.4935 | 40.59 | 128.0726 | 185.0914 | 256.1285 | 341.1828 | 454.2659 | 582.3208 | 667.3699 | 752.4246 | 837.4778 | 924.5098 | 1037.5984 | 1122.6516 | n.d. | 197.1282 | 282.1813 | 395.2658 | 523.3232 | 651.376 | 750.4821 |
| **Trikoningin KA-like XLI** | 769.4777 | 40.9 | 128.1725 | 185.0916 | 256.1284 | 341.1828 | 454.2658 | 582.3209 | 667.3699 | 752.4244 | 837.4778 | 924.5112 | 1037.5978 | 1122.6517 | n.d. | 197.1282 | 282.1813 | 395.2658 | 524.3082 | 652.3625 | 751.4687 |
| **Trikoningin KA-like XLII** | 768.4933 | 41.88 | 128.0725 | 185.0914 | 256.1285 | 341.183 | 454.2661 | 582.3213 | 667.3704 | 752.425 | 837.4784 | 908.5136 | 1021.6212 | 1106.6568 | n.d. | 197.1282 | 282.1813 | 395.2658 | 523.3232 | 651.376 | 750.4819 |
| **Trikoningin KA-like XLIII** |  |  | 128.0724 | 185.0914 | 256.1285 | 341.1828 | 454.2659 | 582.3209 | 667.47 | 752.4246 | 837.478 | 924.5114 | 1037.5988 | 1122.6513 | n.d. | 197.1282 | 282.1813 | 395.2658 | 523.3232 | 651.376 | 750.4819 |
| **Trikoningin KA-like XLIV** | 769.4781 | 42.36 | 128.0728 | 185.0918 | 256.1287 | 341.183 | 454.2661 | 582.3212 | 667.3705 | 752.4251 | 837.4785 | 908.5185 | 1021.593 | 1106.6569 | n.d. | 197.1282 | 282.1813 | 395.2658 | 524.3074 | 652.3608 | 751.4677 |
| **Trikoningin KA-like XLV** |  |  | 128.0726 | 185.0914 | 256.1284 | 341.1829 | 454.266 | 582.3209 | 667.3701 | 752.4247 | 837.4781 | 924.5117 | 1037.599 | 1122.6514 | n.d. | 197.1282 | 282.1813 | 395.2658 | 524.3074 | 652.3608 | 751.4677 |
| **Trikoningin KA-like XLVI** | 768.4938 | 43.33 | 128.0724 | 185.0913 | 256.1285 | 341.1927 | 454.2658 | 582.3207 | 667.3698 | 752.4244 | 837.4778 | 908.5149 | 1021.5497 | 1106.6566 | n.d. | 197.1282 | 282.1812 | 395.2657 | 523.3229 | 651.3762 | 750.4819 |
| **Trikoningin KA-like XLVII** |  |  | 128.0716 | 199.1077 | 270.1445 | 355.1987 | 468.2814 | 596.337 | 681.3874 | 766.441 | 851.4935 | 938.5308 | 1051.608 | 1136.6566 | n.d. | 197.1282 | 282.1812 | 395.2657 | 523.3229 | 651.3762 | 750.4819 |
| **Trikoningin KA-like XLVIII** | 769.4774 | 43.83 | 128.0722 | 185.0913 | 256.1284 | 341.1828 | 454.2659 | 582.3209 | 667.37 | 752.4246 | 837.478 | 908.515 | 1021.5933 | 1106.6566 | 98.033 | 197.1282 | 282.1812 | 395.2657 | 524.3074 | 652.3611 | 751.4683 |
| **Trikoningin KA-like XLIX** |  |  | 128.0722 | 199.1062 | 270.1445 | 355.1987 | 468.2814 | 596.337 | 681.3874 | 766.4409 | 851.4949 | 938.5244 | 1051.6083 | 1136.6667 | 98.033 | 197.1282 | 282.1812 | 395.2657 | 524.3074 | 652.3611 | 751.4683 |
| **Trikoningin KA-like L** | 768.4936 | 46.05 | 128.0724 | 185.092 | 256.1285 | 341.183 | 454.2662 | 582.3214 | 667.3706 | 752.4252 | 837.4788 | 924.5181 | 1037.515 | 1122.6511 | n.d. | 197.1282 | 282.1812 | 395.1265 | 523.3231 | 651.3759 | 750.4825 |
| **Trikoningin KA-like LI** |  |  | 128.0725 | 199.1071 | 270.1443 | 355.1985 | 468.2811 | 596.3365 | 681.3868 | 766.4402 | 851.4936 | 938.5272 | 1051.6091 | 1136 | n.d. | 197.1282 | 282.1812 | 395.1265 | 523.3231 | 651.3759 | 750.4825 |
| **Trikoningin KA-like LII** | 769.4771 | 46.43 | 128.0717 | 185.0919 | 256.1288 | 341.183 | 454.2662 | 582.3214 | 667.3705 | 752.4252 | 837.4785 | 924.5143 | 1037.6699 | 1122.6511 | n.d. | 197.1282 | 282.1812 | 395.2657 | 524.3073 | 652.3608 | 751.4667 |
| **Trikoningin KA-like LIII** |  |  | 128.0725 | 199.1071 | 270.1442 | 355.1985 | 468.2812 | 596.3365 | 681.3868 | 766.4402 | 851.4935 | 938.5285 | 1051.696 | 1136.6672 | n.d. | 197.1282 | 282.1812 | 395.2657 | 524.3073 | 652.3608 | 751.4667 |
| **Trikoningin KA-like LIV** | 768.494 | 47.39 | 128.0727 | 199.1069 | 270.1443 | 355.1985 | 468.2813 | 596.3365 | 681.3871 | 766.4405 | 851.4938 | 922.5285 | 1035.6191 | 1120.6728 | n.d. | 197.1281 | 282.1812 | 395.2657 | 523.3229 | 651.3757 | 750.4814 |
| **Trikoningin KA-like LV** |  |  | 128.0718 | 199.1085 | 270.1443 | 355.1987 | 468.2817 | 596.3375 | 681.3876 | 766.4412 | 851.4939 | 938.5255 | 1051.696 | 1136.6675 | n.d. | 197.1281 | 282.1812 | 395.2657 | 523.3229 | 651.3757 | 750.4814 |
| **Trikoningin KA-like LVI** | 769.4764 | 47.77 | 128.0727 | 199.1069 | 270.1442 | 355.1985 | 468.2811 | 596.03367 | 681.3868 | 766.4403 | 851.4937 | 922.5285 | 1035.6198 | 1120.677 | 98.0405 | 197.1281 | 282.1812 | 395.2656 | 524.3075 | 652.3605 | 751.4678 |
| **Trikoningin KA-like LVII** |  |  | 128.0719 | 199.1082 | 270.1452 | 355.1984 | 468.2816 | 596.3378 | 681.3872 | 766.3302 | 851.493 | 938.5258 | 1051.6947 | 1136.6674 | 98.0405 | 197.1281 | 282.1812 | 395.2656 | 524.3075 | 652.3605 | 751.4678 |
| **Peptaibol** | **y_7_** | **RT y_7_ (min)** | **R1** | **R2** | **R3** | **R4** | **R5** | **R6** | **R7** | **R8** | **R9** | **R10** | **R11** | **R12** | **R13** | **R14** | **R15** | **R16** | **R17** | **R18** | **R19** |
| **Dorothopsin A-a I** | 670.37 | 12.56 | n.d. | 199.1083 | 284.1607 | 369.2143 | 482.2974 | 610.35 | 695.4032 | 780.4569 | 865.5085 | 952.5378 | 1065.6313 | 1150.6819 | n.d. | 197.1283 | 282.1814 | 395.2661 | 524.3068 | n.d. | 652.3592 |
| **Dorothopsin A-a II** | 670.3707 | 13.59 | 128.0721 | 199.1072 | 284.1606 | 369.2141 | 482.2973 | 610.3507 | 695.403 | 780.4564 | 865.5097 | 952.5378 | 1065.6292 | 1150.6822 | n.d. | 197.1283 | 282.1815 | 395.2661 | 524.3068 | n.d. | 652.3516 |
| **Dorothopsin A-a III** | 740.4619 | 32.28 | 128.4079 | 199.108 | 284.1606 | 369.2143 | 482.2975 | 610.3499 | 695.4033 | 780.4566 | 865.5104 | 952.5399 | n.d. | 1150.6833 | n.d. | 197.1284 | 282.1815 | 381.2509 | 509.3075 | 637.3615 | 722.4618 |
| **Dorothopsin A-a IV** | 740.4618 | 33.3 | n.d. | 199.1087 | 284.1606 | 369.2149 | 482.2974 | 610.3501 | 695.4032 | 780.4565 | 865.5105 | 952.5437 | 1065.6316 | 1150.6833 | n.d. | 197.1284 | 282.1815 | 381.2506 | 509.3081 | 637.3604 | 722.45 |
| **Dorothopsin A-a V** | 741.4455 | 33.76 | n.d. | 199.1087 | 284.1606 | 369.2142 | 482.2974 | 610.3501 | 695.4032 | 780.4565 | 865.5105 | 952.5437 | 1065.6316 | 1150.6842 | n.d. | 197.1283 | 282.1815 | 381.2508 | 510.1927 | 638.3459 | 723.4368 |
| **Dorothopsin A-a VI** | 754.478 | 36.28 | 128.0728 | 199.1079 | 284.1607 | 369.2142 | 482.2973 | 610.3506 | 695.403 | 780.4566 | 865.5099 | 952.5288 | 1065.629 | 1150.6829 | n.d. | 197.1283 | 282.1814 | 395.266 | 523.3233 | 651.3765 | 736.4656 |
| **Dorothopsin A-a VII** | 754.4775 | 37.53 | 128.0729 | 199.1072 | 284.1605 | 369.2139 | 482.287 | 610.35 | 695.4023 | 780.4559 | 865.5095 | 952.5389 | 1065.6274 | 1150.6823 | n.d. | 197.1283 | 282.1814 | 395.2659 | 523.3233 | 651.376 | 736.4656 |
| **Dorothopsin A-a VIII** | 755.4619 | 37.98 | 128.0727 | 199.107 | 284.1605 | 369.214 | 482.2971 | 610.35 | 695.4026 | 780.4561 | 865.5095 | 952.5402 | 1065.6285 | 1150.6833 | n.d. | 197.1283 | 282.1815 | 395.2659 | 524.311 | 652.3664 | 737.4559 |
| **Dorothopsin A-a IX** | 741.52 | 39.99 | 142.0877 | 213.1228 | 298.1771 | 383.2299 | 496.3131 | 624.3658 | 709.419 | 794.4721 | 879.5254 | 966.5574 | 1079.6531 | 1164.6994 | n.d. | 197.1283 | 282.1816 | 381.2508 | 510.1923 | 638.3456 | 723.4355 |
| **Dorothopsin A-a X** | 754.4787 | 40 | 142.0886 | 213.1223 | 298.177 | 383.2297 | 496.3128 | 624.3652 | 709.4186 | 794.4718 | 827.5256 | 966.5545 | 1079.6514 | 1164.6996 | 98.706 | 197.1283 | 282.1815 | 395.2661 | 523.3237 | 651.3762 | 736.4655 |
| **Dorothopsin A-a XI** | 755.4648 | 40.4 | 142.0886 | 213.1223 | 298.177 | 383.2297 | 496.3128 | 624.3652 | 709.4186 | 794.4718 | 879.5256 | 966.5545 | 1079.656 | 1164.6996 | n.d. | 197.1284 | 282.1815 | 395.2661 | 524.3077 | 652.3611 | 737.4518 |
| **Dorothopsin A-a XII** | 768.494 | 41.9 | 128.0724 | 199.1082 | 284.1607 | 369.2143 | 482.2975 | 610.351 | 695.4033 | 780.4568 | 865.5106 | 952.5388 | 1065.6303 | 1150.6834 | n.d. | 197.1283 | 282.1815 | 395.266 | 523.323 | 651.3766 | 750.4817 |
| **Dorothopsin A-a XIII** | 768.4942 | 42.26 | 142.0878 | 213.1229 | 298.177 | 383.2298 | 496.3129 | 624.3654 | 709.4188 | 794.472 | 879.5264 | 966.5505 | 1079.6606 | 1164.6997 | n.d. | 197.1284 | 282.1815 | 395.2661 | 523.3232 | 651.3768 | 750.4796 |
| **Peptaibol** | **y_7_** | **RT y_7_ (min)** | **R1** | **R2** | **R3** | **R4** | **R5** | **R6** | **R7** | **R8** | - | **R9** | **R10** | **R11** | **R12** | **R13** | **R14** | **R15** | **R16** | **R17** | **R18** |
| **Dorothopsin A-b I** | 670.37 | 7.84 | 128.0728 | 199.1085 | 284.1605 | 369.2142 | 482.2974 | 610.3506 | 695.403 | 780.4574 | - | 867.4968 | n.d. | 1065.6301 | n.d. | 197.1283 | 282.1814 | 395.2661 | 524.3068 | n.d. | 652.3592 |
| **Dorothopsin A-b II** | 670.3712 | 8.98 | 128.072 | 199.1079 | 284.1606 | 369.2141 | 482.2973 | 610.35 | 695.403 | 780.4567 | - | 867.4906 | n.d. | 1065.6301 | n.d. | 197.1283 | 282.1815 | 395.2661 | 524.3068 | n.d. | 652.3516 |
| **Dorothopsin A-b III** | 740.4608 | 24.46 | 128.0725 | 199.108 | 284.1606 | 369.2141 | 482.2973 | 610.351 | 695.4031 | 780.4557 | - | 867.4849 | n.d. | 1065.6301 | n.d. | 197.1282 | 282.1815 | 381.2509 | 509.3075 | 637.3615 | 722.4518 |
| **Dorothopsin A-b IV** | 741.4459 | 25.72 | 128.0731 | 199.1072 | 284.1606 | 369.2142 | 482.2975 | 610.3505 | 695.4031 | 780.4568 | - | 867.4914 | n.d. | 1065.6301 | n.d. | 197.1283 | 282.1815 | 381.2508 | 510.2927 | 638.3459 | 723.4368 |
| **Dorothopsin A-b V** | 740.4614 | 29.68 | 128.0724 | 199.1068 | 284.1606 | 369.2141 | 482.2973 | 610.3502 | 695.403 | 780.4571 | - | 867.491 | 980.5982 | 1065.6301 | n.d. | 197.1282 | 282.1815 | 381.2509 | 509.3075 | 637.3615 | 722.4518 |
| **Dorothopsin A-b VI** | 741.45 | 30.61 | 128.0718 | 199.1079 | 284.1606 | 369.2141 | 482.2974 | 610.3503 | 695.4032 | 780.456 | - | 867.4946 | n.d. | 1065.6308 | n.d. | 197.1283 | 282.1815 | 381.2508 | 510.2927 | 638.3459 | 723.4368 |
| **Dorothopsin A-b VII** | 754.4779 | 29.29 | 128.0731 | 199.1072 | 284.1606 | 369.2142 | 482.2975 | 610.3505 | 695.4031 | 780.4568 | - | 867.4785 | 980.5959 | 1065.6299 | n.d. | 197.1282 | 282.1814 | 395.2658 | 523.3243 | 651.3758 | 736.4656 |
| **Dorothopsin A-b VIII** | 755.4622 | 30.44 | 128.0722 | 199.107 | 284.1606 | 369.2141 | 482.2973 | 610.35 | 695.403 | 780.4564 | - | 867.4888 | 980.61 | 1065.6308 | n.d. | 197.1282 | 282.1814 | 395.266 | 523.3072 | 652.3608 | 737.45 |
| **Peptaibol** | **y_7_** | **RT y_7_ (min)** | **R1** | **R2** | **R3** | **R4** | **R5** | **R6** | **R7** | **R8** | - | **R9** | **R10** | **R11** | **R12** | **R13** | **R14** | **R15** | - | **R16** | **R17** |
| **Dorothopsin A-c I** | 541.3342 | 9.5 | 128.072 | 199.1079 | 284.1606 | 369.2141 | 482.2973 | 610.35 | 695.403 | 780.4567 | - | 867.4906 | 980.575 | 1065.6296 | n.d. | 197.1282 | 282.1813 | 395.266 | - | n.d. | 523.3228 |
| **Peptaibol** | **y_7_** | **RT y_7_ (min)** | **R1** | **R2** | **R3** | **R4** | **R5** | **R6** | **R7** | **R8** | **R9** | **R10** | **R11** | **R12** | **R13** | **R14** | **R15** | **R16** | - | **R17** | **R18** |
| **Dorothopsin A-d I** | 541.3337 | 16.09 | 128.0726 | 199.107 | 284.1607 | 369.2142 | 482.2973 | 610.3505 | 695.403 | 780.4567 | 865.5099 | 952.5438 | 1065.6286 | 1150.6814 | 98.0405 | 197.1283 | 282.1815 | 395.266 | - | n.d. | 523.3181 |
| **Dorothopsin A-d II** | 541.3349 | 18.36 | 128.0726 | 199.1091 | 284.1604 | 369.2142 | 482.2974 | 610.3507 | 695.4033 | 780.4567 | 865.5095 | n.d. | 1065.6307 | 1150.6814 | n.d. | 197.1282 | 282.1815 | 395.2663 | - | n.d. | 523.3038 |
| **Dorothopsin A-d III** | 541.3349 |  | n.d. | 213.1234 | 298.177 | 383.2301 | 496.3131 | 624.366 | 709.4192 | 794.4723 | 879.5269 | 966.563 | n.d. | 1164.6997 | n.d. | 197.1282 | 282.1815 | 395.2663 | - | n.d. | 523.3038 |
| \| **Peptaibol** \| \| --- \| | **y_7_** | **RT y_7_ (min)** | **R1** | **R2** | **R3** | **R4** | **R5** | **R6** | **R7** | **R8** |  | **R9** | **R10** | **R11** | **R12** | **R13** | **R14** |  | - | **R15** | **R16** |
| **Dorothopsin A-e I** | 413.2868 | 10.69 | 128.072 | 199.1074 | 284.1605 | 369.2141 | 482.2971 | 610.3502 | 695.4029 | 780.4568 | - | 867.4816 | 980.5465 | 1065.6295 | Pro | 197.1283 | 282.1815 | - | - | n.d. | 395.2648 |
| **Peptaibol** | **y_7_** | **RT y_7_ (min)** | **R1** | **R2** | **R3** | **R4** | **R5** | **R6** | **R7** | **R8** | **R9** | **R10** | **R11** | **R12** | **R13** | **R14** | **R15** |  | - | **R16** | **R17** |
| **Dorothopsin A-f I** | 413.28 | 18.29 | 128.0726 | 199.1091 | 284.1604 | 369.2142 | 482.2974 | 610.3507 | 695.4033 | 780.4567 | 865.5095 | n.d. | 1065.6307 | 1150.6814 | n.d. | 197.1283 | 282.1814 | - | - | n.d. | 395.2684 |
| **Dorothopsin A-f II** | 413.2778 | 19.49 | n.d. | 199.1083 | 284.1608 | 369.2143 | 482.2974 | 610.3502 | 695.4034 | 780.457 | 865.5106 | 952.5482 | 1065.6294 | 1150.6835 | n.d. | 197.1283 | 282.1815 | - | - | n.d. | 395.2648 |
| **Peptaibol** | **y_7_** | **RT y_7_ (min)** | **R1** | **R2** | **R3** | **R4** | **R5** | **R6** | **R7** | **R8** | **R9** | **R10** | **R11** | **R12** | **R13** | **R14** | **R15** | **R16** | **R17** | **R18** | **R19** |
| **Trichorzianin TA-like I** | 855.5053 | 20.26 | 128.0728 | 199.1069 | 270.1441 | 355.1984 | 440.2501 | 568.3052 | 653.3542 | 738.4072 | 823.4608 | 910.4956 | 1023.5886 | 1108.6356 | n.d. | 211.1437 | 296.1975 | 409.2814 | 537.3387 | 665.3912 | 837.4930 |
| **Trichorzianin TA-like II** | 841.49 | 21.39 | 128.0727 | 199.1069 | 270.1442 | 355.1986 | 440.2503 | 568.3055 | 653.3547 | 738.4077 | 823.4614 | 910.4916 | 1023.5805 | 1108.6356 | n.d. | 197.1282 | 282.1813 | 395.2658 | 523.3231 | 651.3758 | 823.4755 |
| **Trichorzianin TA-like III** | 855.5053 | 23.02 | 128.0728 | 185.0909 | 256.1286 | 341.1828 | 440.2502 | 568.3052 | 653.3543 | 738.4073 | 823.4611 | 910.4955 | 1023.5874 | 1108.6358 | n.d. | 211.1438 | 296.1976 | 409.2814 | 537.3387 | 665.3915 | 837.4938 |
| **Trichorzianin TA-like IV** | 841.4896 | 24.2 | 128.0728 | 185.0913 | 256.1286 | 341.1829 | 440.2502 | 568.3042 | 653.3545 | 738.4075 | 823.4611 | 910.4944 | 1023.5888 | 1108.6359 | 98.0336 | 197.1282 | 282.1812 | 395.2657 | 523.323 | 651.3756 | 823.4772 |
| **Trichorzianin TA-like V** | 855.5047 | 25.43 | 128.0817 | 199.1438 | 270.1442 | 355.1984 | 440.2501 | 568.3054 | 653.3543 | 738.4072 | 823.4611 | 910.4944 | 1023.5888 | 1108.6355 | n.d. | 211.1437 | 296.1974. | 409.2811 | 537.3384 | 665.3909 | 837.4927 |
| **Trichorzianin TA-like VI** | 855.5052 | 25.9 | 128.0728 | 199.1069 | 270.1442 | 355.1984 | 440.2501 | 568.3053 | 653.3542 | 738.4072 | 823.461 | 910.4966 | 1023.5868 | 1108.6357 | 98.0409 | 211.1438 | 296.1975 | 409.2814 | 537.3388 | 665.3911 | 837.493 |
| **Trichorzianin TA-like VII** | 841.489 | 26.72 | 128.0729 | 199.1069 | 270.1441 | 355.1984 | 440.25 | 568.3052 | 653.3542 | 738.4071 | 823.4609 | 910.4932 | n.d. | n.d. | n.d. | 197.1281 | 282.1812 | 395.2656 | 523.3229 | 651.3754 | 823.4768 |
| **Trichorzianin TA-like VIII** | 816.4933 | 27.55 | 128.0725 | 199.1069 | 286.1398 | 371.1931 | 456.2445 | 584.3 | 669.3497 | 754.4044 | 839.4585 | 926.4882 | 1039.5817 | 1124.6303 | 98.0414 | 211.1137 | 296.1974 | 409.2812 | 537.3358 | 665.3912 | 798.4812 |
| **Trichorzianin TA-like IX** | 841.4897 | 28.13 | 128.0728 | 199.1071 | 270.1441 | 355.1984 | 454.2657 | 582.3208 | 653.3542 | 738.4072 | 823.4609 | 910.4951 | 1023.5762 | 1108.6362 | 98.0416 | 197.1281 | 282.1812 | 395.2656 | 523.3228 | 651.3755 | 823.4756 |
| **Trichorzianin TA-like X** | 855.5045 | 28.62 | 128.0769 | 199.107 | 270.1442 | 355.1985 | 454.2658 | 582.3209 | 667.3699 | 752.4242 | 837.4778 | 924.5106 | 1037.5923 | 1122.6513 | 98.0342 | 211.1438 | 296.1976 | 409.2814 | 537.3387 | 665.3913 | 837.4936 |
| **Trichorzianin TA-like XI** | 841.4893 | 28.84 | 128.0729 | 199.1069 | 270.1441 | 355.1984 | 454.2657 | 582.3207 | 667.3696 | 752.4239 | 837.4775 | 924.5128 | 1037.5934 | 1122.6513 | n.d. | 197.1281 | 282.1812 | 395.2656 | 523.3229 | 651.3755 | 823.4764 |
| **Trichorzianin TA-like XII** | 816.4931 | 28.98 | 128.0727 | 199.1069 | 270.1439 | 355.1985 | 440.2502 | 568.3058 | 653.3544 | 724.3918 | 809.4451 | 896.4833 | 1009.0903 | 1094.6195 | n.d. | 211.1438 | 296.1975 | 409.2813 | 537.3388 | 665.3912 | 798.4804 |
| **Trichorzianin TA-like XIII** | 855.5051 | 29.09 | 128.0728 | 199.1068 | 270.1441 | 355.1983 | 454.2657 | 582.3207 | 667.3696 | 752.4238 | 837.4774 | 924.5107 | 1037.5949 | 1122.6516 | 98.4355 | 211.1437 | 296.1974 | 409.2811 | 537.3384 | 665.3911 | 837.4935 |
| **Trichorzianin TA-like XIV** | 802.4776 | 29.25 | 128.073 | 199.1071 | 286.1397 | 371.1931 | 456.2445 | 584.3002 | 669.35 | 754.4046 | 839.459 | 926.4888 | 1039.0947 | 1124.63 | 98.0414 | 197.1281 | 282.1821 | 395.2656 | 523.3229 | 651.3754 | 784.4663 |
| **Trichorzianin TA-like XV** | 841.4890 | 29.94 | 128.0729 | 199.107 | 270.1442 | 355.1984 | 454.2658 | 582.3209 | 667.3698 | 752.4241 | 837.4777 | 924.5098 | 1037.5989 | 1122.6511 | 98.0408 | 197.1282 | 282.1812 | 395.2657 | 523.3231 | 651.3755 | 823.4764 |
| **Trichorzianin TA-like XVI** | 816.4933 | 30.54 | 128.0729 | 215.1014 | 286.1397 | 371.1931 | 470.2604 | 598.3157 | 683.3658 | 768.4192 | 853.4725 | 940.505 | 1053.5913 | 1138.6465 | n.d. | 211.1437 | 296.1974 | 409.2812 | 537.3384 | 665.3911 | 798.4808 |
| **Trichorzianin TA-like XVII** | 855.5053 | 31.09 | 128.0727 | 199.107 | 270.1443 | 355.1985 | 454.2659 | 582.3209 | 667.3702 | 752.4246 | 837.4781 | 908.5146 | 1021.5983 | 1106.657 | n.d. | 211.1438 | 296.1975 | 409.2813 | 537.3386 | 665.3913 | 837.4939 |
| **Trichorzianin TA-like XVIII** | 802.478 | 31.33 | 128..0730 | 199.1069 | 270.1443 | 355.1986 | 440.2503 | 568.3057 | 653.3547 | 724.3922 | 809.4456 | 896.4785 | 1009.5721 | 1094.6208 | 98.0333 | 197.1281 | 282.1812 | 395.2657 | 523.3231 | 651.3756 | 784.4648 |
| **Trichorzianin TA-like XIX** | 841.4894 | 31.97 | 128.0729 | 199.1069 | 270.1442 | 355.1983 | 454.2657 | 582.3207 | 667.3696 | 752.4238 | 837.4774 | 908.5145 | 1021.5964 | 1106.6567 | 98.0339 | 197.1282 | 282.1812 | 395.2657 | 523.323 | 651.3758 | 823.4771 |
| **Trichorzianin TA-like XX** | 802.4774 | 32.35 | 128.0773 | 215.1015 | 286.1398 | 371.1931 | 470.2604 | 598.3156 | 683.3659 | 768.4193 | 853.4728 | 940.5046 | 1053.6939 | 1138.6462 | n.d. | 197.1281 | 292.1812 | 395.2656 | 523.3229 | 651.3752 | 784.4656 |
| **Trichorzianin TA-like XXI** | 816.4929 | 32.78 | 128.073 | 199.1069 | 270.1442 | 355.1984 | 440.2501 | 568.3053 | 653.3543 | 738.4073 | 823.4612 | 910.4938 | 1023.5843 | 1108.6359 | n.d. | 211.1437 | 296.1974 | 409.2811 | 537.3384 | 665.3909 | 798.4803 |
| **Trichorzianin TA-like XXII** | 816.4927 | 33.16 | 128.0728 | 199.1069 | 270.144 | 355.1984 | 440.25 | 586.3052 | 653.3542 | 738.4072 | 823.4609 | 910.4944 | 1023.5822 | 1108.6356 | n.d. | 211.1438 | 296.1975 | 409.2813 | 537.3386 | 665.3912 | 798.4813 |
| **Trichorzianin TA-like XXIII** | 802.4775 | 33.81 | 128.0728 | 199.107 | 270.1443 | 355.1986 | 440.2502 | 568.3055 | 653.3546 | 738.4076 | 823.4614 | 910.4945 | 1023.5841 | 1108.6361 | 98.0342 | 198.1252 | 282.1812 | 395.2657 | 523.3231 | 651.376 | 784.4664 |
| **Trichorzianin TA-like XXIV** | 816.4932 | 34.42 | 128.0727 | 199.1071 | 270.1442 | 355.1985 | 454.2659 | 582.3211 | 653.3546 | 738.4076 | 823.4614 | 910.4959 | 1023.5819 | 1108.6364 | n.d. | 211.1438 | 296.1976 | 409.2815 | 537.3389 | 665.3916 | 798.4812 |
| **Trichorzianin TA-like XXV** | 802.4773 | 35.02 | 128.0730 | 199.107 | 270.1442 | 355.1985 | 440.2502 | 568.3055 | 653.3545 | 738.4075 | 823.4614 | 910.4911 | 1023.5833 | 1108.6356 | 98.0409 | 197.1281 | 282.1812 | 395.2656 | 523.3228 | 651.3755 | 784.4662 |
| **Trichorzianin TA-like XXVI** | 816.49 | 35.97 | 128.0728 | 199.107 | 270.1441 | 355.1983 | 454.2657 | 582.3208 | 667.3697 | 752.424 | 827.4775 | 924.5091 | 1037.5964 | 1122.6514 | n.d. | 211.1437 | 296.1974 | 409.2811 | 537.3384 | 665.3911 | 798.4805 |
| **Trichorzianin TA-like XXVII** | 816.4927 | 36.45 | 128.073 | 199.107 | 270.1442 | 355.1985 | 454.2659 | 582.321 | 667.37 | 752.4243 | 837.478 | 924.507 | 1037.5985 | 1122.6512 | 98.0443 | 211.1437 | 296.1974 | 409.2813 | 537.3384 | 665.3911 | 798.4816 |
| **Trichorzianin TA-like XXVIII** | 802.4775 | 37.03 | 128.0728 | 199.107 | 270.1443 | 355.1985 | 454.2659 | 582.321 | 667.37 | 752.4244 | 837.4778 | 924.511 | 1037.6002 | 1122.6511 | n.d. | 197.1282 | 282.1813 | 395.2657 | 523.3231 | 651.3757 | 784.4666 |
| **Trichorzianin TA-like XXIX** | 802.4771 | 38.2 | 128.0728 | 199.107 | 207.1441 | 355.1983 | 454.2657 | 582.3208 | 667.3697 | 752.4240 | 837.4775 | 924.5091 | 1037.5991 | 1122.6511 | 98.0335 | 197.1282 | 282.1813 | 395.2657 | 523.3231 | 651.3757 | 784.4655 |
| **Trichorzianin TA-like XXX** | 816.487 | 38.7 | 128.073 | 199.107 | 270.1443 | 355.1985 | 454.266 | 582.3211 | 667.37 | 752.4244 | 837.478 | 908.5157 | 1021.5994 | n.d. | 98.0414 | 211.1439 | 296.1997 | 409.2816 | 537.339 | 665.3917 | 798.4802 |
| **Trichorzianin TA-like XXXI** | 802.4772 | 40.1 | 128.0729 | 199.107 | 270.1443 | 355.1985 | 454.2659 | 582.231 | 667.37 | 752.4243 | 837.4778 | 908.5157 | 1021.5966 | 1106.6566 | n.d. | 197.1281 | 282.1812 | 395.2656 | 523.3231 | 651.3754 | 787.4666 |
| **Trichorzianin TA-like XXXII** | 816.4932 | 40.36 | 128.0728 | 199.107 | 270.1441 | 355.1984 | 454.2658 | 582.3208 | 667.3698 | 752.424 | 837.4777 | 924.5083 | 1037.5354 | 1122.6517 | n.d. | 211.1438 | 296.1975 | 409.2814 | 537.3387 | 665.3914 | 798.4809 |
| \| **Trichorzianin TA-like XXXIII** \| \| --- \| | 802.4772 | 42.16 | 128.0729 | 199.1071 | 270.1444 | 355.1986 | 454.2661 | 582.3212 | 667.3702 | 752.4264 | 837.4781 | 924.5153 | 1037.5416 | 1122.6512 | n.d. | 197.1282 | 282.1812 | 395.2657 | 523.323 | 651.3757 | 784.4663 |
| **Peptaibol** | **y_7_** | **RT y_7_ (min)** | **R1** | **R2** | **R3** | **R4** | **R5** | **R6** | **R7** | **R8** | **R9** | **R10** | **R11** | **R12** | **R13** | **R14** | **R15** | **R16** | **R17** | **R18** | **R19** |
| **Peptaibol-like I** | 683.4041 | 4.57 | 128.073 | 199.107 | 270.1443 | 355.1986 | 440.2503 | 568.3058 | 653.3547 | 738.4078 | 823.4617 | 910.4929 | 1023.5861 | 1108.6356 | 98.0411 | 211.143 | 296.1972 | 409.2813 | 537.3327 |  |  |
| **Peptaibol-like II** | 668.4039 | 5.69 | 128.0729 | 199.107 | 270.1441 | 355.1984 | 454.2658 | 582.3209 | 667.3699 | 752.4241 | 837.4777 | 924.5114 | 1037.601 | 1122.6515 | 98.0341 | 197.1282 | 282.1813 | 395.2658 | 523.3233 |  |  |
| **Peptaibol-like III** | 683.404 | 6.81 | 128.0728 | 199.1069 | 270.1443 | 355.1986 | 440.2503 | 568.3056 | 653.3548 | 738.4079 | 823.4616 | 910.4936 | 1023.5924 | 1108.6366 | n.d. | 211.1439 | 296.1977 | 409.2814 | 537.3391 |  |  |
| **Peptaibol-like IV** | 669.388 | 6.9 | 128.0728 | 199.1072 | 270.1442 | 355.1986 | 440.2503 | 568.3042 | 653.3547 | 738.4079 | 823.4612 | 910.495 | 1023.5935 | 1108.6366 | n.d. | 197.1283 | 282.1813 | 395.2658 | 523.3234 |  |  |
| **Peptaibol-like V** | 668.403 | 7.4 | 128.073 | 199.107 | 270.1442 | 355.1985 | 440.2502 | 568.3055 | 653.3546 | 738.4076 | 823.4614 | 910.494 | 1023.5905 | 1108.6356 | 98.0338 | 197.1281 | 282.1813 | 395.2661 | 523.3225 |  |  |
| **Peptaibol-like VI** | 683.4032 | 7.75 | 128.0731 | 199.107 | 270.1442 | 355.1985 | 440.2501 | 568.3054 | 653.3545 | 738.4075 | 823.4612 | 910.4944 | 1023.5849 | 1108.6351 | n.d. | 211.1438 | 296.1975 | 409.2813 | 537.3388 |  |  |
| **Peptaibol-like VII** | 669.3866 | 8.09 | 128.0727 | 199.107 | 270.1442 | 355.1985 | 440.2502 | 568.3055 | 653.3545 | 738.4075 | 823.4615 | 910.4962 | 1023.5805 | 1108.635 | 98.0431 | 197.1281 | 282.1812 | 395.2656 | 523.3228 |  |  |
| **Peptaibol-like VIII** | 682.4203 | 8.28 | 128.0721 | 199.1071 | 270.1443 | 355.1985 | 454.266 | 582.321 | 667.3704 | 752.4247 | 837.4781 | 924.5204 | 1037.5777 | 1122.6519 | n.d. | 211.1438 | 296.1975 | 409.2812 | 537.3387 |  |  |
| **Peptaibol-like IX** | 669.3875 | 9.12 | 128.073 | 199.1074 | 270.1443 | 355.1986 | 454.2661 | 582.3212 | 667.3704 | 752.4247 | 837.4785 | 924.512 | 1037.5926 | 1122.6524 | 98.366 | 197.1282 | 282.1813 | 395.2659 | 523.3234 |  |  |
| **Peptaibol-like X** | 683.4039 | 9.59 | 128.073 | 199.1068 | 270.1443 | 355.1985 | 454.266 | 582.3212 | 667.3702 | 752.4246 | 837.4783 | 924.5105 | 1037.5875 | 1122.6515 | 98.0337 | 211.1437 | 296.1975 | 409.2813 | 537.3387 |  |  |
| **Peptaibol-like XI** | 669.3869 | 9.97 | 128.0728 | 199.107 | 270.1442 | 355.0985 | 454.2658 | 582.3211 | 667.37 | 752.4243 | 837.4779 | 924.5055 | 1037.5874 | 1122.6513 | n.d. | 197.1282 | 282.1812 | 395.2657 | 523.3231 |  |  |
| **Peptaibol-like XII** | 668.4038 | 10.27 | 128.0731 | 199.1072 | 270.1443 | 355.1986 | 454.2661 | 582.321 | 667.3704 | 752.4257 | 837.4786 | 924.509 | 1037.5839 | 1122.6513 | n.d. | 197.1282 | 282.1814 | 395.2658 | 523.3231 |  |  |
| **Peptaibol-like XIII** | 682.4193 | 10.64 | 128.073 | 199.1072 | 270.1443 | 355.1986 | 440.2504 | 568.3055 | 653.3547 | 738.4078 | 823.4617 | 910.4935 | 1023.5806 | 1108.6371 | n.d. | 211.1438 | 296.1975 | 409.2813 | 537.3387 |  |  |
| **Peptaibol-like XIV** | 668.4029 | 11.97 | 128.0727 | 199.1069 | 270.1442 | 355.1984 | 440.2501 | 568.3054 | 653.3543 | 738.4073 | 823.4613 | 910.4926 | 1023.5815 | 1108.6356 | 98.0409 | 197.1281 | 282.1812 | 395.2656 | 523.3229 |  |  |
| **Peptaibol-like XV** | 682.42 | 12.19 | 128.073 | 199.1069 | 270.1443 | 355.1985 | 440.2502 | 568.3055 | 653.3545 | 738.4075 | 823.4614 | 910.4948 | 1023.5843 | 1108.6359 | n.d. | 211.1438 | 296.1975 | 409.2812 | 537.3387 |  |  |
| **Peptaibol-like XVI** | 668.4036 | 13.37 | 128.0731 | 199.1072 | 270.1442 | 355.1985 | 454.2659 | 582.3209 | 667.3701 | 752.4244 | 837.4779 | 924.5107 | 1037.5803 | 1122.6525 | n.d. | 197.1282 | 282.1813 | 395.2657 | 523.3234 |  |  |
| **Peptaibol-like XVII** | 682.4201 | 13.82 | 128.0731 | 199.1069 | 270.1441 | 355.1985 | 440.2502 | 568.3055 | 653.3546 | 738.4077 | 823.4614 | 910.4948 | 1023.5813 | 1108.6367 | n.d. | 211.1437 | 296.1976 | 409.2813 | 537.3393 |  |  |
| **Peptaibol-like XVIII** | 668.4028 | 14.45 | 128.0728 | 199.1069 | 270.144 | 355.1983 | 454.2656 | 582.3207 | 667.3696 | 752.4238 | 837.4772 | 924.5107 | 1037.5872 | 1122.651 | n.d. | 197.1281 | 282.1812 | 395.2657 | 523.3229 |  |  |
| **Peptaibol-like XIX** | 682.4196 | 14.73 | 128.0731 | 199.1069 | 270.1441 | 355.1984 | 454.2657 | 582.3209 | 667.3698 | 752.4241 | 837.4778 | 924.5101 | 1037.5919 | 1122.6511 | 98.0345 | 211.1437 | 296.1975 | 409.2813 | 537.3386 |  |  |
| **Peptaibol-like XX** | 683.4027 | 14.73 | 128.0731 | 199.1069 | 270.1441 | 355.1984 | 454.2657 | 582.3209 | 667.3698 | 752.4241 | 837.4778 | 924.5101 | 1037.5919 | 1122.6511 | n.d. | 197.1282 | 282.1813 | 395.266 | 523.3237 |  |  |
| **Peptaibol-like XXI** | 668.4035 | 16.01 | 128.0728 | 199.1068 | 270.1442 | 355.1984 | 454.2658 | 582.3209 | 667.3699 | 752.4243 | 837.4775 | 924.5078 | 1037.5966 | 1122.652 | 98.0411 | 197.1283 | 282.1813 | 395.2659 | 523.3233 |  |  |
| **Peptaibol-like XXII** | 682.4224 | 16.51 | 128.0729 | 199.107 | 270.1441 | 355.1983 | 440.2499 | 568.3051 | 653.3541 | 738.407 | 823.4608 | 910.4926 | 1023.5817 | 1108.64 | 98.0666 | 211.1438 | 296.1975 | 409.2812 | 523.3387 |  |  |
| **Peptaibol-like XXIII** | 683.4035 | 16.93 | 128.0728 | 199.1069 | 270.144 | 355.1983 | 440.25 | 568.3052 | 653.3541 | 738.4071 | 823.4609 | 910.4925 | 1023.5814 | 1108.6355 | 98.0351 | 197.1281 | 282.1812 | 395.2656 | 523.323 |  |  |
| **Peptaibol-like XXIV** | 683.4089 | 18.36 | 128.0728 | 199.1068 | 270.1441 | 355.1983 | 440.2499 | 568.3051 | 653.3541 | 738.4069 | 823.4607 | 910.4942 | 1023.5812 | 1108.64 | 98.2474 | 197.1282 | 282.1814 | 395.2659 | 523.3234 |  |  |
| **Peptaibol-like XXV** | 683.4036 | 19.95 | 128.0729 | 199.1069 | 270.1441 | 355.1983 | 454.2656 | 582.3206 | 667.3696 | 752.4238 | 837.4773 | 924.509 | 1037.5873 | 1122.6512 | 98.0412 | 197.1282 | 282.1812 | 395.2657 | 523.3229 |  |  |
| **Peptaibol-like XXVI** | 683.4038 | 21.55 | 128.0729 | 199.1068 | 270.1442 | 355.1985 | 454.2658 | 582.321 | 667.3699 | 752.4242 | 837.4777 | 924.5098 | 1037.6011 | 1122.6519 | 98.0412 | 197.1282 | 282.1813 | 395.266 | 523.3235 |  |  |
| **Peptaibol-like XXVII** | 873.4789 | 9.81 | 128.0729 | 199.1069 | 270.1441 | 355.1983 | 440.25 | 568.3051 | 653.3541 | 738.407 | 823.4608 | 910.4931 | 1023.5512 | 1108.6355 | n.d. | 197.1281 | 282.1812 | 395.2656 | 523.3229 | 651.3757 |  |
| **Peptaibol-like XXVIII** | 887.4956 | 10.53 | 128.073 | 199.1072 | 270.1443 | 355.1986 | 440.2504 | 568.3055 | 653.3547 | 738.4078 | 823.4617 | 910.4935 | 1023.5833 | 1108.6368 | n.d. | 211.1439 | 296.1976 | 409.2815 | 537.339 | 665.3917 |  |
| **Peptaibol-like XXIX** | 873.4786 | 11.6 | 128.0731 | 199.1069 | 270.144 | 355.1982 | 440.2498 | 568.305 | 653.3539 | 738.4068 | 823.4607 | 910.4962 | 1023.5781 | 1108.6355 | n.d. | 197.1281 | 282.1812 | 395.2657 | 523.3229 | 651.3756 |  |
| **Peptaibol-like XXX** | 873.4786 | 11.9 | 128.0728 | 199.1069 | 270.1442 | 355.1984 | 440.2501 | 568.3054 | 653.3543 | 738.4073 | 823.4613 | 910.4926 | 1023.5816 | 1108.635 | 98.0342 | 197.1281 | 282.1812 | 395.2656 | 523.3229 | 651.3755 |  |
| **Peptaibol-like XXXI** | 887.4948 | 12.00 | 128.0727 | 199.1069 | 270.1442 | 355.1984 | 440.2501 | 568.3054 | 653.5343 | 738.4073 | 823.4613 | 910.4926 | 1023.582 | 1108.6356 | 98.0415 | 211.1438 | 296.1975 | 409.2814 | 537.3387 | 665.3919 |  |
| **Peptaibol-like XXXII** | 887.4957 | 12.78 | 128.0726 | 199.1069 | 270.1442 | 355.1985 | 454.2659 | 582.3209 | 667.37 | 752.4242 | 837.478 | 924.5088 | 1037.5804 | 1122.6523 | n.d. | 211.1438 | 296.1976 | 409.2815 | 537.3388 | 665.3916 |  |
| \| **Peptaibol-like XXXIII** \| \| --- \| | 873.4789 | 14.04 | 128.0727 | 199.1069 | 270.1441 | 355.1984 | 454.2657 | 582.3206 | 667.3696 | 752.4239 | 837.4774 | 924.5119 | 1037.5855 | 1122.6513 | n.d. | 197.1281 | 282.1812 | 395.2656 | 523.3229 | 651.3755 |  |
| **Peptaibol-like XXXIV** | 887.4942 | 14.45 | 128.0729 | 199.1069 | 270.144 | 355.1983 | 440.2499 | 568.3051 | 653.354 | 738.4069 | 823.4608 | 910.4951 | 1023.5789 | 1108.6354 | n.d. | 211.1437 | 296.1975 | 409.2813 | 537.3389 | 665.3915 |  |
| **Peptaibol-like XXXV** | 873.4797 | 15.49 | 128.0728 | 199.107 | 270.1442 | 355.1984 | 454.2658 | 582.3205 | 667.3699 | 752.4241 | 837.4775 | 924.5097 | 1037.6965 | 1122.6515 | n.d. | 197.1283 | 282.1812 | 395.2659 | 523.3232 | 651.3759 |  |
| **Peptaibol-like XXXVI** | 887.4944 | 16.57 | 128.0729 | 199.107 | 270.1441 | 355.1983 | 440.2499 | 568.3051 | 653.3541 | 738.407 | 823.4608 | 910.4926 | 1023.5749 | 1108.6358 | n.d. | 211.1438 | 296.1975 | 409.281 | 537.3386 | 665.3868 |  |
| **Peptaibol-like XXXVII** | 887.4953 | 17.62 | 128.0729 | 199.107 | 270.1442 | 355.1984 | 440.2501 | 568.3054 | 653.3542 | 738.4071 | 823.4609 | 910.4934 | 1023.574 | 1108.6361 | 98.0418 | 211.1437 | 296.1975 | 409.2812 | 537.3386 | 665.3917 |  |
| **Peptaibol-like XXXVIII** | 873.4793 | 17.81 | 128.0729 | 199.1069 | 270.1441 | 355.1983 | 440.2499 | 568.3051 | 653.354 | 738.4069 | 823.4606 | 910.4941 | 1023.5841 | 1108.6363 | n.d. | 197.1281 | 282.1812 | 395.2656 | 523.3228 | 651.3758 |  |
| \| **Peptaibol-like XXXIX** \| \| --- \| | 887.4951 | 19.43 | 128.0727 | 199.1069 | 270.1442 | 355.1984 | 454.2657 | 582.3206 | 667.3699 | 752.4239 | 837.4774 | 924.512 | 1037.5899 | 1122.6517 | n.d. | 211.1436 | 296.1978 | 409.2808 | 537.3381 | 665.3939 |  |
| \| **Peptaibol-like XL** \| \| --- \| | 887.4945 | 20.6 | 128.0728 | 199.1068 | 270.1441 | 355.1983 | 454.2657 | 582.3207 | 667.3936 | 752.3238 | 837.3775 | 924.5092 | 1037.5946 | 1122.6513 | n.d. | 211.1438 | 296.1976 | 409.2814 | 537.3386 | 665.3914 |  |
| **Peptaibol-like XLI** | 873.4793 | 20.69 | 128.0729 | 199.1069 | 270.1442 | 355.1984 | 454.2658 | 582.3209 | 667.3697 | 752.424 | 837.4775 | 924.5104 | 1037.5998 | 1122.6515 | n.d. | 197.1281 | 282.1812 | 395.2656 | 523.3228 | 651.3755 |  |
| \| **Peptaibol** \| \| --- \| | **y_7_** | **RT y_7_ (min)** | **R1** | **R2** | **R3** | **R4** | **R5** | **R6** | **R7** | **R8** | **R9** | **R10** | **R11** | **R12** | **R13** | **R14** | **R15** | **R16** | **R17** | **R18** | **R19** |
| **Tricholongin LB-like I** | 754.4781 | 16.82 | 128.0724 | 185.1932 | 332.1616 | 417.2135 | 502.2654 | 630.3181 | 715.371 | 800.4243 | 885.4775 | 972.515 | 1085.7421 | 1170.6563 | n.d. | 197.128 | 282.181 | 381.25 | 509.307 | 637.3593 | 736.4614 |
| **Tricholongin LB-like II** | 740.4605 | 19.69 | 128.0724 | 185.0912 | 256.1284 | 341.1287 | 426.2346 | 554.2893 | 639.3378 | 724.3916 | 809.4449 | 896.4817 | 1009.6986 | 1094.6185 | n.d. | 197.1281 | 282.1811 | 367.2346 | 495.2918 | 623.3442 | 722.448 |
| **Tricholongin LB-like III** | 740.4608 | 20.39 | 114.0582 | 171.1127 | 318.146 | 403.1977 | 488.2497 | 616.3021 | 701.355 | 786.4086 | 871.4626 | 958.4946 | 1071.7394 | 1156.6363 | n.d. | 197.1281 | 282.1811 | 367.2345 | 495.2918 | 623.3444 | 722.4498 |
| **Tricholongin LB-like IV** | 754.4775 | 22.32 | 128.1726 | 185.0912 | 256.1283 | 341.1826 | 426.2345 | 554.2891 | 639.3386 | 724.3914 | 809.4449 | 896.4777 | 10096971 | 1094.6196 | n.d. | 197.1281 | 282.1811 | 381.2501 | 509.3071 | 637.36 | 736.4645 |
| **Tricholongin LB-like V** | 740.469 | 22.83 | 128.0732 | 185.0914 | 348.1562 | 433.2079 | 518.2596 | 646.3121 | 731.3652 | 816.4181 | 901.4726 | 988.502 | 1101.7084 | 1186.6457 | n.d. | 197.1281 | 282.1811 | 367.2345 | 495.2918 | 623.3444 | 722.4496 |
| **Tricholongin LB-like VI** | 754.4774 | 22.94 | 114.0581 | 171.1126 | 318.1459 | 403.1976 | 488.2497 | 616.3021 | 701.3549 | 786.4084 | 871.4623 | 958.4971 | 1071.739 | 1156.5352 | n.d. | 197.1281 | 282.1811 | 381.2501 | 509.3072 | 637.3601 | 736.4651 |
| **Tricholongin LB-like VII** | 754.4777 | 25.48 | 128.0733 | 185.0916 | 348.156 | 4332073 | 518.2596 | 646.312 | 731.3651 | 816.4183 | 901.4724 | 988.5018 | 1101.7783 | 1186.6448 | n.d. | 197.1821 | 282.181 | 381.25 | 509.307 | 637.3599 | 736.4645 |
| **Tricholongin LB-like VIII** | 740.4607 | 26.14 | 128.073 | 185.0911 | 332.1614 | 417.2132 | 502.265 | 630.3157 | 701.3549 | 786.4084 | 871.4622 | 958.4916 | 1071.7392 | 1156.6356 | 98.041 | 197.1821 | 282.1811 | 367.2345 | 495.2918 | 623.3445 | 722.4494 |
| **Tricholongin LB-like IX** | 740.4608 | 26.66 | 128.0731 | 185.0912 | 332.1613 | 403.1975 | 488.2496 | 616.3018 | 701.3548 | 786.4082 | 871.4623 | 958.4914 | 1071.7395 | 1156.6351 | 98.0423 | 197.1281 | 282.1812 | 367.2346 | 495.292 | 623.3446 | 722.4499 |
| **Tricholongin LB-like X** | 754.4774 | 27.13 | 128.1729 | 185.091 | 332.1613 | 20.febr | 488.2491 | 616.3018 | 701.3542 | 786.4077 | 871.4615 | 958.4973 | 1071.7358 | 1156.6345 | n.d. | 197.1281 | 282.1811 | 381.25 | 509.3071 | 637.3599 | 736.4659 |
| **Tricholongin LB-like XI** | 740.4607 | 27.43 | 128.0732 | 185.0911 | 332.1614 | 417.2132 | 502.265 | 630.3173 | 715.3703 | 800.4236 | 885.577 | 972.5111 | 1085.7438 | 1170.6507 | n.d. | 197.128 | 282.1811 | 367.2347 | 495.2912 | 623.2346 | 722.4491 |
| **Tricholongin LB-like XII** | 754.4772 | 28.14 | 128.1732 | 185.0912 | 332.1615 | 417.2133 | 502.2652 | 630.3178 | 715.3706 | 786.4086 | 871.4623 | 958.4946 | 1071.7395 | 1156.6348 | n.d. | 197.1281 | 282.1811 | 381.2501 | 509.3071 | 637.36 | 736.464 |
| **Tricholongin LB-like XIII** | 740.467 | 28.7 | 128.073 | 185.091 | 332.1614 | 417.2132 | 502.265 | 630.3175 | 715.3705 | 800.4238 | 885.4775 | 972.5103 | 1085.7545 | 1170.6508 | n.d. | 197.1281 | 282.1811 | 367.2345 | 495.2918 | 623.3443 | 722.4493 |
| **Tricholongin LB-like XIV** | 754.4773 | 29.36 | 128.073 | 185.0913 | 332.1614 | 403.1975 | 488.2496 | 616.3021 | 701.3548 | 786.4083 | 871.4623 | 958.5038 | 1071.7391 | 1156.6353 | n.d. | 197.128 | 282.1812 | 381.2501 | 509.3072 | 637.3604 | 736.4658 |
| **Tricholongin LB-like XV** | 740.461 | 29.91 | 228.083 | 185.0913 | 332.1614 | 417.2131 | 502.2649 | 630.3173 | 715.3702 | 800.4235 | 885.4771 | 972.5117 | 1085.7533 | 1170.6509 | 98.1424 | 197.1281 | 282.1811 | 367.2346 | 495.2918 | 623.3444 | 722.4492 |
| **Tricholongin LB-like XVI** | 754.4774 | 31.37 | 228.0734 | 185.0938 | 332.1613 | 417.2131 | 502.2649 | 630.3173 | 715.3702 | 800.4235 | 885.4772 | 972.5134 | 1085.7646 | 1170.6509 | n.d. | 197.1281 | 282.1812 | 381.25 | 509.307 | 637.36 | 736.4643 |
| **Tricholongin LB-like XVII** | 740.4609 | 32.02 | 128.0731 | 199.1071 | 346.1758 | 431.2285 | 516.2803 | 644.3331 | 729.3859 | 814.4393 | 899.4932 | 986.5237 | 1099.6121 | 1184.6664 | n.d. | 197.1281 | 282.1811 | 367.2346 | 495.2917 | 623.3446 | 722.4495 |
| **Tricholongin LB-like XVIII** | 754.4774 | 32.53 | 128.0732 | 185.0911 | 332.1615 | 417.2133 | 502.2651 | 630.3177 | 715.3705 | 800.4238 | 885.4772 | 972.5179 | 1085.7547 | 1170.6508 | 98.0431 | 197.1282 | 282.1812 | 381.2502 | 509.3073 | 637.3605 | 736.4643 |
| **Tricholongin LB-like XIX** | 754.4776 | 34.64 | 128.0823 | 199.107 | 346.1769 | 431.2285 | 516.2803 | 644.3333 | 729.3859 | 814.4393 | 899.4932 | 986.53 | 1099.7715 | 1184.6667 | 98.034 | 197.1281 | 282.1812 | 381.2502 | 509.3073 | 637.3601 | 736.4647 |
| **Tricholongin LB-like XX** | 740.461 | 34.74 | 128.0731 | 199.1071 | 346.1772 | 431.2288 | 516.2806 | 644.3336 | 729.3864 | 814.4398 | 899.4939 | 970.5237 | 1083.7465 | 1168.6716 | 98.0414 | 197.1281 | 282.1812 | 367.2346 | 495.2918 | 623.3445 | 722.4498 |
| **Tricholongin LB-like XXI** | 754.4775 | 37.27 | 128.0732 | 199.107 | 346.1772 | 431.2283 | 516.28 | 644.3329 | 729.3854 | 814.4387 | 899.4927 | 970.5295 | 1083.6128 | 1168.6716 | 98.0414 | 197.128 | 282.181 | 381.2499 | 509.3068 | 637.3597 | 736.4641 |
| **Peptaibol** | **y_7_** | **RT y_7_ (min)** | **R1** | **R2** | **R3** | **R4** | **R5** | **R6** | **R7** | **R8** | **R9** | **R10** | **R11** | **R12** | **R13** | **R14** | **R15** | **R16** | **R17** | **R18** | **R19** |
| **Dorothopsin B I** | 726.4458 | 20.25 | 190.0885 | n.d. | 332.1618 | 417.2137 | 516.2809 | 644.334 | 729.3869 | 814.4405 | 899.4938 | 986.5295 | 1085.738 | 1170.6517 | n.d. | 197.1281 | 281.1812 | 367.2346 | 495.2919 | 623.3446 | 708.4335 |
| **Dorothopsin B II** | 727.4289 | 20.39 | 190.0897 | 247.1087 | 332.1616 | 417.2135 | 516.2806 | 644.3337 | 729.3864 | 814.4399 | 899.4937 | 986.5245 | 1085.7382 | 11706517 | 98.0413 | 197.1282 | 282.1812 | 367.2347 | 496.2764 | 624.3302 | 709.4194 |
| **Dorothopsin B III** | 726.4456 | 21.53 | 190.0879 | 247.1072 | 332.1617 | 417.2136 | 530.2964 | 658.3491 | 743.402 | 814.4401 | 899.4944 | 986.5229 | 1099.751 | 1184.6672 | n.d. | 197.1281 | 282.1812 | 367.2346 | 495.2921 | 623.3443 | 708.4344 |
| **Dorothopsin B IV** | 727.4284 | 21.68 | n.d. | 247.108 | 332.1617 | 417.2136 | 530.2965 | 658.3487 | 743.4022 | 814.4401 | 899.4942 | 986.5305 | 1099.751 | 1184.6672 | n.d. | 197.1282 | 282.1813 | 367.2343 | 496.2764 | 624.3305 | 709.4196 |
| **Dorothopsin B V** | 726.4458 | 22.34 | 190.0892 | 247.1103 | 332.1615 | 417.2133 | 516.2805 | 644.3334 | 729.386 | 814.4395 | 899.4938 | 986.5335 | 1099.7517 | 1185.6674 | n.d. | 197.1282 | 282.1813 | 367.2347 | 495.2921 | 623.3447 | 708.434 |
| **Dorothopsin B VI** | 727.4294 | 22.51 | 190.0895 | 247.1065 | 332.1616 | 417.2135 | 516.2806 | 644.3336 | 729.3863 | 814.4398 | 899.494 | 986.5282 | 1099.7462 | 1184.5571 | n.d. | 197.1282 | 282.1812 | 367.2347 | 496.2763 | 624.3299 | 709.4195 |
| **Dorothopsin B VII** | 726.4456 | 23.4 | 229.0989 | 286.1196 | 371.17 | 456.2241 | 569.3046 | 697.3603 | 782.4138 | 867.4676 | 952.5213 | 1039.5594 | 1152.6412 | 1237.6937 | 98.0416 | 197.1282 | 282.1813 | 367.2347 | 495.2921 | 623.3447 | 708.434 |
| **Dorothopsin B VIII** | 727.4293 | 23.65 | 229.0994 | 286.1186 | 371.17 | 456.2241 | 569.3047 | 597.3602 | 728.4138 | 867.4677 | 952.5214 | 1039.5549 | 1152.6414 | 1237.6939 | n.d. | 197.1282 | 282.1813 | 367.2348 | 496.2767 | 624.3305 | 709.42 |
| **Dorothopsin B IX** | 726.4458 | 23.81 | 190.0878 | 247.1089 | 332.1617 | 417.2136 | 530.2966 | 658.3489 | 743.4022 | 814.4403 | 899.4941 | 986.5276 | 1099.75 | 1184.6671 | 98.0416 | 197.1283 | 282.1814 | 367.2349 | 495.2923 | 623.345 | 708.4343 |
| **Dorothopsin B X** | 727.4285 | 24.06 | 190.0891 | 247.1096 | 332.1616 | 417.2135 | 530.2963 | 658.3489 | 743.4017 | 814.4392 | 899.4936 | n.d. | 1099.7516 | 1184.6663 | n.d. | 197.1282 | 282.1813 | 367.2348 | 496.2763 | 624.3293 | 709.4189 |
| **Dorothopsin B XI** | 726.446 | 25.05 | 190.0892 | 247.1098. | 332.1615 | 417.2134 | 516.2805 | 644.3337 | 729.3862 | 814.4396 | 899.4933 | 986.5275 | 1099.7489 | 1184.6677 | n.d. | 197.1282 | 282.1812 | 367.2347 | 495.2921 | 623.3448 | 708.4344 |
| **Dorothopsin B XII** |  |  | 229.1339 | 286.1185 | 05.jan | 456.2241 | 569.3046 | 697.3603 | 782.4139 | 867.4677 | 952.5215 | 1039.5552 | n.d. | 1237.694 | n.d. | 197.1282 | 282.1812 | 367.2347 | 495.2921 | 623.3448 | 708.4344 |
| **Dorothopsin B XIII** | 726.4454 | 25.99 | 190.0891 | 247.1059 | 332.1616 | 417.2135 | 530.2963 | 658.3487 | 743.4017 | 828.4553 | 913.5072 | 1000.5419 | 1113.7664 | 1198.6826 | n.d. | 197.1282 | 282.1813 | 367.2348 | 495.2921 | 623.3448 | 708.4342 |
| **Dorothopsin B XIV** |  |  | 229.0959 | 286.1183 | 371.17 | 456.2238 | 569.3043 | 697.3598 | 782.4133 | 867.4672 | 952.5207 | 1039.5554 | 1152.6888 | 1237.693 | n.d. | 197.1282 | 282.1813 | 367.2348 | 495.2921 | 623.3448 | 708.4342 |
| **Dorothopsin B XV** | 727.4284 | 26.18 | 190.0885 | 247.1097 | 332.1616 | 417.2135 | 530.2963 | 658.3489 | 743.4017 | 828.4554 | 913.5083 | 1000.5419 | 1113.7656 | 1198.6824 | n.d. | 197.1283 | 282.1813 | 367.2349 | 496.2769 | 624.3314 | 709.4209 |
| **Dorothopsin B XVI** |  |  | 229.994 | 286.1182 | 371.17 | 456.2242 | 569.3047 | 697.3607 | 782.4132 | 867.4678 | 952.5206 | 1039.5574 | 1152.6888 | 1237.693 | n.d. | 197.1283 | 282.1813 | 367.2349 | 496.2769 | 624.3314 | 709.4209 |
| **Dorothopsin B XVII** | 726.4459 | 27.78 | 190.0877 | 247.1074 | 332.1617 | 417.2136 | 516.3808 | 644.3337 | 729.3865 | 814.4401 | 899.4997 | 986.5258 | 1099.7617 | 1184.6671 | 98.034 | 197.1282 | 282.1813 | 367.2347 | 495.2921 | 623.3448 | 708.3431 |
| **Dorothopsin B XVIII** |  |  | 229.0984 | 2861196 | 371.17 | 456.2241 | 569.3047 | 697.3605 | 782.4139 | 867.4679 | 952.5212 | 1039.548 | n.d. | 1237.6938 | 98.034 | 197.1282 | 282.1813 | 367.2347 | 495.2921 | 623.3448 | 708.3431 |
| **Dorothopsin B XIX** |  |  | 190.0889 | 247.1085 | 332.16 | 417.2133 | 530.2961 | 658.3487 | 743.4014 | 828.4551 | 913.5082 | 1000.5419 | 1113.7684 | 1198.6832 | 98.034 | 197.1282 | 282.1813 | 367.2347 | 495.2921 | 623.3448 | 708.3431 |
| **Dorothopsin B XX** |  |  | 229.0963 | 300.1343 | 385.19 | 470.2392 | 583.3201 | 711.376 | 796.429 | 881.4829 | 966.5376 | 1053.5583 | 1166.6522 | 1251.7093 | 98.034 | 197.1282 | 282.1813 | 367.2347 | 495.2921 | 623.3448 | 708.3431 |
| **Dorothopsin B XXI** | 727.4298 | 28.01 | 190.0877 | 247.1074 | 332.16 | 417.2136 | 516.2803 | 644.3337 | 729.3865 | 814.4401 | 899.4947 | 986.5269 | 1099.7753 | 1184.6685 | 98.041 | 197.1281 | 282.1812 | 367.2347 | 496.2766 | 624.3309 | 709.4199 |
| **Dorothopsin B XXII** |  |  | 229.0994 | 286.1192 | 371.17 | 456.2242 | 569.3047 | 697.3602 | 782.4141 | 867.4679 | 952.5208 | 1039.5604 | n.d. (1152) | 1237.6937 | 98.041 | 197.1281 | 282.1812 | 367.2347 | 496.2766 | 624.3309 | 709.4199 |
| **Dorothopsin B XXIII** |  |  | 190.0893 | 247.1083 | 332.16 | 417.2135 | 530.2963 | 658.3486 | 743.4017 | 828.4553 | 913.5085 | 1000.5422 | 1113.7685 | 1198.6832 | 98.041 | 197.1281 | 282.1812 | 367.2347 | 496.2766 | 624.3309 | 709.4199 |
| **Dorothopsin B XXIV** |  |  | 229.0959 | 300.1344 | 385.19 | 470.2394 | 583.3204 | 711.3762 | 796.4294 | 881.4833 | 966.5377 | 1053.5667 | 1166.6522 | 1251.7093 | 98.041 | 197.1281 | 282.1812 | 367.2347 | 496.2766 | 624.3309 | 709.4199 |
| **Dorothopsin B XXV** | 726.4456 | 28.58 | n.d. | 247.1092 | 332.1614 | 417.2133 | 530.2961 | 658.3486 | 743.4014 | 828.4548 | 913.5084 | 1000.5443 | 1113.6292 | 1198.8228 | 98.034 | 197.1282 | 282.1813 | 367.2347 | 495.2921 | 623.3448 | 708.4341 |
| **Dorothopsin B XXVI** | 727.4288 | 28.73 | n.d. | n.d. | 332.1616 | 417.2134 | 530.2962 | 658.3488 | 743.4016 | 828.4552 | 913.5082 | 1000.549 | 1113.7652 | 1198.6823 | 98.0429 | 197.1282 | 282.1812 | 367.2347 | 496.2754 | 624.3301 | 709.42 |
| **Dorothopsin B XXVII** | 726.4459 | 29.68 | 190.089 | 261.1232 | 346.1771 | 431.2288 | 544.3118 | 672.3646 | 757.4185 | 842.4721 | 927.5251 | 1014.5605 | 1127.6415 | 1212.6989 | n.d. | 197.1282 | 282.1813 | 367.2347 | 495.2921 | 623.3447 | 708.4338 |
| **Dorothopsin B XXVIII** | 727.4292 | 29.89 | 190.0892 | 261.1233 | 346.1771 | 431.2287 | 544.3116 | 672.3646 | 757.4183 | 842.4719 | 927.5249 | 1014.5641 | 1127.6403 | 1212.6986 | n.d. | 197.1282 | 282.1813 | 367.2348 | 496.2765 | 624.3304 | 709.42 |
| **Dorothopsin B XXIX** | 726.4459 | 30.57 | 190.0892 | 247.1087 | 332.1616 | 417.2135 | 530.2963 | 658.3487 | 743.4018 | 828.4554 | 913.5082 | 1000.5434 | 1113.6285 | 1198.6831 | 98.0405 | 197.1282 | 282.1813 | 367.2348 | 495.2921 | 623.3449 | 708.4348 |
| **Dorothopsin B XXX** |  |  | 229.0961 | 300.1346 | 19.jan | 470.2393 | 583.3203 | 711.3759 | 796.4291 | 881.483 | 966.3577 | 1053.5603 | 1166.6564 | 1251.7093 | 98.0405 | 197.1282 | 282.1813 | 367.2348 | 495.2921 | 623.3449 | 708.4348 |
| \| **Dorothopsin B XXXI** \| \| --- \| | 727.4295 | 30.6 | 190.0892 | 247.1087 | 332.1616 | 417.2135 | 530.2963 | 658.3487 | 743.4018 | 828.4554 | 913.5082 | 1000.5434 | 1113.6285 | 1198.6831 | n.d. | 197.1282 | 282.1813 | 367.2348 | 496.2764 | 624.3301 | 709.3195 |
| \| **Dorothopsin B XXXII** \| \| --- \| |  |  | 229.0961 | 300.1346 | 19.jan | 470.2393 | 583.3203 | 711.3759 | 796.4291 | 881.483 | 966.3577 | 1053.5603 | 1166.6564 | 1251.7093 | n.d. | 197.1282 | 282.1813 | 367.2348 | 496.2764 | 624.3301 | 709.3195 |
| \| **Dorothopsin B XXXIII** \| \| --- \| | 726.4456 | 32.41 | 190.0889 | 261.1224 | 346.1769 | 431.2286 | 544.3115 | 672.4644 | 757.4181 | 842.4716 | 927.5241 | 1014.5692 | 1127.7827 | 1212.6981 | 98.034 | 197.1282 | 282.1813 | 367.2347 | 495.2921 | 623.3448 | 708.4341 |
| \| **Dorothopsin B XXXIV** \| \| --- \| | 727.429 | 32.56 | 190.089 | 261.1226 | 346.177 | 431.2287 | 544.3116 | 672.3645 | 757.4183 | 842.4718 | 927.5247 | 1014.5638 | 1127.6159 | 1212.6981 | n.d. | 197.1281 | 282.1812 | 367.2347 | 496.2766 | 624.3309 | 709.4199 |
| \| **Dorothopsin B XXXV** \| \| --- \| | 726.4456 | 35.05 | 190.0891 | 261.1227 | 336.177 | 431.2287 | 544.3115 | 672.3646 | 767.4182 | 842.4718 | 927.5247 | 1014.5618 | 1127.7832 | 1212.6983 | n.d. | 197.1282 | 282.1813 | 367.2347 | 495.2921 | 623.3448 | 708.434 |
| \| **Dorothopsin B XXXVI** \| \| --- \| | 727.43 | 35.18 | 190.0891 | 261.1227 | 336.177 | 431.2287 | 544.3115 | 672.3646 | 767.4182 | 842.4718 | 927.5247 | 1014.5618 | 1127.7832 | 1212.6983 | n.d. | 197.1281 | 282.1812 | 367.2347 | 496.2765 | 624.3304 | 709.4196 |
| **Peptaibol** | **y_7_** | **RT y_7_ (min)** | **R1** | **R2** | **R3** | **R4** | **R5** | **R6** | **R7** | **R8** | **R9** | **R10** | **R11** | **R12 (b_12_)** | **R13** | **R14** | **R15** | **R16** | **R17** | **R18** | **R19** |
| **Strigosellin A I** | 789.4447 | 12.97 | 128.073 | 215.1015 | 300.1559 | 371.1931 | 456.245 | 584.3002 | 669.3493 | 754.4036 | 825.4399 | 896.4778 | 995.5515 | 1080.604 | n.d. | 245.128 | 330.0823 | 415.2343 | 544.2766 | 672.3307 | 771.4374 |
| **Strigosellin A II** | 788.4615 | 13.58 | 128.0731 | 215.1019 | 300.056 | 371.1931 | 456.245 | 584.3002 | 669.3492 | 754.4035 | 825.4399 | 896.4782 | 995.5484 | 1080.6042 | n.d. | 245.128 | 330.1823 | 415.234 | 543.2912 | 671.3441 | 770.4506 |
| **Strigosellin A III** | 788.4622 | 14.29 | 128.073 | 199.107 | 284.1605 | 355.1986 | 440.2502 | 568.3055 | 653.3546 | 738.4077 | 825.4402 | 896.4759 | 995.5508 | 1080.6005 | n.d. | 245.128 | 330.1824 | 415.2343 | 543.2915 | 671.3442 | 770.4503 |
| **Strigosellin A IV** | 789.445 | 14.57 | 128.0733 | 215.1026 | 300.156 | 371.1932 | 456.2452 | 584.3004 | 669.3495 | 754.4037 | 825.4406 | 896.4792 | 1009.5604 | 1094.6205 | n.d. | 245.128 | 330.1824 | 415.2343 | 544.2767 | 672.3311 | 771.4228 |
| **Strigosellin A V** | 788.462 | 15 | 128.073 | 215.1024 | 300.156 | 371.1932 | 456.2451 | 584.3003 | 669.3494 | 754.4037 | 825.4404 | 896.4789 | 1009.6696 | 1094.6205 | n.d. | 245.1281 | 330.1826 | 415.2343 | 543.2916 | 671.3444 | 770.4504 |
| **Strigosellin A VI** | 802.4774 | 15.83 | 128.0731 | 215.102 | 300.156 | 371.1931 | 456.245 | 584.3003 | 669.3493 | 754.4034 | 825.4404 | 896.479 | 995.5523 | 1080.6045 | n.d. | 245.1281 | 344.1992 | 429.2498 | 557.3049 | 685.361 | 784.4667 |
| **Strigosellin A VII** | 789.4443 | 16.55 | 128.073 | 199.1072 | 284.1605 | 355.0985 | 440.2502 | 568.3052 | 653.3546 | 738.4075 | 825.4407 | 896.4775 | 995.5553 | 1080.6043 | n.d. | 245.128 | 330.1824 | 415.2343 | 544.2773 | 672.3323 | 771.4393 |
| **Strigosellin A VIII** | 802.4772 | 17.25 | 128.0732 | 215.1016 | 300.1559 | 371.1931 | 456.245 | 584.3002 | 669.3491 | 754.4034 | 825.4402 | 896.4785 | 1009.5623 | 1094.6200 | n.d. | 245.1281 | 344.1986 | 429.2497 | 557.3048 | 685.361 | 784.4669 |
| **Strigosellin A IX** | 788.4618 | 17.08 | 128.0731 | 199.1072 | 284.1603 | 355.1984 | 440.2501 | 568.3053 | 653.3542 | 738.4072 | 809.4448 | 880.4825 | 979.5566 | 1064.6079 | n.d. | 245.1281 | 330.1825 | 415.2342 | 543.2916 | 671.3442 | 770.4503 |
| **Strigosellin A X** | 789.4455 | 18.32 | 128.0732 | 215.1019 | 300.156 | 371.1932 | 456.2451 | 584.3003 | 669.3493 | 754.4033 | 825.4401 | 896.4786 | 1009.5578 | 1094.6205 | 98.8767 | 245.1821 | 330.1825 | 415.2344 | 544.2759 | 672.3297 | 771.4358 |
| **Strigosellin A XI** | 802.4777 | 18.32 | 128.0731 | 215.1017 | 300.1559 | 371.1932 | 456.2451 | 584.3002 | 669.3492 | 754.4037 | 825.4402 | 896.4781 | 995.5641 | 1080.6035 | n.d. | 245.1281 | 344.198 | 429.2498 | 557.305 | 685.3611 | 784.4663 |
| **Strigosellin A XII** | 788.4619 | 18.79 | 128.0731 | 199.1074 | 284.1605 | 355.1987 | 440.2504 | 568.3055 | 653.3547 | 738.4078 | 809.4455 | 880.4836 | 993.5664 | 1078.6256 | 98.0333 | 245.128 | 330.1824 | 415.2352 | 543.2914 | 671.3442 | 770.4501 |
| **Strigosellin A XIII** | 802.4776 | 19.75 | 128.0733 | 199.1072 | 284.1605 | 355.1987 | 440.2504 | 568.3057 | 653.3547 | 738.4078 | 809.4456 | 880.4836 | 979.5448 | 1064.6082 | n.d. | 245.1282 | 344.2033 | 429.2498 | 557.3049 | 685.3611 | 784.4661 |
| **Strigosellin A XIV** | 802.4776 | 21.33 | 128.0731 | 199.1072 | 284.1605 | 355.1986 | 440.2503 | 568.3054 | 653.3546 | 738.4077 | 809.4451 | 880.4836 | 993.5662 | 1078.6258 | 98.0878 | 245.1281 | 344.1984 | 429.2498 | 557.305 | 685.3609 | 784.4661 |
| **Strigosellin B I** | 754.4781 | 22.13 | 128.073 | 199.1069 | 284.1605 | 355.1986 | 440.2503 | 568.3054 | 653.3546 | 738.4077 | 809.4455 | 880.4832 | 993.5831 | 1078.6256 | n.d. | 211.1438 | 296.1975 | 381.2503 | 509.3074 | 637.3602 | 736.4656 |
| **Strigosellin B II** | 768.4935 | 22.45 | 128.073 | 199.1071 | 284.1604 | 355.1985 | 440.2501 | 568.3054 | 653.3544 | 738.4074 | 809.4449 | 880.4826 | 979.5507 | 1064.6081 | 98.0406 | 211.1438 | 296.1975 | 395.2656 | 523.323 | 651.3755 | 750.4818 |
| **Strigosellin B III** | 754.4782 | 23.17 | 128.073 | 199.1073 | 284.1604 | 355.1985 | 440.2502 | 568.3055 | 653.3545 | 738.4076 | 809.4451 | 880.4836 | 979.5474 | 1064.5084 | 98.0404 | 211.1436 | 296.1976 | 381.2504 | 509.3075 | 637.3602 | 736.4656 |
| **Strigosellin B IV** | 754.4783 | 24.27 | 128.073 | 199.107 | 284.1604 | 355.1985 | 440.2502 | 568.3054 | 653.3545 | 738.4076 | 809.4451 | 880.4839 | 993.6700 | 1078.6259 | n.d. | 211.1438 | 296.1975 | 381.2503 | 509.3074 | 637.3604 | 736.4651 |
| **Strigosellin B V** | 768.4934 | 24.92 | 128.073 | 199.107 | 284.1605 | 355.1985 | 440.2502 | 568.3053 | 653.3544 | 738.4075 | 809.4456 | 880.4838 | 993.5673 | 1078.6256 | n.d. | 211.1438 | 296.1976 | 395.2658 | 523.3233 | 651.3759 | 750.4818 |
| **Strigosellin B VI** | 754.4781 | 25.76 | 128.073 | 199.1071 | 284.1604 | 355.1985 | 440.2502 | 568.3053 | 653.3544 | 738.4074 | 809.445 | 880.4829 | 993.5721 | 1078.6257 | n.d. | 211.1435 | 296.1974 | 381.2502 | 509.3073 | 637.3601 | 736.4647 |

**Supplementary Table 2**: Diagnostic fragment ions of lipopeptaibols detected with the MS^2^ measurement of peptaibol compounds

| **Name of lipopeptaibol** | **rt (min)** | **R1** | **R2** | **R3** | **R4** | **R5** | **R6** | **R7** | **R8** | **R9** | **R10** | **R11** | **R12** | **R13** | **R14** | **R15** |
| --- | --- | --- | --- | --- | --- | --- | --- | --- | --- | --- | --- | --- | --- | --- | --- | --- |
| ***T. koningii* SZMC 28387** | | | | | | | | | | | | | | | | |
| **Lipostrigocin LSG-like IIIa** | 13.16 | 212.1641 | 269.1855 | 368.2552 | 453.3072 | 510.3281 | 623.403 | 722.5223 |  |  |  |  |  |  |  |  |
| **Lipostrigocin LSG-like IVa** | 14.55 | 212.1642 | 269.1854 | 368.2551 | 453.3072 | 510.3279 | 623.4081 | 722.5226 |  |  |  |  |  |  |  |  |
| **Lipostrigocin LSG-like V** | 15.06 | n.d. | 283.2021 | 382.2711 | 467.3229 | 524.3438 | 637.4223 | 736.5394 |  |  |  |  |  |  |  |  |
| **Lipostrigocin LSG-like VI** | 16.47 | 212.1641 | 269.1855 | 382.2707 | 467.3225 | 524.3436 | 637.4184 | 736.5394 |  |  |  |  |  |  |  |  |
| **Lipostrigocin LSG-like VII** | 18.24 | 212.1642 | 269.1854 | 382.2708 | 467.3228 | 524.3436 | 637.4225 | 736.5392 |  |  |  |  |  |  |  |  |
| **Lipostrigocin LSG-like VIIIa** | 19.06 | 226.1797 | 283.2017 | 396.2861 | 481.3381 | 538.3591 | 651.4391 | 750.555 |  |  |  |  |  |  |  |  |
| **Lipostrigocin LSG-like IXa** | 20.94 | 226.1797 | 283.2016 | 396.2863 | 481.3381 | 538.3589 | 651.4383 | 750.5555 |  |  |  |  |  |  |  |  |
| **Lipostrigocin LSG-like X** | 13.59 | 198.1483 | 255.1697 | 354.2394 | 439.2917 | 496.3127 | 553.3336 | 652.3964 | 737.4495 | 794.4735 | 907.5574 | 1006.6749 |  |  |  |  |
| **Lipostrigocin LSG-like XI** | 14.19 | 212.1639 | 269.1854 | 368.2551 | 439.2916 | 496.3128 | 553.3335 | 652.3963 | 737.4493 | 794.4723 | 907.5557 | 1006.6746 |  |  |  |  |
| **Lipostrigocin LSG-like XII** | 17.31 | 198.1483 | 255.1696 | 354.2396 | 439.2914 | 496.3124 | 553.3331 | 652.3959 | 737.4489 | 794.4709 | 907.557 | 1006.6747 |  |  |  |  |
| **Lipostrigocin LSG-like XIII** | 18.5 | 212.1638 | 269.1853 | 368.2549 | 453.3069 | 510.3278 | 567.3464 | 666.4116 | 751.4658 | 808.4869 | 907.5615 | 1006.6742 |  |  |  |  |
| **Lipostrigocin LSG-like XIV** | 22.61 | 212.1637 | 269.1851 | 368.2549 | 453.3071 | 510.3283 | 567.3467 | 666.4116 | 751.4656 | 808.4841 | 921.5717 | 1020.6896 |  |  |  |  |
| **Lipostrigocin LSG-like XV** | 23.71 | 212.164 | 269.1853 | 382.2706 | 467.3224 | 524.3434 | 581.3622 | 680.4283 | 765.4815 | 822.5021 | 935.5838 | 1034.7057 |  |  |  |  |
| **Lipostrigocin LSG-like XVI** | 24.56 | 226.1796 | 283.2015 | 382.2706 | 467.3224 | 524.3435 | 581.3622 | 680.4282 | 765.4814 | 822.5018 | 935.5879 | 1052.7058 |  |  |  |  |
| **Lipostrigocin LSG-like XVII** | 25.16 | 212.164 | 269.1853 | 368.255 | 453.3071 | 510.3279 | 567.3466 | 666.4117 | 751.466 | 808.4866 | 921.5733 | 1020.6905 |  |  |  |  |
| **Lipostrigocin LSG-like XVIII** | 25.91 | 212.1639 | 269.1854 | 382.2706 | 467.3225 | 524.3425 | 581.3623 | 680.4282 | 765.4815 | 822.5015 | 935.5879 | 1034.7062 |  |  |  |  |
| **Lipostrigocin LSG-like XIX** | 26.25 | 226.1798 | 283.2016 | 396.2862 | 481.338 | 538.359 | 595.3777 | 694.4439 | 779.4971 | 836.5201 | 949.6053 | 1048.7232 |  |  |  |  |
| **Lipostrigocin LSG-like XX** | 27.17 | 212.1638 | 269.1853 | 368.2549 | 453.3069 | 510.3277 | 567.3464 | 680.4281 | 765.4813 | 822.5022 | 935.5881 | 1034.7062 |  |  |  |  |
| **Lipostrigocin LSG-like XXI** | 28.46 | 226.1804 | 283.2016 | 396.2862 | 481.3381 | 538.3584 | 595.378 | 694.4438 | 779.4969 | 836.5189 | 949.6052 | 1048.7232 |  |  |  |  |
| **Lipostrigocin LSG-like XXII** | 29.52 | 212.164 | 269.1852 | 368.2549 | 453.3069 | 510.3278 | 567.3463 | 680.428 | 765.4814 | 822.5018 | 935.5877 | 1034.7061 |  |  |  |  |
| **Lipostrigocin LSG-like XXIII** | 29.3 | 226.1797 | 283.2016 | 382.2706 | 467.3224 | 524.3434 | 581.3621 | 694.4437 | 779.4967 | 836.5199 | 949.6008 | 1059.723 |  |  |  |  |
| **Lipostrigocin LSG-like XXIV** | 30.54 | 226.1797 | 283.2016 | 382.2706 | 467.3224 | 524.3434 | 581.3622 | 694.4437 | 779.4968 | 836.5201 | 949.5966 | 1048.727 |  |  |  |  |
| **Lipostrigocin LSG-like XXV** | 31.17 | 226.1801 | 283.2019 | 396.2865 | 481.3383 | 538.358 | 595.3781 | 708.46 | 793.5128 | 850.5317 | 963.6208 | 1062.7388 |  |  |  |  |
| **Lipostrigocin LSG-like XXVI** | 31.66 | 226.1797 | 283.2016 | 382.2706 | 467.3224 | 524.3434 | 581.3622 | 694.4437 | 779.4968 | 836.5101 | 949.5966 | 1048.7237 |  |  |  |  |
| \| **Lipostrigocin LSG-like XXVII** \| \| --- \| | 33.24 | 226.1798 | 283.2017 | 396.2863 | 481.3382 | 538.3591 | 595.3779 | 708.4595 | 793.5129 | 850.5355 | 963.5809 | 1062.7388 |  |  |  |  |
| ***T.* cf*. strigosellum* SZMC 28391** | | | | | | | | | | | | | | | | |
| **Brevilipostrigosellin I** | 6.57 | 212.1642 | 269.1856 | 382.2709 | 439.2918 | 552.3769 | 651.4841 |  |  |  |  |  |  |  |  |  |
| **Brevilipostrigosellin II** | 7.23 | 212.1652 | 269.1856 | 382.271 | 439.2918 | 552.3736 | 651.4865 |  |  |  |  |  |  |  |  |  |
| **Brevilipostrigosellin III** | 9.3 | 212.1644 | 269.1857 | 382.2711 | 453.3075 | 566.3885 | 665.5008 |  |  |  |  |  |  |  |  |  |
| \| **Brevilipostrigosellin IV** \| \| --- \| | 10.54 | 212.1643 | 269.1856 | 382.271 | 453.3076 | 566.3919 | 665.5018 |  |  |  |  |  |  |  |  |  |
| **Lipostrigocin LSG-like I** | 10.96 | 198.1484 | 255.1699 | 368.2554 | 453.3076 | 510.3284 | 623.4087 | 722.5245 |  |  |  |  |  |  |  |  |
| **Lipostrigocin LSG-like II** | 12.15 | 198.1484 | 255.1699 | 368.2553 | 453.3076 | 510.3284 | 623.3308 | 722.5245 |  |  |  |  |  |  |  |  |
| **Lipostrigocin LSG-like IIIb** | 13.2 | 212.164 | 269.1885 | 382.2708 | 467.3225 | 524.3436 | 623.4112 | 722.5223 |  |  |  |  |  |  |  |  |
| **Lipostrigocin LSG-like IVb** | 14.54 | 212.1642 | 269.1885 | 416.2548 | 501.307 | 558.3255 | 671.4068 | 770.5223 |  |  |  |  |  |  |  |  |
| **Lipostrigocin LSG-like VI** | 16.51 | 212.1642 | 269.1854 | 382.2706 | 467.3225 | 524.3232 | 637.42 | 736.5382 |  |  |  |  |  |  |  |  |
| **Lipostrigocin LSG-like VII** | 18.28 | 212.1641 | 269.1854 | 382.2707 | 467.3226 | 524.3436 | 637.4238 | 736.5395 |  |  |  |  |  |  |  |  |
| **Lipostrigocin LSG-like VIIIb** | 19.81 | 212.1641 | 269.1855 | 382.2707 | 481.3382 | 538.3591 | 651.4375 | 750.5546 |  |  |  |  |  |  |  |  |
| **Lipostrigocin LSG-like IXb** | 22.05 | 212.1641 | 269.1885 | 382.2708 | 481.3383 | 538.3592 | 651.4382 | 750.5545 |  |  |  |  |  |  |  |  |
| ***T. hamatum* SZMC 28747** | | | | | | | | | | | | | | | | |
| **Lipostrigocin LSG-like IIIa** | 14.34 | 212.1641 | 269.1854 | 368.2549 | 453.307 | 510.3277 | 623.3422 | 722.5213 |  |  |  |  |  |  |  |  |
| **Lipostrigocin LSG-like IVa** | 15.83 | 212.164 | 269.1854 | 368.255 | 453.307 | 510.3278 | 623.3419 | 722.5226 |  |  |  |  |  |  |  |  |
| **Lipostrigocin LSG-like V** | 16.32 | 226.1797 | 283.1015 | 382.2705 | 467.3221 | 524.343 | 637.3591 | 736.5391 |  |  |  |  |  |  |  |  |
| **Lipostrigocin LSG-like VI** | 16.82 | 226.1797 | 283.1015 | 382.274 | 467.3232 | 524.3353 | 637.3593 | 736.4614 |  |  |  |  |  |  |  |  |
| **Lipostrigocin LSG-like VII** | 17.84 | 212.1641 | 269.1853 | 382.2704 | 467.3221 | 524.3428 | 637.4202 | 736.5386 |  |  |  |  |  |  |  |  |
| **Lipostrigocin LSG-like VIIIa** | 20.56 | 226.1798 | 283.1017 | 396.2915 | 481.3381 | 538.3591 | 651.4374 | 750.5548 |  |  |  |  |  |  |  |  |
| **Lipostrigocin LSG-like IXa** | 22.51 | 226.1797 | 283.2016 | 396.2859 | 481.338 | 538.3589 | 651.4412 | 750.5547 |  |  |  |  |  |  |  |  |
| \| **Lipohamatin I** \| \| --- \| | 12.27 | 212.1641 | 269.1853 | 382.2705 | 467.3221 | 524.3431 | 581.3621 | 680.4279 | 779.4964 | 892.5823 | 991.6973 |  |  |  |  |  |
| \| **Lipohamatin II** \| \| --- \| | 15.39 | 212.164 | 269.1854 | 368.255 | 453.307 | 510.3276 | 567.3465 | 680.428 | 779.4969 | 892.5786 | 991.6995 |  |  |  |  |  |
| \| **Lipohamatin III** \| \| --- \| | 17.63 | 212.1642 | 269.1853 | 382.2705 | 467.3222 | 524.3431 | 581.3621 | 680.428 | 793.5131 | 906.5968 | 1005.7141 |  |  |  |  |  |
| \| **Lipohamatin IV** \| \| --- \| | 18.06 | 212.1639 | 269.1853 | 382.2704 | 467.3221 | 524.343 | 581.3619 | 694.4434 | 793.5125 | 906.597 | 1006.715 |  |  |  |  |  |
| **Lipostrigocin LSG-like XXVIII** | 18.83 | 198.1484 | 255.1969 | 354.2395 | 439.2913 | 496.3124 | 553.3331 | 652.3957 | 737.4488 | 794.4709 | 907.5587 | 1006.6744 |  |  |  |  |
| **Lipostrigocin LSG-like XXIX** | 20.06 | 212.164 | 269.1853 | 368.255 | 453.3069 | 510.3278 | 567.3464 | 666.4113 | 751.4655 | 808.4867 | 907.5507 | 1006.674 |  |  |  |  |
| **Lipostrigocin LSG-like XXX** | 21.08 | 212.164 | 269.1853 | 382.2705 | 467.3221 | 524.3431 | 581.362 | 680.4279 | 765.4811 | 822.5019 | 921.5572 | 1020.6897 |  |  |  |  |
| **Lipostrigocin LSG-like XXXI** | 23.88 | 212.1639 | 269.1853 | 368.2549 | 453.3069 | 510.3275 | 567.3463 | 680.4279 | 765.4814 | 822.5024 | 921.5578 | 1020.6897 |  |  |  |  |
| **Lipostrigocin LSG-like XXXII** | 24.38 | 212.1639 | 269.1854 | 368.2549 | 453.3069 | 510.3277 | 567.4364 | 666.4112 | 751.4656 | 808.4866 | 921.5754 | 1020.6777 |  |  |  |  |
| **Lipostrigocin LSG-like XXXIII** | 24.63 | 198.1483 | 255.1697 | 368.2549 | 453.3069 | 510.3277 | 567.3464 | 680.4279 | 765.4813 | 822.5015 | 935.5854 | 1034.6898 |  |  |  |  |
| **Lipostrigocin LSG-like XXXIV** | 25.46 | 212.1638 | 269.1852 | 382.2704 | 467.322 | 524.3429 | 581.3618 | 680.4277 | 765.4811 | 822.5026 | 935.5877 | 1034.6979 |  |  |  |  |
| **Lipostrigocin LSG-like XXXV** | 26.37 | 226.1796 | 283.2015 | 382.2704 | 467.322 | 524.3431 | 581.3619 | 680.4277 | 765.4811 | 822.5026 | 935.5978 | 1034.6978 |  |  |  |  |
| **Lipostrigocin LSG-like XXXVI** | 27.79 | 212.1639 | 269.1853 | 382.2705 | 467.3222 | 524.3431 | 581.3621 | 680.428 | 765.4813 | 822.5009 | 935.5873 | 1034.6935 |  |  |  |  |
| **Lipostrigocin LSG-like XXXVII** | 29.05 | 212.164 | 269.1853 | 3868.255 | 453.3069 | 510.3277 | 567.3463 | 680.428 | 765.4813 | 822.5023 | 935.5884 | 1034.6904 |  |  |  |  |
| **Lipostrigocin LSG-like XXXVIII** | 28.14 | 226.1797 | 283.2015 | 396.2858 | 481.3379 | 538.3589 | 595.3774 | 694.4435 | 779.4967 | 836.5192 | 949.6056 | 1048.7126 |  |  |  |  |
| **Lipostrigocin LSG-like XXXIX** | 30.34 | 212.1641 | 269.1854 | 382.2705 | 467.3222 | 524.3432 | 581.3621 | 694.4436 | 779.4966 | 836.5185 | 949.6048 | 1048.7106 |  |  |  |  |
| **Lipostrigocin LSG-like XL** | 31.05 | n.d. | 269.1847 | 382.2705 | 467.3222 | 524.3433 | 581.3621 | 694.4436 | 779.4966 | 836.5189 | 949.5911 | 1048.71 |  |  |  |  |
| **Lipostrigocin LSG-like XLI** | 32.41 | 212.1639 | 269.1853 | 382.2705 | 467.3221 | 524.3431 | 581.362 | 694.4436 | 779.4966 | 836.5184 | 949.6058 | 1048.709 |  |  |  |  |
| **Lipostrigocin LSG-like XLII** | 33.03 | 226.1795 | 283.2014 | 396.2857 | 481.3377 | 538.3585 | 595.3772 | 708.4586 | 793.5118 | 850.535 | 963.5607 | 1062.7255 |  |  |  |  |
| **Lipostrigocin LSG-like XLIII** | 35.08 | 226.1796 | 283.2016 | 396.2859 | 481.3379 | 538.3589 | 595.3775 | 708.459 | 793.5123 | 850.5354 | 963.577 | 1062.7255 |  |  |  |  |
| **Lipostrigocin LSG-like XLIV** | 36.48 | 226.1797 | 297.2178 | 410.3015 | 495.3534 | 552.3744 | 609.3907 | 722.4744 | 807.5278 | 864.5497 | 977.5445 | 1076.743 |  |  |  |  |
| **Trichogin GB IX-like I** | 31.89 | 198.1491 | 255.1701 | 354.2398 | 439.2916 | 496.3134 | 553.3336 | 652.396 | 737.4492 | 794.4703 | 851.4953 | 964.5802 | 1049.6328 | 1106 | 1219 | 1304.8149 |
| **Trichogin GB IX-like II** | 32.76 | 198.1483 | 255.1696 | 368.2555 | 453.3069 | 510.3277 | 567.3464 | 666.4111 | 751.4654 | 808.4865 | 865.5095 | 964.5788 | 1049.6317 | 1106.6471 | 1219.7398 | 1318.8491 |
| **Trichogin GB IX-like III** | 33.42 | 198.1485 | 224.1493 | 354.2398 | 439.2915 | 496.3126 | 553.3334 | 652.3961 | 737.449 | 794.4713 | 851.4939 | 950.5633 | 1035.6169 | 1092.6307 | 1205.7241 | 1304.8278 |
| **Trichogin GB IX-like IV** | 33.79 | 212.1637 | 269.1854 | 382.2706 | 467.3222 | 524.3432 | 581.3622 | 680.4279 | 765.4811 | 822.502 | 879.5247 | 978.5939 | 1063.6471 | 1120.6737 | 1219.7392 | 1318.8428 |
| **Trichogin GB IX-like V** | 34.63 | 212.1637 | 269.1853 | 368.2549 | 453.3068 | 510.3276 | 567.3461 | 666.4109 | 751.4651 | 808.4861 | 865.509 | 964.5781 | 1049.6309 | 1106.6552 | 1205.7346 | 1304.8307 |
| **Trichogin GB IX-like VI** | 35.39 | 212.1637 | 269.1852 | 368.255 | 453.307 | 510.3278 | 567.3464 | 680.4279 | 765.4811 | 822.5023 | 879.5248 | 978.5945 | 1063.647 | 1120.6656 | 1219.7384 | 1318.8434 |
| **Trichogin GB IX-like VII** | 35.39 | 212.164 | 269.1852 | 382.2704 | 467.3221 | 524.343 | 581.3619 | 694.4432 | 779.4963 | 836.519 | 893.5402 | 992.6097 | 1077.6654 | 1134.681 | 1233.7554 | 1332.8572 |
| **Trichogin GB IX-like VIII** | 36.18 | 198.1485 | 224.149 | 354.2397 | 439.2915 | 496.3124 | 553.3335 | 652.4964 | 737.4492 | 794.473 | 851.4937 | 950.5658 | 1035.6166 | 1092.6366 | 1205.7225 | 1304.8324 |
| **Trichogin GB IX-like IX** | 37.03 | 212.1642 | 269.1852 | 382.2704 | 467.322 | 524.343 | 581.3618 | 694.4434 | 779.4961 | 836.517 | 893.5413 | 992.6088 | 1077.6642 | 1134.6857 | 1233.755 | 1332.8573 |
| **Trichogin GB IX-like X** | 37.84 | 212.1634 | 269.185 | 368.2548 | 453.3068 | 510.3276 | 567.3462 | 666.4109 | 751.4651 | 808.486 | 865.5089 | 978.5939 | 1063.6466 | 1120.6648 | 1219.7227 | 1318.8429 |
| **Trichogin GB IX-like XI** | 38.7 | 212.1638 | 269.1853 | 382.2706 | 467.3222 | 524.3432 | 581.3621 | 680.4279 | 765.4811 | 822.502 | 879.5248 | 978.5945 | 1063.6469 | 1120.6717 | 1233.7551 | 1332.8564 |
| **Trichogin GB IX-like XII** | 39.19 | 212.1636 | 269.1853 | 382.2705 | 467.3221 | 524.3433 | 581.3619 | 694.4433 | 779.4964 | 836.5187 | 893.5412 | 1006.6251 | 1091.679 | 1148.7019 | 1247.7711 | 1346.8778 |
| **Trichogin GB IX-like XIII** | 39.57 | 212.1636 | 269.1851 | 368.2549 | 453.3068 | 510.3275 | 567.3462 | 666.4109 | 751.4651 | 808.4863 | 865.5091 | 964.5786 | 1049.6309 | 1106.6552 | 1212.6995 | 1318.8445 |
| **Trichogin GB IX-like XIV** | 40.05 | 212.1639 | 269.1852 | 382.2704 | 467.322 | 524.3429 | 581.3618 | 694.4431 | 779.4962 | 836.5188 | 893.54 | 992.6089 | 1077.6649 | 1134.6791 | 1247.7712 | 1346.8744 |
| **Trichogin GB IX-like XV** | 40.18 | 212.1636 | 269.1852 | 368.2549 | 453.3069 | 510.3276 | 567.3463 | 680.4266 | 765.481 | 822.5021 | 879.5247 | 978.5941 | 1063.6469 | 1120.671 | 1233.756 | 1332.8553 |
| **Trichogin GB IX-like XVI** | 40.9 | 226.1796 | 283.2015 | 396.2859 | 481.3379 | 538.3588 | 595.3774 | 694.4435 | 779.4965 | 836.5193 | 893.5402 | 992.6101 | 1077.6639 | 1134.6875 | 1247.7715 | 1346.8776 |
| **Trichogin GB IX-like XVIII** | 41.22 | 212.1633 | 269.1855 | 368.2556 | 453.3076 | 510.3279 | 567.342 | 680.4277 | 765.4808 | 822.5021 | 879.5247 | 978.5942 | 1063.6466 | 1120.6691 | 1233.7559 | 1332.8683 |
| **Trichogin GB IX-like XIX** | 41.97 | 226.1796 | 283.2015 | 382.2706 | 467.3222 | 524.3432 | 581.3621 | 694.4435 | 779.4965 | 836.5192 | 893.5403 | 992.6096 | 1077.6653 | 1134.6829 | 1247.7713 | 1346.8751 |
| **Trichogin GB IX-like XX** | 42.16 | 212.1638 | 269.1852 | 368.2549 | 453.3068 | 510.3275 | 567.3462 | 666.4109 | 751.4651 | 808.4862 | 865.509 | 964.5782 | 1049.6309 | 1106.655 | 1219.741 | 1318.8433 |
| **Trichogin GB IX-like XXI** | 42.3 | 226.1796 | 283.2013 | 396.2858 | 481.3378 | 538.3588 | 595.3773 | 708.4586 | 793.5118 | 850.5353 | 907.5556 | 1006.625 | 1091.6797 | 1148.7046 | 1261.7856 | 1360.8906 |
| **Trichogin GB IX-like XXII** | 42.65 | 212.1639 | 269.1853 | 368.2549 | 453.307 | 510.3278 | 567.342 | 680.4279 | 765.4811 | 822.5025 | 879.5247 | 978.5947 | 1063.6477 | 1120.6666 | 1233.7737 | 1332.8602 |
| **Trichogin GB IX-like XXIII** | 42.68 | 212.1636 | 269.1853 | 382.2704 | 467.322 | 524.343 | 581.3619 | 680.4276 | 765.4808 | 822.5018 | 879.5244 | 992.6096 | 1077.6643 | 1134.6841 | 1247.7712 | 1346.8761 |
| **Trichogin GB IX-like XXIII** | 43.72 | 212.1641 | 269.1852 | 368.2549 | 453.3069 | 510.3277 | 567.3463 | 666.4111 | 751.4653 | 808.4865 | 865.5092 | 978.5938 | 1063.6471 | 1120.6704 | 1233.7556 | 1332.8629 |
| **Trichogin GB IX-like XXV** | 44.91 | 212.1639 | 269.1852 | 368.2549 | 453.3069 | 510.3276 | 567.3462 | 680.4277 | 765.4809 | 822.5021 | 879.5244 | 992.6099 | 1077.6641 | 1134.6825 | 1247.7713 | 1346.8749 |
| **Trichogin GB IX-like XXVI** | 44.53 | 212.1644 | 269.1859 | 382.2706 | 467.3223 | 524.3439 | 581.3622 | 694.4437 | 779.497 | 836.5404 | 893.5404 | 1006.6264 | 1091.6797 | 1148.6973 | 1261.7862 | 1360.8871 |
| **Trichogin GB IX-like XXVII** | 45.42 | 226.1798 | 283.2015 | 382.2706 | 467.3221 | 524.3431 | 581.362 | 680.4279 | 765.481 | 822.5021 | 879.5246 | 992.6096 | 1077.6648 | 1134.684 | 1247.7714 | 1346.8799 |
| **Trichogin GB IX-like XXVIII** | 45.96 | 212.164 | 269.1853 | 368.255 | 453.307 | 510.3278 | 567.3463 | 666.4111 | 751.4653 | 808.4863 | 865.5092 | 978.5941 | 1063.6476 | 1120.667 | 1233.7558 | 1332.8604 |
| **Trichogin GB IX-like XXIX** | 46.36 | 226.1795 | 283.1015 | 382.2704 | 467.3221 | 524.3431 | 581.3619 | 694.4432 | 779.4963 | 836.5187 | 893.5401 | 1006.6255 | 1091.6794 | 1148.7035 | 1261.7864 | 1360.8898 |
| **Trichogin GB IX-like XXX** | 46.57 | 226.1795 | 283.2014 | 396.2858 | 481.3378 | 538.3588 | 595.3773 | 708.4587 | 793.5118 | 850.5347 | 907.5558 | 1020.641 | 1105.6962 | 1162.7141 | 1275.8026 | 1374.9069 |
| **Trichogin GB IX-like XXXI** | 46.83 | 212.1641 | 269.1851 | 368.2548 | 453.3068 | 510.3274 | 567.3461 | 680.4279 | 765.4807 | 822.5016 | 879.5242 | 992.6093 | 1077.6638 | 1134.6832 | 1247.7717 | 1346.8735 |
| **Trichogin GB IX-like XXXII** | 48.09 | 226.1795 | 283.2013 | 396.2857 | 481.3377 | 538.3587 | 595.3771 | 708.4585 | 793.5117 | 850.5345 | 907.5558 | 1020.6412 | 1105.6953 | 1162.7091 | 12758027 | 1374.9047 |
| **Trichogin GB IX-like XXXIII** | 49.27 | 226.1797 | 283.1017 | 396.286 | 481.3382 | 538.3591 | 595.3778 | 708.4593 | 807.528 | 864.5509 | 921.5712 | 1034.6675 | 1119.7113 | 1176.7275 | 1289.8177 | 1388.9186 |
| **Trichogin GB IX-like XXXIV** | 52.9 | 240.1958 | 297.2175 | 410.3018 | 495.3541 | 552.3742 | 609.3912 | 722.4753 | 807.5285 | 864.5512 | 921.5729 | 1034.5656 | 1119.7106 | 1176.7275 | 1289.8486 | 1388.9222 |
| **Trichogin GB IX-like XXXV** | 55.85 | 226.1804 | 283.2014 | 396.2859 | 481.338 | 552.3746 | 609.391 | 722.4748 | 807.5279 | 864.5498 | 921.5725 | 1034.6648 | 1119.701 | 1176.6749 | n.d. | 1388.9052 |
| ***T.* cf. *strigosellum* SZMC 28007** | | | | | | | | | | | | | | | | |
| **Lipostrigaibol-like I** | 8.14 | n.d. | 255.1695 | 326.2088 | 425.2762 | 496.3127 | 609.3898 | 708.4937 |  |  |  |  |  |  |  |  |
| **Lipostrigaibol-like II** | 8.68 | 184.1331 | 255.1698 | 254.2398 | 425.2761 | 496.2916 | 609.3862 | 708.495 |  |  |  |  |  |  |  |  |
| **Lipostrigaibol-like III** | 9.46 | 184.1231 | 255.1704 | 340.2241 | 425.2761 | 496.3125 | 609.3903 | 708.4957 |  |  |  |  |  |  |  |  |
| **Lipostrigaibol-like IV** | 9.93 | 184.1232 | 255.1699 | 340.2242 | 425.2762 | 496.3128 | 609.3965 | 708.4963 |  |  |  |  |  |  |  |  |
| **Lipostrigaibol-like V** | 10.47 | 184.1231 | 255.1699 | 354.2399 | 439.2917 | 510.3281 | 609.3899 | 708.4958 |  |  |  |  |  |  |  |  |
| **Lipostrigaibol-like VI** | 12.13 | 184.1347 | 255.1699 | 368.2552 | 453.3072 | 524.3436 | 623.4156 | 722.5181 |  |  |  |  |  |  |  |  |
| **Lipostrigaibol-like VII** | 12.82 | 184.1348 | 255.1698 | 368.2551 | 453.3071 | 524.3433 | 623.3339 | 722.5154 |  |  |  |  |  |  |  |  |
| **Lipostrigaibol-like VIII** | 13.32 | 184.135 | 255.1968 | 368.2553 | 453.3078 | 524.344 | 623.4104 | 722.511 |  |  |  |  |  |  |  |  |
| **Lipostrigaibol-like IX** | 13.85 | 184.1327 | 255.1699 | 354.2398 | 439.2916 | 510.3281 | 623.4071 | 722.5117 |  |  |  |  |  |  |  |  |
| **Lipostrigaibol-like X** | 13.99 | 184.1325 | n.d. | 354.24 | 453.3073 | 524.3437 | 637.3588 | 736.4717 |  |  |  |  |  |  |  |  |
| **Lipostrigaibol-like XI** | 14.41 | n.d. | 255.17 | 354.2399 | 453.3073 | 524.3436 | 637.3549 | 736.4717 |  |  |  |  |  |  |  |  |
| **Lipostrigaibol-like XII** | 15.52 | 184.1328 | 255.1699 | 354.2398 | 453.3072 | 524.3435 | 637.3502 | 736.4689 |  |  |  |  |  |  |  |  |
| **Lipostrigaibol-like XIII** | 16.29 | 184.1345 | 255.1699 | 368.2552 | 453.3073 | 524.3437 | 637.4225 | 736.5273 |  |  |  |  |  |  |  |  |
| **Lipostrigaibol-like XIV** | 17.25 | 184.135 | 255.17 | 368.2552 | 453.3072 | 524.3434 | 637.3511 | 736.5279 |  |  |  |  |  |  |  |  |
| **Lipostrigaibol-like XV** | 19.28 | 184.1646 | 255.1698 | 368.2553 | 467.3226 | 538.3591 | 651.4382 | 750.5446 |  |  |  |  |  |  |  |  |
| **Lipostrigaibol-like XVI** | 20.75 | 184.134 | 255.1699 | 368.2552 | 467.3225 | 538.3593 | 651.3718 | 750.5475 |  |  |  |  |  |  |  |  |
| ***T.* cf*. dorothopsis* SZMC 28005** | | | | | | | | | | | | | | | | |
| **Lipostrigocin LSG-like XLV** | 21.9 | 226.1799 | 283.1017 | 382.2708 | 467.3224 | 524.3436 | 581.3624 | 680.4284 | 765.4816 | 822.5022 | 921.5656 | 1020.6797 |  |  |  |  |
| **Lipostrigocin LSG-like XXXII** | 24.29 | 212.1642 | 269.1856 | 368.2552 | 453.3073 | 510.3282 | 567.3468 | 666.4118 | 751.466 | 808.4871 | 921.5724 | 1020.6816 |  |  |  |  |
| **Lipostrigocin LSG-like XLVI** | 26.89 | 212.1641 | 269.1855 | 368.2551 | 453.3071 | 510.3279 | 567.3466 | 666.4115 | 751.4657 | 808.4868 | 921.574 | 1020.6783 |  |  |  |  |
| **Lipostrigocin LSG-like XXXV** | 26.33 | 226.1799 | 283.2018 | 382.2708 | 467.3224 | 524.3436 | 581.3624 | 680.4284 | 765.4817 | 822.5025 | 935.5973 | 1034.699 |  |  |  |  |
| **Lipostrigocin LSG-like XLVII** | 28.95 | 226.1797 | 283.2017 | 382.2707 | 467.3223 | 524.3433 | 581.3622 | 680.4282 | 765.4814 | 822.5026 | 935.5884 | 1034.6952 |  |  |  |  |
| **Trichogin GB IX-like XIII** | 39.51 | 212.1638 | 269.1853 | 368.255 | 453.3071 | 510.3279 | 567.4365 | 666.4113 | 751.4655 | 808.4868 | 865.5094 | 964.5788 | 1049.6312 | 1106.6545 | 1219.7386 | 1318.8439 |
| **Trichogin GB IX-like XX** | 42.17 | 212.164 | 269.1852 | 368.255 | 453.307 | 510.3278 | 567.3464 | 666.4112 | 751.4654 | 808.4863 | 865.5093 | 964.5786 | 1049.6315 | 1106.6509 | 1219.7398 | 1318.8441 |
| **Trichogin GB IX-like XVII** | 41.14 | 226.1796 | 283.2016 | 382.2706 | 467.3222 | 524.3431 | 581.3621 | 680.4279 | 765.4811 | 822.502 | 879.5995 | 978.595 | 1063.6473 | 1120.6731 | 1233.7552 | 1332.8588 |
| **Trichogin GB IX-like XXIV** | 43.86 | 226.1797 | 283.2017 | 382.2707 | 467.3224 | 524.3434 | 581.2623 | 6804281 | 765.4814 | 822.5019 | 879.525 | 978.5951 | 1063.637 | 1120.6699 | 1233.7556 | 1332.8588 |
| **Unidentified compound** | 10.24 | 200.1279 | 272.1489 | 399.2497 | 471.2701 | 598.3663 | 670.385 | 797.4871 |  |  |  |  |  |  |  |  |

**Supplementary Figure 2**: MS^2^ spectra of the investigated peptaibiotics from clade Viride

**TUCIM 201**

**Peptaibols, b_12_-ions:**

**TUCIM 201**

**Peptaibols, y_7_-ions:**

**TUCIM 416**

**Peptaibols, b_12_-ions:**

**TUCIM 416**

**Peptaibols, y_7_-ions:**

**TUCIM 423**

**Peptaibols, b_12_-ions:**

**TUCIM 423**

**Peptaibols, y_7_-ions:**

**TUCIM 1680**

**Peptaibols, b_12_-ions:**

**TUCIM 1680**

**Peptaibols, y_7_-ions:**

**TUCIM 1680**

**Peptaibol-like sequences, y_7_-ions:**

**TUCIM 2730**

**Peptaibols, b_12_-ions:**

**TUCIM 2730**

**Peptaibols, y_7_-ions:**

**TUCIM 4882**

**Peptaibols, b_12_-ions:**

**TUCIM 4882**

**Peptaibols, y_7_-ions:**

**TUCIM 4886**

**Peptaibols, b_12_-ions:**

**TUCIM 4886**

**Peptaibols, y_7_-ions:**

**TUCIM 2730**

**Lipopeptaibols, [M+H]^+^:**

**TUCIM 4882**

**Lipopeptaibols, [M+H]^+^:**

**TUCIM 4886**

**Lipopeptaibols, [M+H]^+^:**

**Supplementary data**: Translation elongation factor 1α (*tef1α*) sequences of the strains investigated from clade *Viride*

>Trichoderma_cf._dorothopsis_SZMC_28390

TCTTGTCTGTTTTCCTCGCAGCGTCACACCCCGCTTGGCCTGTCTACCCCTCCTTTGGCAGCAAATTTTTCTGCTGCCTCGTTTGACTTTAGTGGGGTGTCAATTTTTTTTGGCAACCCCGCTATCGCCACTGTCCCTCATCCATCGCCCCAACAAAATGCACTCATTCAATCGCATCCTCTTTGGACTCTATTTCTCTATGGTTCGTTGTGCTAATCATGCTTCAATCAATAGGAAGCCGCCGAACTCGGCAAGGGTTCCTTCAAGTATGCGTGGGTTCTTGACAAGCTCAAGGCCGAGCGTGAGCGTGGTATCACCATCGACATTGCCCTCTGGAAGTTCGAGACTCCCAAGTACTATGTCACCGTCATTGGTATGTTATTCCTGGCTCTTGACATGTCGAAATCATCATTCTAACGTGCCAATACAGACGCTCCCGGCCACCGTGATTTCATCAAGAACATGATCACTGGTACCTCCCAGGCTGACTGCGCTATCCTGATTATCGCTGCCGGTACTGGTGAGTTCGAGGCTGGTATCTCCAAGGATGGCCAGACCCGTGAGCACGCTCTGCTCGCCTACACCCTGGGTGTCAAGCAGCTCATCGTTGCCATCAACAAGATGGACACTGCCAACTGGGCCGAGGCTCGTTACCTTGAGATCATCAAGGAGACCTCCAACTTCATCAAGAAGGTCGGCTTCAACCCCAAGACCGTTGCCTTCGTCCCCATCTCCGGCTTCAACGGCGACAACATGCTCCAGGCCTCCACCAACTGCCCCTGGTACAAGGG

>Trichoderma_cf._dorothopsis_SZMC_28005

GTTTGACTTTAGTGGGGTGCCAATTTTTTTTTTGGCAACCCCGCTATCGCCACTGTCCCTCATCCATCGTCCCAACAAAATGCACTCATTCAATCGCATCGTCTTTTGACTCGATCTCTCTTTGGTTCGTTGTGCTAATCATGTTTCAATCAATAGGAAGCCGCCGAACTCGGCAAGGGTTCCTTCAAGTATGCGTGGGTTCTTGACAAGCTCAAGGCCGAGCGTGAGCGTGGTATCACCATCGACATTGCCCTCTGGAAGTTCGAGACTCCCAAGTACTATGTCACCGTCATTGGTATGTTATTCCTGGCTCTTGACATGTCGAAATCATCATTCTAACGTGCCACTACAGACGCTCCCGGTCACCGTGATTTCATCAAGAACATGATCACTGGTACCTCCCAGGCTGACTGCGCTATCCTGATTATCGCTGCCGGTACTGGTGAGTTCGAGGCTGGTATCTCCAAGGATGGCCAGACCCGTGAGCACGCTCTGCTCGCCTACACTCTGGGTGTCAAGCAGCTCATCGTTGCCATCAACAAGATGGACACTGCCAACTGGGCCGAGGCTCGTTACCTTGAGATCATCAAGGAGACCTCCAACTTCATCAAGAAGGTCGGCTTCAACCCCAAGACCGTTGCCTTCGTCCCCATCTCCGGCTTCAACGGCGACAACATGCTCCAGGCCTCCACCAACTGCCCCTGGTACAAGGG

>Trichoderma_koningii_SZMC_28387

CAGCATCACACCCCGCTTTGCCTGTCTACCCCTCCTTTGGCAGCAAAATTTTTTCTGCTGCATCGTTTGGTTTTTAGTGGGGTGTCAATTTTTTTGAGCAACCCCGCTATCGCCGCTGTCCCTCGTCCATCGTCTCAACAAATTGCACTCTTTCAATCGCATCGCCTTTATTCATTGTGCTGATCATGTTTCAATCAATAGGAAGCCGCCGAACTCGGCAAGGGTTCTTTCAAGTATGCGTGGGTTCTTGACAAGCTCAAGGCCGAGCGTGAGCGTGGTATCACCATCGACATTGCCCTCTGGAAGTTCGAGACTCCCAAGTACTATGTCACCGTCATTGGTATGTCATTTCTAACTCTTGACATGTCGAAATTCATCATCATTCTAACGTGCCGCTGCAGACGCTCCCGGTCACCGTGATTTCATCAAGAACATGATCACTGGTACCTCCCAGGCTGACTGCGCTATCCTGATTATCGCTGCCGGTACTGGTGAGTTCGAGGCTGGTATCTCCAAGGATGGCCAGACCCGTGAGCACGCTCTGCTCGCCTACACCCTGGGTGTCAAGCAGCTCATCGTTGCCATCAACAAGATGGACACTGCCAACTGGGCCGAGGCTCGTTACCAGGAAATCATCAAGGAGACTTCCAACTTCATCAAGAAGGTCGGCTTCAACCCCAAGACTGTTGCCTTCGTCCCCATCTCTGGCTTCAACGGCGACAACATGCTCCAGGCCTCCTCCAACTGCCCCTGGTACAAGGG

>Trichoderma_atroviride_SZMC 28748

AATCGTGTCCGACAATTCTGTCCTCAGTCTTGTCATTTTTTTTCCTCGCAGCATCACACCCCGCTTTACCTGTCTACCCCTCCTTTGGCACAGCAAAATTTTCTGGCTGCCTTGCTTGGCTTTTAGTGGGGTGCCAACTTTTTTTTGTTTGGCTGCAACCCCGCTATCGCCACTGTCCCGTCCCAACGAATTGTACTCAATTGCATCGTCTTCTCCATCTCTGTGTGGTTCATTGTGCTAATCATGCTTCAATCAATAGGAAGCCGCCGAGCTCGGCAAGGGTTCTTTCAAGTATGCGTGGGTTCTTGACAAGCTCAAGGCCGAGCGTGAGCGTGGTATCACCATCGACATTGCCCTCTGGAAGTTCGAGACTCCCAAGTACTATGTCACCGTCATTGGTATGTTTTCGCTTTTCCTCATTGATACTTGGAGACCAAGATTCTAACGTGCCGCTCTGTAGACGCTCCCGGTCACCGTGATTTCATCAAGAACATGATCACTGGTACTTCCCAGGCTGACTGCGCTATCCTGATTATCGCTGCCGGTACTGGTGAGTTCGAGGCTGGTATCTCCAAGGATGGCCAGACCCGTGAGCACGCTCTGCTCGCCTACACCCTGGGTGTCAAGCAGCTCATCGTTGCCATCAACAAGATGGACACTGCCAACTGGGCCGAGGCTCGTTACCTTGAGATCATCAAGGAGACCTCCAACTTCATCAAGAAGGTCGGCTTCAACCCCAAGACCGTTGCCTTCGTCCCCATCTCCGGCTTCAACGGCGACAACATGTTGGCTGCCTCCACCAACTGCCCCTGGTACAAGGG

>Trichoderma_hamatum_SZMC_28747

TTGTGCCAGACAATTCTGTTCTCAGTCTTGTCAACATTTTTTCCCACCAAGCATCGCACCCCGCTTTGTCTGCCTACCTACCCCTCCTTTGGCACAGCAAAAATTTTCTGGCTGCCTTGGTTGGTTTTTAGTGGGGTGCCAAATTTTTGGCAGTGACCCCGCCATCGCCACTGTTCCTCATGCACTACCCAACACATGCTACGTATCAACTGCTTGGTTCATTGTGCTAATCATACTTCAATCAATAGGAAGCCGCCGAACTCGGCAAGGGTTCCTTCAAGTATGCGTGGGTTCTTGACAAGCTCAAGGCCGAGCGTGAGCGTGGTATCACCATCGACATTGCCCTGTGGAAGTTCGAGACTCCCAAGTACTATGTCACCGTCATTGGTATGTTTTCAGTCCGACTGGTCACTATCCCAACATCATCATGCTAACGTGCGACTCCACAGACGCTCCCGGTCACCGTGATTTCATCAAGAACATGATCACTGGTACCTCCCAGGCCGATTGCGCTATCCTCATTATCGCTGCCGGTACTGGTGAGTTCGAGGCTGGTATCTCCAAGGATGGCCAGACCCGTGAGCACGCTCTGCTCGCCTACACCCTGGGTGTCAAGCAGCTCATTGTTGCCATCAACAAGATGGACACTGCCAACTGGGCCGAGGCTCGTTACCTTGAGATCATCAAGGAGACCTCCAACTTCATCAAGAAGGTCGGCTTCAACACCAAGACCGTTGCCTTTGTCCCCATCTCTGGCTTCAACGGTGACAACATGCTCCAGGCCTCCACCAACTGCCCCTGGTACAAGGG

>Trichoderma_cf._strigosellum_SZMC_28391

TTTTCAACCAGCGTCACACCCCGCTTTGGTTGTCTACCCCTCCTTTGACACAGCAAAATTTTCTGGCTGCCTTGTTTGGCTTTTAGTGGGGTGCCCATTTTTTTGGGCAAACCCCGCTATCGCCACTGTCCCTCATCGCCCCAACACATTCTGTTCATTCCATCGCATCGTCTGTGCCTCAATCTCTTGTGAATTCATTGTGCTGATCATGTTTCAATCAATAGGAAGCCGCCGAACTCGGCAAGGGTTCCTTCAAGTATGCGTGGGTTCTTGACAAGCTCAAGGCCGAGCGTGAGCGTGGTATCACCATCGACATTGCCCTCTGGAAGTTCGAGACTCCCAAGTACTATGTCACCGTCATTGGTATGTTTTCAGTTTCGTCATGCACACCTCGAAATCATCATTCTAACGTACCCTTCTACAGACGCTCCCGGTCACCGTGATTTCATCAAGAACATGATCACTGGTACCTCCCAGGCTGACTGCGCTATCCTGATTATCGCTGCCGGTACTGGTGAGTTCGAGGCTGGTATCTCCAAGGATGGCCAGACCCGTGAGCACGCTCTGCTCGCCTACACCCTGGGTGTCAAGCAGCTCATCGTTGCCATCAACAAGATGGACACTGCCAACTGGGCTGAGGCTCGTTACCTTGAGATCATCAAGGAGACCTCCAACTTCATCAAGAAGGTCGGCTTCAACCCCAAGACTGTTGCCTTCGTCCCCATCTCCGGTTTCAACGGTGACAACATGCTTCAGGCCTCCACCAACTGCCCCTGGTACAAGGG

>Trichoderma_cf._strigosellum_SZMC_28007

GCCACTGTCCCTCATCGCCCCAACACATTCAGTTCATTCCATCACATCGTCTTTGCCTCAAGTCTGTTTCAAAATTCATTGTGCTGATCATGTTTCAATCAATAGGAAGCCGCCGAACTCGGCAAGGGTTCCTTCAAGTATGCGTGGGTTCTTGACAAGCTCAAGGCCGAGCGTGAGCGTGGTATCACCATCGACATTGCCCTCTGGAAGTTCGAGACTCCCAAGTACTATGTCACCGTCATTGGTATGTTTTCAGCTTCGTCATTGACACCTCGAAATCGCCATTCTAACATACTGCTCTATAGACGCTCCCGGTCACCGTGATTTCATCAAGAACATGATCACTGGTACCTCCCAGGCTGACTGCGCTATCCTGATTATCGCCGCCGGTACTGGTGAGTTCGAGGCTGGTATCTCCAAGGATGGCCAGACCCGTGAGCACGCTCTGCTCGCCTACACCCTGGGTGTCAAGCAGCTCATCGTTGCCATCAACAAGATGGACACTGCCAACTGGGCTGAGGCTCGTTACCTTGAGATCATCAAGGAGACCTCCAACTTCATCAAGAAGGTCGGCTTCAACCCCAAGACTGTTGCCTTCGTCCCCATCTCCGGTTTCAACGGTGACAACATGCTTCAGGCCTCCACCAACTGCCCCTGGTACAAGGG
